# A trade-off in evolution: the adaptive landscape of spiders without venom glands

**DOI:** 10.1093/gigascience/giae048

**Published:** 2024-08-05

**Authors:** Yiming Zhang, Yunxiao Shen, Pengyu Jin, Bingyue Zhu, Yejie Lin, Tongyao Jiang, Xianting Huang, Yang Wang, Zhe Zhao, Shuqiang Li

**Affiliations:** Key Laboratory of Zoological Systematics and Evolution, Institute of Zoology, Chinese Academy of Sciences, Beijing 100101, China; Hebei Key Laboratory of Animal Diversity, College of Life Sciences, Langfang Normal University, Langfang 065000, China; University of Chinese Academy of Sciences, Beijing 101408, China; Key Laboratory of Zoological Systematics and Evolution, Institute of Zoology, Chinese Academy of Sciences, Beijing 100101, China; University of Chinese Academy of Sciences, Beijing 101408, China; Key Laboratory of Zoological Systematics and Evolution, Institute of Zoology, Chinese Academy of Sciences, Beijing 100101, China; Key Laboratory of Zoological Systematics and Evolution, Institute of Zoology, Chinese Academy of Sciences, Beijing 100101, China; University of Chinese Academy of Sciences, Beijing 101408, China; Key Laboratory of Zoological Systematics and Evolution, Institute of Zoology, Chinese Academy of Sciences, Beijing 100101, China; Hebei Key Laboratory of Animal Diversity, College of Life Sciences, Langfang Normal University, Langfang 065000, China; Key Laboratory of Zoological Systematics and Evolution, Institute of Zoology, Chinese Academy of Sciences, Beijing 100101, China; University of Chinese Academy of Sciences, Beijing 101408, China; Key Laboratory of Zoological Systematics and Evolution, Institute of Zoology, Chinese Academy of Sciences, Beijing 100101, China; University of Chinese Academy of Sciences, Beijing 101408, China; Key Laboratory of Zoological Systematics and Evolution, Institute of Zoology, Chinese Academy of Sciences, Beijing 100101, China; University of Chinese Academy of Sciences, Beijing 101408, China; Key Laboratory of Zoological Systematics and Evolution, Institute of Zoology, Chinese Academy of Sciences, Beijing 100101, China; Key Laboratory of Zoological Systematics and Evolution, Institute of Zoology, Chinese Academy of Sciences, Beijing 100101, China

**Keywords:** venom gland deficient, adaptive evolution, genome, *Octonoba sinensis*

## Abstract

**Background:**

Venom glands play a key role in the predation and defense strategies of almost all spider groups. However, the spider family Uloboridae lacks venom glands and has evolved an adaptive strategy: they excessively wrap their prey directly with spider silk instead of paralyzing it first with toxins. This shift in survival strategy is very fascinating, but the genetic underpinnings behind it are poorly understood.

**Results:**

Spanning multiple spider groups, we conducted multiomics analyses on *Octonoba sinensis* and described the adaptive evolution of the Uloboridae family at the genome level. We observed the coding genes of *myosin* and *twitchin* in muscles are under positive selection, energy metabolism functions are enhanced, and gene families related to tracheal development and tissue mechanical strength are expanded or emerged, all of which are related to the unique anatomical structure and predatory behavior of spiders in the family Uloboridae. In addition, we also scanned the elements that are absent or under relaxed purifying selection, as well as toxin gene homologs in the genomes of 2 species in this family. The results show that the absence of regions and regions under relaxed selection in these spiders’ genomes are concentrated in areas related to development and neurosystem. The search for toxin homologs reveals possible gene function shift between toxins and nontoxins and confirms that there are no reliable toxin genes in the genome of this group.

**Conclusions:**

This study demonstrates the trade-off between different predation strategies in spiders, using either chemical or physical strategy, and provides insights into the possible mechanism underlying this trade-off. Venomless spiders need to mobilize multiple developmental and metabolic pathways related to motor function and limb mechanical strength to cover the decline in adaptability caused by the absence of venom glands.

## Introduction

“Venomous” is a common way that people perceive spiders (Araneae). The toxic, painful, and even fatal bite is always frightening. Indeed, almost all spiders are venomous. In the earliest diverging suborder Mesothelae, fangs that could inject venom are already present [1]. Some highly toxic species make spiders even more notorious. As an important means of hunting and defense, the toxin system gives spiders an outstanding advantage in environmental suitability and has allowed them to spread throughout the world. Of course, there are always exceptions, and some outliers are believed to be lacking venom glands. Currently, known spiders without venom grands include *Holarchaea* (2 species) [2–4] and the entire family Uloboridae, with the latter being the most prosperous group [5]. Compared to *Holarchaea*, which has a smaller body size, fewer species, and limited distribution, the family Uloboridae provides a satisfactory model for us to study the evolution of an important synapomorphy of spiders and the adaptation strategy brought about by the loss of important functional traits.

Due to the absence of venom glands, the predation methods of uloborids are also relatively specialized and excessive. Generally speaking, using venom to paralyze prey is an effective chemical attack strategy. However, many kinds of spiders integrate both chemical attack and physical attack strategies. Certain species within the Araneoidea family utilize entanglement initially to restrain larger prey before a venomous final strike [6]. Although this predatory tactic may alleviate the selective pressure associated with venom usage, entanglement appears rudimentary in comparison to the prey-wrapping behavior exhibited by uloborids. In Uloboridae, this physical attack as the sole means of attack can span from a few minutes to nearly an hour, with the spider silk utilized sometimes exceeding a hundred meters in length [7–9]. Therefore, considerable physical endurance is indispensable for the successful execution of this predatory tactic. Previous anatomical records indicate uloborids have well-developed trachea [10], and many branches of the trachea extend into the prosoma and appendages [11]. However, there is no relevant research that can link these adaptive characteristics to the venom gland deficiency of this group.

**Figure 1:**
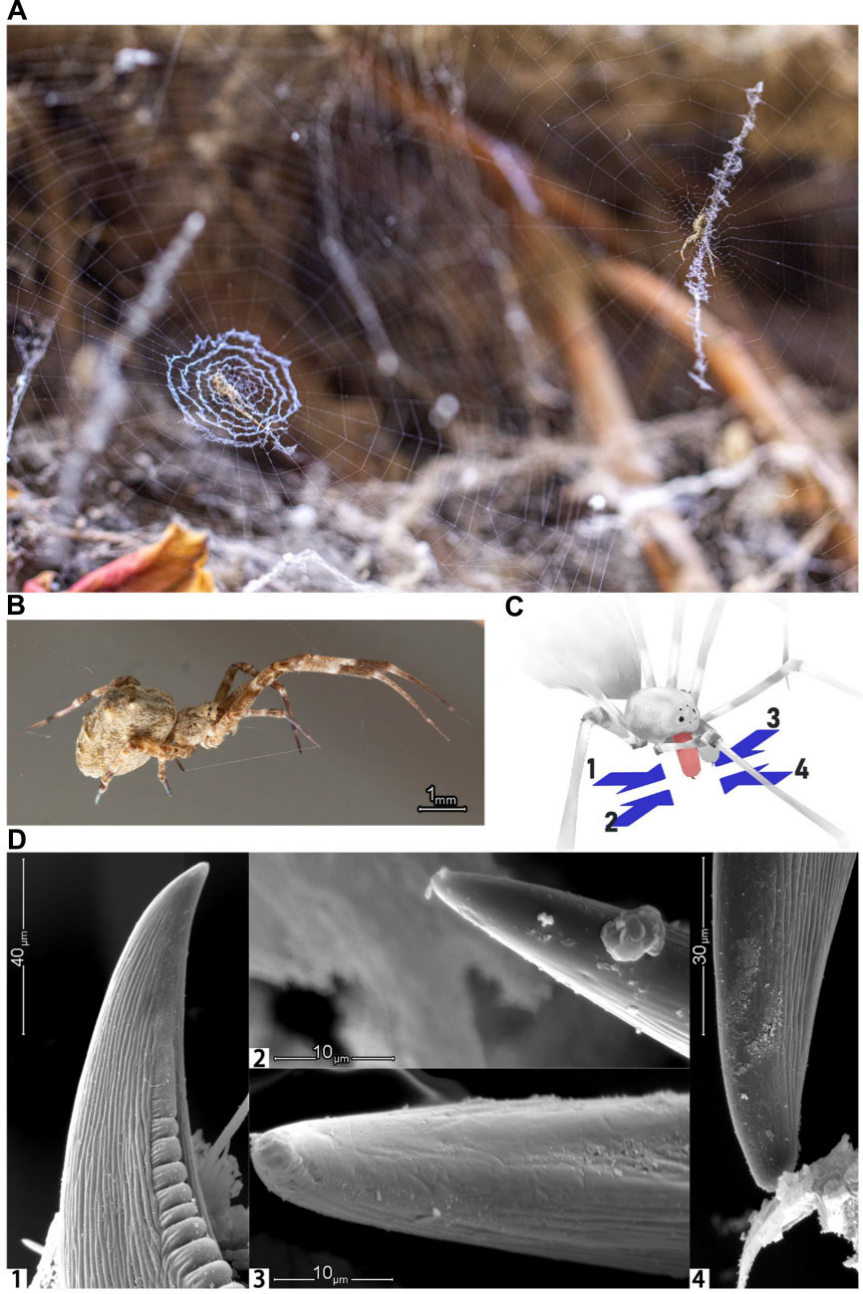
Observations of *Octonoba sinensis*. (A) Two specimens of *O. sinensis* on their orb webs in their natural environment. (B) Adult female of *O. sinensis*. (C) The perspective diagram in (D), with arrows numbered 1–4 indicating the shooting angles of the 4 images in (D). (D) Scanning electron microscope image of *O. sinensis* fangs.

The spider *Octonoba sinensis* (NCBI:txid198412) belongs to the family Uloboridae. Their body size is relatively larger than other members of the family [12], and this species is widely distributed in East Asia, Southeast Asia, and North America [2]. Their habitat is close to human buildings, and the populations are large, so they can be easily collected in cities (Fig. 1A, B). These aforementioned characteristics make it an ideal model species of the Uloboridae to study the biological characteristics of this family.

In this study, we generated a chromosome-level genome assembly of *O. sinensis*. High-quality annotation was performed on the genomes of *Pardosa pseudoannulata* and *Dolomedes plantarius*, as well as optimization of annotation for the genomes of *Uloborus diversus* and *Latrodectus elegans*. Furthermore, we assembled a full-protein reference for *Deinopis* sp. using transcriptome data. By leveraging multiomics datasets from various spider species, we explored the molecular basis of the unique adaptive strategies in the family Uloboridae. In the selection pressure analysis, we found that some important genes related to muscle movement have undergone significant positive selection. In gene family analysis, we observed the absence or relaxation in selection pressure of toxins and developmental related genes. Gene families related to tracheal development, skeletal development, and tissue force-bearing structures have significantly expanded or emerged in the genomes of this group. Moreover, energy metabolism–related genes exhibit high expression, and enzyme activity in related pathways is significantly increased. These all provide a plausible explanation for the improvement in respiratory efficiency and the well-developed tracheal system observed.

## Results

### Prey-wrapping behavior observations and fang morphology

Through our observations, like other species in the family Uloboridae, *O. sinensis* only arrest their prey through extensive silk wrapping. The time usually exceeds 3 minutes (sometimes even 8–9 minutes), during which there may be several brief breaks (Additional Files 1–3). This time is much higher than the previously recorded wrapping time of other spider species (Araneoidea, 10.1 ± 1.2 seconds for flies, 43.2 ± 6.7 seconds for dragonflies) [6].

In addition, for the first time, we examined the fangs of *O. sinensis* from multiple angles with a scanning electron microscope (SEM). Generally speaking, if a species has venom glands, a channel opening for injecting venom should be found on the fangs [1, 13]. We did not observe this in *O. sinensis*, which further provides evidence that uloborids are not equipped to deliver venom (Fig. 1C, D).

### Genome assembly and annotation

**Figure 2:**
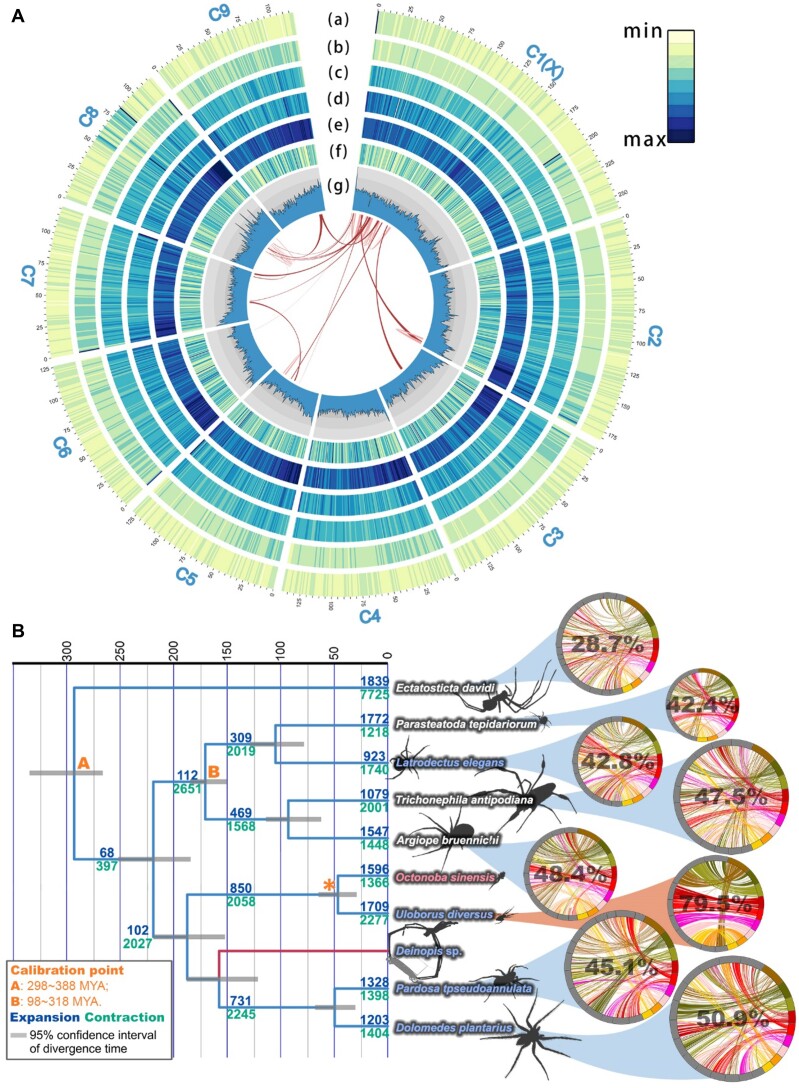
Genome and comparative genomic analysis results from *Octonoba sinensis*. (A) Assembly and structural annotation of the *O. sinensis* genome. The distribution of different elements is marked with lowercase letters from the outside to the inside in the circle diagram, a: gene; b: SINE, c: LINE, d: LTR; e: DNA transposon; f: highly conserved elements (HCEs); g: GC content. The inner lines of the circle graph are collineation, dark red lines are collineation between chromosomes, and pink lines represent collineation within the chromosome. (B) Phylogeny of multiple spider species. All nodes received 100% bootstrap support. New annotated genomes provided in this study are marked in light blue, and new genome assemblies and annotations are marked in light red. The 95% confidence interval of the divergence time is represented by the gray bars on the phylogeny, and the number of expanded and contracted gene families at each node is represented by light blue and light green, respectively. The asterisk indicates the foreground branch in the selection pressure analysis. The branch with only transcriptome data cannot deploy CAFE analysis and is highlighted by a red branch. The collinearity between *O. sinensis* and other spider species is visualized through circle diagrams positioned adjacent to each respective species. The proportion of collinearity segments within the *O. sinensis* genome is emphasized in bold percentages.

We assembled an *O. sinensis* genome of 1.34 Gb, which is slightly smaller than the prediction of 1.47 Gb based on Illumina data (Additional File 4: Supplementary Table  S1). The average GC content is 32.57%, and N50 value is 139.92 Mb. A total of 20 scaffolds were obtained, of which more than 99.9% of the sequences were loaded onto 9 scaffolds that reached the chromosome level (Fig. 2A), which was consistent with the previous karyotype analysis of *O. sinensis* [14]. The BUSCO [15, 16] complete analysis was 95.3% (arachnida_odb10, Additional File 4: Supplementary Table  S2).

In the annotation of repetitive sequences of the genome, we found that the proportion of repetitive regions was 55.08%, and the most recognizable element was DNA transposons, which accounted for 18.5% of the genome (Additional File 4: Supplementary Table  S3, Additional File 5: Supplementary Fig. S1), similar to other spider groups with genomic data [17–19]. In our assembly, 24,579 coding genes were annotated, and 24,563 genes have obtained effective functional annotation in at least one of the following databases: NCBI-Nr, Swiss-Prot, or EggNOG v5.0 [20]. The chromosome loading rate of coding genes was 99.09%. The BUSCO assessment of protein level was 94.8% (Additional File 4: Supplementary Table  S2).

### Divergence time estimation and synteny analysis

Genomes of *O. sinensis, U. diversus* [22], and the model species—the house spider (*Parasteatoda tepidariorum*) [21]—and 7 other representative species (Additional File 4: Supplementary Table  S4) were selected for orthologous gene identification (see Methods). A total of 1,560 single-copy gene families shared by all species were identified and used to construct the phylogenetic tree (Fig. 2B). All nodes have 100% ultrafast bootstrap support, and the topolog and the estimated divergence time of each node are similar to those of previous studies [23–25].

Among the species involved in the above analysis, genomes with chromosome-level assemblies were selected for synteny analysis with *O. sinensis* (Additional File 4: Supplementary Table  S4). There is a trend that the closer the relationship, the stronger the collinearity (Fig. 2B). Because *Deinopis* sp. lack comprehensive genomic data, this species was not included in the synteny analysis (red branch, Fig. 2B).

### Genes under positive selection and energy metabolism in muscle

**Figure 3:**
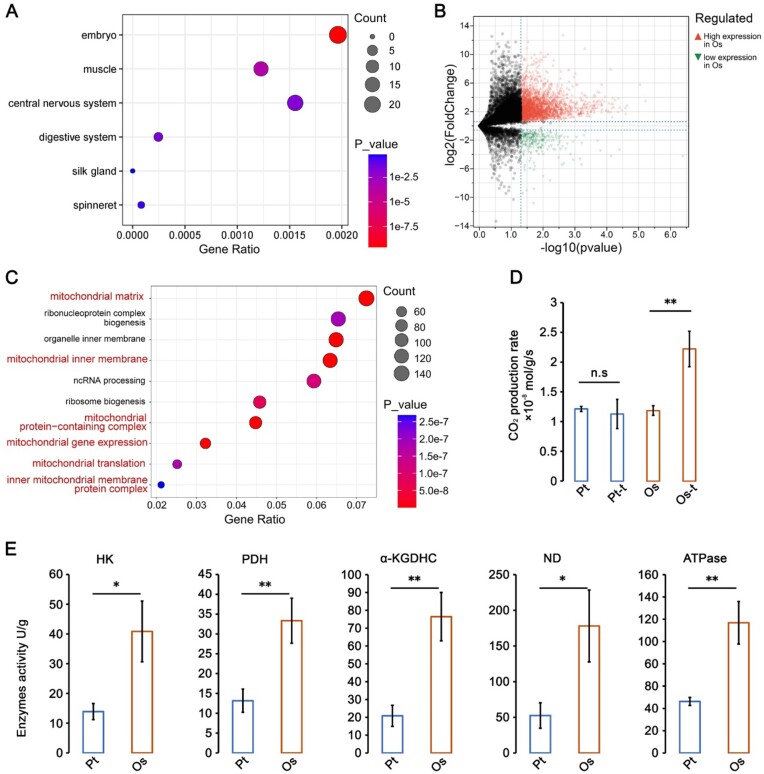
Positive selection genes and energy metabolism. (A) Tissue enrichment of genes under positive selection. (B) Differential expression analysis of homologous genes in the legs of *Parasteatoda tepidariorum* and *Octonoba sinensis* (only reciprocal best hit genes were considered, fold-change > 1.5, P < 0.05). Genes exhibiting higher expression levels in *O. sinensis* legs are designated with red upper triangles, whereas those with higher expression in *P. tepidariorum* legs are designated with green lower triangles. (C) GO terms enrichment analysis. Compared to the legs of *P. tepidariorum*, genes with higher expression (fold-change > 1.5, *P* < 0.05) in the legs of *O. sinensis* were analyzed. GO terms related to the mitochondrion are highlighted in red. Metabolic rate measurement (D) and enzyme activity (E) of hexokinase (HK), pyruvate dehydrogenase (PDH), alpha-ketoglutarate dehydrogenase complex (α-KGDHC), NADH dehydrogenase (ND), and ATP synthase (ATPase). Os: *O. sinensis*; Pt: *P. tepidariorum*; -t: under fatigue treatment. Significant differences are denoted by *P < 0.05 and **P < 0.01; n.s., not significant.

In the selection pressure analysis, at the node of the Uloboridae (asterisk, Fig. 2B), 401 genes are under positive selection (Additional File 4: Supplementary Table  S5). Although these genes did not achieve effective Gene Ontology (GO) enrichment (*P*-adjust < 0.05), we found that there is tissue preference in the expression of some positive selection genes (PSGs), and in *O. sinensis*, these genes have the highest enrichment in embryos and muscles (Fig. 3A). It is worth noting that these PSGs in muscle tissue include *myosin* (gene ID: g27351, *P* = 2.59e-04), an important molecular motor [26, 27], and *twitchin* (gene ID: g8872, *P* = 7.55e-05), a key regulator of muscle movement [28].

To further explore the evolution of the motor function of Uloboridae, we compared the transcriptome data of the legs between *O. sinensis* and other species. We used the model species *P. tepidariorum*, which is also a web-building spider, as a control. Under consistent standardized conditions, the results showed that a large number of genes were differentially expressed (Fig. 3B, Additional File 4: Supplementary Table  S6). Compared to the legs of *P. tepidariorum*, genes with higher expression in the legs of *O. sinensis* were most enriched in the mitochondrial matrix; meanwhile, significant enrichment was also observed in other GO terms about mitochondria (Fig. 3C, Additional File 5: Supplementary Fig. S2). This result suggests that at least in the legs, *O. sinensis* requires greater energy consumption compared to typical web-building spiders. On this basis, we examined the activities of several key enzymes in the mitochondria involved in energy metabolism, including hexokinase, pyruvate dehydrogenase, α-ketoglutarate dehydrogenase, NADH dehydrogenase, and ATP synthase. Our results revealed that the activity levels of these 5 enzymes in the body of *O. sinensis* were higher compared to those in *P. tepidariorum* (Fig. 3E). Furthermore, while there was no significant difference in CO_2_ production rates between *O. sinensis* and *P. tepidariorum* in a resting state, *O. sinensis* exhibited significantly higher rates under fatigue treatment (Fig. 3D). These findings suggest that the evolution related to energy metabolism in the motor organs may be a key factor in the sustained output power of species in the Uloboridae family.

### Expanded and new emergent gene families

**Figure 4:**
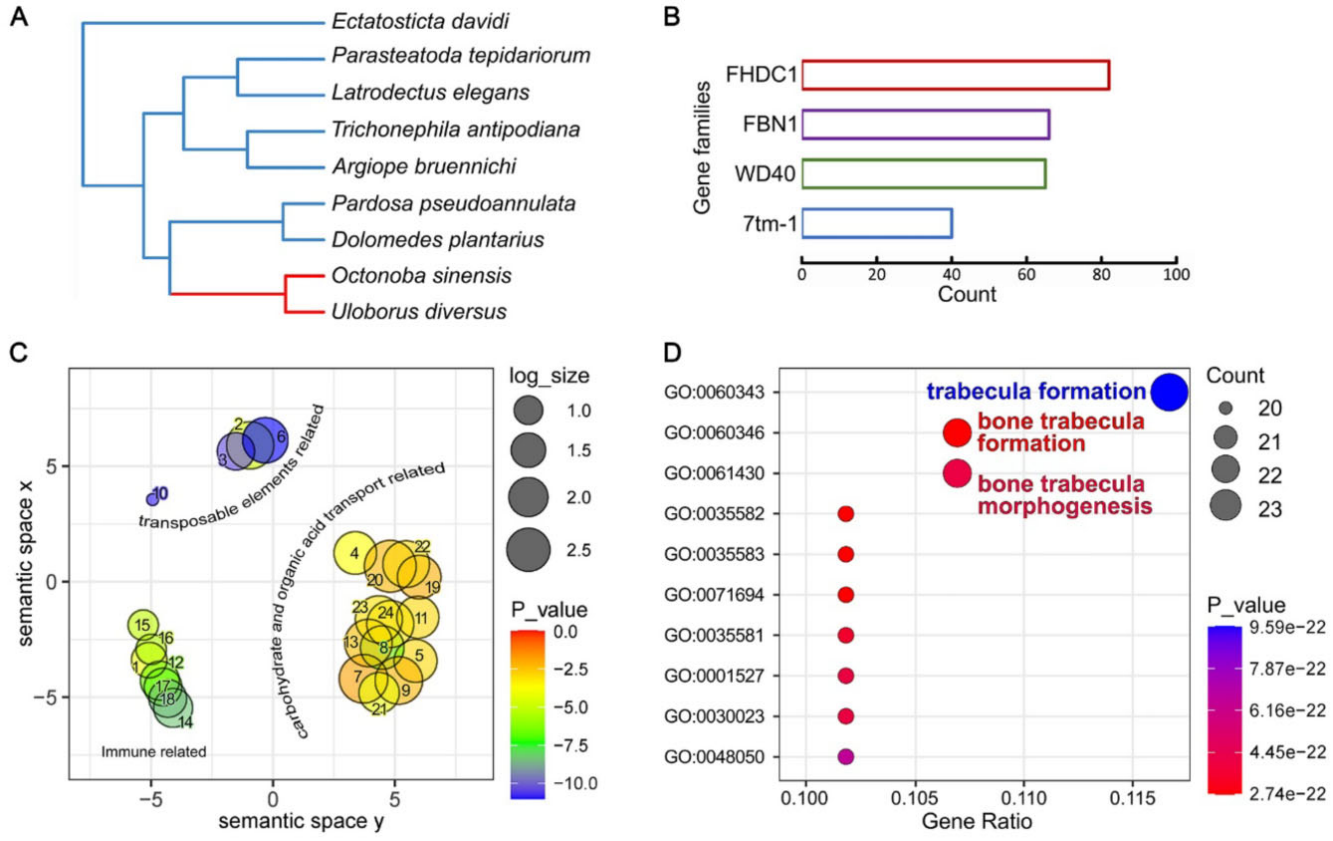
Expanded and new emergent gene families. (A) Species phylogeny for calculating the expansion and contraction of gene families. Uloboridae branches are in red. (B) The 4 superfamilies with the highest number of clearly annotated genes. (C) REVIGO plot of GO enrichment results for the other expanded gene families, excluding 4 superfamilies. The numbers in the figure refer to different GO terms: 1, response to molecules of bacterial origin; 2, DNA recombination; 3, DNA transposition; 4, receptor-mediated endocytosis; 5, carbohydrate transport; 6, DNA integration; 7, organic anion transport; 8, sialic acid transport; 9, organic acid transport; 10, transposition; 11, carbohydrate transmembrane transport; 12, response to type II interferon; 13, carboxylic acid transport; 14, response to interleukin 4; 15, cellular response to biotic stimulus; 16, cellular response to molecule of bacterial origin; 17, cellular response to type II interferon; 18, cellular response to interleukin 4; 19, monoatomic anion transmembrane transport; 20, inorganic cation transmembrane transport; 21, carbohydrate derivative transport; 22, proton transmembrane transport; 23, organic acid transmembrane transport; 24, carboxylic acid transmembrane transport. (D) GO enrichment of new emergent gene families in Uloboridae.

To compare the genomic differences between Uloboridae and other spiders, we used CAFE v4.2 [29] to analyze the gene family expansions and contractions. Results indicate that 123 gene families have undergone significant expansion at the ancestral node of the Uloboridae (Fig. 4A). In *O. sinensis*, we found 4 of these families to have a high number of annotated genes that corresponded to FH2 domain containing 1 (FHDC1), FBN1, WD40 repeat proteins (WD40), and 7-(pass)-transmembrane domain receptors 1 (7tm_1) (Fig. 4B, Additional File 4: Supplementary Table  S7). Interestingly, studies have shown that FHDC1 proteins not only play a crucial role in the development of the tracheal system in fruit flies [30] but also have significant implications in muscle movement [31, 32]. In addition, the preproprotein of FBN1 is proteolytically processed to generate 2 proteins, including the extracellular matrix component fibrillin 1 and the protein hormone asprosin. Fibrillin 1 is an extracellular matrix glycoprotein that serves as a structural component of calcium-binding microfibrils. These microfibrils provide force-bearing structural support in elastic and nonelastic connective tissue throughout the body. Asprosin has been shown to regulate glucose homeostasis [33]. Apart from the 4 superfamilies mentioned above, we have deployed GO enrichment analysis of the other expanded gene families. REVIGO [34] results show that these annotated genes were mainly enriched in the transport of carbohydrates and organic acids, the immune system, and the functions related to transposable elements (Fig. 4C).

Based on the results of orthologous gene identification, we screened for gene families that are exclusively shared in Uloboridae and not found in any other spider species (species in Fig. 2B), designating them as new emergent gene families. In total, 269 such gene families have been identified in both *O. sinensis* and *U. diversus*, with a total of 658 members in the *O. sinensis* genome (Additional File 4: Supplementary Table  S8). GO enrichment results showed that these genes are more enriched in GO terms that are related to bone trabecular development (Fig. 4D).

Generally speaking, spiders lack endurance, necessitating the prompt subduing of their prey within a brief timeframe during hunting [13]. However, uloborids can exercise intensely for nearly an hour [9]. Our results indicate that genes related to tracheal development, skeletal development, tissue force-bearing structures, and energy metabolism have significantly expanded or emerged in the genome of Uloboridae. We believe that the evolution of these aspects is highly likely to be related to their increased endurance.

### Absent regions and genes under relaxed purifying selection

**Figure 5:**
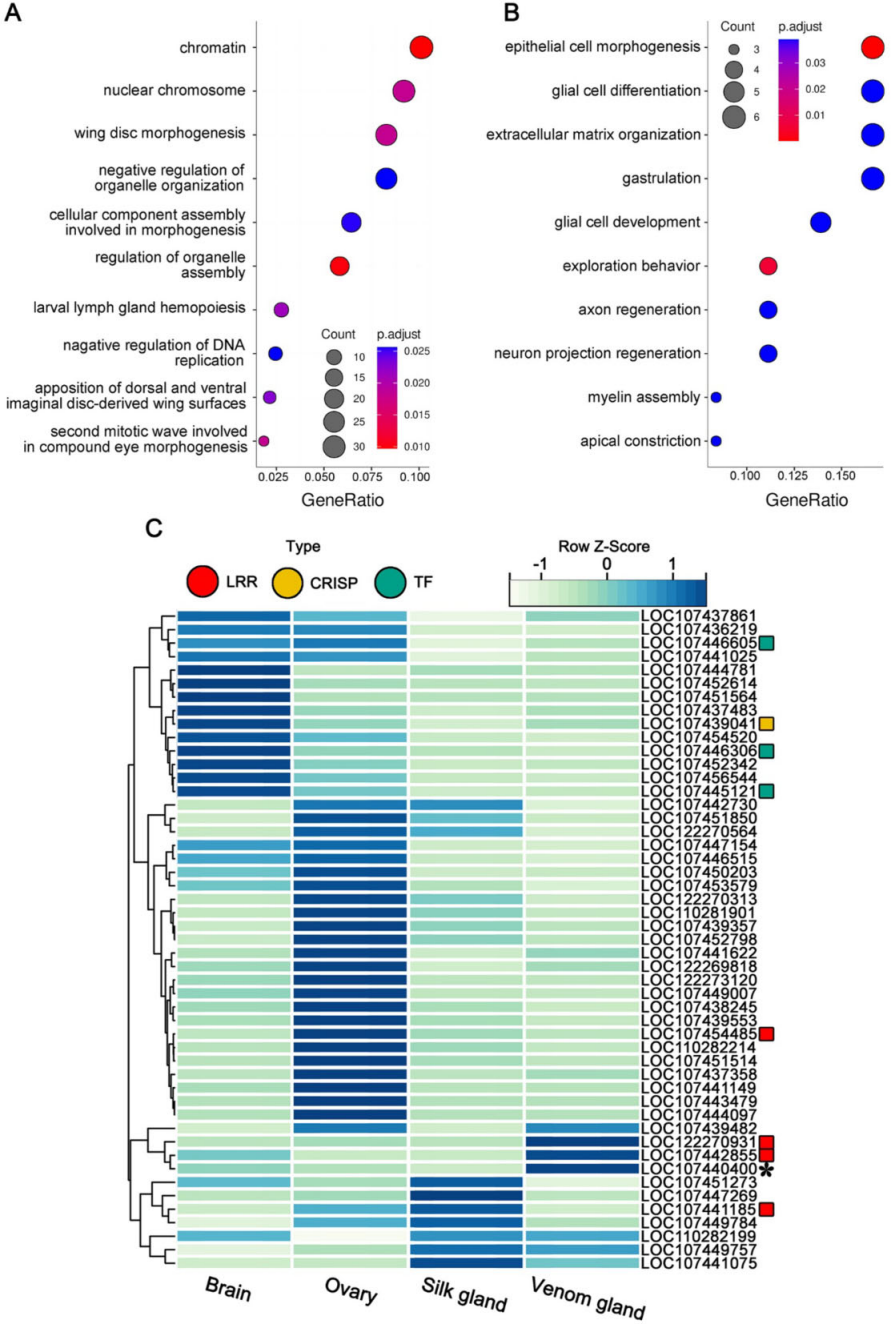
Absent regions and regions under relaxed selection. (A) GO enrichment of genes under relaxed selection in *Octonoba sinensis*. (B) GO enrichment of genes related to missing HCEs in *O. sinensis*. (C) The expression patterns of the genes specifically absent in Uloboridae were examined in the model species *Parasteatoda tepidariorum*. The heatmap is plotted based on the *z*-score transformed from transcripts per million (TPM) values. Different-colored markers are used to distinguish between distinct gene types, while an asterisk identifies the gene that is notably absent from the venom gland–specific expression module of *P. tepidariorum*.

Due to the absence of venom glands, the genes or functional regions specifically involved in the venom gland system in Uloboridae may be subjected to relaxed purifying selection or gradually lost from the genome. To obtain this information, highly conserved elements (HCEs) were searched spanning 9 spider genomes (Fig. 4A), and the sites with Uloboridae-specific deletions among them were identified (Additional File 5: Supplementary Fig. S3, Additional File 4: Supplementary Tables S9–S10). In addition, absent genes and genes under selective relaxation in the *O. sinensis* and *U. diversus* genomes were analyzed against the background of species with venom glands (Fig. 2B, Additional File 5: Supplementary Fig. S3, Additional File 4: Supplementary Tables S11–S12). We conducted functional enrichment on the above results and found that the biggest difference between Uloboridae and background species comes from the development, especially the neurodevelopment, related to gene family (Fig. 5A, B).

In the homologs missing from Uloboridae, we found that 5 genes belong to 2 toxin-related gene families (LRR and CRISP), as well as 3 transcription factors. It is worth noting that the expression patterns of the homologs of 2 LRR genes (LOC122270931, LOC107442855) in *P. tepidariorum* indicate their highest expression in venom glands, although neither of them has been annotated as homologs to known toxin genes (Fig. 5C). Three transcription factors belong to important components that activate transcription: “protein c-ets-2,” the “cooled coil and C2 domain containing protein (CC2D)” that regulates neurotransmitter expression, and the component of the STAGA complex: “ataxin-7.”

In addition, we referred to the venom gland–specific expression module of *P. tepidariorum* obtained in a previous study (*n* = 1,088, Additional File 4: Supplementary Table  S13) [21] and found that only 1 gene (LOC107440400), which is specifically lost in Uloboridae, intersects with this module. This gene is a SOBP (Sine Oculis-Binding Protein Homolog), and the protein encoded by this gene is involved in the development of the cochlea, and genetic defects are also related to intellectual disability (Fig. 5C).

### Deficiency of toxin genes in *O. sinensis*

**Figure 6:**
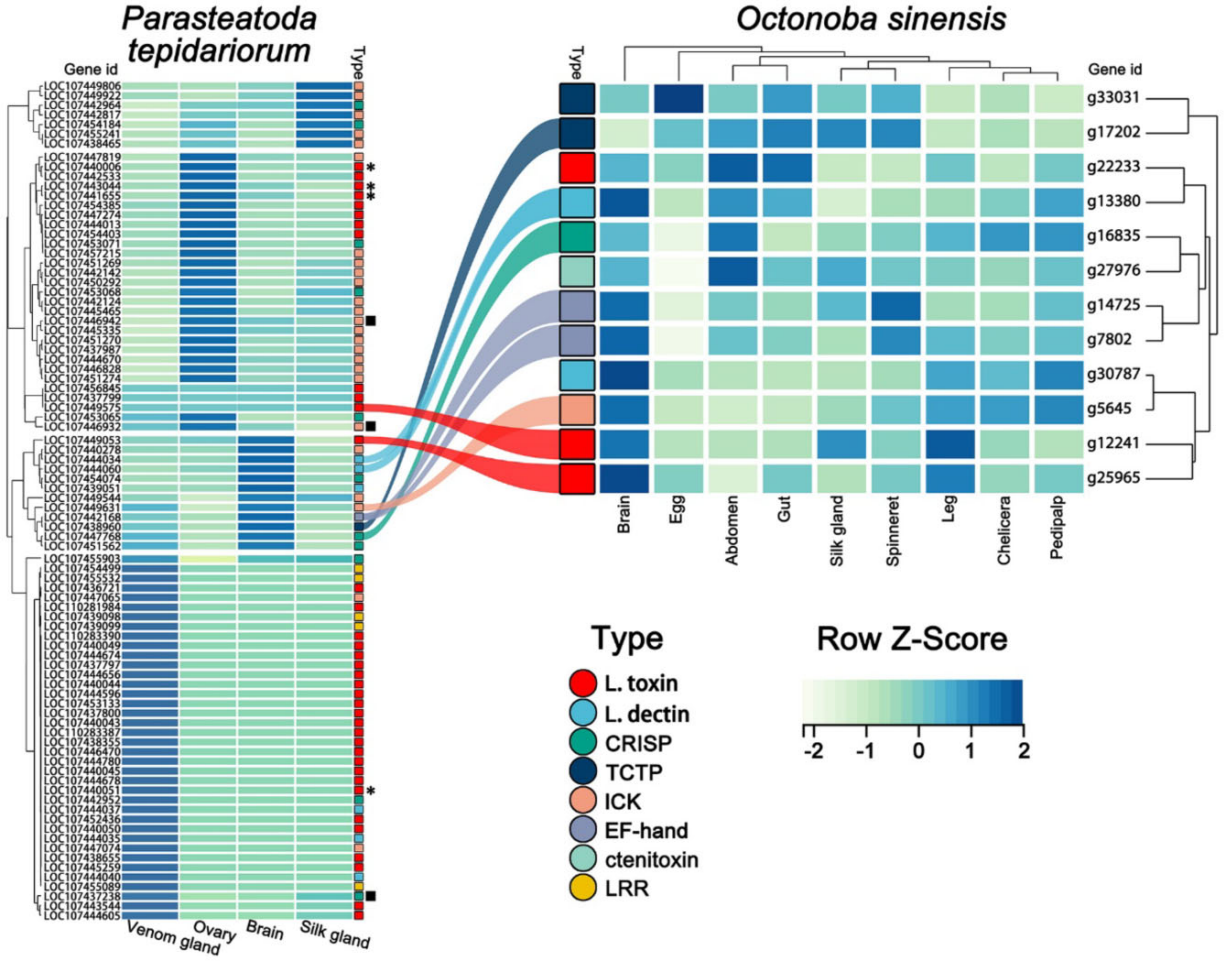
The expression patterns of toxin homolog coding genes in *Octonoba sinensis* and *Parasteatoda tepidariorum*. The heatmap is plotted based on the *z*-score transformed from TPM values. The ribbon connects the homolog genes. The black asterisks indicate the best match of possible *O. sinenesis* pseudogenes in *P. tepidariorum*. The black squares indicate homologs matching the *O. sinenesis* existence position in the collinearity (see Fig. 7B: red ribbons).

To search for toxin genes in *O. sinensis*, we integrated the results of previous studies and established a comprehensive toxin protein database (Additional File 6) and screened toxin gene homologs with the same threshold in different species. In the *O. sinensis* and *U. diversus* genomes, we identified 12 and 11 homologs, respectively, that have similar structures to members of 6 major toxin or venom component gene families (latrotoxin, latrodectin, CRISP, ICK, TCTP, EF-hand, and ctenitoxin). Our findings indicate that although the Uloboridae family has a relatively low number of toxin-related homologs, their count still exceeds that of some venomous spiders, such as *Ectatosticta davidi* [35–38] (Additional File 4: Supplementary Table  S14). Nevertheless, we discovered that none of these *O. sinensis* genes’ orthologs in *P. tepidariorum* exhibit high expression levels in venom glands (Fig. 6). Given that *O. sinensis* had 3 latrotoxin homologs out of 12 toxin homologs (highest category, Additional File 4: Supplementary Table S14) and latrotoxins are not known outside of Theridiidae, a phylogenetic analysis of this gene family has been conducted. In this analysis, we found that the 3 homologs in *O. sinensis* are not clustered on the same branch as the reported latrotoxin (Additional File 5: Supplementary Fig. S4) [39]. There is a hypothesis regarding the evolution of venom components that ancestors of toxin proteins were originally proteins with normal physiological functions that were recruited in venom glands to play the role of venom components [40], and our results also support this viewpoint.

To further explore the evolutionary processes of *O. sinensis* toxin genes in the absence of venom glands, we conducted a pseudogene search on noncoding regions of the *O. sinensis* genome, but traditional search methods did not identify pseudogenes (blastn, E-value 1e-5, matching length 50 bp) [41]. However, we did find more traces of toxin gene homologs in the blastx search through the protein sequence in the toxin genes database. These results include 48 different genomic regions, but only 1 of which had an effective hit with the relatively reliable toxin gene of *P. tepidariorum* (LOC107440051) (Additional File 4: Supplementary Table  S15, Fig. 6).

**Figure 7:**
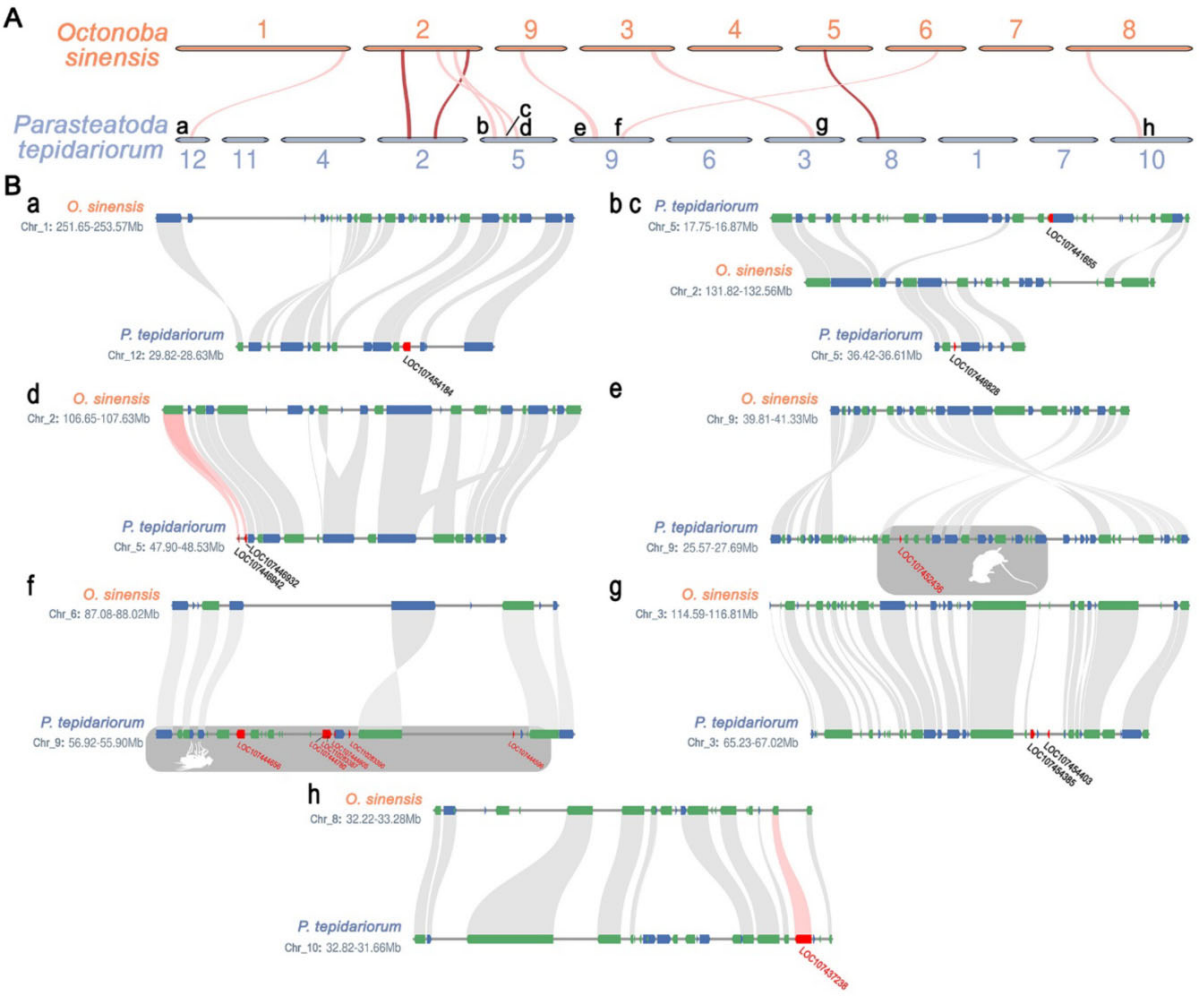
Collinearity containing toxin homologs between *Octonoba sinensis* and *Parasteatoda tepidariorum*. (A) Collinearity of all toxin homologs. If the toxin homologs were lost in *O. sinensis*, the collinearity fragments are represented by a pink ribbon and otherwise by a dark red band. (B) Gene correspondence in each collinearity fragment where toxin gene deletion occurs in (A). Genes located on the positive strand of the genome are represented in blue, while those located on the negative strand are represented in green. Each lowercase letter corresponds to the collinearity fragments represented by the same letter in (A); the toxin gene names in *P. tepidariorum* are listed, and the red gene names highlight the toxin genes that are highly expressed in the venom glands. The toxin genes in the gray background belong to the latrotoxin gene family, and different silhouettes indicate the type of toxin and the target group of toxicity. Mouse = vertebrates (alpha-latrotoxin), fly = insects (delta-latrotoxin); the red ribbons indicate that the collinearity of the linked genes is extremely weak, below the threshold (see Methods).

By searching for toxin gene homologs in collinearity fragments of *O. sinensis* and *P. tepidariorum*, a particular class of genes was found in *O. sinensis*. These genes are located in the same place as the *P. tepidariorum* toxin gene homologs in the collinearity segment, but they can no longer be identified as toxin genes (below the minimum recognition threshold; see Methods) (Fig. 7B, red ribbon). There are 3 pairs of such genes, including 1 pair of CRISP genes (g31478∼LOC107437238) and 2 pairs of ICK genes (g6736∼LOC107446942 and g6736∼LOC107446932). Their expression patterns in *P. tepidariorum* indicate that only the CRISP gene is a reliable toxin gene (Fig. 6, black squares). In *O. sinensis*, this CRISP gene (g31478) cannot be unambiguously classified as a homolog of toxin genes due to changes in protein structure (Additional File 4: Supplementary Table  S16). However, compared with other toxin genes, for which it is difficult to find pseudogenes, the ortholog of this CRISP gene in *O. sinensis* has complete gene structures and can be expressed in multiple tissues of a venomless spider (Additional File 4: Supplementary Table  S17). These all indicate that this CRISP gene (g31478) must play the role of a nontoxic gene. We believe that this observation suggests a potential functional shift between toxic and nontoxic genes in spiders.

## Discussion

During evolution, the loss of important organs is accompanied by a series of adaptive evolution, such as the enhancement of nonvisual senses of some eyeless organisms [42–46]. In Uloboridae, due to the absence of venom glands, prey-wrapping as the sole attack strategy is excessive. Multiomics analysis revealed the absence or possible functional shift of toxin genes in *O. sinensis*. At the same time, compared to venomous spiders, a series of genomic changes related to muscle, aerobic respiration, and metabolism of energy substances were observed in the genomes. These findings imply an evolutionary trade-off between the loss of venom glands and the enhancement of physical attack strategies.

Both gene family evolution and selection pressure shaped the genomic change related to the specialized physical attack of Uloboridae. For example, *myosin* and *twitchin* were under positive selection. Myosin provides the driving force for muscle contraction as a molecular motor [26, 27], while twitchin is located at the junction of muscle fibers and regulates the speed and force of muscle contraction [28]. The positive selection of these 2 genes suggests that they may play a role in the strengthening of muscle energy efficiency and contraction strength. Additionally, the gene family expansions of key proteins involved in muscle fiber connection, FHDC1 [31, 32], and the structural component of connective tissue, FBN1 [33], likely provided genetic resources for the optimization of muscle and connective tissue.

Aerobic respiration is the process that directly supplies energy to muscles. We found a higher expression of mitochondria-related genes in the legs of *O. sinensis* compared to *P. tepidariorum* (a venomous spider) (Fig. 3C). In addition, physiological and biochemical measurements also indicate an advantage of uloborids in respiratory efficiency and related enzyme activity (Fig. 3D, E). Moreover, the expansion of gene families involved in tracheal development [30], coupled with previous records that uloborids possess a more complex tracheal system compared to other web-building spiders [10, 11], all indicates that uloborids have the ability to provide sufficient oxygen supply for enhanced aerobic respiration.

The supply of energy substances is also crucial for sustained physical output. We found that the gene families involved in the transport of carbohydrates and organic acids have undergone expansion (Fig. 4C), which may help uloborids’ enhanced utilization and metabolism of substrates (or intermediate products) in aerobic respiration. Additionally, asprosin, encoded by an expanded gene family [33], acts as hormone-regulating glucose homeostasis, potentially mobilizing energy reserves to further provide energy for long-term physical activity.

Simultaneously, through analyzing expression patterns and synteny relationships, we discovered that toxin genes in venomous spiders are expressed in other tissues of uloborids and may perform nontoxic functions. This observation suggests a potential functional shift between toxic and nontoxic genes in spiders. In addition to toxin genes, the absence of certain genes exclusively in uloborids raises concerns. These include protein c-ets-2, CC2D, and ataxin-7, which are transcription factors involved in various metabolic and developmental processes. Additionally, notable missing genes include the SOBP gene and 2 LRR genes. The former is involved in nervous and organ development, while the latter has a similar structural domain to LRR toxin genes, all of which are highly expressed in venom glands of model species.

For predators, there exists a pervasive evolutionary trade-off between chemical and physical attack strategies. Previous research has frequently favored the exploration of chemical strategies, particularly venom. However, against the backdrop of nearly all spiders being toxic predators, our study delves into the genetic basis underlying the alternative choice in this trade-off. Unsurprisingly, reliable toxin gene was not identified in Uloboridae, but these adaptive evolutions ranging from muscle to aerobic respiration and then to the supply of energy substances provide strong support for the exceptional physical endurance demands of this group and compensate for their decreased adaptability due to the absence of venom glands. Furthermore, some development-related gene and element deletions were observed in uloborids. Although the association between these deletions and the absence of spider venom glands remains unclear, they still hold potential for exploring the evolutionary mechanisms underlying this phenomenon.

## Methods

### Sample collection and DNA extraction

Live *O. sinensis* were field-captured from Olympic Park, Chaoyang District, Beijing, China. To minimize the contamination of impurities in the digestive tract as much as possible, all samples were starvation-reared for more than 1 week at room temperature. Genome DNA for both short- and long-read sequencing were isolated from the cephalothoraxes of adult female spiders using the Blood & Cell Culture DNA Kit (QIAGEN).

### Observation of predation behavior and examination of fangs

We recorded a series of videos to observe the predation behavior, using a Logitech StreamCam 960–001,282. Wrapping duration was timed. If the spider stopped for more than 3 seconds, the timing also stopped but would continue if it began again. If the spider began to soak (*O. sinensis* will emit liquid onto their prey before eating) or left the prey, the video was terminated.

The dissected spider fangs were pasted onto a copper substrate at different angles, dried with a CO_2_ critical point drying method, coated with gold, and then observed with the SEM (model: FEI Quanta 450).

### Genome sequencing and genome size estimation

Short-insert libraries of *O. sinensis* were sequenced with the DNBSEQ-G400 (RRID:SCR_017980) using paired-end (PE) reads of 150 bp (BGI). To remove low-quality reads and adapters, raw reads were trimmed by Trimmomatic (RRID:SCR_011848) v0.39 [47]. A total of 259.82 Gb of clean data were obtained for *O. sinensis* for survey analysis and assembly correction.

For long-read sequencing, “SMRTbell” (double-stranded DNA template capped by hairpin loops at both ends) libraries were constructed according to the standard protocol of PacBio using 15 kb of preparation solution (PacBio). The high-fidelity (HiFi) libraries were sequenced on 3 SMRT cells on the PacBio Sequel II system (RRID:SCR_017990) in Circular Consensus Sequencing mode at Novogene Technology and generated 67.18 Gb HiFi data (3,967,026 reads) total [48].

To further improve the continuity of the assembled genomes, chromosome conformation capture (Hi-C) experiments were conducted [49]. Hi-C libraries were prepared following a published protocol with minor modifications [50]. For cross-linking, samples were fixed with 1% formaldehyde. The cross-linked DNA was digested with MboI restriction endonuclease and marked with biotin-14-dCTP to remove nonligated DNA fragments. The ligated DNA was extracted with a QIAamp DNA Mini Kit (Qiagen). The purified DNA was then sheared to ∼350-bp fragments and followed by a standard Illumina library preparation protocol [51]. Hi-C sequencing of *O. sinensis* was conducted on the DNBSEQ-G400 platform with PE 150 bp (BGI). We then filtered the raw reads using Juicer v1.6.2 [52] to remove low-quality reads and adapters, yielding 201.99 Gb of clean data.

Before *de novo* assembly, we estimated the genome size of this species. Using the Illumina data, Jellyfish (RRID:SCR_005491) (v2.1.3) [53] was employed to calculate the frequency of each *k*-mer (*k* = 17–31). Then, the genome size of *O. sinensis* was estimated using a method based on *k*-mer distribution.

### *De novo* genome assembly and quality assessment

PacBio reads were first assembled using 2 *de novo* assemblers: hifiasm (RRID:SCR_021069) v0.15.2 [54] and wtdbg2 (RRID:SCR_017225) v2.5 [55]. The best assembly was selected according to the optimal continuity and completeness. The final version of contigs was polished with Racon (RRID:SCR_017642) v1.4.17 for 3 rounds based on long reads and NextPolish (RRID:SCR_025232) v1.4.0 [56] using short reads. Contig-level genome completeness assessment was performed using BUSCO (RRID:SCR_015008) v5.2.2 [15, 16]. Genome consistency assessment was evaluated by mapping the short reads to the genome with Minimap2 (RRID:SCR_018550) v2.24-r1122 [57] and SAMtools/BCFtools (RRID:SCR_005227) v1.10 [58].

We used Hi-C–based proximity-guided assembly to generate chromosomal-level genome assemblies for *O. sinensis*. Hi-C library sequencing data were mapped to the contig-level genome using Juicer (RRID:SCR_017226). The 3D-DNA (RRID:SCR_017227) v180922 [59] pipeline was executed to construct the chromosomes and correct the errors. We further performed correction with Juicebox Assembly Tools (RRID:SCR_021172) v1.11.08 [60]. The completeness of the chromosomal level assembly was assessed by BUSCO.

### RNA extraction, sequencing, and expression analysis

Different tissue samples (spinneret, leg, brain, gut, silk gland, pedipalp, chelicera, and abdomen) from adult female *O. sinensis* and eggs (development stage undetermined) were dissected for total RNA extraction using an RNAsimple Total RNA kit (TIANGEN). The RNA sequencing libraries were constructed with insert sizes of ∼150 bp and sequenced on the NovaSeq 6000 platform (RRID:SCR_016387). We produced about 6 Gb of data per sample. Low-quality reads, reads with adapters, and unknown bases were filtered using Trimmomatic (RRID:SCR_011848).

In addition, legs from adult female *P. tepidariorum* were subjected to transcriptome analysis according to the above process (see Data Availability), and transcriptome data from 4 tissue transcriptomes (PRJNA934108, including brain, ovary, silk gland, and venom gland) were downloaded from the Sequence Read Archive (SRA) database.

Clean reads were aligned to the genome using Hisat2 (RRID:SCR_015530) [61], followed by the quantification of all samples with HTSeq (RRID:SCR_005514) [62] to determine the count value. Subsequently, TPMs were derived through automated scripts.

In the differential expression analysis across 2 species, only reciprocal best hit (RBH) genes were extracted for quantification. To facilitate comparison, ortholog (RBH) gene IDs of *O. sinensis* were replaced by *P. tepidariorum* gene IDs for comparison and figure illustration. Finally, differential expression analysis was conducted by R package LIMMA (RRID:SCR_010943) [63].

### Genome annotation

The RepeatModeler (RRID:SCR_015027) v2.0.2 [64] and RepeatMasker (RRID:SCR_012954) v4.1.2-p1 [65] pipelines were used to annotate repetitive sequences in the genome.

Gene annotation was based on the braker (RRID:SCR_018964) v2.1.6 [66] pipeline, which combines the whole protein sequences of the 9 other species in this study (Additional File 4: Supplementary Table  S4) and more than 320 Gb of multitissue transcriptome data for comprehensive annotation.

Gene function annotation is based on NCBI-Nr, Swiss-Prot (RRID:SCR_021164), and EggNOG (RRID:SCR_002456) v5.0 databases. The transfer RNA was predicted using the program tRNAscan-SE (RRID:SCR_008637) v2.09 [67]. Other noncoding RNAs were annotated with the Rfam (RRID:SCR_007891) v14.8 [68] database through infernal (RRID:SCR_011809) v1.1.4 [69].

Among the other species involved in this study, *P. pseudoannulata* [70] and *D. plantarius* only have assembled sequence data currently. The genome of *L. elegans* has high assembly quality [37], but the protein BUSCO score is only 63.7%; *U. diversus* only has 15,750 annotated protein-coding genes, significantly less than other spider species. To obtain more reliable results for downstream analysis, we annotated the genomes of the aforementioned species based on transcriptome data. For *P. pseudoannulata, D. plantarius*, and *L. elegans*, we used our annotations, and for *U. diversus*, we used our annotation to supplement the original one. All annotation strategies are based on transcriptome data from the SRA (Additional File 4: Supplementary Table  S18) database and carried out through the TransDecoder (RRID:SCR_017647) pipelines. We also added *Deinopis* sp. of Deinopidae and assembled all of its proteins sequence using transcriptome data (DRR297048), and quality control was done using fastp (RRID:SCR_016962) version 0.21.0 [71]. *De novo* assemblies were done using Trinity (RRID:SCR_013048) v2.11.0 [72] under default settings. Open Reading Frame (ORF) prediction was done using TransDecoder. Redundancy reduction was done with CD-HIT (RRID:SCR_007105) version 4.8.1 (-c 0.98 -n 10) [73]. All the newly provided annotations mentioned above have a protein BUSCO score of over 90% (Additional File 4: Supplementary Table  S2).

### Orthologous gene identification, phylogenetic and synteny analysis

We used OrthoFinder (RRID:SCR_017118) v2.5.4 [74] to analyze the annotation information of species in this study (Additional File 4: Supplementary Table  S5). In the pipeline, Mafft (RRID:SCR_011811) v7.453 [75] was used to perform multiple sequence alignment, blastp (RRID:SCR_001010) v2.9.0+ [76] was used to perform sequence searches, and the phylogenetic tree was constructed using IQ-TREE (RRID:SCR_017254) v2.2.0 [77]. The calibration points are from fossil specimens [23]. The analysis was run twice, once to calculate the divergence time of different species and the second to calculate the expansion and contraction of gene families. The latter did not include *Deinopis* sp., as the only transcriptome data cannot determine the number of gene copies (Fig. 4A).

The phylogenetic tree of latrotoxin homologs (Additional File 5: Supplementary Fig. S4) was reconstructed (1,000 bootstrap) using the neighbor-joining method, following alignment of the full-length protein sequences via Mafft v7.453.

Collinearity analysis was conducted between *O. sinensis* and other species with chromosome-level genomes (*T. antipodiana, A. bruennichi, L. elegans, U. diversus, P. pseudoannulata, D. plantarius*, and *E. davidi* [78]) using the MCscan (RRID:SCR_017650) pipeline (Python version) in the jcvi toolkit (RRID:SCR_021641) [79].

### Test for selection pressure and gene family expansions/contractions

One-to-one ortholog identification among 10 species (Fig. 2B) was performed using the RBH method by blastp v2.9.0+. *O. sinensis* was used as a reference species. Finally, 5,848 RBH clusters (Additional File 4: Supplementary Table  S19) were retained for analysis.

To scan for genes under positive selection in *O. sinensis*, RBH clusters were used for the branch site model analysis using CODEML in the PAML v4.9j toolkit [80]. Each gene family sets *O. sinensis* and *U. diversus* as foreground branches (Fig. 2B). “Model A” and “Model A-null” models were compared. “Model A” assumes that the selection pressure of foreground branches is greater than that of background branches, and “Model A-null” is an alternative hypothesis.

To statistically test which genes of Uloboridae are under relaxed purifying selection, we used RELAX in the HYPHY (RRID:SCR_016162) v2.5.2 [81] toolkit to infer the free relaxation parameter *k* at the node of *O. sinensis* and *U. diversus* branches for genes shared by all species in the phylogeny of Fig. 2B (Additional File 4: Supplementary Table  S4). The relaxation parameter *k* is an exponent for selection parameters between the foreground and the background branches. A *k* > 1 suggests selection is more intensified in the foreground branch vs. the background branch and vice versa.

For gene family expansions/contractions, mcmctree in the PAML (RRID:SCR_014932) v4.9j toolkit was used to estimate the divergence time of each node in the phylogenetic tree of OrthoFinder pipeline results (without *Deinopis* sp.) (Fig. 4A). Next, we used CAFE (RRID:SCR_005983) v4.2 [29] under the default parameters to analyze the gene family expansions and contractions of 9 spider species.

### Identification of HCEs

To identify the HCEs, we initially generated pairwise sequence alignments across all 9 spider genomes (Fig. 4A) with LASTZ (RRID:SCR_018556) v1.04.15 [82] and chainNet [83], using the *P. tepidariorum* genome as the reference. We then used MULTIZ v11.2 [84] to combine the pairwise alignments into multiple sequence alignments. Subsequently, we ran phyloFit in the PHAST package (RRID:SCR_003204) [85] with the topology from OrthoFinder to estimate the neutral (“nonconserved”) model based on 4-fold degenerate sites. With the nonconserved model as input, we ran phastCons [86] to estimate conserved models with its intrinsic function and predicted the HCEs.

The distribution of HCEs in exons, introns, 2,000 bp upstream and 2,000 bp downstream of genes, and intergenic regions was summarized with Annovar (RRID:SCR_012821) [87, 88] based on the genome annotation information of *O. sinensis* or *P. tepidariorum*.

### Homologs of toxin gene family identification and analysis

Based on ArachnoServer 3.0 [89], a specialized spider venom database, and integrating toxin protein sequences obtained from other toxin research of spiders [37, 38, 90], we have compiled a new reference dataset. This dataset was used to conduct a blastp search for candidate toxin genes (E-value less than 1e-10, matching length greater than 70% of the reference sequence, and hit area mismatch less than 30%). We also established hidden Markov models for different types of toxin proteins based on the database and further confirmed the results obtained from blastp using HMMER (RRID:SCR_005305) v 3.3 [91]. To search for hidden toxin-related pseudogenes in *O. sinensis*, we referred to the identification criteria of human genome pseudogenes [41] and used blastn v2.9.0+ and blastx v2.9.0+ to search for candidates.

### Physiological index measurement

Assays were performed as described previously [92]. CO_2_ production rate was used as a proxy for metabolic rate (MR). The assays were conducted in a closed-circuit system with a volume of 73.3 mL at a temperature of 25°C, a pressure of 100.6 kPa, and a flow rate of 110 mL/min. MR was calculated as the amount of CO_2_ produced per gram of body mass per second, using the equation MR = MCO_2_/T/body mass, where MCO_2_ represents the amount of oxygen substance (in mol). To put spiders into a state of fatigue, we stimulated the spider’s legs with a dissecting needle and kept it in a high-intensity state of exercise for 10 minutes.

Enzyme activity was measured using the corresponding reagent kit (Wuhan Mosak Biotechnology Co., Ltd. KT50129, KT50577, KT42310, KT41589, KT87867). Female spider individuals in a resting state were fixed in liquid nitrogen after weighing and stored at −80°C. Prior to testing, the samples were homogenized and diluted to 500 μL as the test solution following the supplier’s protocol.

## Supplementary Material

giae048_GIGA-D-23-00275_Original_Submission

giae048_GIGA-D-23-00275_R3

giae048_GIGA-D-23-00275_Revision_1

giae048_GIGA-D-23-00275_Revision_2

giae048_Response_to_Reviewer_Comments_Original_Submission

giae048_Response_to_Reviewer_Comments_Revision_1

giae048_Response_to_Reviewer_Comments_Revision_2

giae048_Reviewer_1_Report_Original_Submission Nadia Ayoub -- 11/10/2023 Reviewed

giae048_Reviewer_1_Report_Revision_1 Nadia Ayoub -- 2/18/2024 Reviewed

giae048_Reviewer_2_Report_Original_Submission Sandra Correa-Garhwal -- 11/13/2023 Reviewed

giae048_Reviewer_2_Report_Revision_1 Sandra Correa-Garhwal -- 3/7/2024 Reviewed

giae048_Reviewer_2_Report_Revision_2 Sandra Correa-Garhwal -- 4/29/2024 Reviewed

giae048_Supplemental_Files

## Data Availability

The sequencing data are deposited in NCBI BioProjects PRJNA1018860 and PRJNA1019401, as well as ScienceDB [93]. All additional supporting data are available in the *GigaScience* repository, GigaDB [94].

## Additional Files

**Additional Files 1–3**. Hunting video.

**Additional File 4:**

**Supplementary Table S1**. Genome survey prediction of *Octonoba sinensis*.

**Supplementary Table S2**. Genome assembly.

**Supplementary Table S3**. Repeat sequences of *Octonoba sinensis* genome.

**Supplementary Table S4**. Transcriptome assembly and genomes used in this study.

**Supplementary Table S5**. Genes under positive selection pressure on the node of the family Uloboridae.

**Supplementary Table S6**. Transcriptome differential expression analysis of the legs of *Parasteatoda tepidariorum* and *Octonoba sinensis*.

**Supplementary Table S7**. Genes list of significantly expanded gene families in *Octonoba sinensis*.

**Supplementary Table S8**. New emergent gene family members in the *Octonoba sinensis* genome.

**Supplementary Table S9**. All HCEs, using the *Parasteatoda tepidariorum* genome as a reference.

**Supplementary Table S10**. Missing HCEs in *Octonoba sinensis* and *Uloborus diversus*.

**Supplementary Table S11**. Specific missing orthologous groups in *Octonoba sinensis* and *Uloborus diversus*.

**Supplementary Table S12**. Genes under relaxed selection pressure in Uloboridae.

**Supplementary Table S13**. *Parasteatoda tepidariorum* venom gland-specific expression module.

**Supplementary Table S14**. Quantitative distribution of different toxin homologs across species.

**Supplementary Table S15**. “Pseudogene” blastx.

**Supplementary Table S16**. Protein domain search.

**Supplementary Table S17**. Expression levels (TPM) in different organizations of the genes connected by the red band to the *Parasteatoda tepidariorum* toxin gene in Fig. 7H.

**Supplementary Table S18**. The SRA data used for genome annotation.

**Supplementary Table S19**. Clusters of Reciprocal Best Hits (RBH) and RBH between *Octonoba sinensis* and *Parasteatoda tepidariorum*.

**Additional File 5:**

**Supplementary Fig. S1**. Recognizable elements of the *Octonoba sinensis* genome.

**Supplementary Fig. S2**. GO enrichment analysis of genes expressed at higher levels (fold-change > 4, *P* < 0.05) in *O. sinensis* legs relative to those in *P. tepidariorum* legs.

**Supplementary Fig. S3**. Distribution of all highly conserved elements (HCEs) in *P. tepidariorum* and missing HCEs and genes in *O. sinensis*.

**Supplementary Fig. S4**. Phylogenetic tree of homologs of neurotoxin genes (Latrotoxin).

**Additional File 6**. Dataset of toxin reference genes.

## Abbreviations

BUSCO: Benchmarking Universal Single-Copy Orthologs; GO: Gene Ontology; HCE: highly conserved element; HiFi: high-fidelity; MR: metabolic rate; ORF: open reading frame; PE: paired end; PSG: positive selection gene; RBH: reciprocal best hit; SEM: scanning electron microscope; SRA: Sequence Read Archive; TPM: transcripts per million.
