# Supplementary material for: A trade-off in evolution: the adaptive landscape of spiders without venom glands
Source: Gigascience. 2024 Aug 5;13:giae048. doi: 10.1093/gigascience/giae048 (PMC11299198; doi:10.1093/gigascience/giae048)
Supplement: giae048_GIGA-D-23-00275_Original_Submission [file giae048_giga-d-23-00275_original_submission.pdf]

GigaScience

A Trade-off in Evolution: The Adaptive Landscape of Spiders without Venom Glands

--Manuscript Draft--

**Manuscript Number:**	GIGA-D-23-00275
**Full Title:**	A Trade-off in Evolution: The Adaptive Landscape of Spiders without Venom Glands
**Article Type:**	Research
**Funding Information:**	Strategic Priority Research Program of Chinese Academy of Sciences (XDB31000000) — professor Shuqiang Li

Abstract:

Background

Venom glands play a key role in the predation and defense strategies of almost all spider groups. However, spiders of family Uloboridae have evolved an adaptive strategy without venom glands. They excessively wrap their prey directly with spider silk instead of paralyzing it first with toxins. This shift of survival strategy is very fascinating, but the genetic basis behind it is poorly understood.

Results

We conducted multi-omics analyses on Octonoba sinensis, a widely distributed species of the Uloboridae family. We identified numerous positive selection signals in pathways related to energy metabolism. Additionally, we observed a significant expansion of gene families associated with the trachea development and actin regulation, such as members of the Formin superfamily. The detection of relevant physiological indicators and previous anatomical observations suggest that these adaptive evolutions contribute to cover the decline in adaptability caused by the absence of venom glands. And, we found that the absence of regions and regions under relaxed selection in the spider genome are concentrated in the field of development, especially neuro-development. In addition, we also discovered that certain toxin genes have been retained in the genome of O. sinensis, indicating that these toxins may serve other functions, including oral toxicity and even non-toxic functions.

Conclusions

This study demonstrates the trade-off between different predation strategies in spiders and provides insights into the mechanism underlying this trade-off. Additionally, we have opened up a new perspective for further research on the evolution of spider venom systems.

**Corresponding Author:**	Shuqiang Li, Ph.D.<br>Institute of Zoology Chinese Academy of Sciences<br>Beijing, CHINA
**Corresponding Author Secondary Information:**	
**Corresponding Author's Institution:**	Institute of Zoology Chinese Academy of Sciences
**Corresponding Author's Secondary Institution:**	
**First Author:**	Yiming Zhang
**First Author Secondary Information:**	
**Order of Authors:**	Yiming Zhang
	Yunxiao Shen
	Bingyue Zhu
	Pengyu Jin

Powered by Editorial Manager® and ProduXion Manager® from Aries Systems Corporation

	Yejie Lin
	Yang Wang
	Shuqiang Li, Ph.D.
**Order of Authors Secondary Information:**	
**Additional Information:**	
**Question**	**Response**
Are you submitting this manuscript to a special series or article collection?	No
**Experimental design and statistics**<br><br>Full details of the experimental design and statistical methods used should be given in the Methods section, as detailed in our Minimum Standards Reporting Checklist. Information essential to interpreting the data presented should be made available in the figure legends.<br><br>Have you included all the information requested in your manuscript?	Yes
**Resources**<br><br>A description of all resources used, including antibodies, cell lines, animals and software tools, with enough information to allow them to be uniquely identified, should be included in the Methods section. Authors are strongly encouraged to cite Research Resource Identifiers (RRIDs) for antibodies, model organisms and tools, where possible.<br><br>Have you included the information requested as detailed in our Minimum Standards Reporting Checklist?	Yes
**Availability of data and materials**<br><br>All datasets and code on which the conclusions of the paper rely must be either included in your submission or deposited in publicly available repositories (where available and ethically	Yes

appropriate), referencing such data using a unique identifier in the references and in the "Availability of Data and Materials" section of your manuscript.<br><br>Have you have met the above requirement as detailed in our Minimum Standards Reporting Checklist?	

Manuscript Click here to access/download;Manuscript;giga.docx

A Trade-off in Evolution: The Adaptive Landscape of Spiders without Venom Glands

Yiming Zhang†[1,2,3], Yunxiao Shen†[1,3], Bingyue Zhu[1,3], Pengyu Jin[1], Yejie Lin[2], Yang Wang[1,3] and Shuqiang Li[1]*

[1]Key Laboratory of Zoological Systematics and Evolution, Institute of Zoology, Chinese Academy of Sciences, Beijing 100101, China

[2]Hebei Key Laboratory of Animal Diversity, College of Life Sciences, Langfang Normal University, Langfang 065000, China

[3]University of Chinese Academy of Sciences, Beijing 101408, China

† Yiming Zhang and Yunxiao Shen contributed equally to this work.

*Corresponding author: E-mail: lisq@ioz.ac.cn

Abstract

Background: Venom glands play a key role in the predation and defense strategies of almost all spider groups. However, spiders of family Uloboridae have evolved an adaptive strategy without venom glands. They excessively wrap their prey directly with spider silk instead of paralyzing it first with toxins. This shift of survival strategy is very fascinating, but the genetic basis behind it is poorly understood.

Results: We conducted multi-omics analyses on *Octonoba sinensis*, a widely distributed species of the Uloboridae family. We identified numerous positive selection signals in pathways related to energy metabolism. Additionally, we observed a significant expansion of gene families associated with the trachea development and actin regulation, such as members of the Formin superfamily. The detection of relevant physiological indicators and previous anatomical observations suggest that these adaptive evolutions contribute to cover the decline in adaptability caused by the absence of venom glands. And, we found that the absence of regions and regions under relaxed selection in the spider genome are concentrated in the field of development, especially neuro-development. In addition, we also discovered that certain toxin genes have been retained in the genome of *O. sinensis*, indicating that these toxins may serve other functions, including oral toxicity and even non-toxic functions.

Conclusions: This study demonstrates the trade-off between different predation strategies in spiders and provides insights into the mechanism underlying this trade-off. Additionally, we have opened up a new perspective for further research on the evolution of spider venom systems.

Keywords: Spider, Venom gland deficient, Adaptive evolution, Genome, *Octonoba sinensis*

Introduction

"Venomous" is a common way that people perceive spiders (Araneae). The toxic, painful and even fatal bite is always frightening. Indeed, almost all spiders are venomous. In the earliest divergent suborder Mesothelae, they already had fangs that could inject venom [1], and some highly toxic species make spiders even more notorious. However, as an important means of hunting and defense, the toxin system gives spiders an outstanding advantage in environmental suitability and has allowed them to spread throughout the world. Uloboridae is one of the most peculiar groups of spiders, and their most important feature is that all members of this family do not have venom glands [2]. The characteristic of venom gland deficiency in this family provides a satisfactory model for us to study the evolution of an important synapomorphy of spiders and the adaptation strategy changes brought about by the loss of important functional traits.

The whole organism is so tied together that when slight variations in one part occur, and are accumulated through natural selection, other parts become modified. This is a very important subject, most imperfectly understood [3]. Due to the absence of venom glands, the predation methods of these spiders are also relatively specialized. Observations of the predatory behavior of this group have found that, unlike other spider species that rely on instantaneous explosive force to subdue their prey, the long-time and high-intensity wrapping of prey is very common [4–6]. However, this strategy inevitably incurs a higher energy output burden. Previous anatomical records indicate that spiders in the family Uloboridae have well-developed trachea, and many branches of the trachea extend into the prosoma and appendages [7, 8]. Unfortunately, there is no relevant research that can link these anatomical characteristics to the specialized predatory strategies of this group.

Octonoba sinensis belongs to the family Uloboridae. Their body size is relatively larger than other members of the family [9], and this species is widely distributed in East Asia, Southeast Asia and North America [10]. Its habitat is close to human buildings, and the populations are large, so they can be easily collected in cities (Figure 1AB). These aforementioned characteristics make it a model species of the Uloboridae to study the biological characteristics of this family.

In this study, we generated a chromosome-level genome assembly of *O. sinensis*. By leveraging multi-omics datasets from various spider species as a background, we explored the molecular basis of the unique adaptive strategies in this group. We found that numerous genes related to energy metabolism in *O. sinensis* have undergone significant positive selection. Specifically, the activity of several key enzymes involved in aerobic respiration was notably enhanced. Additionally, the expansion of gene families associated with tracheal development and actin regulation in this species offers a plausible explanation for the improvement in respiratory efficiency and the well-developed tracheal system observed in its anatomy. Moreover, our investigation of toxin genes in *O. sinensis* provides a fresh perspective on the complex evolutionary history of spider venom.

Results

1. Observation of predation behavior and fangs

Through our observations, like other species in the family Uloboridae, *O. sinensis* only arrest their prey through extensive silk wrapping. The time usually exceeds 3 minutes (sometimes even 8–9 minutes), during which there may be several brief breaks (Additional file 1–3). This time is much higher than the previously recorded wrapping time of all species of family Araneoidea (9.7 ± 3.0 seconds for small prey, 26 ± 42 seconds for big prey) [11]. In addition, for the first time, we observe the fangs of *O. sinensis*, with a multi-angle under Scanning Electron Microscope (SEM). Generally speaking, if a species has venom glands, a channel opening for injecting venom should be found on the fangs [1, 12]. We did not observe this in *O. sinensis*, which once again proves that the hunting strategy of this group does not use venom injection (Figure 1C).

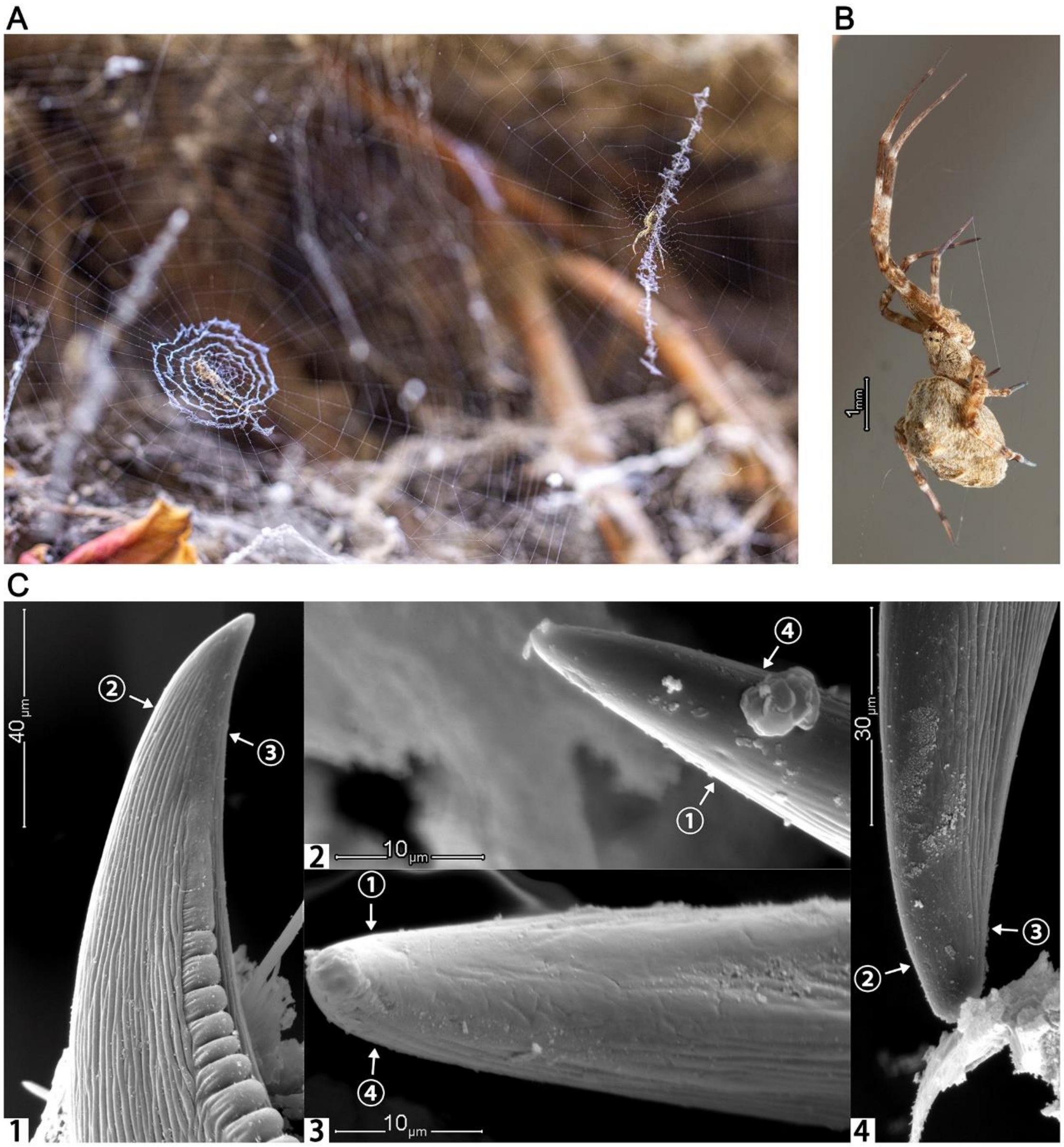

Fig. 1: Observation of *Octonoba sinensis*. (A) Two specimens of *O. sinensis* on their orb-webs in their natural environment. (B) Adult of *O. sinensis*. (C) SEM image of *O. sinensis* fangs. Four images were taken from four angles of the fang. The arrow represents the direction from which the images were taken and the number in the circle represents the image number.

2. Genome assembly and annotation

We assembled an *O. sinensis* genome of 1.34 Gb, which is slightly smaller than the prediction of 1.47

Gb based on Illumina data (Additional file 4: Table S1). Average GC content is 32.57%, N50 value is 139.92 Mb. A total of 20 scaffolds were obtained, of which more than 99.9% of the sequences were loaded onto 9 scaffolds that reached the chromosome level (Figure 2A), which was consistent with the previous karyotype analysis of *O. sinensis* [13]. The BUSCO genome completeness score is 95.3% [14, 15], as a percentage of 2934 genes in arachnida_odb10, and the Illumina reads mapping rate is 98.11%, (Additional file 4: Table S2).

In the annotation of repetitive sequences of the genome, we found that the proportion of repetitive regions was 55.08%, and the most recognizable element was DNA transposons, which accounted for 18.5% of the genome (Additional file 4: Table S3, Additional file 5: Figure S1). In spider species from which genomic data have been attained, most species are dominated by DNA transposons [16–18]. In our assembly, 24579 coding genes were annotated, of which 99.93% (24563 genes) have obtained effective functional annotation in at least one of NCBI-Nr (http://www.ncbi.nlm.nih.gov), Swiss-Prot (http://www.uniprot.org/) or EggNOG v5.0 databases (http://eggnog5.embl.de/) [19]. The chromosome loading rate of coding genes is 99.09%. The BUSCO assessment of protein level is 94.8% (Additional file 4: Table S4).

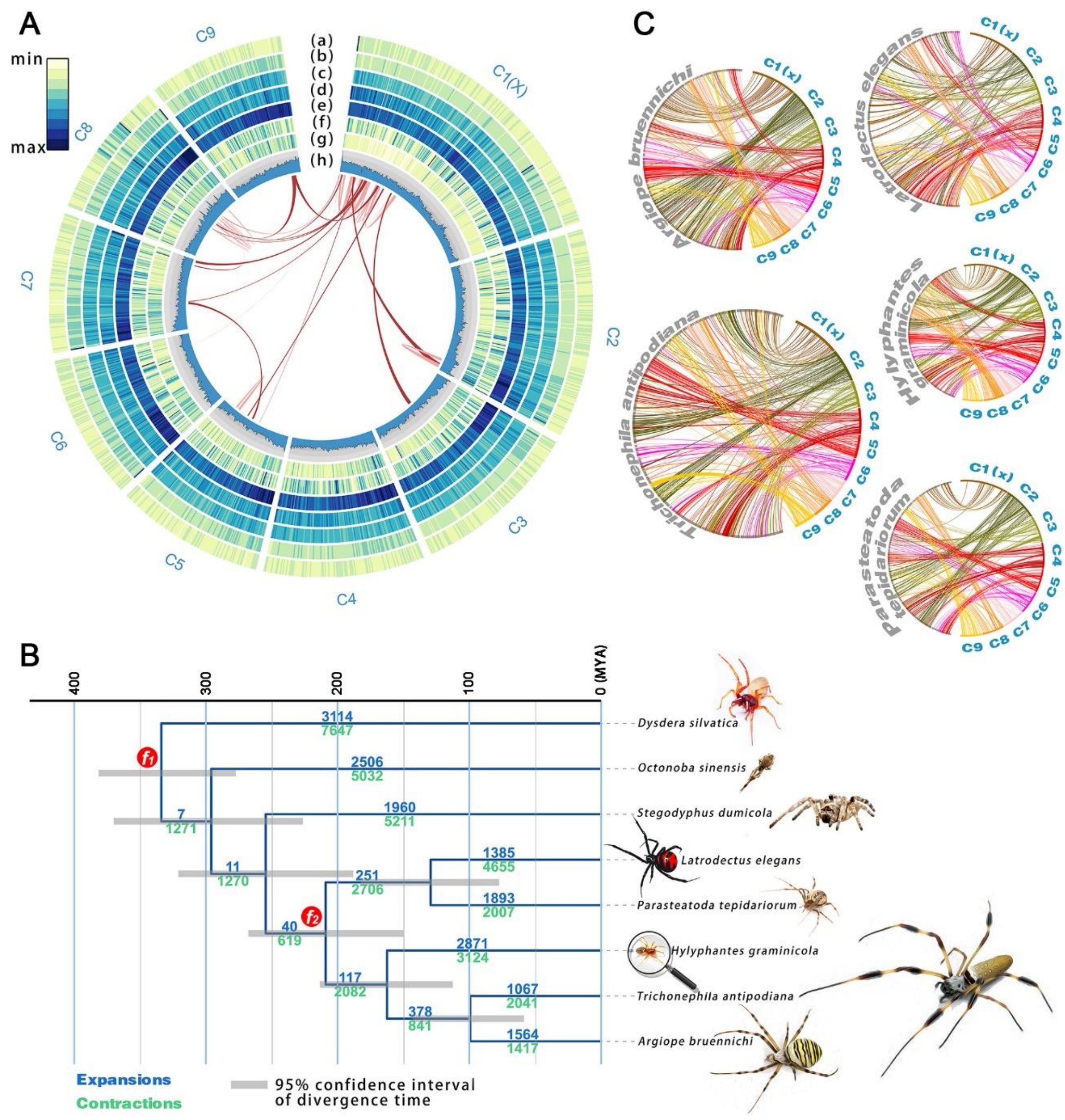

Fig. 2: Genome and phylogeny of *Octonoba sinensis*. (A) Assembly and structural annotation of the *O. sinensis* genome. The distribution of different elements is marked with lowercase letters from the outside to the inside in the circle diagram, a: gene; b: SINE, c: LINE, d: LTR; e: DNA transposon; f: Highly-Conserved Elements (HCEs); g: DNA Sequence Polymorphisms (DnaSPs); h: GC content. The inner lines of the circle graph are collineation, dark red lines are collineation between chromosomes, pink lines represent collineation within the chromosome. (B) Phylogeny of multiple spider species. The 95% confidence interval of the divergence time is represented by the gray strip on

the phylogeny, and the number of expanded and contracted gene families at each node is represented by light blue and light green numbers respectively. The fossil calibration points are marked with red dots, f_1: 276–380 million years ago (MYA) (TIMETREE database, http://www.timet ree.org/), f_2: 98–318 MYA (IZCAS-Ar42702Fo). (C) The collinearity between *O. sinensis* and other spiders. The chromosomes are arranged clockwise from large to small, and the color of the connecting lines is based on the *O. sinensis* chromosomes.

3. Divergence time estimation and synteny analysis

Genomes of model species — the house spider (*Parasteatoda tepidariorum*), *O. sinensis* and six other representative species with good structural and functional annotated genomes (Additional file 4: Table S5) were selected for homologous gene identification (see methods). A total of 18567 gene families (Ortho-groups) were obtained from the eight species, 12785 gene families were identified in *O. sinensis*. In addition, 1791 single copy gene families shared by all species were identified and used to construct the phylogenetic tree (Figure 2B), the topological structure and the divergence time of each node prediction are similar to those of previous studies [20–22].

Among the species involved in the above analysis, genomes with chromosome level assemblies were selected for synteny analysis with *O. sinensis* (Additional file 4: Table S5). Perhaps due to the distant genetic relationship of *Dysdera silvatica*, no effective collinearity relationship can be found, so only the collinearity between *O. sinensis* and five species is shown. In the displayed results, the collinearity regions of *O. sinensis* and *Argiope bruennichi* are the most, 45.26% (608.53 Mb) of all sequences of the *O. sinensis* were in the collinearity segment, followed by the collinearity region with *Hylyphantes graminicola*, which is 44.62% (599.91 Mb), *Trichonephila antipodiana*, which is 43.94% (590.72 Mb), and *P. tepidariorum*, which is 35.52% (477.45 Mb), and finally with *Latrodectus elegans*, which is 31.11% (418.83 Mb) (Figure 2C).

4. The evolution of energy metabolism

In the process of evolution, the appearance and disappearance of any genetic trait are hidden in a vast

amount of information even if the molecular basis of these traits may only be simple changes. To clarify this underlying mechanism, we conducted a series of comparative genomic analyses (see methods). In the analysis of selection pressure, 810 genes were under positive selection (Additional file 4: Table S6). Although these genes did not achieve effective GO enrichment (p. adjust < 0.05), it was still found them were most enriched in the mitochondrial matrix, and, GO terms related to energy metabolism and oxygen adaptation also detected high enrichment (Figure 3A). Through weighted correlation network analysis (WGCNA) of positive selection genes (Additional file 5: Figure S2A, Additional file 4: Table S7), we found that genes in muscle-specific module were also significantly enriched in energy metabolism related items (Additional file 5: Figure S2B, Figure 3BC). Further gene functional annotation indicates that a large number of positive selection signals were found in the most common and conserved glycolysis, tricarboxylic acid cycle, and oxidative phosphorylation pathways in organisms (Additional file 4: Table S6, Figure 3D).

To investigate the potential contributions of the aforementioned findings, we conducted an analysis using the model species *P. tepidariorum* as a control. We examined the activities of several key enzymes, including hexokinase (HK), pyruvate dehydrogenase (PDH), α-ketoglutarate dehydrogenase (α-KGDHC), NADH dehydrogenase (ND), and ATP synthase (ATPase), which are detected to be under positive selection. Our results revealed that the activity levels of these five enzymes in the body of *O. sinensis* were higher compared to those in *P. tepidariorum* (Additional file 5: Figure S2C). Furthermore, while there was no significant difference in CO_2 production rates between *O. sinensis* and *P. tepidariorum* in a resting state, *O. sinensis* exhibited significantly higher rates under fatigue treatment (Additional file 5: Figure2C). These findings suggest that the evolution of energy metabolism pathways may serve as a key factor contributing to the sustained power output of *O. sinensis*.

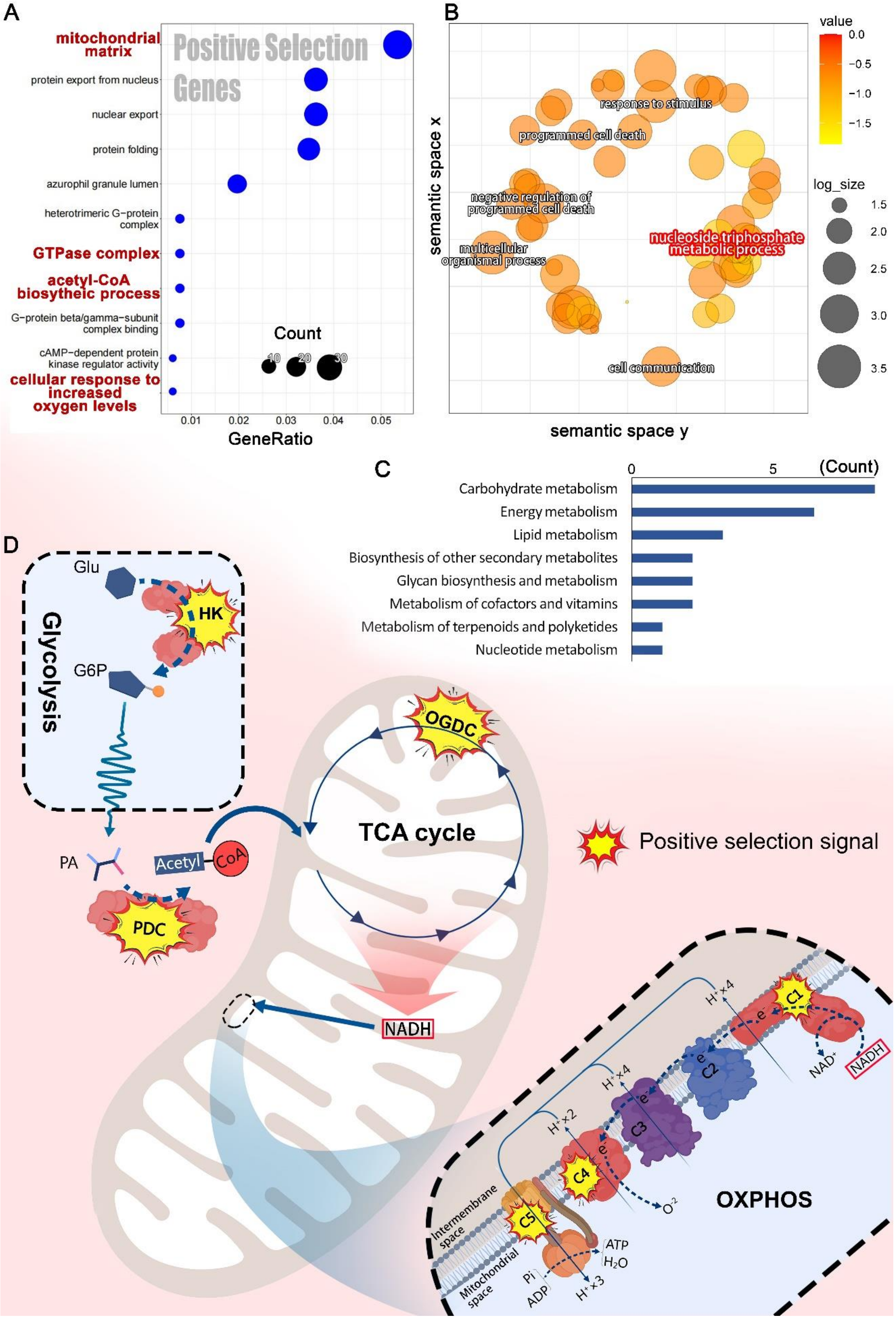
A
mitochondrial matrix
protein export from nucleus
nuclear export
protein folding
azurophil granule lumen
heterotrimeric G-protein complex
GTPase complex
acetyl-CoA biosytheic process
G-protein beta/gamma-subunit complex binding
cAMP-dependent protein kinase regulator activity
cellular response to increased oxygen levels
Positive Selection Genes
Count
10
20
30
0.01
0.02
0.03
0.04
0.05
GeneRatio
B
value
0.0
−0.5
−1.0
−1.5
log_size
1.5
2.0
2.5
3.0
3.5
response to stimulus
programmed cell death
negative regulation of programmed cell death
multicellular organismal process
nucleoside triphosphate metabolic process
cell communication
semantic space x
semantic space y
C
0
5
(Count)
Carbohydrate metabolism
Energy metabolism
Lipid metabolism
Biosynthesis of other secondary metabolites
Glycan biosynthesis and metabolism
Metabolism of cofactors and vitamins
Metabolism of terpenoids and polyketides
Nucleotide metabolism
D
Glu
HK
G6P
Glycolysis
PA
Acetyl
CoA
PDC
OGDC
TCA cycle
Positive selection signal
NADH
C1
C2
C3
C4
C5
NAD+
OXPHOS
Intermembrane space
Mitochondrial space
ATP
H2O
Pi
ADP
O2

Fig. 3: Positive selection signals related to energy metabolism. (A) GO enrichment of genes under positive selection. GO terms related to mitochondrial matrix, energy metabolism and oxygen adaptation are highlighted in red color. (B) REVIGO clusters of the significantly enriched GO terms for muscle-specific module. (C) KEGG enrichment for muscle-specific module. (D) Positive selection signals in the main energy metabolism pathways. Glu: glucose; G6P: Glucose 6-phosphate; HK: Hexokinase; PA: Pyruvic acid; PDC: Pyruvate dehydrogenase complex; OGDC: Oxoglutarate dehydrogenase complex; C1–5: Mitochondrial respiratory complex 1–5; C1: NADH dehydrogenase; C2: Succinate dehydrogenase; C3: Coenzyme Q; C4: Cytochrome c oxidase; C5: ATP synthase.

5. The evolution related to respiratory system and actin

In the results of the GO enrichment of significantly expanded gene families (Additional file 4: Table S8), it was found that these GO terms were most enriched in relation to trachea development and actin regulation (Figure 4A). Further annotation indicates that these GO terms collectively point to the same gene cluster: the Formin homology (FH) protein. Studies have shown that FH proteins not only play a crucial role in the development of the tracheal system in fruit fly [23], but also have significant implications in muscle movement [24, 25]. The significant increase in CO_2 production rate of *O. sinensis* under fatigue treatment may also be related to their well-developed tracheal system. These members of the FH family are distributed among 8 Ortho-groups. We found that the copy number of these Ortho-groups members expanded during and after the divergence between Uloboridae and other groups in the UDOH grade (Figure 4B). (UDOH: spider families of Uloboridae, Deinopidae, Oecobiidae and Hersiliidae, the other three groups all have venom glands) [26].

Generally speaking, spiders lack endurance, and the vast majority of species rely on instantaneous explosive power for hunting [12]. However, species of Uloboridae are able to exercise intensity for nearly an hour [6]. We believe that the evolution of the energy metabolism, respiratory system and actin are related to their endurance.

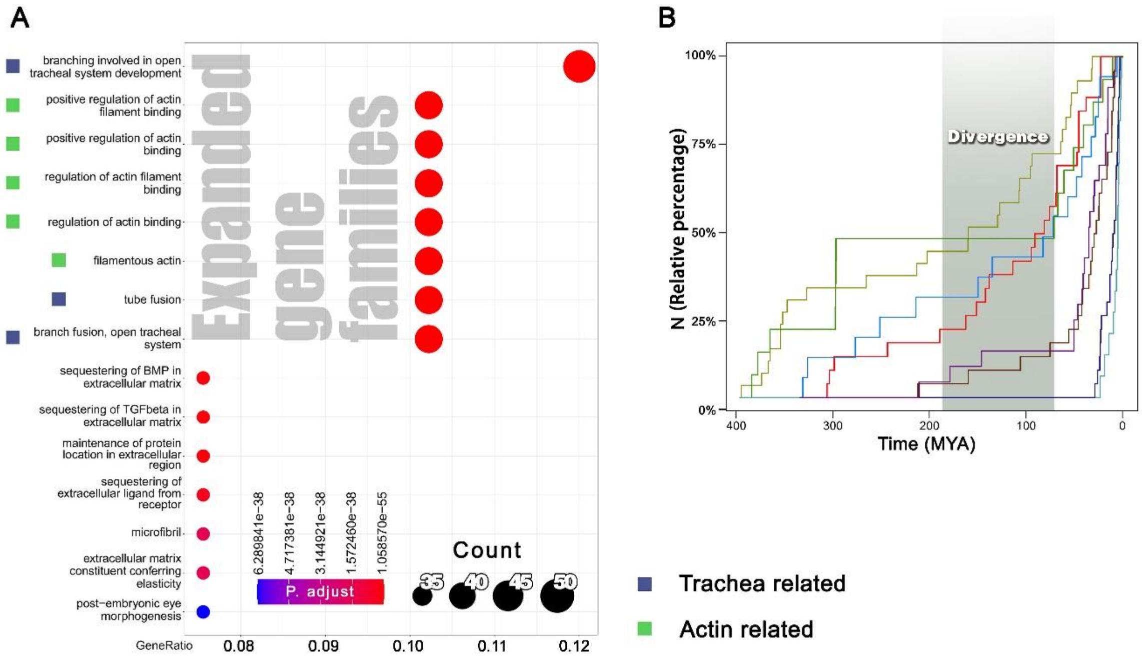

Fig. 4. Significantly expanded gene families. (A) GO enrichment of significantly expanded gene families. (B) The lineages-through time (LTT) plot of trachea development and actin regulation gene families. The gray band is the estimated divergence time between the Uloboridae and the nearest most-closely-related group with venom glands. The GO terms that we are focusing on have been labeled with using different colors: dark blue - trachea related, green - actin related.

6. Absent regions and regions under relaxed selection

The absent regions and regions under selective relaxation in the *O. sinensis* genome were analyzed against the background of species with venom glands (Additional file 4: Table S5). First, we carried out the expansion and contraction analysis of gene families (Figure 2B) and identified the specific genes missing in *O. sinensis* (No gene family experiencing significant contraction was found.) (Additional file 5: Figure S3, Additional file 4: Table S9); We searched for Highly-Conserved Elements (HCEs) spanning eight spider genomes and identified the sites with *O. sinensis* specific deletions among them (Additional file 5: Figure S3, Additional file 4: Table S10 S11). We reconstructed and annotated the absent regions of *O. sinensis* using the *P. tepidariorum* genome as a

reference. In addition, genes under relaxed selection (Additional file 4: Table S12) and the DNA Sequence Polymorphisms (DnaSPs) in the *O. sinensis* genome were identified (Figure 2A, Additional file 4: Table S13). We conducted functional enrichment on the above results and found that the biggest difference between *O. sinensis* and background species comes from the development, especially the neuro-development related, gene family (Figure 5A–D).

Subsequently, we found that the absent regions in the *O. sinensis* genome intersected with 21 genes in the *P. tepidariorum* venom gland specific expression module (VSM, n = 1088, Additional file 4: Table S14) [27], accounting for only 1.96% of the total number of genes in VSM. Through annotation, it was found that these 21 genes are significantly enriched in biological processes related to signal transduction and involve multiple metabolic or signaling pathways (Figure 5E, Additional file 4: Table S15). The intersection of genes under relaxed selection and genes in the VSM consists of 15 genes, accounting for only 1.4% of the VSM. These genes are mainly enriched in GO terms related to development, especially muscle development (Additional file 4: Table S16). All the intersections above do not contain any known toxin-coding genes (Figure 5F).

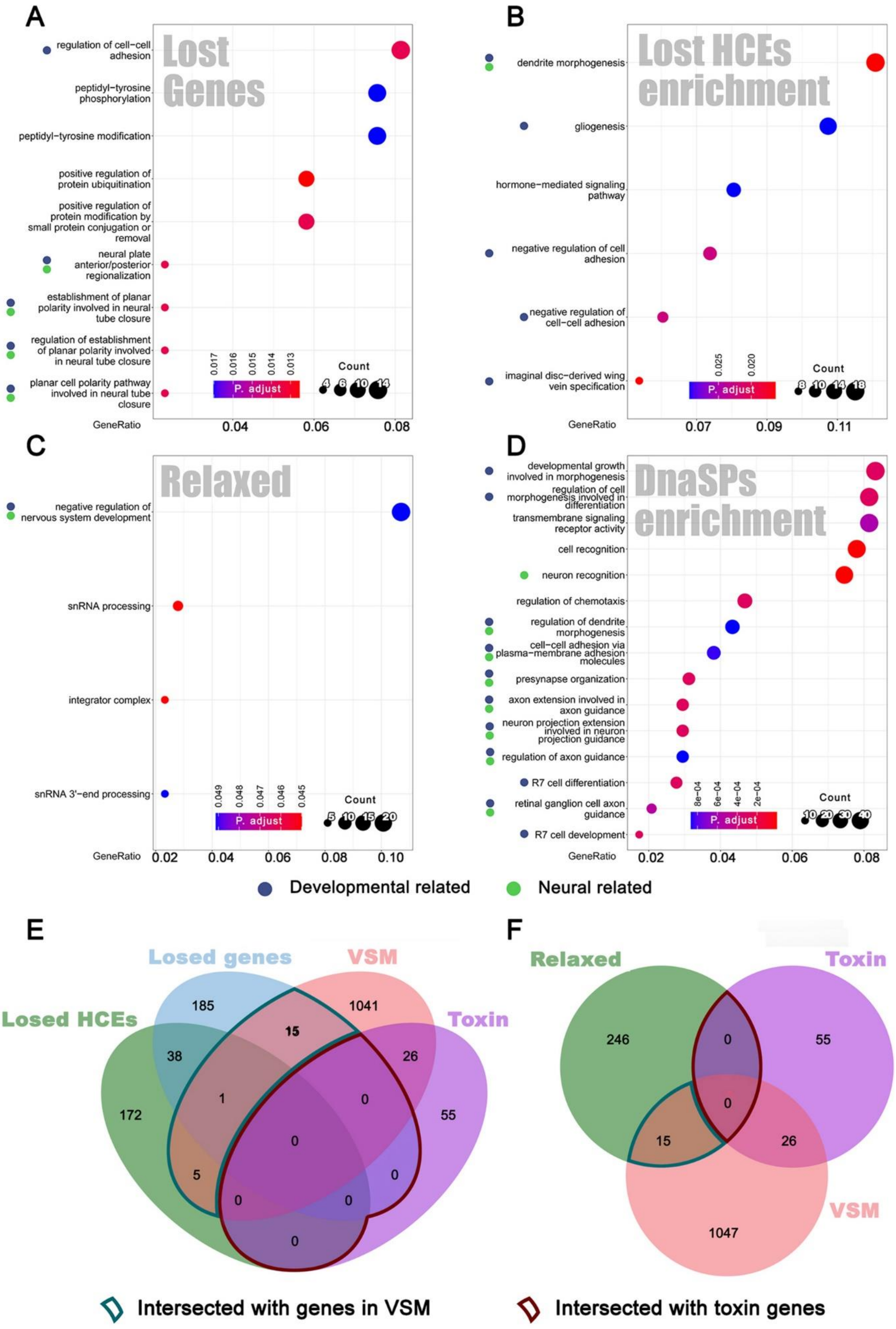
A
Lost Genes
regulation of cell−cell adhesion
peptidyl−tyrosine phosphorylation
peptidyl−tyrosine modification
positive regulation of protein ubiquitination
positive regulation of protein modification by small protein conjugation or removal
neural plate anterior/posterior regionalization
establishment of planar polarity involved in neural tube closure
regulation of establishment of planar polarity involved in neural tube closure
planar cell polarity pathway involved in neural tube closure
P. adjust
Count
GeneRatio
B
Lost HCEs enrichment
dendrite morphogenesis
gliogenesis
hormone−mediated signaling pathway
negative regulation of cell adhesion
negative regulation of cell−cell adhesion
imaginal disc−derived wing vein specification
C
Relaxed
negative regulation of nervous system development
snRNA processing
integrator complex
snRNA 3'−end processing
D
DnaSPs enrichment
developmental growth involved in morphogenesis
regulation of cell morphogenesis involved in differentiation
transmembrane signaling receptor activity
cell recognition
neuron recognition
regulation of chemotaxis
regulation of dendrite morphogenesis
cell−cell adhesion via plasma−membrane adhesion molecules
presynapse organization
axon extension involved in axon guidance
neuron projection extension involved in neuron projection guidance
regulation of axon guidance
R7 cell differentiation
retinal ganglion cell axon guidance
R7 cell development
Developmental related
Neural related
E
Losed genes
VSM
Losed HCEs
Toxin
185
1041
15
38
26
172
1
0
55
0
5
0
0
0
0
F
Relaxed
Toxin
246
0
55
0
15
26
VSM
1047
Intersected with genes in VSM
Intersected with toxin genes

Fig. 5: Absent regions and regions under relaxed selection. (A–B) The missing genes and Highly-Conserved Elements (HCEs) of *O. sinensis* were reconstructed in the *Parasteatoda tepidariorum* genome and GO enrichment based on the background of the *P. tepidariorum* genome. (C) GO enrichment of genes under relaxed selection. (D) GO enrichment of genes related to DnaSPs. The GO terms we focused on in A–D have been labeled using different colors: dark blue - development related, green- neural related. (E) The intersection among absent regions of *O. sinensis* genomes, genes in the venom gland specific expression module (VSM), and toxin genes. (F) The intersection among genes under relaxed selection, genes in the VSM, and toxin genes. In the Venn diagrams of E and F, the intersection we focus on is represented by different colored wireframes: dark blue - intersected with genes in the VSM, dark red - intersected with toxin genes.

7. Differential retention of major toxin genes in *O. sinensis*

In the *O. sinensis* genome, we have identified 12 toxin genes belonging to different gene families (six major families: Latrotoxin, Latodectin, CRISP, ICK, TCTP, EF-hand, ctenitoxin.), less than in most spider species studied , but more than *D. silvatica* [28–31] (Additional file 4: Table S17). Our transcriptome data show that different kinds of toxin genes are expressed in all tissues of *O. sinensis*, but most toxins have the highest expression in the brain. The homologues of toxin genes retained in *O. sinensis* were not expressed in the *P. tepidariorum* venom gland either (Figure 6).

To further explore the evolutionary process of *O. sinensis* toxin genes after the absence of venom glands, we conducted a pseudogene search on non-coding regions of the *O. sinensis* genome, but traditional search methods did not identify pseudogenes (blastn, E-value 10^{-5}, matching length 50 bp) [32]. However, we did find some traces of toxin genes in the blastx search through the protein sequence of toxin genes. These results include 48 different genomic regions, 36 of which belong to the Latrotoxins gene family. One matches best with the toxin gene that is highly expressed in the venom gland of *P. tepidariorum*. Moreover, this gene (LOC107440051) is an alpha-latroinsectoxin which is effective for insects (Additional file 4: Table S18, Figure 6). By searching for toxin genes in collinearity fragments

of *O. sinensis* and *P. tepidariorum*, it can also be found that the toxin genes that are highly expressed in *P. tepidariorum* venom glands are not present in *O. sinensis*, and these include two types of toxins: alpha-latrotoxin, delta-latroinsectotoxin, that specifically target vertebrates and insects (Figure 7). Although they do not conform to the traditional definition of pseudogenes, these signs all suggest that *O. sinensis* once possessed venom that could be used for hunting and self-defense. Moreover, our results indicate that these toxin genes are quickly removed from the genome after losing the constraints of selection pressure.

In addition, we also examined a class of genes in *O. sinensis*. These genes are located in the same place as the *P. tepidariorum* toxin gene in the collinearity segment, but they can no longer be identified as toxin genes (below the minimum recognition threshold, see methods) (Figure 7B, red ribbon). Compared with other toxin genes in which it is difficult to find pseudogenes, these genes have complete gene structures and CDS regions. Transcriptome data also show that they genes can be normally transcribed into mRNA (Additional file 6). We speculate that these genes may have undergone new-functionalization.

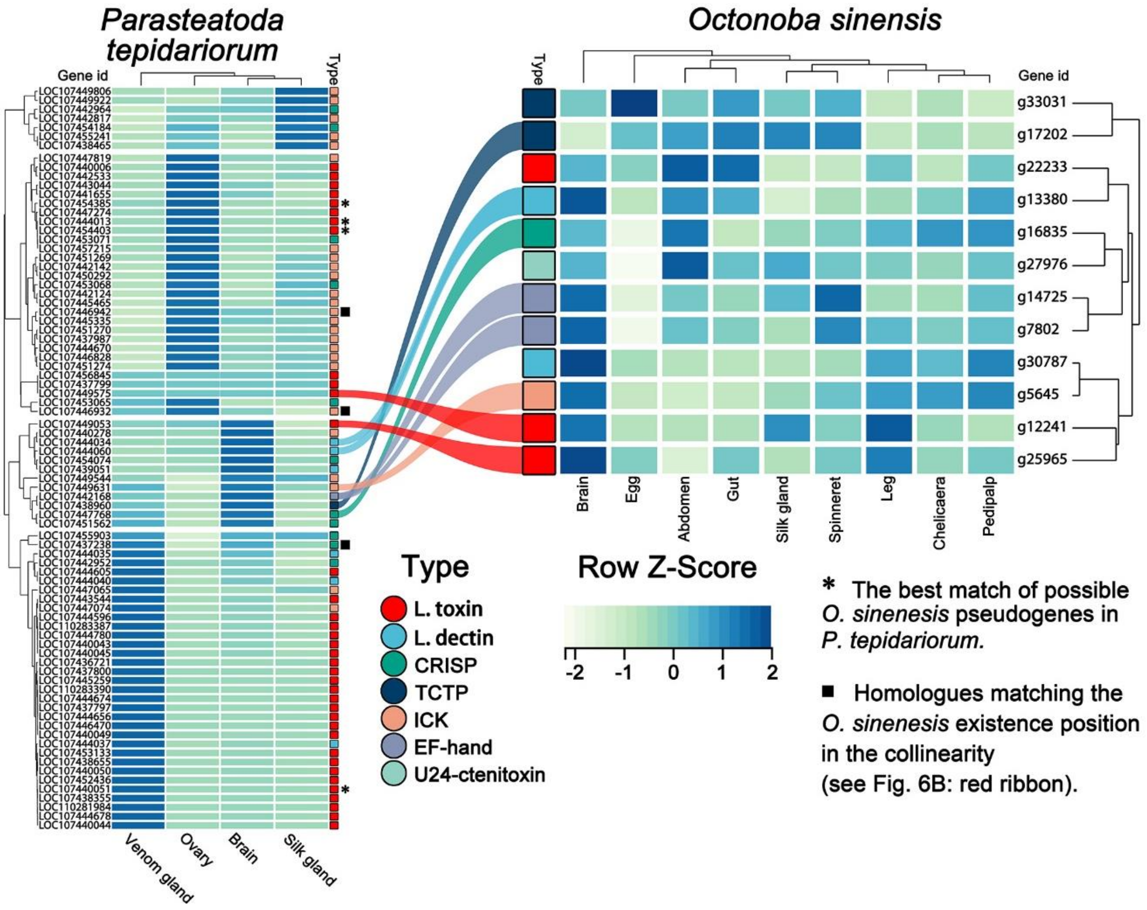

Fig. 6. The expression patterns of toxin genes in *Octonoba sinensis* and *Parasteatoda tepidariorum*. The ribbon connects the homologous genes. The black labels (asterisks and squares) indicate genes found in *P. tepidariorum* that are related to traces of the *O. sinensis* genome.

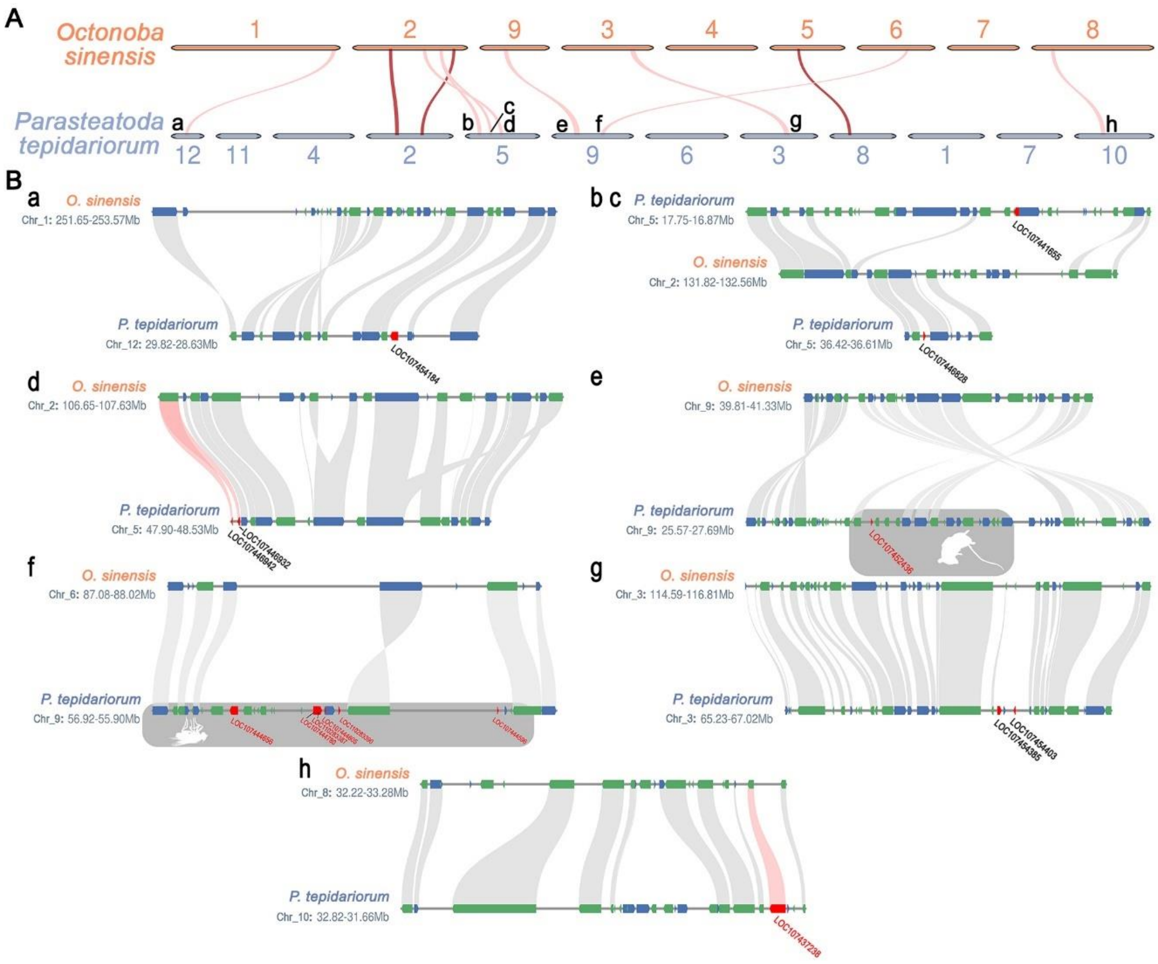

Fig. 7. Collinearity containing toxin genes between *Octonoba sinensis* and *Parasteatoda tepidariorum*. (A) Collinearity of all toxin genes. If the toxin gene is lost in the *O. sinensis*, the collinearity fragments are represented by a pink ribbon. (B) Gene correspondence in each collinearity fragment where toxin gene deletion occurs in A. Each lowercase letter corresponds to the collinearity fragments represented by the same letter in A; The toxin gene names in *P. tepidariorum* are listed, and the red gene names highlight the toxin genes that are highly expressed in the venom glands. The toxin genes in the gray background belong to the Latrotoxin gene family, and different silhouettes indicate the type of toxin and the target group of toxicity. Mouse = vertebrates (alpha-latrotoxin), Fly = insects (delta-latrotoxin); The red ribbons indicates that the collinearity of the linked genes is extremely weak, below the threshold (see methods).

Discussion

During the process of evolution, the loss of important organs is caused by a series of developmental changes. Typical examples include various species of eyeless organisms [33–37], as well as limb degeneration in some quadrupeds [38–40]. We summarized the specific differences in the genome of *O. sinensis* relative to the background and found that many genes related to absent regions and regions under relaxed selection were enriched in developmental GO terms (Fig 3A–D), which was consistent with previous studies [32–39]. Simultaneously, using venom or controlling venom glands is a complex neural activity [41–43]. Our results show that the enrichment of GO terms related to specialized neuro-development suggests that some of these genes may be involved in the development of the nervous control system of venom glands.

Although the web-building behavior of spiders is well known, the venom system seems more important than the former. Using webs for predation is at least 50 million years (MYs) later than the role of venom glands in hunting [21], moreover, among the youngest and diverse Retrolateral Tibial Apophysis (RTA) clade, the vast majority of spiders abandoned webs in predatory activities. This resulted in most extant spiders relying solely on the injection of toxins to immobilize their prey [26, 44]. From published data, species without venom glands are rare, and all spider species without venom glands are adept at relying on webs for predation [1, 12], this seems to be a trade-off. Within the orb-weaving spiders (Araneoidea), many species first use entanglement to restrain large prey and then use venom [11]. Although entanglement is extremely crude compared to the behavior of Uloboridae species, this predatory strategy may lessen selective pressure for the use of venom. Therefore, ingenious orb-weaving and prey wrapping behavior may be a “preadaptation” [3] or “exaptation” [45] of the absence of venom glands. In Uloboridae, the behavior of wrapping prey is highly specialized, and the high-intensity and lengthy physical output requires extremely high endurance. Previous studies have shown that the entire process can last from a few minutes to nearly an hour, and the spider silk used can reach over a hundred meters [4–6]. The most interesting discovery in this study is that there were many genes under positive selection in the energy metabolism pathway, and the *O. sinensis* trachea and actin related gene families have experienced expansion. We believe that these evolutionary characteristics provide a prerequisite for the endurance required for continuous prey wrapping.

Our results indicate that all major types of toxin gene can be identified in the *O. sinensis* genome (Additional file 4: Table S17). In the model species *P. tepidariorum*, a large number of toxin genes identified in previous studies were not highly expressed in the venom glands. A long time ago, toxins have been detected in other parts of the spider's body [46, 47]. Some studies have also explored the toxicity of these toxins [48–50] and demonstrated that those not expressed in venom glands do have a certain impact on predators [46]. Therefore, we believe that the crossover between "Venom (Trauma toxicity)" function and "Poison (Oral toxicity)" function is one of the reasons for the whole-body expression of some toxin types.

In addition, the expression of neurotoxin genes in the brain (Figure 6), the role of CRISP in animal reproductive processes [51], and that some non-venom gland expressed toxin genes are often located at the base of the phylogenetic tree (Additional file 5: Figure S4). Based on this, we speculate that the current toxin genes may have originally played non-toxic roles in other tissues. During evolution, some homologues are mobilized in the venom glands, and under the action of natural selection, the toxic function of these homologues are amplified and gradually evolved into specialized toxin genes. Thus, different spider groups have different selection pressures for toxins, ultimately resulting in such diverse and complex toxin compositions in extant species.

Methods

1. Sample collection and DNA extraction

Live spiders of *O. sinensis* were field-captured from Olympic Park, Chaoyang District, Beijing, China. We sampled the same population several times over a two-year time span to establish a database for identifying DnaSPs. All samples were starvation reared for more than one week at room temperature. Genome DNA for both short and long read sequencing were isolated from the cephalothoraxes of adult spiders using the Qiagen Blood & Cell Culture DNA Kit (QIAGEN, Hilden, Germany).

2. Observation of predation behavior and examination of fangs

We recorded a series of videos to observe the predation behavior. Wrapping duration was timed. If the spider stopped for more than 3 seconds, the timing also stopped, but would continue if it began again.

If the spider begins to soak (*O. sinensis* will spit out liquid to soak their prey before eating) or leave the prey, the video is terminated.

The dissected spider fangs were pasted onto a copper substrate at different angles, dried with a CO_2 critical point drying method, coated with gold, and then observed with the SEM (model: FEI Quanta 450).

3. Genome sequencing and genome size estimation

Short-insert (300–400 bp) libraries of *O. sinensis* were sequenced with the MGISEQ-2000 platform using paired-ends (PE) 150 bp (BGI, Shenzhen, China). To remove low-quality reads and adapters, raw reads were trimmed by Trimmomatic v0.39 [52]. A total of 259.82 Gb of clean data were obtained for *O. sinensis* for survey analysis, assembly correction, and subsequent genome DnaSPs search.

For long read sequencing, 'SMRTbell' (double-stranded DNA template capped by hairpin loops at both ends) libraries were constructed according to the standard protocol of PacBio using 15 kb of preparation solution (PacBio, California, USA). The high-fidelity (HiFi) libraries were sequenced on three SMRT cells on the PacBio Sequel II system in Circular Consensus Sequencing (CCS) mode We generated 67.18 Gb HiFi data (3,967,026 reads) total [53].

To further improve the continuity of the assembled genomes, chromosome conformation capture (Hi-C) experiments was conducted [54]. Hi-C libraries were prepared following a published protocol with minor modifications [55]. For cross-linking, samples were fixed with 1% formaldehyde. The cross-linked DNA was digested with MboI restriction endonuclease and marked with biotin-14-dCTP to remove non-ligated DNA fragments. The ligated DNA was extracted with a QIAamp DNA Mini Kit (QIAGEN). The purified DNA was then sheared to ~350 bp fragments and followed by a standard Illumina library preparation protocol [56]. Hi-C sequencing of *O. sinensis* was conducted on the MGISEQ-2000 platform with PE 150 bp (BGI, Shenzhen, China). We then filtered the raw reads using Juicer v1.6.2 [57] to remove low-quality reads and adapters, yielding 201.99 Gb of clean data.

Before *de novo* assembly, we estimated the genome size of this species. Using the Illumina data, Jellyfish (v2.1.3) [58] was employed to calculate the frequency of each K-mer (k = 17~31). Then, the genome size of *O. sinensis* was estimated using a method based on K-mer distribution.

4. *De novo* Genome assembly and quality assessment

PacBio reads were first assembled using two *de novo* assemblers: hifiasm v0.15.2 [59] and wtdbg2 v2.5 [60]. The best assembly was selected according to the optimal continuity and completeness. The final version of contigs was polished with Racon v1.4.17 for three rounds (https://github.com/isovic/racon), based on long reads, and NextPolish v1.4.0 [61], using short reads. Contig level genome completeness assessment was performed using BUSCO v5.2.2 [14, 15]. Genome consistency assessment was evaluated by mapping the short reads to the genome with Minimap2 v2.24-r1122 [62] and Samtools v1.10 [63].

We used Hi-C-based proximity-guided assembly to generate chromosomal level genome assemblies for *O. sinensis*. Hi-C library sequencing data were mapped to the contig level genome using Juicer. The 3D-DNA v180922 [64] pipeline was executed to construct the chromosomes and correct the errors. We further performed correction with Juicebox Assembly Tools v1.11.08 [65]. The completeness of the chromosomal level assembly was assessed by BUSCO.

5. RNA extraction and sequencing

For gene annotation, different tissue samples (spinneret, leg, brain, gut, silk gland, pedipalp, chelicera, abdomen and eggs) (Additional file 6) were dissected for total RNA extraction using an RNAsimple Total RNA kit (TIANGEN, Beijing, China). The RNA-seq libraries were constructed with insert sizes of ~150 bp and sequenced on the NovaSeq 6000 platform. We produced ~6 Gb data per sample. Low-quality reads, reads with adapters, and unknown bases were filtered using Trimmomatic.

For gene expression pattern analyses, we further collected four tissue transcriptomes (including brain, ovary, silk gland and venom gland) of *P. tepidariorum*.

6. Genome annotation

The RepeatModeler v2.0.2 [66] and RepeatMasker v4.1.2-p1 [67] pipelines were used to annotate repetitive sequences in genome.

Gene annotation was based on the braker v2.1.6 [68] pipeline, which combines the whole protein sequences of the eight species in this study (Additional file 4: Table S5) and more than 320 Gb multi tissue transcriptome data for comprehensive annotation.

Gene function annotation is based on NCBI-Nr (http://www.ncbi.nlm.nih.gov), Swiss-Prot (http://www.uniprot.org/) and EggNOG v5.0 (http://eggnog5.embl.de/) databases. The tRNA was predicted using the program tRNAscan-SE v2.09 [69]. Other non-coding RNAs were annotated with the Rfam v14.8 [70] database through infernal v1.1.4 [71].

7. Orthologous gene identification and phylogenetic analysis

We used Orthofinder v2.5.4 [72] to analyze the annotation information of species in this study (Additional file 4: Table S5). In the pipeline, Mafft v7.453 [73] was used to perform multiple sequence alignment, blastp v2.9.0+ [74] was used to perform sequence searches, and the phylogenetic tree was constructed using FastTree v2.1.11 [75].

8. Synteny analysis and gene family expansions/contractions

Collinearity analysis was conducted between *O. sinensis* and other species with chromosome level genomes (*T. antipodiana*, *A. bruennichi*, *L. elegans*, *H. graminicola*, *D. silvatica*.) using the MCscan pipeline (Python version) [76] in the jcvi toolkit.

For gene family expansions/contractions, mcmctree in the paml v4.9j toolkit [77] was used to estimate the divergence time of each node in the phylogenetic tree. The two calibration points came from the TIMETREE database (http://www.timet ree.org/) and the fossil record (IZCAS-Ar42700Fo) (Figure 1D). Next, we used Cafe v4.2 [78] under the default parameters to analyze the gene family expansions and contractions of eight spider species. The R script Reconciliation-Assisted Divergence Time Estimation (RADTE) was used to estimate the divergence time among members of gene families that were found to be significantly expanded in *O. sinensis* with the divergence time of each node of the species tree as input.

9. Toxin gene family identification and analysis

Two previous datasets were used as references (Additional file 7) [30][79], and target genes were searched for using blastp v2.9.0+ (E-value less than 1e-20, matching length greater than 70% of the reference sequence, and hit area mismatch less than 30%). We searched for toxin genes of *O. sinensis* and in the annotation information of the *P. tepidariorum* genome, which was used as a reference. For the search of hidden toxin-related pseudogenes in *O. sinensis*, we referred to the identification criteria

of human genome pseudogenes [32], and use blastn v2.9.0+ and blastx v2.9.0+ to search for candidates.

10. Identification of HCEs and DnaSPs

To identify the HCEs, we initially generated pairwise sequence alignments across all eight spider genomes of the eight species (Additional file 4: Table S5) with LASTZ v1.04.15 [80] and chainNet [81], using the *P. tepidariorum* genome as the reference. We then used MULTIZ v11.2 [82] to combine the pairwise alignments into multiple sequence alignments. Subsequently, we ran phyloFit in the PHAST package [83] with the topology from Orthofinder to estimate the neutral ('nonconserved') model based on fourfold degenerate sites. With the nonconserved model as input, we ran phastCons [84] to estimate conserved models with its intrinsic function and predicted the HCEs.

All clean short reads of DNA sequence of *O. sinensis* were used for the DNA Sequence Polymorphisms (DnaSPs) search. We used snappy v4.6.0 (https://github.com/tseemann/snippy/) with default parameters to search based on the assembled *O. sinensis* genome.

The distribution of HCEs and DnaSPs in exons, introns, 2000 bp upstream and 2000 bp downstream of genes, and intergenic regions was summarized with Annovar (https://annovar.openbioinformatics.org/) [85, 86] based on the genome annotation information of *O. sinensis* or *P. tepidariorum*.

11. Test for relaxation of selection and positive selection

To statistically test which genes of *O. sinensis* are under relaxed purifying selection, we used RELAX in the HYPHY v2.5.2 [87] toolkit to infer the free relaxation parameter k of *O. sinensis* branches for gene families shared by the eight species (Additional file 4: Table S5). The relaxation parameter k is an exponent for selection parameters between the foreground and the background branches. A $k > 1$ suggests selection is more intensified in the foreground branch vs. the background branch and vice versa. For all species, we used the *P. tepidariorum* genome as a reference to extract the Reciprocal Best Hits (RBH) based on blast. Finally, 3900 RBH clusters (Additional file 4: Table S19) were retained for analysis.

To scan for genes under positive selection in *O. sinensis*, the 3900 RBH clusters were used for branch site model analysis using the CODEML in PAML. Each gene family sets *O. sinensis* as foreground branches. "Model A" and "Model A-null" models were compared. "Model A" assumes that the selection

pressure of foreground branches is greater than that of background branches, and "Model A-null" is an alternative hypothesis.

12. Physiological index measurement

Assays were performed as described previously[88]. CO_2 production rate was used as a proxy for metabolic rate (MR). The assays were conducted in a closed-circuit system with a volume of 73.3 ml at a temperature of 25 °C, a pressure of 100.6 kPa, and a flow rate of 110 ml/min. MR was calculated as the amount of CO_2 produced per gram of body mass per second, using the equation MR = MCO_2/T/body mass, where MCO_2 represents the amount of oxygen substance (in mol). In order to put spiders into a state of fatigue, stimulating the spider's feet with a dissecting needle and keep it in a high-intensity state of exercise for ten minutes.

Enzyme activity is measured using the corresponding reagent kit (Wuhan Mosak Biotechnology Co., Ltd. KT50129, KT50577, KT42310, KT41589, KT87867). Female spider individuals in a resting state are fixed in liquid nitrogen after weighing and stored at -80 °C. Prior to testing, homogenize the sample and dilute it to 500 μl as the test solution. Follow the supplier's strategy for testing during the testing process.

Additional Files

Additional file 1–3: Hunting video.

Additional file 4: Table S1. Genome survey prediction of *Octonoba sinensis*. **Table S2.** Illumina reads map of the *Octonoba sinensis* genome. **Table S3.** Repeat sequences of *Octonoba sinensis* genome. **Table S4.** Genome assembly. **Table S5.** The genome used in this study. **Table S6.** Genes of *Octonoba sinensis* under positive selection pressure. **Table S7.** Weighted correlation network analysis (WGCNA) of positive selection genes in *Octonoba sinensis*. **Table S8.** Significantly expanded gene families in *Octonoba sinensis*. **Table S9.** Missing orthologous groups in *Octonoba sinensis*. **Table S10.** All HCEs, using the *Parasteatoda tepidariorum* genome as a reference. **Table S11.** Missing HCEs in *Octonoba sinensis*. **Table S12.** Genes of *Octonoba sinensis* under relaxed selection pressure. **Table S13.** Annotation of DnaSPs in *Octonoba sinensis*. **Table S14.** *Parasteatoda tepidariorum* venom gland-

specific expression module. **Table S15.** Intersection of genome missing information and the venom gland specific expression module. **Table S16.** Intersection of genes under relaxed selection pressure and the venom gland specific expression module. **Table S17.** Quantitative distribution of different toxin genes across species. **Table S18.** "Pseudogene" blastx. **Table S19.** Clusters of Reciprocal Best Hits.

Additional file 5: Figure S1. Recognizable elements of the *Octonoba sinensis* genome. **Figure S2.** Analysis of *Octonoba sinensis* positive selection genes and physiological index measurement. **Figure S3.** Distribution of all Highly-Conserved Elements (HCEs) in *Parasteatoda tepidariorum*, and those missing HCEs and genes in *Octonoba sinensis*. **Figure S4.** Phylogenetic tree of neurotoxin genes (latrotoxin).

Additional file 6: Expression Matrix of *Octonoba sinensis*.

Additional file 7: Dataset of toxin reference genes.

Additional file 8: Main analysis script.

Acknowledgements

We are grateful to Prof. Zhonge Hou and Asst. prof. Fengyuan Li for academic suggestions. We thank Wei Wang at Guangxi Normal University for her suggestions on anatomical techniques. Finally, Y.M.Z. wants to thank Lingling Liu, and in particular, for the invaluable support over the years.

Authors' contributions

S.Q.L. and Y.M.Z. conceived and designed the project. Y.X.S. and Y.M.Z. finished the genome assembly and annotation. B.Y.Z., P.Y.J., Y.X.S., and Y.M.Z executed the comparison analysis. Y.J.L. identified the species and provided the spider pictures used in the article. Y.W. recorded the original hunting videos, and Y.M.Z. edited these videos. All authors participated in the discussion. The authors read and approved the final manuscript.

Funding

This study was supported by the Strategic Priority Research Program of the Chinese Academy of Sciences (XDB31000000).

Availability of data and materials

All data generated or analyzed during this study are included in this article, its supplementary information files, and publicly available repositories. Original sequencing data has been uploaded to the NCBI database (in progress). New chromosome-level genome assemblies are deposited in the ScienceDB Digital Repository (https://www.scidb.cn/s/zmqyAb).

Declarations

Ethics approval and consent to participate

Not applicable.

Consent for publication

Not applicable.

Competing interests

The authors declare that they have no competing interests.

References:

[1] Foelix RF, Erb B. Mesothelae have venom glands., 2010.

[2] Opell BD. Revision of the genera and tropical American species of the spider family Uloboridae. Bulletin of the Museum of Comparative Zoology at Harvard College 1979;148:443.

[3] Darwin C. The Origin of Species. Philadelphia: University of Pennsylvania Press, 1859.

[4] Weng JL, Barrantes G, Eberhard WG. Feeding by Philoponella vicina (Araneae, Uloboridae) and how uloborid spiders lost their venom glands. CAN J ZOOL 2006;84:1752.

[5] Eberhard WG, Barrantes G, Weng JL. Tie them up tight: wrapping by Philoponella vicina spiders breaks, compresses and sometimes kills their prey. SCI NAT-HEIDELBERG 2006;93:251.

[6] LUBIN YD. Web buiding and prey capture in the Uloboridae. Spiders:Webs, Behavior, and Evolution. 1986:132.

[7] Opell BD. The relationship of book lung and tracheal systems in the spider family uloboridae. J MORPHOL 1990;206:211.

[8] Opell BD. The respiratory complementarity of spider book lung and tracheal systems. J MORPHOL 1998;236:57.

[9] de Plancy VC. Arachnides recueillis aux environs de Pékin. In: SIMON E, edito. Annales de la Société Entomologique de France. Saint-Germain, 1880.

[10] NMBE - World Spider Catalog., vol. 2023.

[11] Robinson MH, Olazarri J. Units of behavior and complex sequences in the predatory behavior of

Argiope argentata (Fabricius): (Araneae: Araneidae)., 1971.

[12] Rainer F. Biology of Spiders: Oxford university press, 2011.

[13] Wang X, Wang Y, Yang Z, Cui S. On the Karyotype of Octonnba sinensis. Journal of Hebei Normal University (Natural Science) 1997:423.

[14] Seppey M, Manni M, Zdobnov EM. BUSCO: Assessing Genome Assembly and Annotation Completeness. Methods Mol Biol 2019;1962:227.

[15] Simao FA, Waterhouse RM, Ioannidis P, Kriventseva EV, Zdobnov EM. BUSCO: assessing genome assembly and annotation completeness with single-copy orthologs. BIOINFORMATICS 2015;31:3210.

[16] Hu W, Jia A, Ma S, Zhang G, Wei Z, Lu F, Luo Y, Zhang Z, Sun J, Yang T, Xia T, Li Q, Yao T, Zheng J, Jiang Z, Xu Z, Xia Q, Wang Y. A molecular atlas reveals the tri-sectional spinning mechanism of spider dragline silk. NAT COMMUN 2023;14.

[17] Fan Z, Yuan T, Liu P, Wang L, Jin J, Zhang F, Zhang Z. A chromosome-level genome of the spider Trichonephila antipodiana reveals the genetic basis of its polyphagy and evidence of an ancient whole-genome duplication event. GIGASCIENCE 2021;10.

[18] Liu S, Aagaard A, Bechsgaard J, Bilde T. DNA Methylation Patterns in the Social Spider, Stegodyphus dumicola. GENES-BASEL 2019;10:137.

[19] Huerta-Cepas J, Szklarczyk D, Heller D, Hernandez-Plaza A, Forslund SK, Cook H, Mende DR, Letunic I, Rattei T, Jensen LJ, von Mering C, Bork P. eggNOG 5.0: a hierarchical, functionally and phylogenetically annotated orthology resource based on 5090 organisms and 2502 viruses. NUCLEIC ACIDS RES 2019;47:D309.

[20] Magalhaes I, Azevedo G, Michalik P, Ramirez MJ. The fossil record of spiders revisited: implications for calibrating trees and evidence for a major faunal turnover since the Mesozoic. Biol Rev Camb Philos Soc 2019.

[21] Shao L, Zhao Z, Li S. Is phenotypic evolution affected by spiders' construction behaviors? SYST BIOL 2022.

[22] Wheeler WC, Coddington JA, Crowley LM, Dimitrov D, Goloboff PA, Griswold CE, Hormiga G, Prendini L, Ramirez MJ, Sierwald P, Almeida-Silva L, Alvarez-Padilla F, Arnedo MA, Benavides SL, Benjamin SP, Bond JE, Grismado CJ, Hasan E, Hedin M, Izquierdo MA, Labarque FM, Ledford J, Lopardo L, Maddison WP, Miller JA, Piacentini LN, Platnick NI, Polotow D, Silva-Davila D, Scharff N, Szuts T, Ubick D, Vink CJ, Wood HM, Zhang J. The spider tree of life: phylogeny of Araneae based on target-gene analyses from an extensive taxon sampling. CLADISTICS 2017;33:574.

[23] Matusek T, Djiane A, Jankovics F, Brunner D, Mlodzik M, Mihaly J. The Drosophila formin DAAM regulates the tracheal cuticle pattern through organizing the actin cytoskeleton. DEVELOPMENT 2006;133:957.

[24] Iskratsch T, Ehler E. Formin-g muscle cytoarchitecture. Bioarchitecture 2011;1:66.

[25] Valencia DA, Quinlan ME. Formins. CURR BIOL 2021;31:R517.

[26] Shao L, Li S. Early Cretaceous greenhouse pumped higher taxa diversification in spiders. MOL PHYLOGENET EVOL 2018;127:146.

[27] Zhu B, Jin P, Zhang Y, Shen Y, Wang W, Li S. Genomic and transcriptomic analyses support a silk gland origin of spider venom glands. BMC BIOL 2023;21.

[28] Luo J, Ding Y, Peng Z, Chen K, Zhang X, Xiao T, Chen J. Molecular diversity and evolutionary trends

of cysteine-rich peptides from the venom glands of Chinese spider Heteropoda venatoria. Sci Rep 2021;11:3211.

[29] Wang Z, Zhu K, Li H, Gao L, Huang H, Ren Y, Xiang H. Chromosome-level genome assembly of the black widow spider Latrodectus elegans illuminates composition and evolution of venom and silk proteins. GIGASCIENCE 2022;11.

[30] Zhu B, Jin P, Hou Z, Li J, Wei S, Li S. Chromosomal-level genome of a sheet-web spider provides insight into the composition and evolution of venom. MOL ECOL RESOUR 2022;22:2333.

[31] Escuer P, Pisarenco VA, Fernandez-Ruiz AA, Vizueta J, Sanchez-Herrero JF, Arnedo MA, Sanchez-Gracia A, Rozas J. The chromosome-scale assembly of the Canary Islands endemic spider Dysdera silvatica (Arachnida, Araneae) sheds light on the origin and genome structure of chemoreceptor gene families in chelicerates. MOL ECOL RESOUR 2022;22:375.

[32] Zhang Z, Carriero N, Zheng D, Karro J, Harrison PM, Gerstein M. PseudoPipe: an automated pseudogene identification pipeline. BIOINFORMATICS 2006;22:1437.

[33] Protas ME, Trontelj P, Patel NH. Genetic basis of eye and pigment loss in the cave crustacean, *Asellus aquaticus*. Proceedings of the National Academy of Sciences 2011;108:5702.

[34] Gore AV, Tomins KA, Iben J, Ma L, Castranova D, Davis A, Parkhurst A, Jeffery WR, Weinstein BM. An epigenetic mechanism for cavefish eye degeneration. bioRxiv 2017:199018.

[35] Mojaddidi H, Fernandez FE, Erickson PA, Protas ME. Embryonic origin and genetic basis of cave associated phenotypes in the isopod crustacean Asellus aquaticus. SCI REP-UK 2018;8:16589.

[36] Piatigorsky J. A Genetic Perspective on Eye Evolution: Gene Sharing, Convergence and Parallelism. Evolution: Education and Outreach 2008;1:403.

[37] Krishnan J, Rohner N. Cavefish and the basis for eye loss. Philos Trans R Soc Lond B Biol Sci 2017;372.

[38] Saxena A, Cooper KL. Diversification of the vertebrate limb: sequencing the events. CURR OPIN GENET DEV 2021;69:42.

[39] Yi H. How Snakes Came to Slither. SCI AM 2017;318:70.

[40] Mann A, Pardo JD, Maddin HC. Snake-like limb loss in a Carboniferous amniote. NAT ECOL EVOL 2022;6:614.

[41] Morgenstern D, King GF. The venom optimization hypothesis revisited. TOXICON 2013;63:120.

[42] Bordon K, Cologna CT, Fornari-Baldo EC, Pinheiro-Junior EL, Cerni FA, Amorim FG, Anjolette F, Cordeiro FA, Wiezel GA, Cardoso IA, Ferreira IG, de Oliveira IS, Boldrini-Franca J, Pucca MB, Baldo MA, Arantes EC. From Animal Poisons and Venoms to Medicines: Achievements, Challenges and Perspectives in Drug Discovery. FRONT PHARMACOL 2020;11:1132.

[43] Utkin YN. Animal venom studies: Current benefits and future developments. World J Biol Chem 2015;6:28.

[44] Rainer F, Bruno E. Mesothelae have venom glands. The Journal of Arachnology 2010;38:596.

[45] Gould SJ, Vrba ES. Exaptation—a Missing Term in the Science of Form. PALEOBIOLOGY 1982;8:4.

[46] Russell FE, Maretic Z. Effects of Latrodectus egg poison on web building. TOXICON 1979;17:649.

[47] Buffkin DC, Russell FE, Deshmukh A. Preliminary studies on the toxicity of black widow spider eggs. TOXICON 1971;9:393.

[48] Xu D, Wang X. Transcriptome Analysis to Understand the Toxicity of Latrodectus tredecimguttatus Eggs. Toxins (Basel) 2016;8.

[49] Akhunov AA, Golubenko Z, Abdurashidova NA, Mustakimova EC, Ibragimov FA, Mackessy S. Comparative Biochemistry of the Physiologically Active Components of Venom, Hemolymph, and Eggs of the Karakurt Spider (Latrodectus tredecimguttatus). CHEM NAT COMPD+ 2001;37:562.
[50] Yan Y, Li J, Zhang Y, Peng X, Guo T, Wang J, Hu W, Duan Z, Wang X. Physiological and biochemical characterization of egg extract of black widow spiders to uncover molecular basis of egg toxicity. BIOL RES 2014;47:17.
[51] Gonzalez SN, Sulzyk V, Weigel MM, Cuasnicu PS. Cysteine-Rich Secretory Proteins (CRISP) are Key Players in Mammalian Fertilization and Fertility. Front Cell Dev Biol 2021;9:800351.
[52] Bolger AM, Lohse M, Usadel B. Trimmomatic: a flexible trimmer for Illumina sequence data. BIOINFORMATICS 2014;30:2114.
[53] Wenger AM, Peluso P, Rowell WJ, Chang PC, Hall RJ, Concepcion GT, Ebler J, Fungtammasan A, Kolesnikov A, Olson ND, Topfer A, Alonge M, Mahmoud M, Qian Y, Chin CS, Phillippy AM, Schatz MC, Myers G, DePristo MA, Ruan J, Marschall T, Sedlazeck FJ, Zook JM, Li H, Koren S, Carroll A, Rank DR, Hunkapiller MW. Accurate circular consensus long-read sequencing improves variant detection and assembly of a human genome. NAT BIOTECHNOL 2019;37:1155.
[54] Lu L, Liu X, Huang W, Giusti-Rodríguez P, Cui J, Zhang S, Xu W, Wen Z, Ma S, Rosen JD, Xu Z, Bartels CF, Kawaguchi R, Hu M, Scacheri PC, Rong Z, Li Y, Sullivan PF, Song H, Ming G, Li Y, Jin F. Robust Hi-C Maps of Enhancer-Promoter Interactions Reveal the Function of Non-coding Genome in Neural Development and Diseases. MOL CELL 2020;79:521.
[55] Rao SS, Huntley MH, Durand NC, Stamenova EK, Bochkov ID, Robinson JT, Sanborn AL, Machol I, Omer AD, Lander ES, Aiden EL. A 3D map of the human genome at kilobase resolution reveals principles of chromatin looping. CELL 2014;159:1665.
[56] Meyer M, Kircher M. Illumina sequencing library preparation for highly multiplexed target capture and sequencing. Cold Spring Harb Protoc 2010;2010:t5448.
[57] Durand NC, Shamim MS, Machol I, Rao SS, Huntley MH, Lander ES, Aiden EL. Juicer Provides a One-Click System for Analyzing Loop-Resolution Hi-C Experiments. CELL SYST 2016;3:95.
[58] Marcais G, Kingsford C. A fast, lock-free approach for efficient parallel counting of occurrences of k-mers. BIOINFORMATICS 2011;27:764.
[59] Cheng H, Concepcion GT, Feng X, Zhang H, Li H. Haplotype-resolved de novo assembly using phased assembly graphs with hifiasm. NAT METHODS 2021;18:170.
[60] Ruan J, Li H. Fast and accurate long-read assembly with wtdbg2. NAT METHODS 2020;17:155.
[61] Hu J, Fan J, Sun Z, Liu S. NextPolish: a fast and efficient genome polishing tool for long-read assembly. BIOINFORMATICS 2020;36:2253.
[62] Li H. Minimap2: pairwise alignment for nucleotide sequences. BIOINFORMATICS 2018;34:3094.
[63] Li H, Handsaker B, Wysoker A, Fennell T, Ruan J, Homer N, Marth G, Abecasis G, Durbin R. The Sequence Alignment/Map format and SAMtools. BIOINFORMATICS 2009;25:2078.
[64] Dudchenko O, Batra SS, Omer AD, Nyquist SK, Hoeger M, Durand NC, Shamim MS, Machol I, Lander ES, Aiden AP, Aiden EL. De novo assembly of the Aedes aegypti genome using Hi-C yields chromosome-length scaffolds. SCIENCE 2017;356:92.
[65] Dudchenko O, Shamim MS, Batra S, Durand NC, Musial NT, Mostofa R, Pham M, St Hilaire BG, Yao W, Stamenova E, Hoeger M, Nyquist SK, Korchina V, Pletch K, Flanagan JP, Tomaszewicz A, Mcaloose D,

Cynthia PRE, Novak BJ, Omer AD, Aiden EL. The Juicebox Assembly Tools module facilitates de novo assembly of mammalian genomes with chromosome-length scaffolds for under $1000. Cold Spring Harbor: Cold Spring Harbor Laboratory Press, 2018.

[66] Flynn JM, Hubley R, Goubert C, Rosen J, Clark AG, Feschotte C, Smit AF. RepeatModeler2 for automated genomic discovery of transposable element families. Proc Natl Acad Sci U S A 2020;117:9451.

[67] Tarailo-Graovac M, Chen N. Using RepeatMasker to identify repetitive elements in genomic sequences. Curr Protoc Bioinformatics 2009;Chapter 4:4.

[68] Bruna T, Hoff KJ, Lomsadze A, Stanke M, Borodovsky M. BRAKER2: automatic eukaryotic genome annotation with GeneMark-EP+ and AUGUSTUS supported by a protein database. NAR Genom Bioinform 2021;3:a108.

[69] Chan PP, Lin BY, Mak AJ, Lowe TM. tRNAscan-SE 2.0: improved detection and functional classification of transfer RNA genes. NUCLEIC ACIDS RES 2021;49:9077.

[70] Kalvari I, Nawrocki EP, Ontiveros-Palacios N, Argasinska J, Lamkiewicz K, Marz M, Griffiths-Jones S, Toffano-Nioche C, Gautheret D, Weinberg Z, Rivas E, Eddy SR, Finn RD, Bateman A, Petrov AI. Rfam 14: expanded coverage of metagenomic, viral and microRNA families. NUCLEIC ACIDS RES 2021;49:D192.

[71] Nawrocki EP, Eddy SR. Infernal 1.1: 100-fold faster RNA homology searches. BIOINFORMATICS 2013;29:2933.

[72] Emms DM, Kelly S. OrthoFinder: phylogenetic orthology inference for comparative genomics. GENOME BIOL 2019;20:238.

[73] Katoh K, Standley DM. MAFFT multiple sequence alignment software version 7: improvements in performance and usability. MOL BIOL EVOL 2013;30:772.

[74] Camacho C, Coulouris G, Avagyan V, Ma N, Papadopoulos J, Bealer K, Madden TL. BLAST+: architecture and applications. BMC BIOINFORMATICS 2009;10:421.

[75] Price MN, Dehal PS, Arkin AP. FastTree 2--approximately maximum-likelihood trees for large alignments. PLOS ONE 2010;5:e9490.

[76] Tang H, Bowers JE, Wang X, Ming R, Alam M, Paterson AH. Synteny and collinearity in plant genomes. SCIENCE 2008;320:486.

[77] Yang Z. PAML 4: phylogenetic analysis by maximum likelihood. MOL BIOL EVOL 2007;24:1586.

[78] De Bie T, Cristianini N, Demuth JP, Hahn MW. CAFE: a computational tool for the study of gene family evolution. BIOINFORMATICS 2006;22:1269.

[79] Pineda SS, Chaumeil PA, Kunert A, Kaas Q, Thang M, Le L, Nuhn M, Herzig V, Saez NJ, Cristofori-Armstrong B, Anangi R, Senff S, Gorse D, King GF. ArachnoServer 3.0: an online resource for automated discovery, analysis and annotation of spider toxins. BIOINFORMATICS 2018;34:1074.

[80] Harris RS. IMPROVED PAIRWISE ALIGNMENT OF GENOMIC DNA., vol. Doctor of Philosophy: The Pennsylvania State University, 2007.

[81] Kent WJ, Baertsch R, Hinrichs A, Miller W, Haussler D. Evolution's cauldron: duplication, deletion, and rearrangement in the mouse and human genomes. Proc Natl Acad Sci U S A 2003;100:11484.

[82] Blanchette M, Kent WJ, Riemer C, Elnitski L, Smit AF, Roskin KM, Baertsch R, Rosenbloom K, Clawson H, Green ED, Haussler D, Miller W. Aligning multiple genomic sequences with the threaded blockset aligner. GENOME RES 2004;14:708.

[83] Hubisz MJ, Pollard KS, Siepel A. PHAST and RPHAST: phylogenetic analysis with space/time models. BRIEF BIOINFORM 2011;12:41.

[84] Siepel A, Bejerano G, Pedersen JS, Hinrichs AS, Hou M, Rosenbloom K, Clawson H, Spieth J, Hillier LW, Richards S, Weinstock GM, Wilson RK, Gibbs RA, Kent WJ, Miller W, Haussler D. Evolutionarily conserved elements in vertebrate, insect, worm, and yeast genomes. GENOME RES 2005;15:1034.

[85] Yang H, Wang K. Genomic variant annotation and prioritization with ANNOVAR and wANNOVAR. NAT PROTOC 2015;10:1556.

[86] Wang K, Li M, Hakonarson H. ANNOVAR: functional annotation of genetic variants from high-throughput sequencing data. NUCLEIC ACIDS RES 2010;38:e164.

[87] Kosakovsky PS, Poon A, Velazquez R, Weaver S, Hepler NL, Murrell B, Shank SD, Magalis BR, Bouvier D, Nekrutenko A, Wisotsky S, Spielman SJ, Frost S, Muse SV. HyPhy 2.5-A Customizable Platform for Evolutionary Hypothesis Testing Using Phylogenies. MOL BIOL EVOL 2020;37:295.

[88] Roberts SP, Harrison JF, Dudley R. Allometry of kinematics and energetics in carpenter bees (Xylocopa varipuncta) hovering in variable-density gases. J EXP BIOL 2004;207:993.

Figure 1

Click here to access/download;Figure;Fig.1.jpg

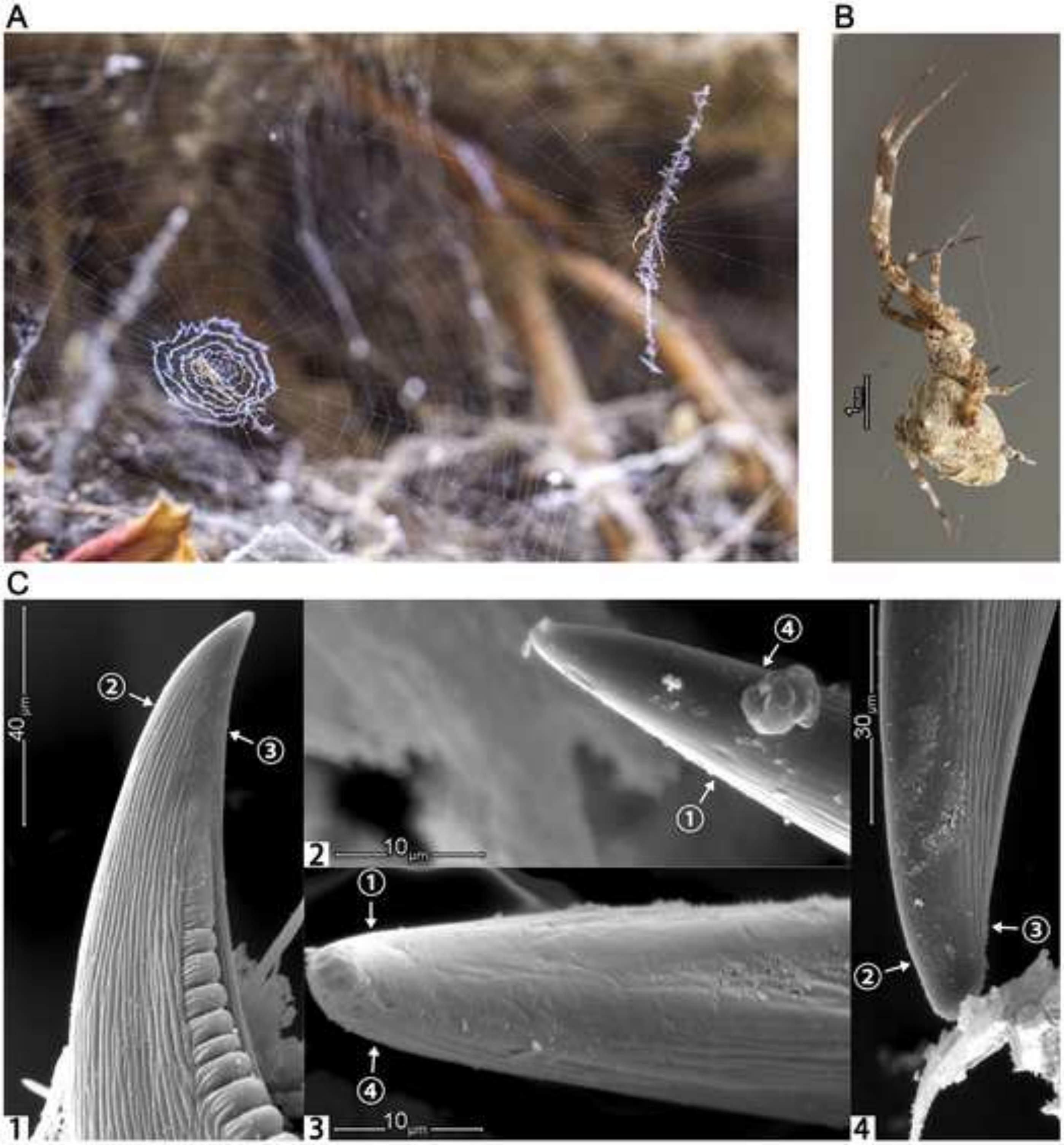

Figure 2

Click here to access/download;Figure;Fig.2.jpg

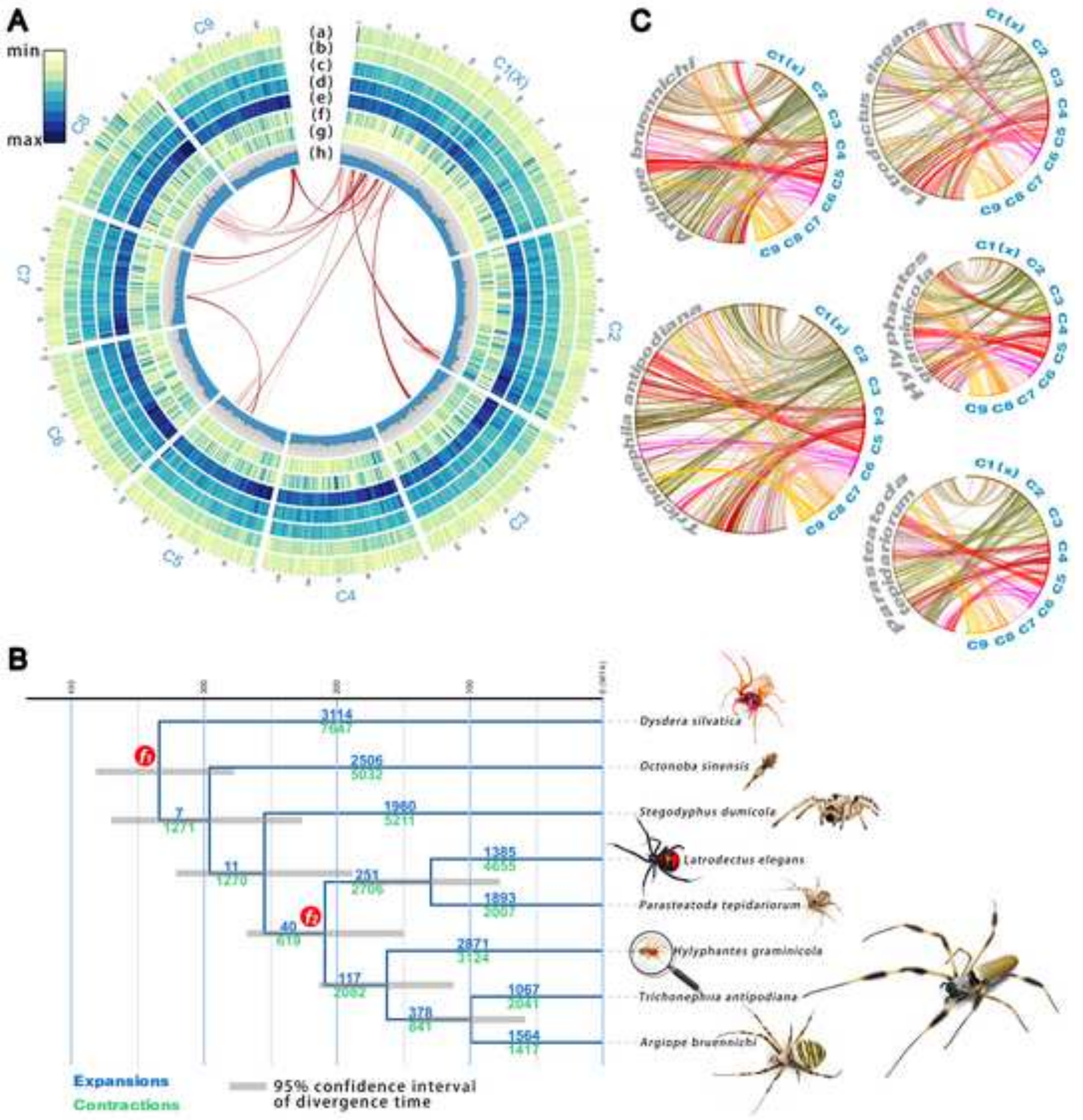

Figure 3 Click here to access/download;Figure;Fig.3.jpg

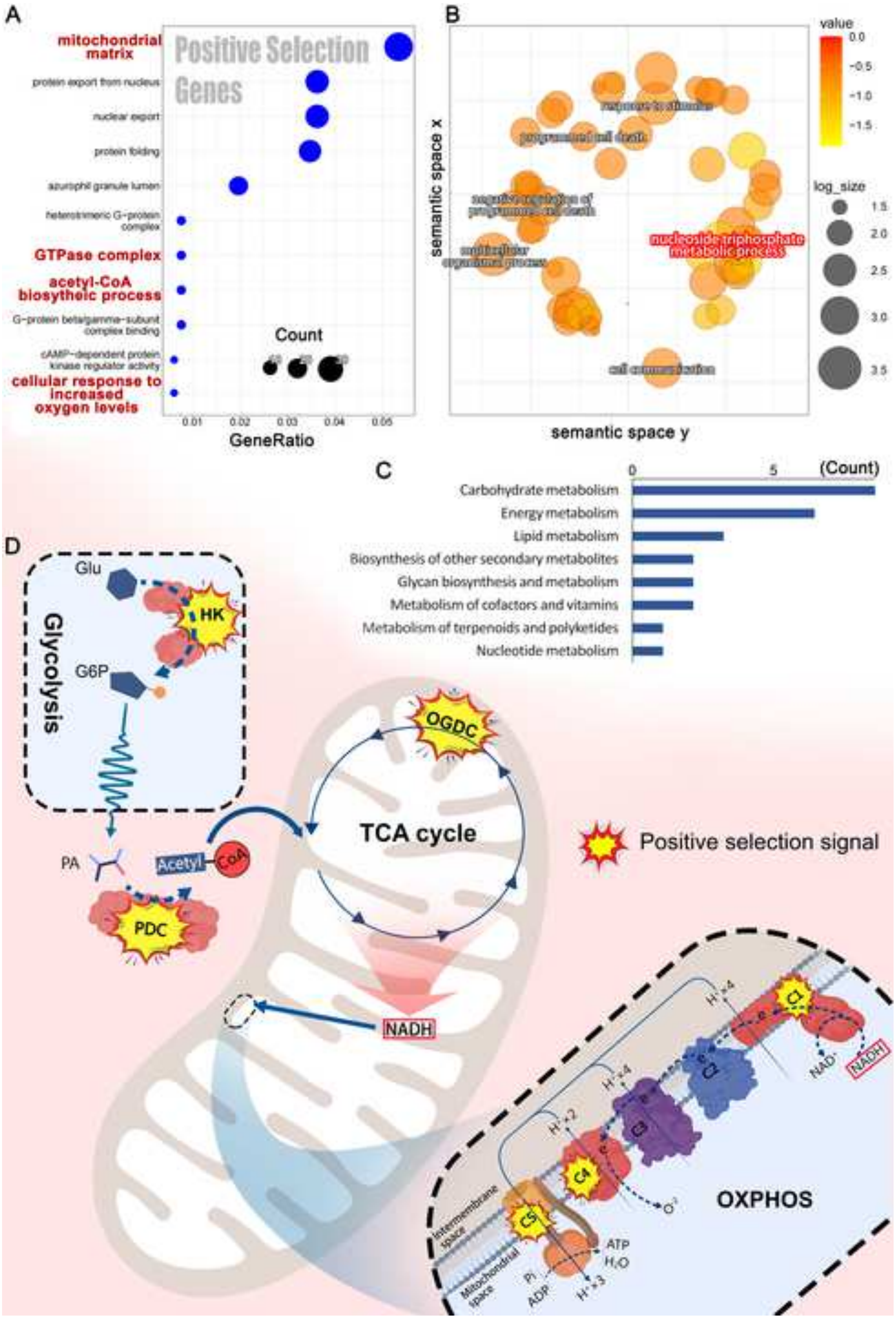

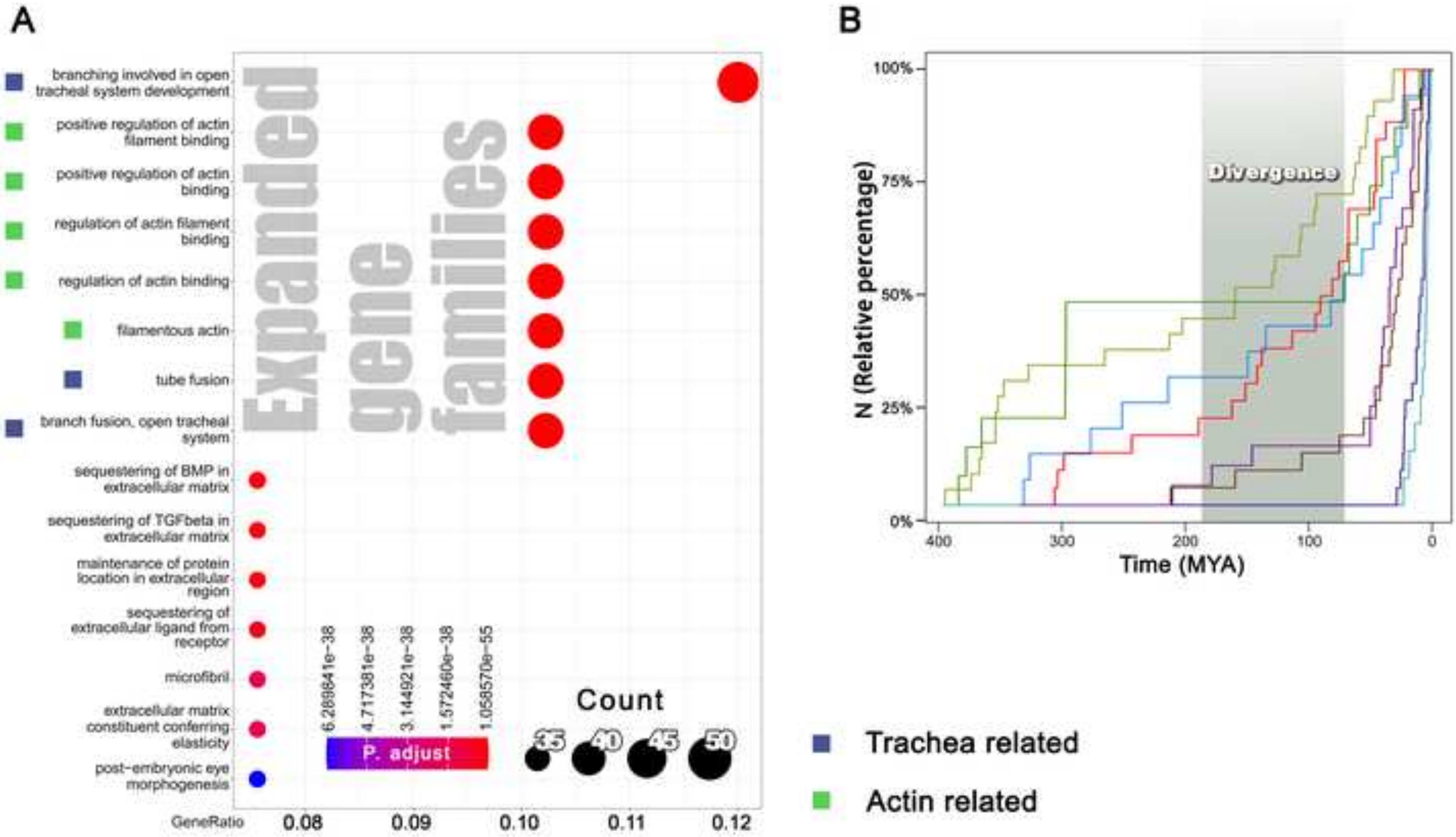
A
branching involved in open tracheal system development
positive regulation of actin filament binding
positive regulation of actin binding
regulation of actin filament binding
regulation of actin binding
filamentous actin
tube fusion
branch fusion, open tracheal system
sequestering of BMP in extracellular matrix
sequestering of TGFbeta in extracellular matrix
maintenance of protein location in extracellular region
sequestering of extracellular ligand from receptor
microfibril
extracellular matrix constituent conferring elasticity
post-embryonic eye morphogenesis
Expanded gene families
6.288841e-38
4.717381e-38
3.144921e-38
1.572460e-38
1.058570e-55
P. adjust
Count
35
40
45
50
GeneRatio
0.08
0.09
0.10
0.11
0.12
B
100%
75%
50%
25%
0%
N (Relative percentage)
Divergence
400
300
200
100
0
Time (MYA)
Trachea related
Actin related

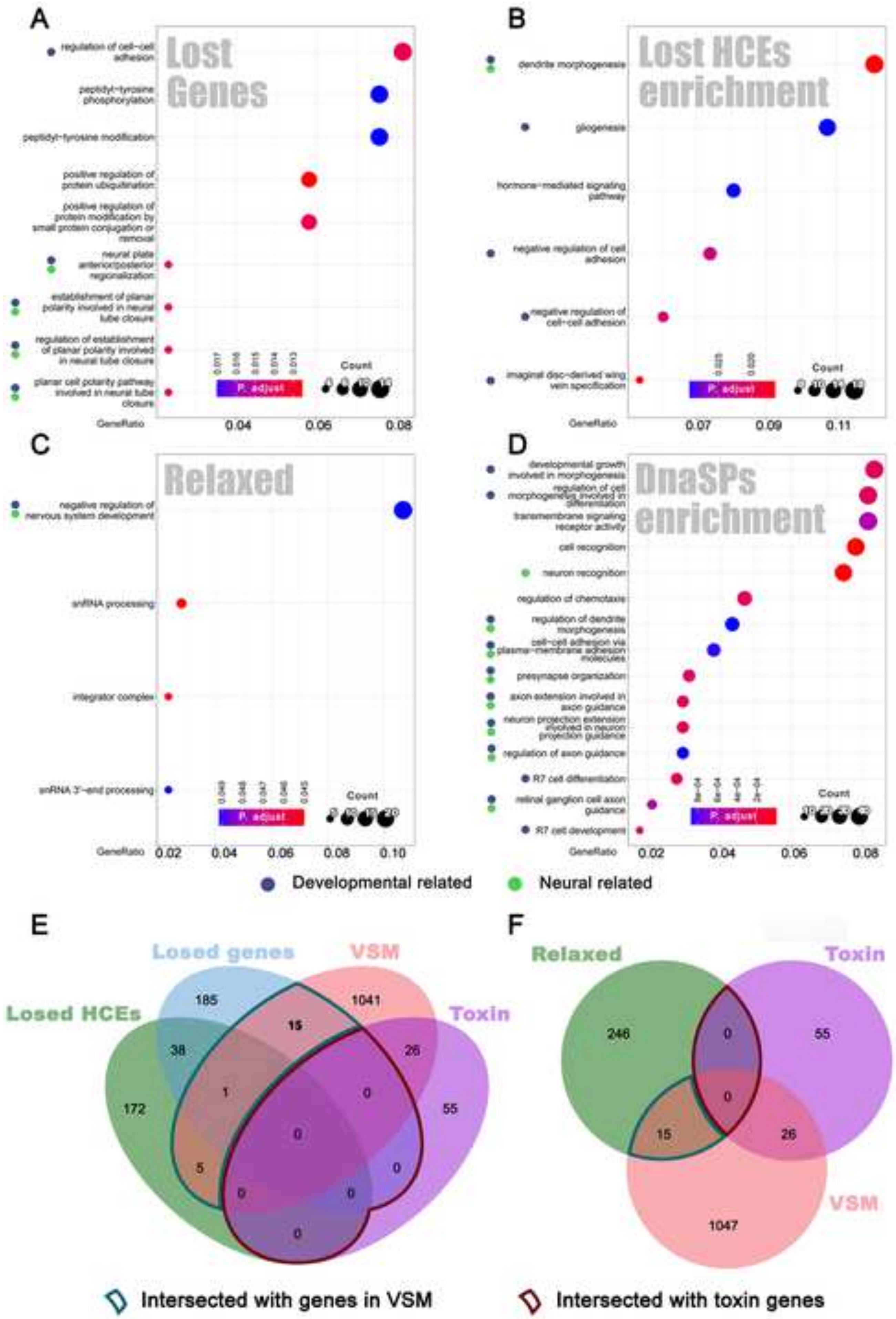
A
Lost Genes
regulation of cell−cell adhesion
peptidyl−tyrosine phosphorylation
peptidyl−tyrosine modification
positive regulation of protein ubiquitination
positive regulation of protein modification by small protein conjugation or removal
neural plate anterior/posterior regionalization
establishment of planar polarity involved in neural tube closure
regulation of establishment of planar polarity involved in neural tube closure
planar cell polarity pathway involved in neural tube closure
p. adjust
Count
GeneRatio
0.04
0.06
0.08
B
Lost HCEs enrichment
dendrite morphogenesis
gliogenesis
hormone−mediated signaling pathway
negative regulation of cell adhesion
negative regulation of cell−cell adhesion
imaginal disc−derived wing vein specification
p. adjust
Count
GeneRatio
0.07
0.09
0.11
C
Relaxed
negative regulation of nervous system development
snRNA processing
integrator complex
snRNA 3'−end processing
p. adjust
Count
GeneRatio
0.02
0.04
0.06
0.08
0.10
D
DnaSPs enrichment
developmental growth involved in morphogenesis
regulation of cell morphogenesis involved in differentiation
transmembrane signaling receptor activity
cell recognition
neuron recognition
regulation of chemotaxis
regulation of dendrite morphogenesis
cell−cell adhesion via plasma−membrane adhesion molecules
presynapse organization
axon extension involved in axon guidance
neuron projection extension involved in neuron projection guidance
regulation of axon guidance
R7 cell differentiation
retinal ganglion cell axon guidance
R7 cell development
p. adjust
Count
GeneRatio
0.02
0.04
0.06
0.08
Developmental related
Neural related
E
Losed genes
VSM
Losed HCEs
Toxin
185
1041
15
38
26
172
1
0
55
0
5
0
0
0
0
Intersected with genes in VSM
F
Relaxed
Toxin
246
0
55
0
15
26
VSM
1047
Intersected with toxin genes

Figure 6

Click here to access/download;Figure;Fig.6.jpg

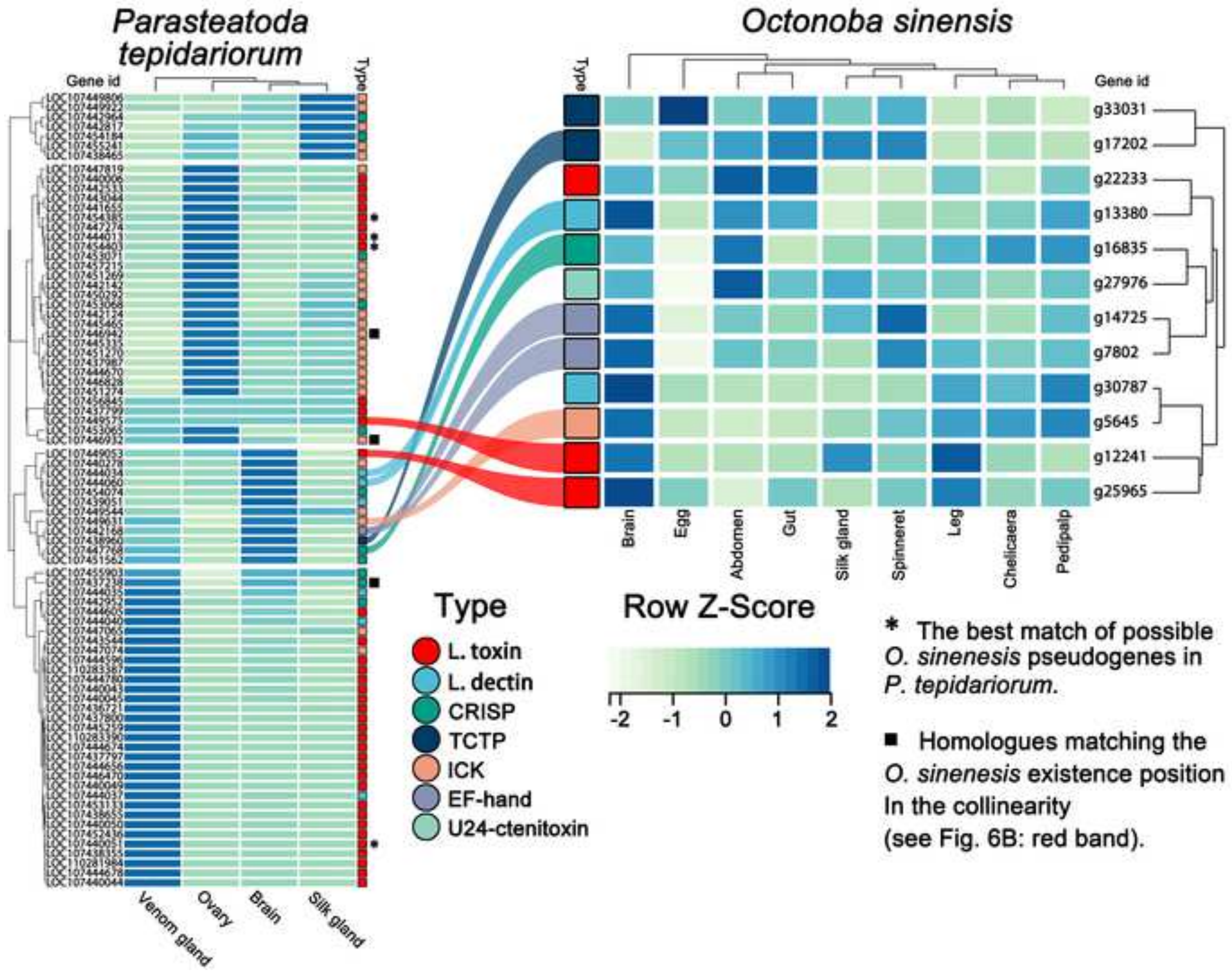

Figure 7

Click here to access/download;Figure;Fig.7.jpg

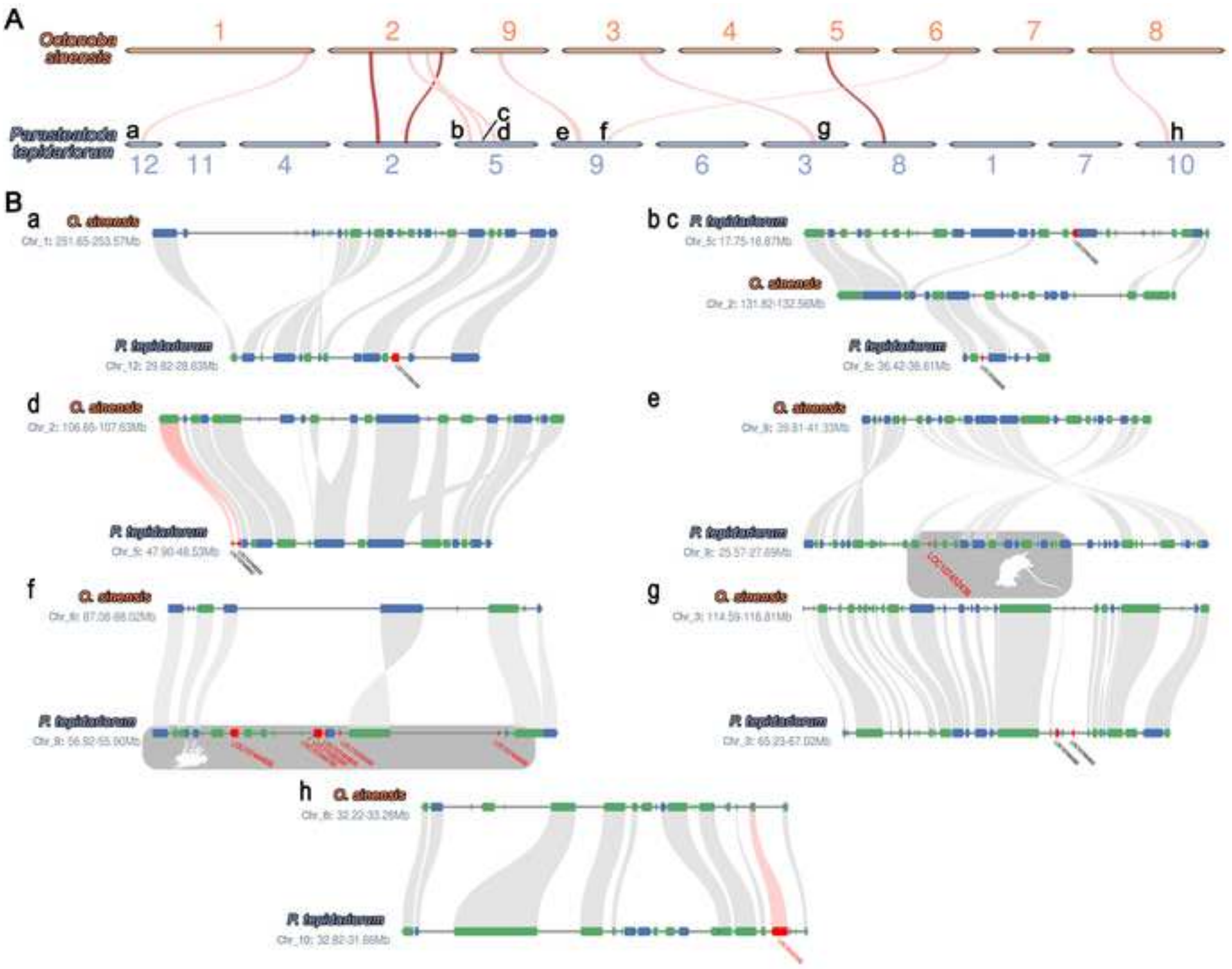

Additional file 1

Click here to access/download
Supplementary Material
Additional file 1.mp4

Additional file 2

Click here to access/download
Supplementary Material
Additional file 2.mp4

Additional file 3

Click here to access/download
Supplementary Material
Additional file 3.mp4

Additional file 4

Click here to access/download
Supplementary Material
Additional file 4-new.xlsx

Additional file 5

Click here to access/download
Supplementary Material
Additional file 5.docx

Additional file 6

Click here to access/download
Supplementary Material
Additional file 6.xls

Additional file 7

Click here to access/download
Supplementary Material
Additional file 7.fa

Additional file 8

Click here to access/download
Supplementary Material
Main analysis process script

Click here to access/download;Personal Cover;Cover letter lfy.doc

中國科学院動物研究所
INSTITUTE OF ZOOLOGY,CHINESE ACADEMY OF SCIENCES

August 31th, 2023

Scott Edmunds, Ph.D.
Editors-in-Chief, GigaScience
GigaScience Press

Dear Editors:

We are pleased to submit our manuscript entitled "A Trade-off in Evolution: The Adaptive Landscape of Spiders without Venom Glands" for consideration in *GigaScience*.

The evolution of traits in a trade-off fashion propounded by Charles Darwin in The Origin of Species is a very important subject. However, our understanding of trade-offs in evolutionary biology remains poor. Uloboridae is one of the venom gland deficient of spiders. Trade-offs between lack of venom glands and a laborious prey wrapping technique in *Octonoba sinensis* (Araneae, Uloboridae) provide us a classic example to understand the trade-off strategy.

Genome sequences from *O. sinensis* reveal that the evolution of energy metabolism pathways is the key factor in their sustained power output. Comparative genomics and transcriptomics suggest that the absence of regions and regions under relaxed selection in *O. sinensis* are concentrated in the field of development, and the number of toxin genes expressed in *O. sinensis* are less than in most spider species.

We combined multi-omics and functional approaches to uncover the evolution of adaptation strategies in the Uloboridae and provide insights into the mechanism underlying this trade-off between different predation strategies. Our study shows that building the organisms is the inter-dependence between sets of traits when one part modified through natural selection, other parts become varied.

Neither the manuscript nor any part of it has been published or is under consideration for review or publication elsewhere. All authors have directly participated in the planning, execution, or analysis of this study, and have read and approved the final version submitted. There is no conflict of interest related to the manuscript.

We believe that this work is well-suited for the broad readership of *GigaScience*. Thank you for receiving our manuscript and considering it for review. We greatly appreciate your time and look forward to your response.

Yours sincerely,

Shuqiang Li, Ph.D.
Professor, Key Laboratory of Zoological Systematics and Evolution
Institute of Zoology, Chinese Academy of Sciences
Beijing 100101, China
