# Supplementary material for: A trade-off in evolution: the adaptive landscape of spiders without venom glands
Source: Gigascience. 2024 Aug 5;13:giae048. doi: 10.1093/gigascience/giae048 (PMC11299198; doi:10.1093/gigascience/giae048)
Supplement: giae048_GIGA-D-23-00275_R3 [file giae048_giga-d-23-00275_r3.pdf]

GigaScience

# A Trade-off in Evolution: The Adaptive Landscape of Spiders without Venom Glands

--Manuscript Draft--

| | |
|---|---|
| **Manuscript Number:** | GIGA-D-23-00275R3 |
| **Full Title:** | A Trade-off in Evolution: The Adaptive Landscape of Spiders without Venom Glands |
| **Article Type:** | Research |
| **Funding Information:** | Strategic Priority Research Program of Chinese Academy of Sciences (XDB31000000) — professor Shuqiang Li<br>the Program of National Natural Sciences Foundation of China (NSFC–32170447) — Dr Zhe Zhao<br>the Program of National Natural Sciences Foundation of China (NSFC–32370490) — Dr Zhe Zhao |
| **Abstract:** | Background: Venom glands play a key role in the predation and defense strategies of almost all spider groups. However, the spider family Uloboridae lacks venom glands and has evolved an adaptive strategy: They excessively wrap their prey directly with spider silk instead of paralyzing it first with toxins. This shift of survival strategy is very fascinating, but the genetic underpinnings behind it are poorly understood.<br>Results: Spanning multiple spider groups, we conducted multi-omics analyses on Octonoba sinensis, and described the adaptive evolution of the Uloboridae family at the genome level. We observed the coding genes of myosin and twitchin in muscles are under positive selection, energy metabolism functions are enhanced, and gene families related to tracheal development and tissue mechanical strength are expanded or emerged, all of which are related to the unique anatomical structure and predatory behavior of spiders in the family Uloboridae. In addition, we also scanned the elements which are absent or under relaxed purifying selection, as well as toxin gene homologs in the genomes of two species in this family. The results show that the absence of regions and regions under relaxed selection in these spiders' genomes are concentrated in areas related to development and neuro-system. The search for toxin homologs also confirms that there are no toxin coding genes available for hunting in the genome of this group.<br>Conclusions: This study demonstrates the trade-off between different predation strategies in spiders, either using venom or physical energy and provides insights into the possible mechanism underlying this trade-off. Venomless spiders need to mobilize multiple developmental and metabolic pathways related to motor function and limb mechanical strength to cover the decline in adaptability caused by the absence of venom glands. |
| **Corresponding Author:** | Shuqiang Li, Ph.D.<br>Institute of Zoology Chinese Academy of Sciences<br>Beijing, CHINA |
| **Corresponding Author Secondary Information:** | |
| **Corresponding Author's Institution:** | Institute of Zoology Chinese Academy of Sciences |
| **Corresponding Author's Secondary Institution:** | |
| **First Author:** | Yiming Zhang |
| **First Author Secondary Information:** | |
| **Order of Authors:** | Yiming Zhang<br>Yunxiao Shen<br>Pengyu Jin, Ph.D.<br>Bingyue Zhu, Ph.D. |

Powered by Editorial Manager® and ProduXion Manager® from Aries Systems Corporation

| | |
|---|---|
| | Yejie Lin |
| | Tongyao Jiang |
| | Xianting Huang |
| | Yang Wang |
| | Zhe Zhao, Ph.D. |
| | Shuqiang Li, Ph.D. |
| **Order of Authors Secondary Information:** | |

**Response to Reviewers:**

Response to reviewers
Dear Editor Zhang

I hope this email finds you well. We have carefully considered all the feedback and have made the necessary revisions to our paper.
We would like to emphasize that the entire review process has been an incredibly enriching experience for us. The insights and suggestions from the reviewers and editor have not only strengthened our research but have also broadened our understanding of the field. We are grateful for the time and effort the reviewers have invested in providing us with such valuable feedback.
Thank you.
Best regards,

Yiming Zhang; Shuqiang Li
Institute of Zoology, Chinese Academy of Sciences
1. Beichen West Road, Chaoyang District
Beijing 100101, P. R. China
Tel: +86-13363636950
Fax: +86-10-64807216
Email: zhangyiming@ioz.ac.cn; lisq@ioz.ac.cn

Editor comments
Please include a point-by-point within the 'Response to Reviewers' box in the submission system. Please ensure you describe additional experiments that were carried out and include a detailed rebuttal of any criticisms or requested revisions that you disagreed with. Please remember to make revisions per Dr. Sandra Correa-Garhwal's comments 34-48 in the second round of review.
>>>Response: Thank you for giving us the opportunity to supplement these responses. We have responded to each point individually.
34.Figure S4: The black text in the figure should be added the figure legend. For the green genes, add the species they come from.
>>>Response: Thank you for your comments. We have moved the black text from the figure to the figure legend and listed the species sources of the reference toxin genes (marked with green color).
“Figure S4: Phylogenetic tree of neurotoxin genes (Latrotoxin). Tips labelled in red font represent Octonoba sinensis genes, those labelled in green font represent the reference sequences of different Latrotoxins genes previously studied, others represent Parasteatoda tepidariorum genes. Tips labelled in green font come from different species, Latrodectus tredecimguttatus: sp|Q25338|LITD_LATTR, sp|P23631|LATA_LATTR, sp|Q02989|LITA_LATTR, sp|Q9XZC0|LCTA_LATTR; Steatoda grossa: sp|L7X8P2|LATA_STEGR; Latrodectus geometricus: sp|L7XCU0|LATA_LATGE; Latrodectus hesperus: sp|P0DJE3|LATA_LATHE; Latrodectus pallidus: sp|L7XDS4|LATA_LATPL; Latrodectus hasselti: sp|G0LXV8|LATA_LATHA. The red font with black stroke is a similar object below the recognition threshold as an outlier.”
35.Lines 281-290: I think is very interesting that there is conservation in the where these genes are in both species regardless of function. The paragraph will benefit from editing because the results are not coming thru. I also suggest that the authors describe all genes that fall under this category, I think that there are only three by looking at Figure 6. Given the small number of instances, it seems odd to only describe one of them. These results should also be added to the discussion: we found these genes, they are expressed (or not) in XX tissue, and we think the function in octonoba

is XX.
>>>Response: It is true that there are three pairs of toxin homologs preserved in the syntenic region, but only one of them was highly expressed in the venom glands of P. tepidariorum, which we consider a relatively reliable toxin gene in P. tepidariorum. The others showed their highest expression in other tissues of P. tepidariorum (Figure 6) and may be performing non-toxic functions, so we didn't have a detailed discussion on the two genes mentioned later.
In the revised manuscript, we mentioned the above content and added some discussion at this paragraph.
“By searching for toxin gene homologs in collinearity fragments of O. sinensis and P. tepidariorum, a particular class of genes was found in O. sinensis. These genes are located in the same place as the P. tepidariorum toxin gene homologs in the collinearity segment, but they can no longer be identified as toxin genes (below the minimum recognition threshold, see methods) (Figure 7B, red ribbon). There are three pairs of such genes, includes one pair of CRISP genes (g31478~LOC107437238) and two pairs of ICK genes (g6736~LOC107446942 and g6736~LOC107446932). Their expression patterns in P. tepidariorum indicate that only the CRISP gene is a reliable toxin gene (Figure 6, black squares). In O. sinensis, this CRISP gene (g31478) cannot be unambiguously classified as a homolog of toxin genes due to changes in protein structure (Additional file 4: Table S16). However, compared with other toxin genes which it is difficult to find pseudogenes, the ortholog of this CRISP gene in O. sinensis have complete gene structures, and can be expressed in multiple tissues of a venomless spider (Additional file 4: Table S17). These all indicate that this CRISP gene (g31478) must play the role of a non-toxic gene. We believe that this observation suggests a potential functional shift between toxic and non-toxic genes in spiders.” (see lines 292–305)
We also mentioned relevant content in the Discussion section of the article.
“Multi-omics analysis revealed the absence or possible functional shift of toxin genes in O. sinensis.” (see lines 330–331)
“Simultaneously, through analyzing expression patterns and synteny relationships, it was discovered that toxin genes in venomous spiders are expressed in other tissues of uloborids and may perform non-toxic functions. This observation suggests a potential functional shift between toxic and non-toxic genes in spiders. In addition to toxin genes, the absence of certain genes in uloborids raises concerns. These include protein c-ets-2, CC2D, and …” (see lines 357–361)
36.Figure 6 The legend that is inside the figure, starting with the asterisk should be included in the legend and maybe not in the figure. It also refers to itself which is odd)
>>>Response: Thank you for your comments. We have moved the corresponding information from the figure to the figure legend.
37.Figure 7. What the red line in A means needs to be included in the legend for panel A. Also, include that the different colors (green vs. blue) in the genes for panel B indicate.
>>>Response: Thank you for your comments. The “red line” indicates that the original toxin homolog has been retained in the collinearity. The genes with different colors in the Figure 7B represent different transcription directions. We have added relevant information in the figure legend.
38.Line 300: add () to A
>>>Response: Thank you, we have made the correction.
39.Line 301: add () to A
>>>Response: Thank you, we have made the correction.
40.For the methods, include the type of camera that was used to film the pre-wrapping behavior.
>>>Response: Thank you for your comments. We have supplemented this information. The text is taken as follows:
“We recorded a series of videos to observe the predation behavior, using Logitech StreamCam 960-001282.” (lines 386–387)
41.Line 393 Additional file 6 has no information related to tissue samples
>>>Response: Thank you for your comments. The tissue sample information involved in Additional file 6 is in the Section 5 of Methods (see lines 428–431). The purpose of Additional file 6 is to demonstrate two genes (Figure 7B, genes linked with red ribbon) in the O. sinensis have indeed been expressed. We have removed other genes in the new modification, leaving only the two required genes. We have renamed the table as Table S17 and placed it in Additional file 4.
42.Line 400: replace "have been" with were

Powered by Editorial Manager® and ProduXion Manager® from Aries Systems Corporation

>>>Response: Thank you, we have made the correction.
43.Line 406 spell out 9. Add "of" after Gb
>>>Response: Thank you, we have made the correction.
44.Section 7 - was this methodology used for figure s4? How was that tree generated?
>>>Response: Thank you, the phylogenetic tree of latrotoxin homologs (Figure S4) was reconstructed using the neighbor-joining method, following alignment of the full-length protein sequences via Mafft. We have supplemented this information in this Section.
45.How were the cluster analyses done?
>>>Response: Thank you for your comments. One-to-one ortholog identification among ten species (Figure 2B) was performed using the RBH method by blastp. O. sinensis was used as a reference species. Finally, 5,848 RBH clusters (Additional file 4: Table S19) were retained for analysis. We have supplemented this information. (lines 484–486)
46.Line 456 spell out HCEs
>>>Response: Thank you, we have made the correction. (see line 502)
47.Line 468: instead of "previous databases" explain what those are. Downloaded sequences from multiple sources (NCBI/Arachnoserver?) that includes multiple species? Or includes venom components from all spiders and other animals?
>>>Response: Thank you, we have supplemented this information. This information comes from previous studies, and we have listed the articles published in these studies as references and added a brief description in the manuscript.
"Based on ArachnoServer 3.0 [89], a specialized spider venom database, and integrating toxin protein sequences obtained from other toxin research of spiders [37, 38, 90], we have compiled a new reference dataset." (see lines 514–516)
48.Availability of data: the NCBI code given is not functional and there is an empty parenthesis for the gigaDB Digital Repository.
>>>Response: Thanks for your suggestion, the relevant data has been uploaded to the FTP server provided by GigaDB and will be made public after the article is published. At that time, there will be a valid link filled in the current parentheses. At this stage, you can obtain the corresponding information through the FTP link.
We have provided a new available BioProject Accession number (PRJNA1018860)
In addition, we have added a public location for sequencing data in SRR, which is ScienceDB (doi.org/10.57760/sciencedb.09166).

Reviewer #2:
I believe the manuscript has shown improvement since the last revision and has effectively addressed most of my concerns and comments. However, I think the discussion section still requires further refinement to convey the main message clearly. For instance, consolidating the concluding remarks into the final paragraph and keeping all conclusions within that paragraph would enhance coherence.
>>>Response: I would like to express my sincere gratitude for your assistance and support throughout the entire process. We have learned a great deal from your feedback and suggestions. Thank you for your valuable contributions.
We have consolidated all concluding remarks into the last paragraph of the discussion.
"For predators, there exists a pervasive evolutionary trade-off between chemical and physical attack strategies. Previous research has frequently favored the exploration of chemical strategies, particularly venom. However, against the backdrop of nearly all spiders being toxic predators, our study delves into the genetic basis underlying the alternative choice in this trade-off. Unsurprisingly, reliable toxin gene was not identified in Uloboridae, but these adaptive evolutions ranging from muscle to aerobic respiration and then to supply of energy substances, provide strong support for the exceptional physical endurance demands of this group, and compensate for their decreased adaptability due to the absence of venom glands. Furthermore, some development-related gene and element deletions were observed in uloborids. Although the association between these deletions and the absence of spider venom glands remains unclear, they still hold potential for exploring the evolutionary mechanisms underlying this phenomenon."
(see lines 366–376)
Please ensure that numbers under ten are spelled out. Additionally, after "protocol" in line 520, add a period. Include the number for the GigaDB Digital Repository. Furthermore, review the references for format consistency. For example, Reference 50 has the year in bold instead of the issue, and Reference 79 is written in all caps.

Powered by Editorial Manager® and ProduXion Manager® from Aries Systems Corporation

| | |
|---|---|
| | >>>Response: Thank you for your comments. We searched the entire text and spelled out the numbers under ten in the manuscript. Except for some special positions, such as such as "k > 1" in line 496<br>There is a period missing at the end of the main text (after "protocol"). Thank you for your reminder. We have made the necessary changes here.<br>For the access address of GigaDB, currently there is only an FTP server connection method available for reviewers to access. I have communicated with the GigaDB office and they said that the corresponding link will be provided when the article is published.<br>We have thoroughly organized the references. The reference 50 you mentioned has the same volume number as the publication year, so the reference management APP automatically generate it in bold (see reference 51). Reference 79, we have made the correction. (see reference 82). |
| **Additional Information:** | |
| **Question** | **Response** |
| Are you submitting this manuscript to a special series or article collection? | No |
| **Experimental design and statistics**<br><br>Full details of the experimental design and statistical methods used should be given in the Methods section, as detailed in our Minimum Standards Reporting Checklist. Information essential to interpreting the data presented should be made available in the figure legends.<br><br>Have you included all the information requested in your manuscript? | Yes |
| **Resources**<br><br>A description of all resources used, including antibodies, cell lines, animals and software tools, with enough information to allow them to be uniquely identified, should be included in the Methods section. Authors are strongly encouraged to cite Research Resource Identifiers (RRIDs) for antibodies, model organisms and tools, where possible.<br><br>Have you included the information requested as detailed in our Minimum Standards Reporting Checklist? | Yes |
| **Availability of data and materials** | Yes |

Powered by Editorial Manager® and ProduXion Manager® from Aries Systems Corporation

| | |
|---|---|
| All datasets and code on which the conclusions of the paper rely must be either included in your submission or deposited in publicly available repositories (where available and ethically appropriate), referencing such data using a unique identifier in the references and in the “Availability of Data and Materials” section of your manuscript.<br><br>Have you have met the above requirement as detailed in our Minimum Standards Reporting Checklist? | |

Manuscript Click here to access/download;Manuscript;giga-June26.docx

# A Trade-off in Evolution: The Adaptive Landscape of Spiders without Venom Glands

Yiming Zhang†[1,2,3], Yunxiao Shen†[1,3], Pengyu Jin[1], Bingyue Zhu[1,3], Yejie Lin[1,2], Tongyao Jiang[1,3], Xianting Huang[1,3], Yang Wang[1,3], Zhe Zhao[1] and Shuqiang Li[1]*

[1]Key Laboratory of Zoological Systematics and Evolution, Institute of Zoology, Chinese Academy of Sciences, Beijing 100101, China

[2]Hebei Key Laboratory of Animal Diversity, College of Life Sciences, Langfang Normal University, Langfang 065000, China

[3]University of Chinese Academy of Sciences, Beijing 101408, China

† Yiming Zhang and Yunxiao Shen contributed equally to this work.

*Corresponding author: E-mail: lisq@ioz.ac.cn

Yiming Zhang [0000-0001-8547-6654]; Yunxiao Shen [0009-0003-9186-5068]; Bingyue Zhu [0000-0001-9875-3286]; Pengyu Jin [0000-0003-1310-2711]; Yejie Lin [0000-0002-6789-2731]; Yang Wang [0009-0007-6882-1066]; Tongyao Jiang [0000-0002-6148-1157]; Shuqiang Li [0000-0002-3290-5416]

**Abstract**

**Background:** Venom glands play a key role in the predation and defense strategies of almost all spider groups. However, the spider family Uloboridae lacks venom glands and has evolved an adaptive strategy: They excessively wrap their prey directly with spider silk instead of paralyzing it first with toxins. This shift in survival strategy is very fascinating, but the genetic underpinnings behind it are poorly understood.

**Results:** Spanning multiple spider groups, we conducted multi-omics analyses on *Octonoba sinensis*, and described the adaptive evolution of the Uloboridae family at the genome level. We observed the coding genes of *myosin* and *twitchin* in muscles are under positive selection, energy metabolism functions are enhanced, and gene families related to tracheal development and tissue mechanical strength are expanded or emerged, all of which are related to the unique anatomical structure and predatory behavior of spiders in the family Uloboridae. In addition, we also scanned the elements that are absent or under relaxed purifying selection, as well as toxin gene homologs in the genomes of two

species in this family. The results show that the absence of regions and regions under relaxed selection in these spiders' genomes are concentrated in areas related to development and neuro-system. The search for toxin homologs revealed possible gene function shift between toxins and non-toxins, and confirms that there are no reliable toxin genes in the genome of this group.

**Conclusions:** This study demonstrates the trade-off between different predation strategies in spiders, either using chemical or physical strategy, and provides insights into the possible mechanism underlying this trade-off. Venomless spiders need to mobilize multiple developmental and metabolic pathways related to motor function and limb mechanical strength to cover the decline in adaptability caused by the absence of venom glands.

**Keywords:** Venom gland deficient, Adaptive evolution, Genome, *Octonoba sinensis*

## Introduction

"Venomous" is a common way that people perceive spiders (Araneae). The toxic, painful, and even fatal bite is always frightening. Indeed, almost all spiders are venomous. In the earliest diverging suborder Mesothelae, fangs that could inject venom are already present [1]. Some highly toxic species make spiders even more notorious. As an important means of hunting and defense, the toxin system gives spiders an outstanding advantage in environmental suitability and has allowed them to spread throughout the world. Of course, there are always exceptions, and some outliers are believed to be lacking venom glands. Currently, known spiders without venom grands include *Holarchaea* (2 species)[2–4] and the entire family Uloboridae, with the latter being the most prosperous group [5]. Compared to *Holarchaea*, which has a smaller body size, fewer species, and limited distribution, the family Uloboridae provides a satisfactory model for us to study the evolution of an important synapomorphy of spiders and the adaptation strategy brought about by the loss of important functional traits.

Due to the absence of venom glands, the predation methods of uloborids are also relatively specialized and excessive. Generally speaking, using venom to paralyze prey is an effective chemical attack strategy.

However, many kinds of spiders integrate both chemical attack and physical attack strategies. Certain species within the Araneoidea family utilize entanglement initially to restrain larger prey before a venomous final strike [6]. Although this predatory tactic may alleviate the selective pressure associated with venom usage, entanglement appears rudimentary in comparison to the prey-wrapping behavior exhibited by uloborids. In Uloboridae, this physical attack as the sole means of attack can span from a few minutes to nearly an hour, with the spider silk utilized sometimes exceeding a hundred meters in length [7–9]. Therefore, considerable physical endurance is indispensable for the successful execution of this predatory tactic. Previous anatomical records indicate uloborids have well-developed trachea [10], and many branches of the trachea extend into the prosoma and appendages [11]. however, there is no relevant research that can link these adaptive characteristics to the venom gland deficiency of this group.

The spider *Octonoba sinensis* (NCBI:txid198412) belongs to the family Uloboridae. Their body size is relatively larger than other members of the family [12], and this species is widely distributed in East Asia, Southeast Asia and North America [2]. Their habitat is close to human buildings, and the populations are large, so they can be easily collected in cities (Figure 1A, B). These aforementioned characteristics make it an ideal model species of the Uloboridae to study the biological characteristics of this family.

In this study, we generated a chromosome-level genome assembly of *O. sinensis*. High-quality annotation was performed on the genomes of *Pardosa pseudoannulata* and *Dolomedes plantarius*, as well as optimization of annotation for the genomes of *Uloborus diversus* and *Latrodectus elegans*. Furthermore, we assembled a full-protein reference for *Deinopis* sp. using transcriptome data. By leveraging multi-omics datasets from various spider species, we explored the molecular basis of the unique adaptive strategies in the family Uloboridae. In the selection pressure analysis, we found that some important genes related to muscle movement have undergone significant positive selection. In gene family analysis, we observed that with the absence or relaxation in selection pressure of toxins and developmental related genes. Gene families related to tracheal development, skeletal development, and tissue force-bearing structures have significantly expanded or emerged in the genomes of this group.

Moreover, energy metabolism related genes exhibit high expression, and enzyme activity in related pathways is significantly increased. These all provide a plausible explanation for the improvement in respiratory efficiency and the well-developed tracheal system observed.

## Results

### 1. Prey-wrapping behavior observations and fang morphology

Through our observations, like other species in the family Uloboridae, *O. sinensis* only arrest their prey through extensive silk wrapping. The time usually exceeds three minutes (sometimes even eight–nine minutes), during which there may be several brief breaks (Additional file 1–3). This time is much higher than the previously recorded wrapping time of other spider species (Araneoidea, 10.1 ± 1.2 seconds for flies, 43.2 ± 6.7 seconds for dragonflies) [6].

In addition, for the first time, we examined the fangs of *O. sinensis* from multiple angles with a Scanning Electron Microscope (SEM). Generally speaking, if a species has venom glands, a channel opening for injecting venom should be found on the fangs [1, 13]. We did not observe this in *O. sinensis*, which further provides evidence that uloborids are not equipped to deliver venom (Figure 1C, D).

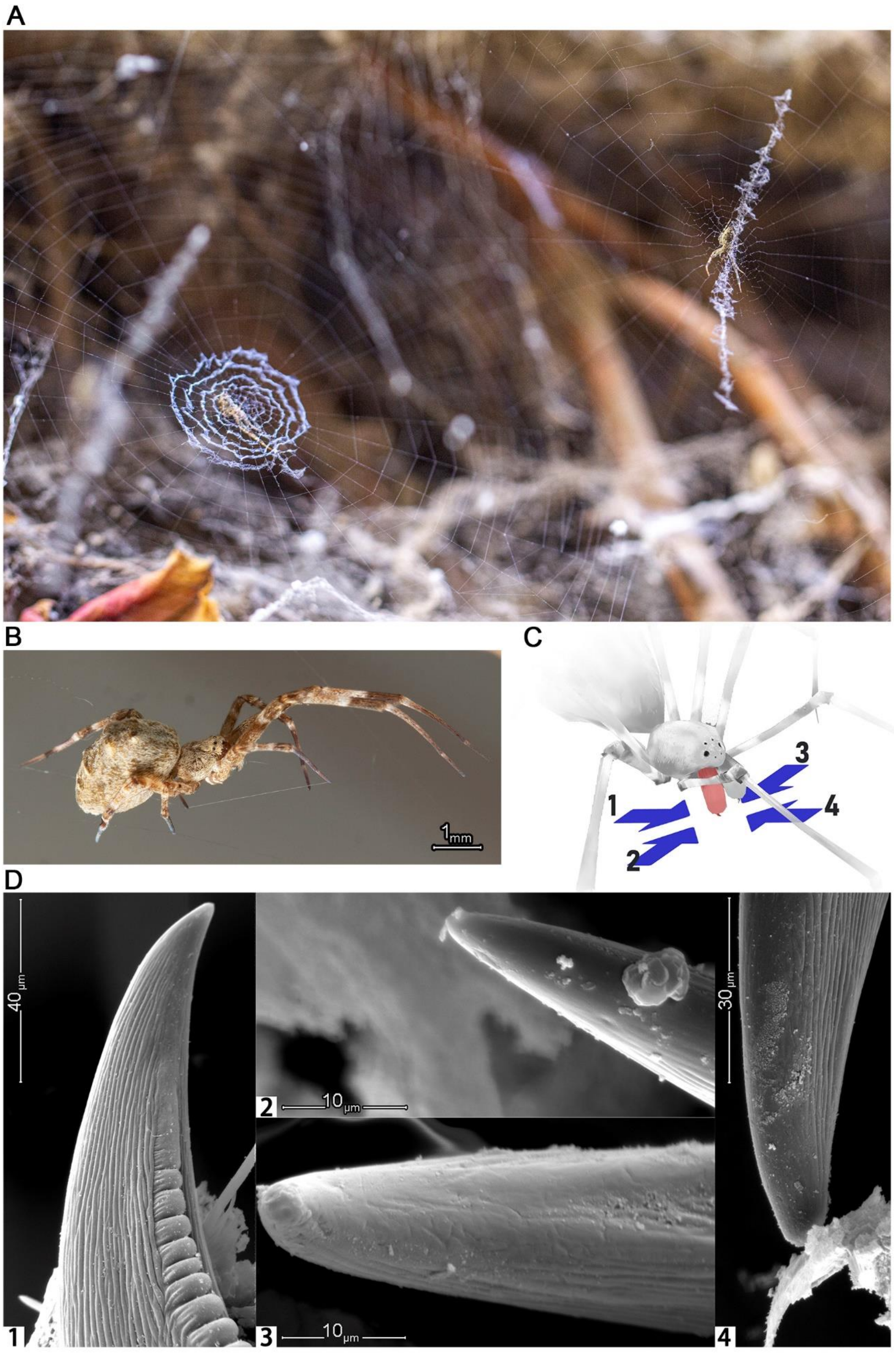
A
B
1mm
C
1
2
3
4
D
40 µm
2
10 µm
30 µm
1
3
10 µm
4

Figure 1: Observations of *Octonoba sinensis*. (A) Two specimens of *O. sinensis* on their orb webs in their natural environment. (B) Adult female of *O. sinensis*. (C) The perspective diagram in (D), with arrows numbered 1–4 indicates the shooting angles of the four images in (D). (D) Scanning electron microscope image of *O. sinensis* fangs.

**2. Genome assembly and annotation**

We assembled an *O. sinensis* genome of 1.34 Gb, which is slightly smaller than the prediction of 1.47 Gb based on Illumina data (Additional file 4: Table S1). The average GC content is 32.57%, N50 value is 139.92 Mb. A total of 20 scaffolds were obtained, of which more than 99.9% of the sequences were loaded onto nine scaffolds that reached the chromosome level (Figure 2A), which was consistent with the previous karyotype analysis of *O. sinensis* [14]. The Benchmarking Universal Single-Copy Orthologs (BUSCO) [15, 16] complete analysis was is 95.3% (arachnida_odb10, Additional file 4: Table S2).

In the annotation of repetitive sequences of the genome, we found that the proportion of repetitive regions was 55.08%, and the most recognizable element was DNA transposons, which accounted for 18.5% of the genome (Additional file 4: Table S3, Additional file 5: Figure S1), similar to other spider groups with genomic data [17–19]. In our assembly, 24,579 coding genes were annotated, and 24,563 genes have obtained effective functional annotation in at least one of the following databases: NCBI-Nr, Swiss-Prot or EggNOG v5.0 databases [20]. The chromosome loading rate of coding genes is 99.09%. The BUSCO assessment of protein level is 94.8% (Additional file 4: Table S2).

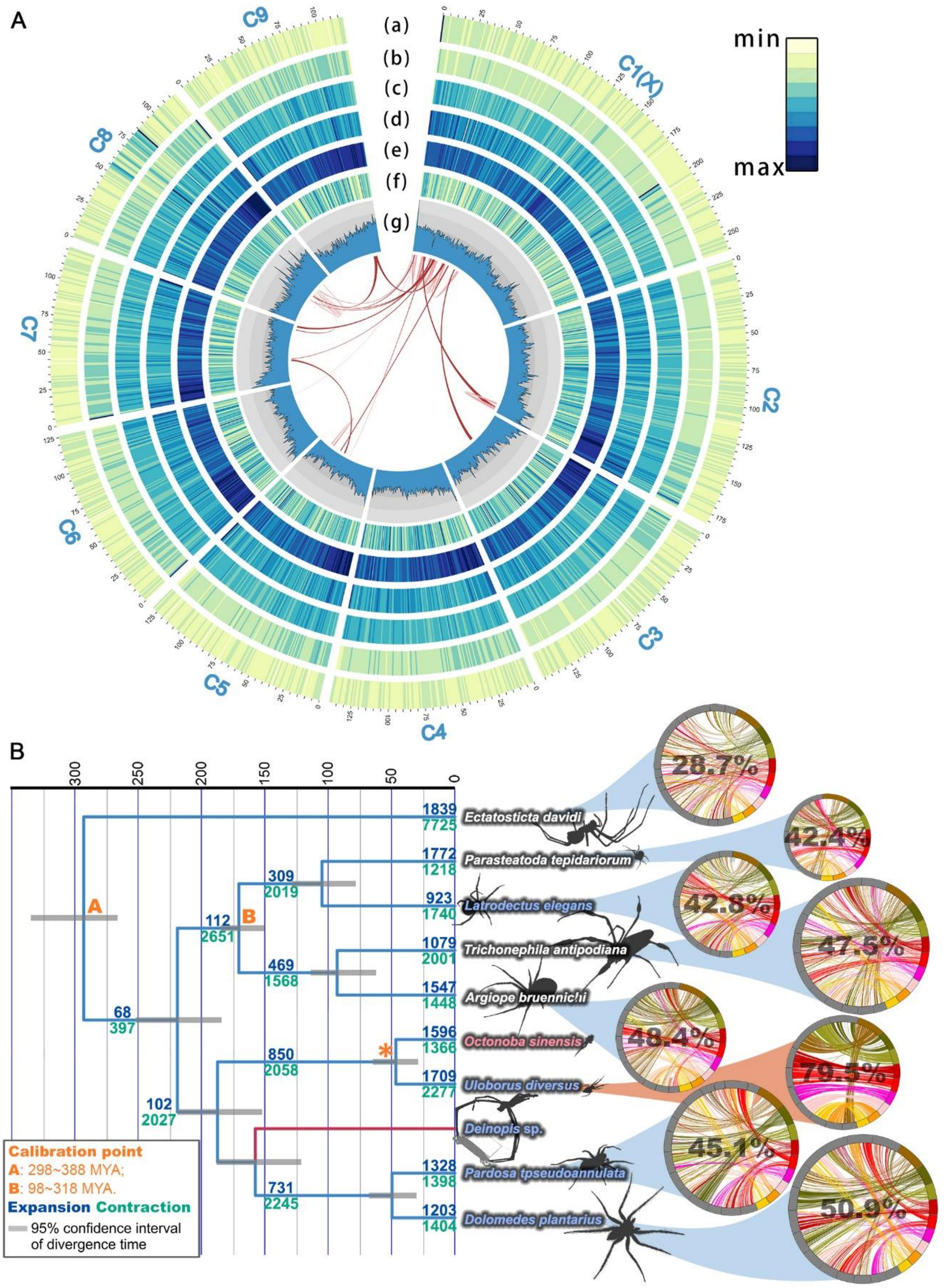


Figure 2: Genome and comparative genomic analysis results from *Octonoba sinensis*. (A) Assembly

and structural annotation of the *O. sinensis* genome. The distribution of different elements is marked with lowercase letters from the outside to the inside in the circle diagram, a: gene; b: SINE, c: LINE, d: LTR; e: DNA transposon; f: Highly-Conserved Elements (HCEs); g: GC content. The inner lines of the circle graph are collineation, dark red lines are collineation between chromosomes, pink lines represent collineation within the chromosome. (B) Phylogeny of multiple spider species. All nodes received 100% bootstrap support. New annotated genomes provided in this study are marked in light blue font, and new genome assemblies and annotations marked in light red font. The 95% confidence interval of the divergence time is represented by the gray bars on the phylogeny, and the number of expanded and contracted gene families at each node is represented by light blue and light green respectively. The asterisk indicates the foreground branch in the selection pressure analysis. The branch with only transcriptome data cannot deploy CAFE analysis and is highlighted by a red branch. The collinearity between *O. sinensis* and other spider species is visualized through circle diagrams positioned adjacent to each respective species. The proportion of collinearity segments within the *O. sinensis* genome is emphasized in bold percentages.

**3. Divergence time estimation and synteny analysis**

Genomes of *O. sinensis*, *U. diversus*[22] as well as the model species — the house spider (Parasteatoda tepidariorum) [21] and seven other representative species (Additional file 4: Table S4) were selected for orthologous gene identification (see methods). A total of 1,560 single-copy gene families shared by all species were identified and used to construct the phylogenetic tree (Figure 2B). All nodes have 100% ultrafast bootstrap support, and the topolog and the estimated divergence time of each node are similar to those of previous studies [23–25].

Among the species involved in the above analysis, genomes with chromosome-level assemblies were selected for synteny analysis with *O. sinensis* (Additional file 4: Table S4). There is a trend that the closer the relationship, the stronger the collinearity (Figure 2B). Because *Deinopis* sp. lack comprehensive genomic data, this species was not included in the synteny analysis (red branch, Figure 2B).

**4. Genes under positive selection and energy metabolism in muscle**

In the selection pressure analysis, at the node of the Uloboridae (asterisk, Figure 2B), 401 genes are under positive selection (Additional file 4: Table S5). Although these genes did not achieve effective GO enrichment (p. adjust < 0.05), we found that there is tissue preference in the expression of some positive selection genes (PSGs), and, in *O. sinensis*, these genes have the highest enrichment in embryos and muscles (Figure 3A). It is worth noting that these PSGs in muscle tissue include *myosin* (gene ID: g27351, p-value = 2.59e-04), an important molecular motor [26, 27], and *twitchin* (gene ID: g8872, p-value = 7.55e-05), a key regulator of muscle movement [28].

To further explore the evolution of the motor function of Uloboridae, we compared the transcriptome data of the legs between *O. sinensis* and other species. We used the model species *P. tepidariorum*, which is also a web-building spider, as a control. Under consistent standardized conditions, the results showed that a large number of genes were differentially expressed (Figure 3B, Additional file 4: Table S6). Compared to the legs of *P. tepidariorum*, genes with higher expression in the legs of *O. sinensis* were most enriched in the mitochondrial matrix, meanwhile significant enrichment was also observed in other GO terms about mitochondria (Figure 3C, Figure S2). This result suggests that at least in the legs, *O. sinensis* requires greater energy consumption compared to typical web-building spiders. On this basis, we examined the activities of several key enzymes in the mitochondria involved in energy metabolism, including hexokinase (HK), pyruvate dehydrogenase (PDH), α-ketoglutarate dehydrogenase (α-KGDHC), NADH dehydrogenase (ND), and ATP synthase (ATPase). Our results revealed that the activity levels of these five enzymes in the body of *O. sinensis* were higher compared to those in *P. tepidariorum* (Figure 3E). Furthermore, while there was no significant difference in $CO_2$ production rates between *O. sinensis* and *P. tepidariorum* in a resting state, *O. sinensis* exhibited significantly higher rates under fatigue treatment (Figure 3D). These findings suggest that the evolution related to energy metabolism in the motor organs may be a key factor in the sustained output power of species in the Uloboridae family.

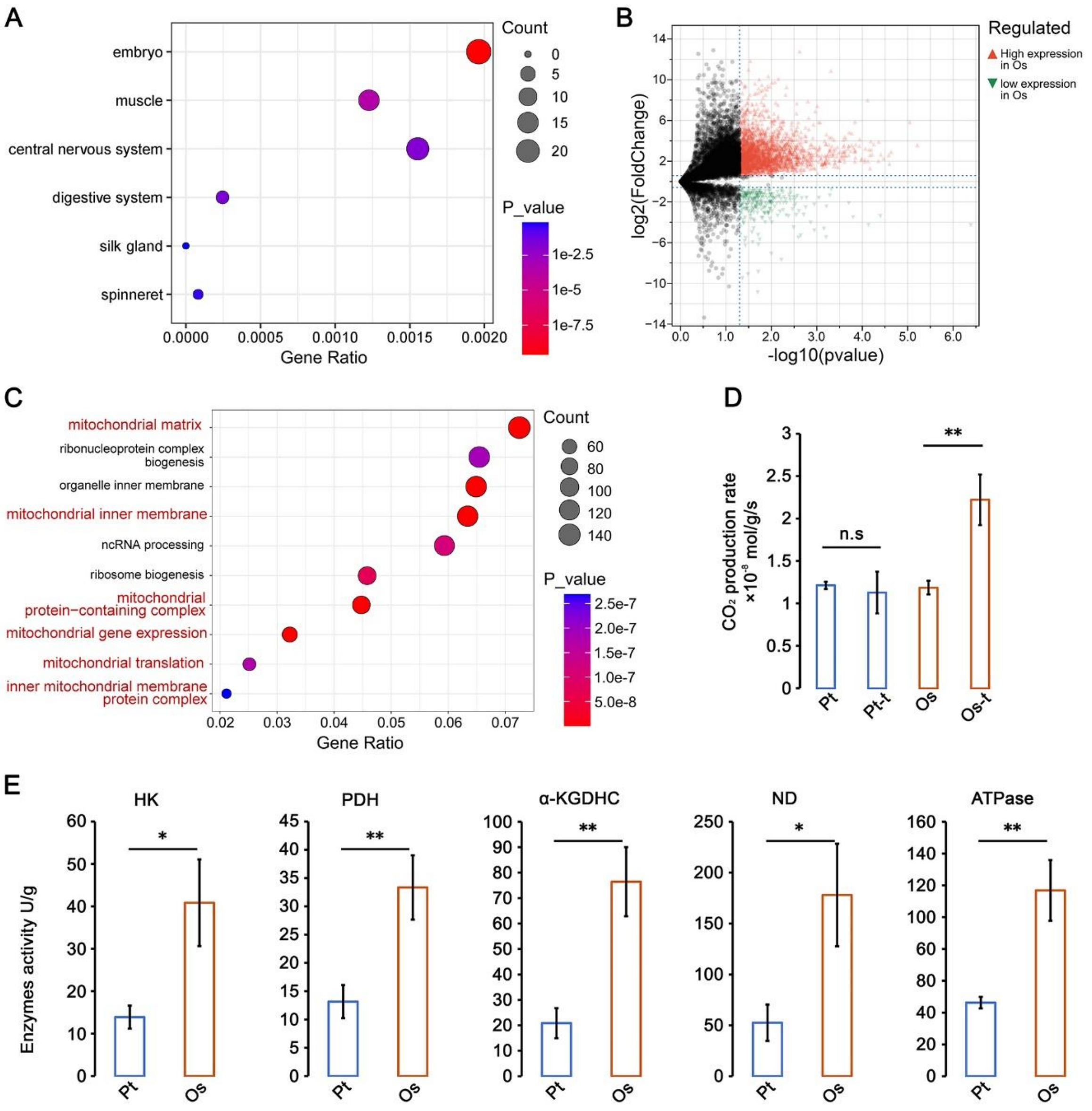


Figure 3: Positive selected genes and energy metabolism. (A) Tissue enrichment of genes under positive selection. (B) Differential expression analysis of homologous genes in the legs of *Parasteatoda tepidariorum* and *Octonoba sinensis* (only Reciprocal Best Hits genes were considered, Fold-change > 1.5, $p < 0.05$). Genes exhibiting higher expression levels in *O. sinensis* legs are designated with red upper triangles, whereas those with higher expression in *P. tepidariorum* legs are designated with green lower triangles. (C) GO terms enrichment analysis. Compared to the legs of *P. tepidariorum*, genes with higher expression (Fold-change > 1.5, $p < 0.05$) in the legs of *O. sinensis* were analyzed. GO terms

related to the mitochondrion are highlighted in red. Metabolic rate measurement (D) and enzyme activity (E) of hexokinase, HK; pyruvate dehydrogenase, PDH; alpha-ketoglutarate dehydrogenase complex, α-KGDHC); NADH dehydrogenase, ND; and ATP synthase, ATPase. Pt: *P. tepidariorum*, Os: *O. sinensis*, -t: under fatigue treatment. Significant differences are denoted by *, $P < 0.05$; **, $P < 0.01$; and n.s., not significant.

**5. Expanded and new emergent gene families**

To compare the genomic differences between Uloboridae and other spiders, we used CAFE v4.2 [29] to analyze the gene family expansions and contractions. Results indicate that 123 gene families have undergone significant expansion at the ancestral node of the Uloboridae (Figure 4A). In *O. sinensis*, we found four of these families to have a high number of annotated genes that corresponded to: FH2 domain containing 1 (FHDC1), FBN1, WD40 repeat proteins (WD40), and seven-(pass)-transmembrane domain receptors 1 (7tm_1) (Figure 4B, Additional file 4: Table S7). Interestingly, studies have shown that FHDC1 proteins not only play a crucial role in the development of the tracheal system in fruit flies [30] but also have significant implications in muscle movement [31, 32]. In addition, the preproprotein of FBN1 is proteolytically processed to generate two proteins including the extracellular matrix component fibrillin-1 and the protein hormone asprosin. Fibrillin-1 is an extracellular matrix glycoprotein that serves as a structural component of calcium-binding microfibrils. These microfibrils provide force-bearing structural support in elastic and nonelastic connective tissue throughout the body. Asprosin has been shown to regulate glucose homeostasis [33]. Apart from the four superfamilies mentioned above, we have deployed GO enrichment analysis of the other expanded gene families. REVIGO [34] results show that these annotated genes were mainly enriched in the transport of carbohydrates and organic acids, the immune system, and the functions related to transposable elements (Figure 4C).

Based on the results of orthologous gene identification, we screened for gene families that are exclusively shared in Uloboridae and not found in any other spider species (species in Figure 2B), designating them as new emergent gene families. 269 such gene families have been identified in both

*O. sinensis* and *U. diversus*, with a total of 658 members in the *O. sinensis* genome (Additional file 4: Table S8). GO enrichment results showed that these genes are more enriched in GO terms that are related to bone trabecular development (Figure 4D).

Generally speaking, spiders lack endurance, necessitating the prompt subduing of their prey within a brief timeframe during hunting [13]. However, uloborids can exercise intensely for nearly an hour [9]. Our results indicate that genes related to tracheal development, skeletal development, tissue force-bearing structures, and energy metabolism have significantly expanded or emerged in the genome of Uloboridae. We believe that the evolution of these aspects is highly likely to be related to their increased endurance.

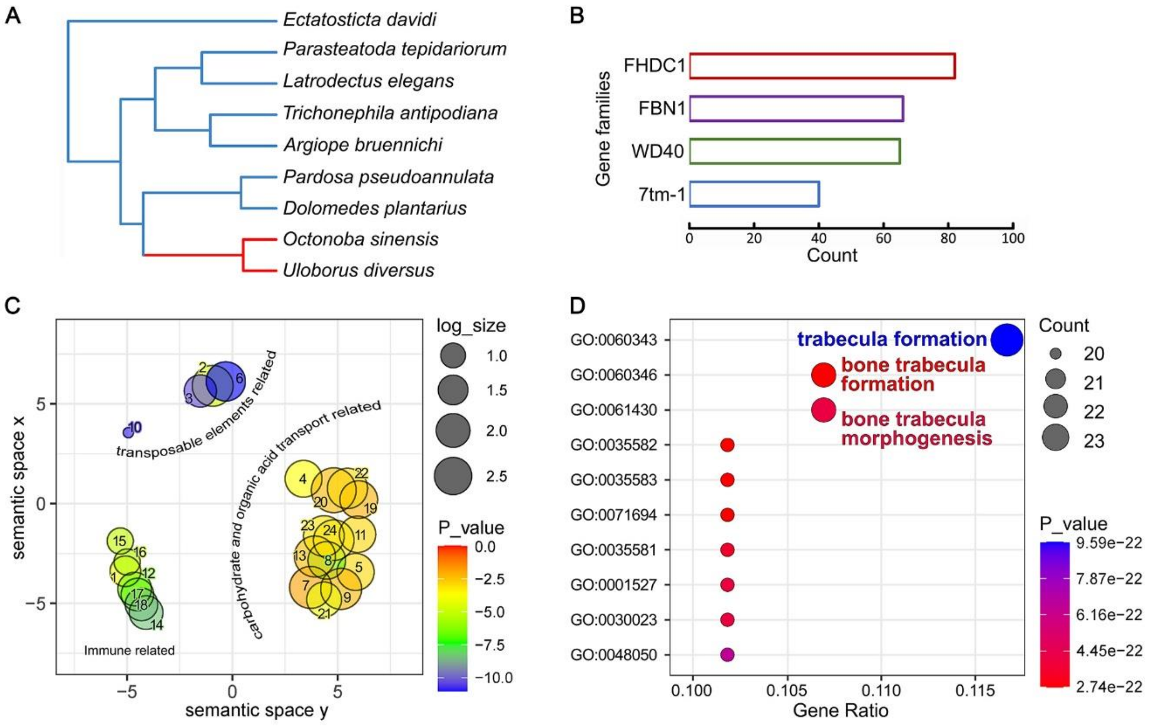


Figure 4. Expanded and new emergent gene families. (A) Species phylogeny for calculating the expansion and contraction of gene families. Uloboridae branches are in red. (B) The four superfamilies with the highest number of clearly annotated genes. (C) REVIGO plot of GO enrichment results for the other expanded gene families, excluding four superfamilies. The numbers in the figure refers to different GO terms: 1-response to molecules of bacterial origin, 2-DNA

recombination, 3-DNA transposition, 4-receptor-mediated endocytosis, 5-carbohydrate transport, 6-DNA integration, 7-organic anion transport, 8-sialic acid transport, 9-organic acid transport, 10-transposition, 11-carbohydrate transmembrane transport, 12-response to type II interferon, 13-carboxylic acid transport, 14-response to interleukin-4, 15-cellular response to biotic stimulus, 16-cellular response to molecule of bacterial origin, 17-cellular response to type II interferon, 18-cellular response to interleukin-4, 19-monoatomic anion transmembrane transport, 20-inorganic cation transmembrane transport, 21-carbohydrate derivative transport, 22-proton transmembrane transport, 23-organic acid transmembrane transport, 24-carboxylic acid transmembrane transport. (D) GO enrichment of new emergent gene families in Uloboridae.

**6. Absent regions and genes under relaxed purifying selection**

Due to the absence of venom glands, the genes or functional regions specifically involved in the venom gland system in Uloboridae may be subjected to relaxed purifying selection or gradually lost from the genome. To obtain this information, Highly-Conserved Elements (HCEs) were searched spanning nine spider genomes (Figure 4A) and the sites with Uloboridae-specific deletions among them were identified (Additional file 5: Figure S3, Additional file 4: Table S9 S10). In addition, absent genes and genes under selective relaxation in the *O. sinensis* and *U. diversus* genomes were analyzed against the background of species with venom glands (Figure 2B, Additional file 5: Figure S3, Additional file 4: Table S11 S12). We conducted functional enrichment on the above results and found that the biggest difference between Uloboridae and background species comes from the development, especially the neuro-development, related gene family (Figure 5A, B).

In the homologs missing from Uloboridae, we found that five genes belong to two toxin related gene families (LRR and CRISP), as well as three transcription factors. It is worth noting that the expression patterns of the homologs of two LRR genes (LOC122270931, LOC107442855) in *P. tepidariorum* indicate their highest expression in venom glands, although neither of them has been annotated as homologs to known toxin genes (Figure 5C). Three transcription factors belong to important components that activate transcription: “protein c-ets-2”; the “cooled coil and C2 domain containing

protein (CC2D)” that regulates neurotransmitter expression; and the component of the STAGA complex: “ataxin-7”.

In addition, we referred to the venom gland-specific expression module of *P. tepidariorum* obtained in a previous study (n = 1,088, Additional file 4: Table S13) [21] and found that only one gene (LOC107440400), which is specifically lost in Uloboridae, intersects with this module. This gene is a SOBP (Sine Oculis-Binding Protein Homolog), and the protein encoded by this gene is involved in the development of the cochlea, and genetic defects are also related to intellectual disability (Figure 5C).

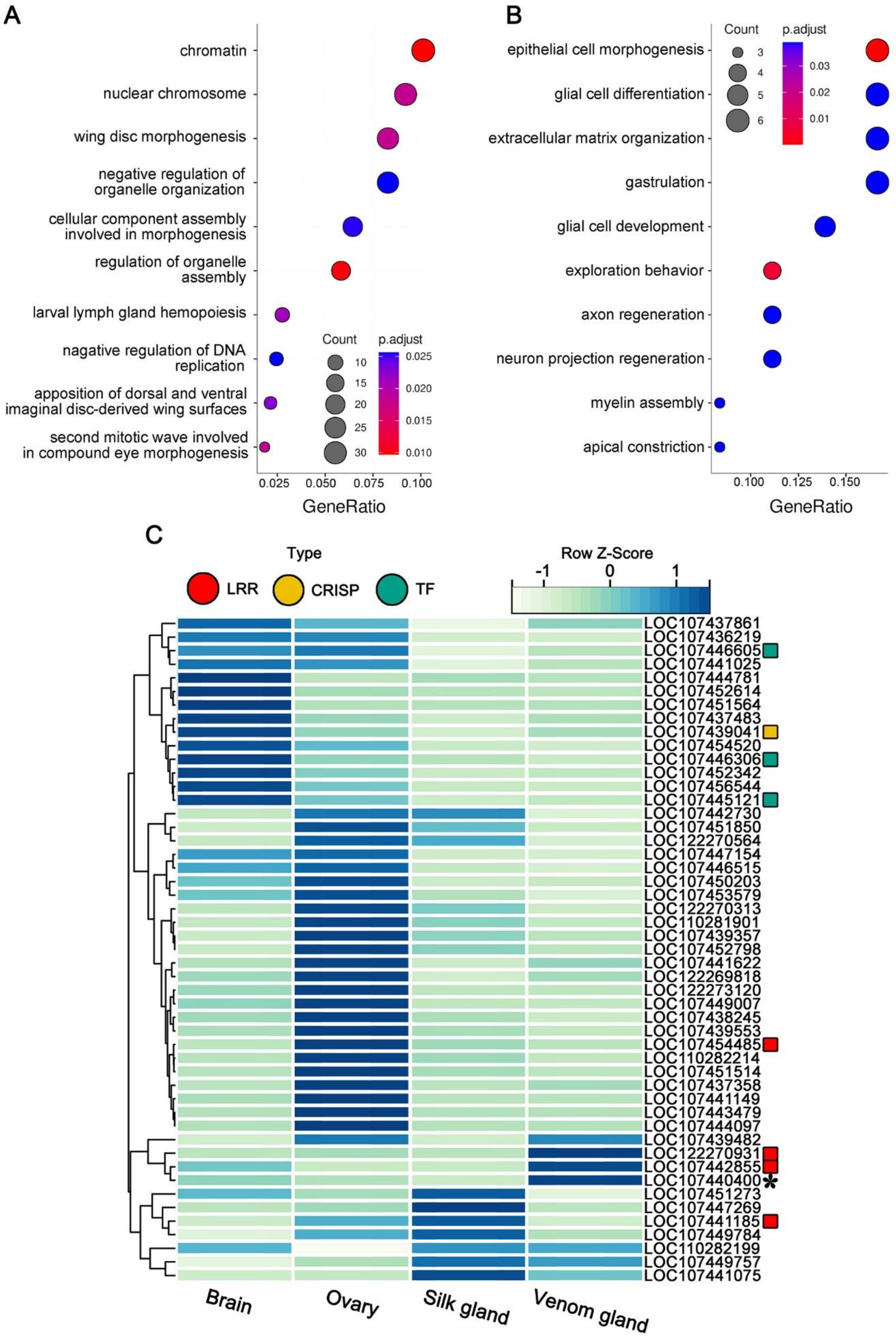
A
chromatin
nuclear chromosome
wing disc morphogenesis
negative regulation of organelle organization
cellular component assembly involved in morphogenesis
regulation of organelle assembly
larval lymph gland hemopoiesis
nagative regulation of DNA replication
apposition of dorsal and ventral imaginal disc-derived wing surfaces
second mitotic wave involved in compound eye morphogenesis
Count
10
15
20
25
30
p.adjust
0.025
0.020
0.015
0.010
0.025
0.050
0.075
0.100
GeneRatio
B
epithelial cell morphogenesis
glial cell differentiation
extracellular matrix organization
gastrulation
glial cell development
exploration behavior
axon regeneration
neuron projection regeneration
myelin assembly
apical constriction
Count
3
4
5
6
p.adjust
0.03
0.02
0.01
0.100
0.125
0.150
GeneRatio
C
Type
LRR
CRISP
TF
Row Z-Score
-1
0
1
LOC107437861
LOC107436219
LOC107446605
LOC107441025
LOC107444781
LOC107452614
LOC107451564
LOC107437483
LOC107439041
LOC107454520
LOC107446306
LOC107452342
LOC107456544
LOC107445121
LOC107442730
LOC107451850
LOC122270564
LOC107447154
LOC107446515
LOC107450203
LOC107453579
LOC122270313
LOC110281901
LOC107439357
LOC107452798
LOC107441622
LOC122269818
LOC122273120
LOC107449007
LOC107438245
LOC107439553
LOC107454485
LOC110282214
LOC107451514
LOC107437358
LOC107441149
LOC107443479
LOC107444097
LOC107439482
LOC122270931
LOC107442855
LOC107440400
*
LOC107451273
LOC107447269
LOC107441185
LOC107449784
LOC110282199
LOC107449757
LOC107441075
Brain
Ovary
Silk gland
Venom gland

Figure 5: Absent regions and regions under relaxed selection. (A) GO enrichment of genes under relaxed selection in *Octonoba sinensis*. (B) GO enrichment of genes related to missing Highly-Conserved Elements (HCEs) in *O. sinensis*. (C) The expression patterns of the genes specifically absent in Uloboridae were examined in the model species *Parasteatoda tepidariorum*. The heatmap is plotted based on the Z-score transformed from Transcripts Per Million (TPM) values. Different colored markers are used to distinguish between distinct gene types, while an asterisk identifies the gene that is notably absent from the venom gland-specific expression module of *P. tepidariorum*.

**7. Deficiency of toxin genes in *O. sinensis***

To search for toxin genes in *O. sinensis*, we integrated the results of previous studies and established a comprehensive toxin protein database (Additional file 6) and screened toxin gene homologs with the same threshold in different species. In the *O. sinensis* and *U. diversus* genomes, we identified 12 and 11 homologs, respectively, that have similar structures to members of six major toxin or venom component gene families (Latrotoxin, Latrodectin, CRISP, ICK, TCTP, EF-hand and ctenitoxin). Our findings indicate that although the Uloboridae family has a relatively low number of toxin-related homologs, their count still exceeds that of some venomous spiders, such as *Ectatosticta davidi* [35–38] (Additional file 4: Table S14). Nevertheless, we discovered that none of these *O. sinensis* genes' orthologs in *P. tepidariorum* exhibit high expression levels in venom glands (Figure 6). Given that *O. sinensis* had three latrotoxin homologs out of 12 toxin homologs (highest category, Table S14) and latrotoxins are not known outside of Theridiidae, a phylogenetic analysis of this gene family has been conducted. In this analysis, we found that the three homologs in *O. sinensis* are not clustered on the same branch as the reported Latrotoxin (Additional file 5: Figure S4) [39]. There is a hypothesis regarding the evolution of venom components that ancestors of toxin proteins were originally proteins with normal physiological functions that were recruited in venom glands to play the role of venom components [40], and our results also support this viewpoint.

To further explore the evolutionary processes of *O. sinensis* toxin genes in the absence of venom glands,

we conducted a pseudogene search on non-coding regions of the *O. sinensis* genome, but traditional search methods did not identify pseudogenes (blastn, E-value 1e-5, matching length 50 bp) [41]. However, we did find more traces of toxin gene homologs in the blastx search through the protein sequence in the toxin genes database. These results include 48 different genomic regions, but only one of which had an effective hit with the relatively reliable toxin gene of *P. tepidariorum* (LOC107440051) (Additional file 4: Table S15, Figure 6).

By searching for toxin gene homologs in collinearity fragments of *O. sinensis* and *P. tepidariorum*, a particular class of genes was found in *O. sinensis*. These genes are located in the same place as the *P. tepidariorum* toxin gene homologs in the collinearity segment, but they can no longer be identified as toxin genes (below the minimum recognition threshold, see methods) (Figure 7B, red ribbon). There are three pairs of such genes, includes one pair of CRISP genes (g31478~LOC107437238) and two pairs of ICK genes (g6736~LOC107446942 and g6736~LOC107446932). Their expression patterns in *P. tepidariorum* indicate that only the CRISP gene is a reliable toxin gene (Figure 6, black squares). In *O. sinensis*, this CRISP gene (g31478) cannot be unambiguously classified as a homolog of toxin genes due to changes in protein structure (Additional file 4: Table S16). However, compared with other toxin genes which it is difficult to find pseudogenes, the ortholog of this CRISP gene in *O. sinensis* have complete gene structures, and can be expressed in multiple tissues of a venomless spider (Additional file 4: Table S17). These all indicate that this CRISP gene (g31478) must play the role of a non-toxic gene. We believe that this observation suggests a potential functional shift between toxic and non-toxic genes in spiders.

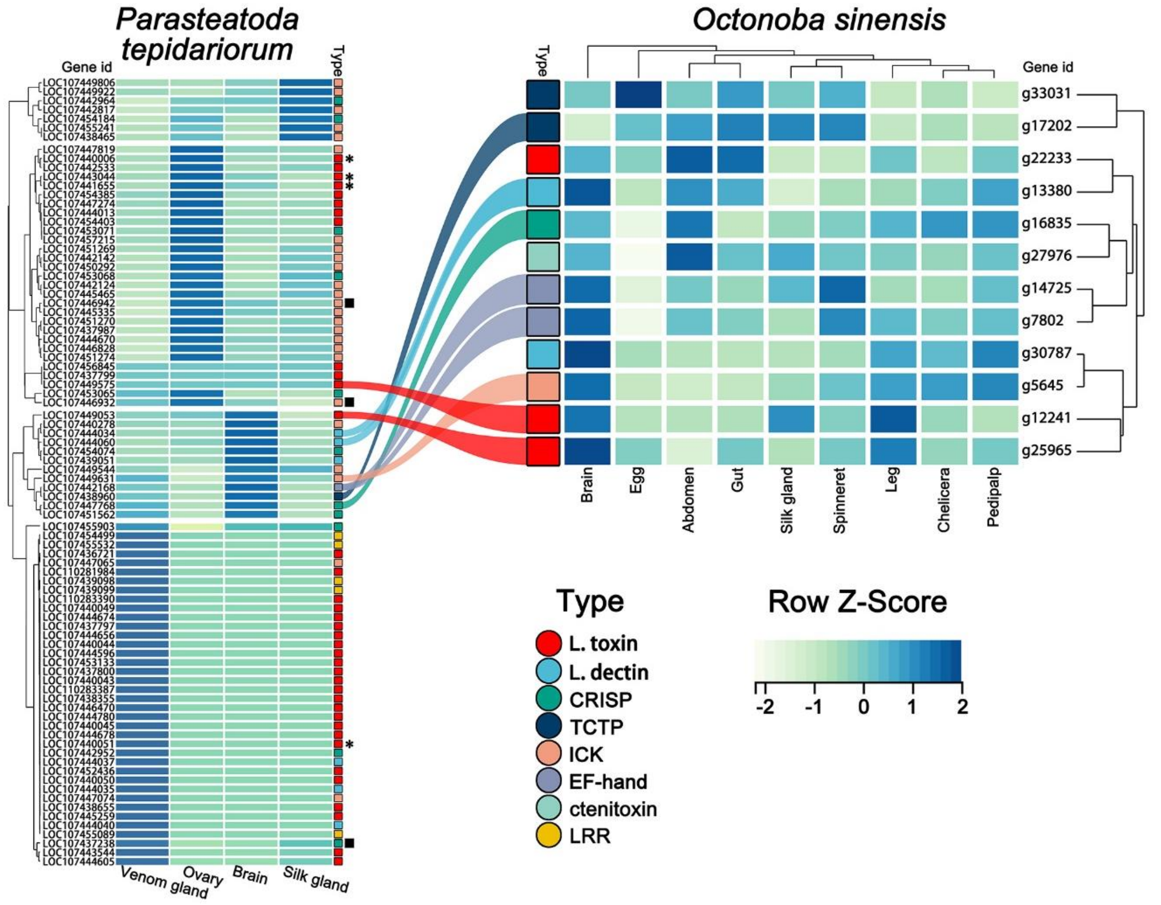


Figure 6. The expression patterns of toxin homolog coding genes in *Octonoba sinensis* and *Parasteatoda tepidariorum*. The heatmap is plotted based on the Z-score transformed from Transcripts Per Million (TPM) values. The ribbon connects the homologs genes. The black asterisks indicate the best match of possible *O. sinenesis* pseudogenes in *P. tepidariorum*. The black squares indicate homologs matching the *O. sinenesis* existence position in the collinearity (see Figure 7B: red ribbons).

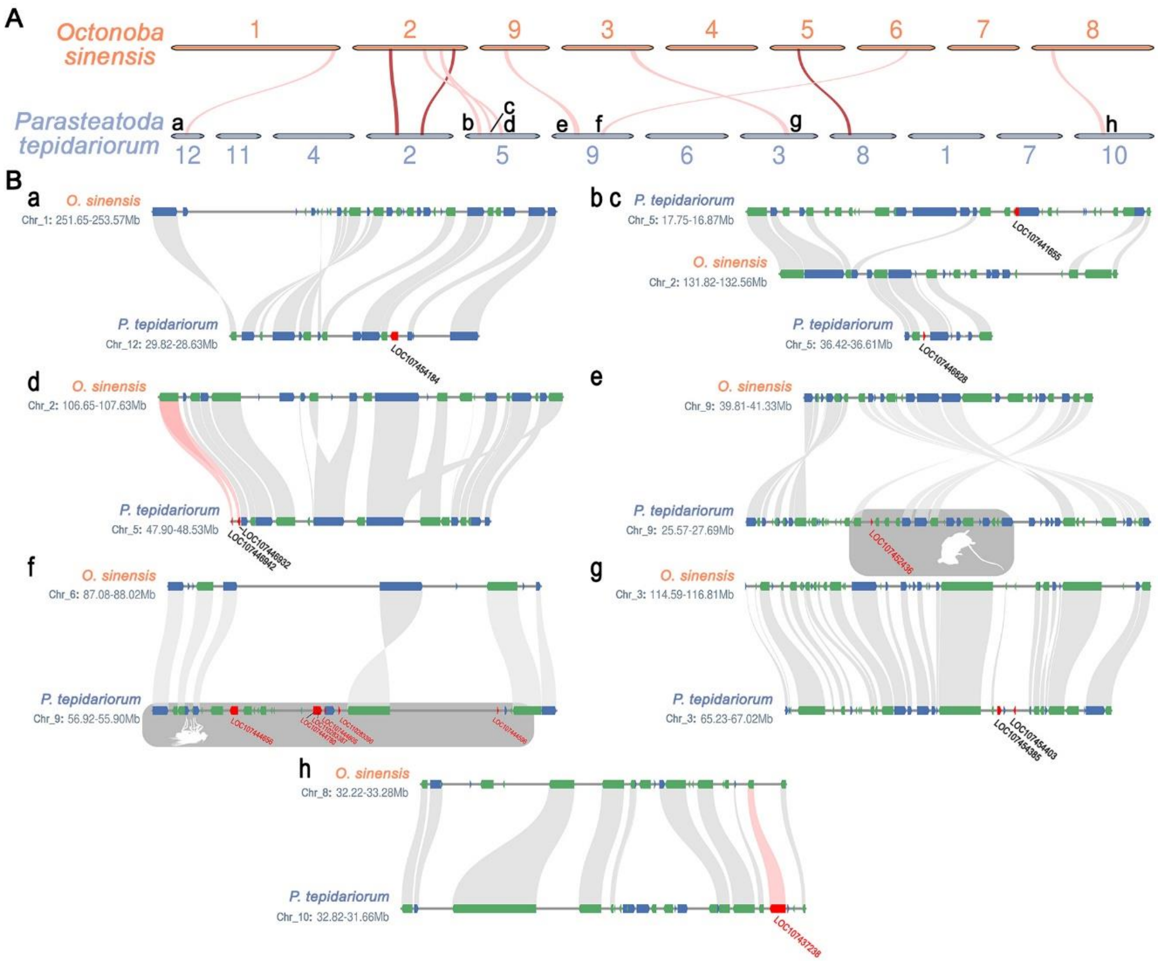


Figure 7. Collinearity containing toxin homologs between *Octonoba sinensis* and *Parasteatoda tepidariorum*. (A) Collinearity of all toxin homologs. If the toxin homologs were lost in *O. sinensis*, the collinearity fragments are represented by a pink ribbon, and otherwise by a dark red band. (B) Gene correspondence in each collinearity fragment where toxin gene deletion occurs in (A). Genes located on the positive strand of the genome are represented in blue, while those located on the negative strand are represented in green. Each lowercase letter corresponds to the collinearity fragments represented by the same letter in (A); the toxin gene names in *P. tepidariorum* are listed, and the red gene names highlight the toxin genes that are highly expressed in the venom glands. The toxin genes in the gray background belong to the Latrotoxin gene family, and different silhouettes indicate the type of toxin and the target group of toxicity. Mouse = vertebrates (alpha-latrotoxin), Fly = insects (delta-latrotoxin); the red ribbons indicate that the collinearity of the linked genes is extremely weak, below the threshold (see methods).

## Discussion

During evolution, the loss of important organs is accompanied by a series of adaptive evolution. such as the enhancement of non-visual senses of some eyeless organisms [42–46]. In Uloboridae, due to the absence of venom glands, prey-wrapping as the sole attack strategy is excessive. Multi-omics analysis revealed the absence or possible functional shift of toxin genes in *O. sinensis*. At the same time, compared to venomous spiders, a series of genomic changes related to muscle, aerobic respiration and metabolism of energy substances were observed in the genomes. These findings imply an evolutionary trade-off between the loss of venom glands and the enhancement of physical attack strategies.

Both gene family evolution and selection pressure shaped the genomic change related to the specialized physical attack of Uloboridae. For example, *myosin* and *twitchin* were under positive selection. Myosin, provides the driving force for muscle contraction as a molecular motor [26, 27], while twitchin, is located at the junction of muscle fibers and regulates the speed and force of muscle contraction [28]. The positive selection of these two genes suggests that they may play a role in the strengthening of muscle energy efficiency and contraction strength. Additionally, the gene family expansions of key proteins involved in muscle fiber connection, FHDC1 [31, 32], and the structural component of connective tissue, FBN1 [33], likely provided genetic resources for the optimization of muscle and connective tissue.

Aerobic respiration is the process that directly supplies energy to muscles. We found a higher expression of mitochondria-related genes in the legs of *O. sinensis* compared to *P. tepidariorum* (a venomous spider) (Figure 3C). In addition, physiological and biochemical measurements also indicate an advantage of uloborids in respiratory efficiency and related enzyme activity (Figure 3D, E). Moreover, the expansion of gene families involved in tracheal development [30], coupled with previous records that uloborids possess a more complex tracheal system compared to other web-building spiders [10, 11], all indicates that uloborids have the ability to provide sufficient oxygen supply for enhanced aerobic respiration.

The supply of energy substances is also crucial for sustained physical output. We found that the gene families involved in the transport of carbohydrates and organic acids have undergone expansion (Figure

4C), which may help uloborids enhanced utilization and metabolism of substrates (or intermediate products) in aerobic respiratory. Additionally, asprosin, encoded by an expanded gene family [33], acts as a hormone regulating glucose homeostasis, potentially mobilizing energy reserves to further provide energy for long-term physical activity.

Simultaneously, through analyzing expression patterns and synteny relationships, we discovered that toxin genes in venomous spiders are expressed in other tissues of uloborids and may perform non-toxic functions. This observation suggests a potential functional shift between toxic and non-toxic genes in spiders. In addition to toxin genes, the absence of certain genes exclusively in uloborids raises concerns. These include protein c-ets-2, CC2D, and ataxin-7, which are transcription factors involved in various metabolic and developmental processes. Additionally, notable missing genes include the SOBP gene and two LRR genes. The former is involved in nervous and organ development, while the latter has a similar structural domain to LRR toxin genes, all of which are highly expressed in venom glands of model species.

For predators, there exists a pervasive evolutionary trade-off between chemical and physical attack strategies. Previous research has frequently favored the exploration of chemical strategies, particularly venom. However, against the backdrop of nearly all spiders being toxic predators, our study delves into the genetic basis underlying the alternative choice in this trade-off. Unsurprisingly, reliable toxin gene was not identified in Uloboridae, but these adaptive evolutions ranging from muscle to aerobic respiration and then to supply of energy substances, provide strong support for the exceptional physical endurance demands of this group, and compensate for their decreased adaptability due to the absence of venom glands. Furthermore, some development-related gene and element deletions were observed in uloborids. Although the association between these deletions and the absence of spider venom glands remains unclear, they still hold potential for exploring the evolutionary mechanisms underlying this phenomenon.

## Methods

### 1. Sample collection and DNA extraction

Live *O. sinensis* were field-captured from Olympic Park, Chaoyang District, Beijing, China. To minimize the contamination of impurities in the digestive tract as much as possible, all samples were starvation-reared for more than one week at room temperature. Genome DNA for both short and long read sequencing were isolated from the cephalothoraxes of adult female spiders using the Qiagen Blood & Cell Culture DNA Kit (QIAGEN, Hilden, Germany).

**2. Observation of predation behavior and examination of fangs**

We recorded a series of videos to observe the predation behavior, using Logitech StreamCam 960-001282. Wrapping duration was timed. If the spider stopped for more than three seconds, the timing also stopped but would continue if it began again. If the spider begins to soak (*O. sinensis* will emit liquid onto their prey before eating) or leave the prey, the video is terminated.

The dissected spider fangs were pasted onto a copper substrate at different angles, dried with a $CO_2$ critical point drying method, coated with gold, and then observed with the SEM (model: FEI Quanta 450).

**3. Genome sequencing and genome size estimation**

Short-insert libraries of *O. sinensis* were sequenced with the DNBSEQ-G400 (RRID:SCR_017980) using paired-ends (PE) reads of 150 bp (BGI, Shenzhen, China). To remove low-quality reads and adapters, raw reads were trimmed by Trimmomatic (RRID:SCR_011848) v0.39 [47]. A total of 259.82 Gb of clean data were obtained for *O. sinensis* for survey analysis and assembly correction.

For long read sequencing, 'SMRTbell' (double-stranded DNA template capped by hairpin loops at both ends) libraries were constructed according to the standard protocol of PacBio using 15 kb of preparation solution (PacBio, California, USA). The high-fidelity (HiFi) libraries were sequenced on three SMRT cells on the PacBio Sequel II system (RRID:SCR_017990) in Circular Consensus Sequencing (CCS) mode at Novogene Technology Co. and generated 67.18 Gb HiFi data (3,967,026 reads) total [48].

To further improve the continuity of the assembled genomes, chromosome conformation capture (Hi-C) experiments were conducted [49]. Hi-C libraries were prepared following a published protocol with minor modifications [50]. For cross-linking, samples were fixed with 1% formaldehyde. The cross-linked DNA was digested with MboI restriction endonuclease and marked with biotin-14-dCTP to

remove non-ligated DNA fragments. The ligated DNA was extracted with a QIAamp DNA Mini Kit (QIAGEN). The purified DNA was then sheared to ~350 bp fragments and followed by a standard Illumina library preparation protocol [51]. Hi-C sequencing of *O. sinensis* was conducted on the DNBSEQ-G400 platform with PE 150 bp (BGI, Shenzhen, China). We then filtered the raw reads using Juicer v1.6.2 [52] to remove low-quality reads and adapters, yielding 201.99 Gb of clean data.

Before *de novo* assembly, we estimated the genome size of this species. Using the Illumina data, Jellyfish (RRID:SCR_005491) (v2.1.3) [53] was employed to calculate the frequency of each K-mer (k = 17–31). Then, the genome size of *O. sinensis* was estimated using a method based on K-mer distribution.

**4. *De novo* Genome assembly and quality assessment**

PacBio reads were first assembled using two *de novo* assemblers: hifiasm (RRID:SCR_021069) v0.15.2 [54] and wtdbg2 (RRID:SCR_017225) v2.5 [55]. The best assembly was selected according to the optimal continuity and completeness. The final version of contigs was polished with Racon (RRID:SCR_017642) v1.4.17 for three rounds based on long reads, and NextPolish (RRID:SCR_025232) v1.4.0 [56] using short reads. Contig level genome completeness assessment was performed using BUSCO (RRID:SCR_015008) v5.2.2 [15, 16]. Genome consistency assessment was evaluated by mapping the short reads to the genome with Minimap2 (RRID:SCR_018550) v2.24-r1122 [57] and SAMtools/BCFtools (RRID:SCR_005227) v1.10 [58].

We used Hi-C-based proximity-guided assembly to generate chromosomal level genome assemblies for *O. sinensis*. Hi-C library sequencing data were mapped to the contig level genome using Juicer (RRID:SCR_017226). The 3D-DNA (RRID:SCR_017227) v180922 [59] pipeline was executed to construct the chromosomes and correct the errors. We further performed correction with Juicebox Assembly Tools (RRID:SCR_021172) v1.11.08 [60]. The completeness of the chromosomal level assembly was assessed by BUSCO.

**5. RNA extraction, sequencing and expression analysis**

Different tissue samples (spinneret, leg, brain, gut, silk gland, pedipalp, chelicera and abdomen) from adult female *O. sinensis* and eggs (development stage undetermined) were dissected for total RNA

extraction using an RNAsimple Total RNA kit (TIANGEN, Beijing, China). The RNA-seq libraries were constructed with insert sizes of ~150 bp and sequenced on the NovaSeq 6000 platform (RRID:SCR_016387). We produced about six Gb of data per sample. Low-quality reads, reads with adapters, and unknown bases were filtered using Trimmomatic (RRID:SCR_011848).

In addition, legs from adult female *P. tepidariorum* were subjected to transcriptome analysis according to the above process (see Availability of data and materials), and transcriptome data from four tissue transcriptomes (PRJNA934108, including brain, ovary, silk gland and venom gland) were downloaded from the Sequence Read Archive (SRA) database.

Clean reads were aligned to the genome using Hisat2 (RRID:SCR_015530) [61], followed by the quantification of all samples with HTSeq (RRID:SCR_005514) [62] to determine the count value. Subsequently, TPMs were derived through automated scripts.

In the differential expression analysis across two species, only Reciprocal Best Hits (RBH) genes were extracted for quantification. To facilitate comparison, ortholog (RBH) gene IDs of *O. sinensis* were replaced by *P. tepidariorum* gene IDs for comparison and figure illustration. Finally, differential expression analysis was conducted by R package LIMMA (RRID:SCR_010943) [63].

**6. Genome annotation**

The RepeatModeler (RRID:SCR_015027) v2.0.2 [64] and RepeatMasker (RRID:SCR_012954) v4.1.2-p1 [65] pipelines were used to annotate repetitive sequences in the genome.

Gene annotation was based on the braker (RRID:SCR_018964) v2.1.6 [66] pipeline, which combines the whole protein sequences of the nine other species in this study (Additional file 4: Table S4) and more than 320 Gb of multi tissue transcriptome data for comprehensive annotation.

Gene function annotation is based on NCBI-Nr, Swiss-Prot (RRID:SCR_021164) and EggNOG (RRID:SCR_002456) v5.0 databases. The tRNA was predicted using the program tRNAscan-SE (RRID:SCR_008637) v2.09 [67]. Other non-coding RNAs were annotated with the Rfam (RRID:SCR_007891) v14.8 [68] database through infernal (RRID:SCR_011809) v1.1.4 [69].

Among the other species involved in this study, *P. pseudoannulata* [70] and *D. plantarius* only have assembled sequence data currently. The genome of *L. elegans* has high assembly quality[37], but the

protein BUSCO score is only 63.7%; *U. diversus* only has 15,750 annotated protein coding genes, significantly less than other spider species. To obtain more reliable results for downstream analysis, we annotated the genomes of the aforementioned species based on transcriptome data. For *P. pseudoannulata*, *D. plantarius* and *L. elegans*, we used our annotations, and for *U. diversus*, we used our annotation to supplement the original one. All annotation strategies are based on transcriptome data from the SRA (Additional file 4: Table S18) database and carried out through the TransDecoder (RRID:SCR_017647) pipelines. We also added *Deinopis* sp. of Deinopidae and assembled all of its proteins sequence using transcriptome data (DRR297048), quality control was done using fastp (RRID:SCR_016962) version 0.21.0 [71]. De novo assemblies were done using Trinity (RRID:SCR_013048) v2.11.0 [72] under default settings. ORF prediction was done using TransDecoder. Redundancy reduction was done with CD-HIT (RRID:SCR_007105) version 4.8.1 (-c 0.98 -n 10) [73]. All the newly provided annotations mentioned above have a protein BUSCO score of over 90% (Additional file 4: Table S2).

**7. Orthologous gene identification, phylogenetic and synteny analysis**

We used OrthoFinder (RRID:SCR_017118) v2.5.4 [74] to analyze the annotation information of species in this study (Additional file 4: Table S5). In the pipeline, Mafft (RRID:SCR_011811) v7.453 [75] was used to perform multiple sequence alignment, blastp (RRID:SCR_001010) v2.9.0+ [76] was used to perform sequence searches, and the phylogenetic tree was constructed using IQ-TREE (RRID:SCR_017254) v2.2.0 [77]. The calibration points are from fossil specimens [23]. The analysis was run twice, once to calculate the divergence time of different species, and the second to calculate the expansion and contraction of gene families. The latter did not include *Deinopis* sp., as the only transcriptome data cannot determine the number of gene copies. (Figure 4A).

The phylogenetic tree of latrotoxin homologs (Figure S4) was reconstructed (1,000 bootstrap) using the neighbor-joining method, following alignment of the full-length protein sequences via Mafft v7.453.

Collinearity analysis was conducted between *O. sinensis* and other species with chromosome level genomes (*T. antipodiana*, *A. bruennichi*, *L. elegans*, *U. diversus, P. pseudoannulata*, *D. plantarius* and *E. davidi* [78].) using the MCscan (RRID:SCR_017650) pipeline (Python version) in the jcvi toolkit

(RRID:SCR_021641) [79].

**8. Test for selection pressure and gene family expansions/contractions**

One-to-one ortholog identification among ten species (Figure 2B) was performed using the RBH method by blastp v2.9.0+. *O. sinensis* was used as a reference species. Finally, 5,848 RBH clusters (Additional file 4: Table S19) were retained for analysis.

To scan for genes under positive selection in *O. sinensis*, RBH clusters were used for the branch site model analysis using CODEML in PAML v4.9j toolkit [80]. Each gene family sets *O. sinensis* and *U. diversus* as foreground branches (Figure 2B). "Model A" and "Model A-null" models were compared. "Model A" assumes that the selection pressure of foreground branches is greater than that of background branches, and "Model A-null" is an alternative hypothesis.

To statistically test which genes of Uloboridae are under relaxed purifying selection, we used RELAX in the HYPHY (RRID:SCR_016162) v2.5.2 [81] toolkit to infer the free relaxation parameter k at the node of *O. sinensis* and *U. diversus* branches for genes shared by all species in the Phylogeny of Figure 2B (Additional file 4: Table S4). The relaxation parameter k is an exponent for selection parameters between the foreground and the background branches. A $k > 1$ suggests selection is more intensified in the foreground branch vs. the background branch and vice versa.

For gene family expansions/contractions, mcmctree in the PAML (RRID:SCR_014932) v4.9j toolkit was used to estimate the divergence time of each node in the phylogenetic tree of OrthoFinder pipeline results (without *Deinopis* sp.) (Figure 4A). Next, we used CAFE (RRID:SCR_005983) v4.2 [29] under the default parameters to analyze the gene family expansions and contractions of nine spider species.

**9. Identification of Highly-Conserved Elements (HCEs)**

To identify the HCEs, we initially generated pairwise sequence alignments across all nine spider genomes (Figure 4A) with LASTZ (RRID:SCR_018556) v1.04.15 [82] and chainNet [83], using the *P. tepidariorum* genome as the reference. We then used MULTIZ v11.2 [84] to combine the pairwise alignments into multiple sequence alignments. Subsequently, we ran phyloFit in the PHAST package (RRID:SCR_003204) [85] with the topology from OrthoFinder to estimate the neutral ('nonconserved') model based on fourfold degenerate sites. With the nonconserved model as input, we ran phastCons [86]

to estimate conserved models with its intrinsic function and predicted the HCEs.

The distribution of HCEs in exons, introns, 2,000 bp upstream and 2,000 bp downstream of genes and intergenic regions was summarized with Annovar (RRID:SCR_012821) [87, 88] based on the genome annotation information of *O. sinensis* or *P. tepidariorum*.

**10. Homologs of toxin gene family identification and analysis**

Based on ArachnoServer 3.0 [89], a specialized spider venom database, and integrating toxin protein sequences obtained from other toxin research of spiders [37, 38, 90], we have compiled a new reference dataset. This dataset was used to conduct a blastp search for candidate toxin genes (E-value less than 1e-10, matching length greater than 70% of the reference sequence, and hit area mismatch less than 30%). We also established hidden Markov models (HMM) for different types of toxin proteins based on the database and further confirmed the results obtained from blastp using HMMER (RRID:SCR_005305) v 3.3 [91]. To search for hidden toxin-related pseudogenes in *O. sinensis*, we referred to the identification criteria of human genome pseudogenes [41], and used blastn v2.9.0+ and blastx v2.9.0+ to search for candidates.

**11. Physiological index measurement**

Assays were performed as described previously [92]. $CO_2$ production rate was used as a proxy for metabolic rate (MR). The assays were conducted in a closed-circuit system with a volume of 73.3 ml at a temperature of 25 °C, a pressure of 100.6 kPa, and a flow rate of 110 ml/min. MR was calculated as the amount of $CO_2$ produced per gram of body mass per second, using the equation $MR = MCO_2/T/body\ mass$, where $MCO_2$ represents the amount of oxygen substance (in mol). To put spiders into a state of fatigue, we stimulated the spider's legs with a dissecting needle and kept it in a high-intensity state of exercise for 10 minutes.

Enzyme activity was measured using the corresponding reagent kit (Wuhan Mosak Biotechnology Co., Ltd. KT50129, KT50577, KT42310, KT41589, KT87867). Female spider individuals in a resting state were fixed in liquid nitrogen after weighing and stored at -80 °C. Prior to testing, homogenize the samples were homogenized and diluted to 500 μl as the test solution following the supplier’s protocol.

**Additional Files**

**Additional file 1–3:** Hunting video.

**Additional file 4: Table S1.** Genome survey prediction of *Octonoba sinensis*. **Table S2.** Genome assembly. **Table S3.** Repeat sequences of *Octonoba sinensis* genome. **Table S4.** Transcriptome assembly and genomes used in this study. **Table S5.** Genes under positive selection pressure on the node of the family Uloboridae. **Table S6.** Transcriptome differential expression analysis of the legs of *Parasteatoda tepidariorum* and *Octonoba sinensis*. **Table S7.** Genes list of significantly expanded gene families in *Octonoba sinensis*. **Table S8.** New emergent gene family members in the *Octonoba sinensis* genome. **Table S9.** All HCEs, using the *Parasteatoda tepidariorum* genome as a reference. **Table S10.** Missing HCEs in *Octonoba sinensis* and *Uloborus diversus*. **Table S11.** Specific missing orthologous groups in *Octonoba sinensis* and *Uloborus diversus*. **Table S12.** Genes under relaxed selection pressure in Uloboridae. **Table S13.** *Parasteatoda tepidariorum* venom gland-specific expression module. **Table S14.** Quantitative distribution of different toxin homologs across species. **Table S15.** "Pseudogene" blastx. **Table S16.** Protein domain search. **Table S17.** Expression levels (TPM) in different organizations of the genes connected by the red band to the *Parasteatoda tepidariorum* toxin gene in Figure 7h. **Table S18.** The SRA data used for genome annotation. **Table S19.** Clusters of Reciprocal Best Hits (RBH) and RBH between *Octonoba sinensis* and *Parasteatoda tepidariorum*.

**Additional file 5: Figure S1.** Recognizable elements of the *Octonoba sinensis* genome. **Figure S2.** GO enrichment analysis of genes expressed at higher levels (Fold-change $> 4$, $p < 0.05$) in *O. sinensis* legs relative to those in *P. tepidariorum* legs. **Figure S3.** Distribution of all Highly-Conserved Elements (HCEs) in *P. tepidariorum* and missing HCEs and genes in *O. sinensis*. **Figure S4.** Phylogenetic tree of homologs of neurotoxin genes (Latrotoxin).

**Additional file 6:** Dataset of toxin reference genes.

**Acknowledgments**

We are grateful to Prof. Zhonge Hou and Assis. Prof. Fengyuan Li for academic suggestions. We thank Wei Wang at Guangxi Normal University for her suggestions on anatomical techniques. Sincere thanks

to Dr. Nadia Ayoub and Dr. Sandra Correa-Garhwal for their careful review and valuable suggestions on this article. Finally, Y.M.Z. wants to thank Lingling Liu, in particular, for the invaluable support over the years.

**Authors' contributions**

S.Q.L. and Y.M.Z. conceived and designed the project. Y.X.S. and Y.M.Z. finished the genome assembly and annotation. B.Y.Z., P.Y.J., Y.X.S., and Y.M.Z executed the comparison analysis. Y.J.L. and Z.Z. identified the species and provided the spider pictures used in the article. T.Y.J assembled and annotated transcriptome data of *Deinopis* sp. Y.W. recorded the original hunting videos and Y.M.Z. edited the videos. X.T.H. sent a new transcriptome for testing and analysis. All authors participated in the discussion and reviewed the final manuscript.

**Funding**

This study was supported by the Strategic Priority Research Program of the Chinese Academy of Sciences (XDB31000000), and the Program of National Natural Sciences Foundation of China (NSFC–32170447, NSFC–32370490).

**Data Availability**

The sequencing data is deposited in NCBI BioProjects PRJNA1018860 and PRJNA1019401, and ScienceDB [93]. All additional supporting data are available in the *GigaScience* repository, GigaDB [94].

**Declarations**

**Ethics approval and consent to participate**

Not applicable.

**Consent for publication**

Not applicable.

**Competing interests**

The authors declare that they have no competing interests.

# References


1. Foelix RF, Erb B. Mesothelae have venom glands. in: 2010.
2. NMBE - World Spider Catalog. https://wsc.nmbe.ch/. Accessed,2023.
3. Forster RR, Platnick NI. A review of the archaeid spiders and their relatives, with notes on the limits of the superfamily Palpimanoidea (Arachnida, Araneae). Bulletin of the AMNH ; v. 178, article 1. in: 1984.
4. Rix MG. A Review of the Tasmanian Species of Pararchaeidae and Holarchaeidae (Arachnida, Araneae). The Journal of Arachnology 2005;**33**(1):135-152.
5. Opell BD. Revision of the genera and tropical American species of the spider family Uloboridae. Bulletin of the Museum of Comparative Zoology at Harvard College 1979;**148**:443-549.
6. Robinson MH, Olazarri J. Units of behavior and complex sequences in the predatory behavior of Argiope argentata (Fabricius): (Araneae: Araneidae). in: 1971.
7. Weng JL, Barrantes G, Eberhard WG. Feeding by Philoponella vicina (Araneae, Uloboridae) and how uloborid spiders lost their venom glands. Can J Zool 2006;**84**(12):1752-1762. doi:10.1139/z06-149.
8. Eberhard WG, Barrantes G, Weng JL. Tie them up tight: wrapping by Philoponella vicina spiders breaks, compresses and sometimes kills their prey. Sci Nat-Heidelberg 2006;**93**(5):251-4. doi:10.1007/s00114-006-0094-1.
9. Lubin YD. Web buiding and prey capture in the Uloboridae. Spiders:Webs, Behavior, and Evolution. 1986:132-171.
10. Opell BD. The relationship of book lung and tracheal systems in the spider family uloboridae. J Morphol 1990;**206**(2):211-216. doi:10.1002/jmor.1052060207.
11. Opell BD. The respiratory complementarity of spider book lung and tracheal systems. J Morphol 1998;**236**(1):57-64. doi:10.1002/(SICI)1097-4687(199804)236:1<57::AID-JMOR4>3.0.CO;2-L.
12. de Plancy VC. Arachnides recueillis aux environs de Pékin. In: Simon E, ed Annales de la Société Entomologique de France. Saint-Germain; 1880.
13. Rainer F. Biology of Spiders.: Oxford university press; 2011.
14. Wang X, Wang Y, Yang Z, et al. On the Karyotype of Octonnba sinensis. Journal of Hebei Normal University (Natural Science) 1997;(04):423-426.
15. Seppey M, Manni M, Zdobnov EM. BUSCO: Assessing Genome Assembly and Annotation Completeness. Methods Mol Biol 2019;**1962**:227-245. doi:10.1007/978-1-4939-9173-0_14.
16. Simao FA, Waterhouse RM, Ioannidis P, et al. BUSCO: assessing genome assembly and annotation completeness with single-copy orthologs. Bioinformatics 2015;**31**(19):3210-2. doi:10.1093/bioinformatics/btv351.
17. Hu W, Jia A, Ma S, et al. A molecular atlas reveals the tri-sectional spinning mechanism of spider dragline silk. Nat Commun 2023;**14**(1). doi:10.1038/s41467-023-36545-6.
18. Fan Z, Yuan T, Liu P, et al. A chromosome-level genome of the spider Trichonephila antipodiana reveals the genetic basis of its polyphagy and evidence of an ancient whole-genome duplication event. Gigascience 2021;**10**(3). doi:10.1093/gigascience/giab016.
19. Liu S, Aagaard A, Bechsgaard J, et al. DNA Methylation Patterns in the Social Spider, Stegodyphus dumicola. Genes-Basel 2019;**10**(2):137. doi:10.3390/genes10020137.
20. Huerta-Cepas J, Szklarczyk D, Heller D, et al. eggNOG 5.0: a hierarchical, functionally and

phylogenetically annotated orthology resource based on 5090 organisms and 2502 viruses. Nucleic Acids Res 2019;**47**(D1):D309-D314. doi:10.1093/nar/gky1085.

21. Zhu B, Jin P, Zhang Y, et al. Genomic and transcriptomic analyses support a silk gland origin of spider venom glands. Bmc Biol 2023;**21**(1):82. doi:10.1186/s12915-023-01581-7.
22. Miller J, Zimin AV, Gordus A. Chromosome-level genome and the identification of sex chromosomes in Uloborus diversus. Gigascience 2022;**12**. doi:10.1093/gigascience/giad002.
23. Magalhaes I, Azevedo G, Michalik P, et al. The fossil record of spiders revisited: implications for calibrating trees and evidence for a major faunal turnover since the Mesozoic. Biol Rev Camb Philos Soc 2019. doi:10.1111/brv.12559.
24. Shao L, Zhao Z, Li S. Is phenotypic evolution affected by spiders' construction behaviors? Syst Biol 2022. doi:10.1093/sysbio/syac063.
25. Wheeler WC, Coddington JA, Crowley LM, et al. The spider tree of life: phylogeny of Araneae based on target-gene analyses from an extensive taxon sampling. Cladistics 2017;**33**(6):574-616. doi:10.1111/cla.12182.
26. Foth BJ, Goedecke MC, Soldati D. New insights into myosin evolution and classification. Proc Natl Acad Sci U S A 2006;**103**(10):3681-6. doi:10.1073/pnas.0506307103.
27. Squire J. Special Issue: The Actin-Myosin Interaction in Muscle: Background and Overview. Int J Mol Sci 2019;**20**(22). doi:10.3390/ijms20225715.
28. Ayme-Southgate A, Vigoreaux J, Benian G, et al. Drosophila has a twitchin/titin-related gene that appears to encode projectin. Proc Natl Acad Sci U S A 1991;**88**(18):7973-7. doi:10.1073/pnas.88.18.7973.
29. De Bie T, Cristianini N, Demuth JP, et al. CAFE: a computational tool for the study of gene family evolution. Bioinformatics 2006;**22**(10):1269-71. doi:10.1093/bioinformatics/btl097.
30. Matusek T, Djiane A, Jankovics F, et al. The Drosophila formin DAAM regulates the tracheal cuticle pattern through organizing the actin cytoskeleton. Development 2006;**133**(5):957-66. doi:10.1242/dev.02266.
31. Iskratsch T, Ehler E. Formin-g muscle cytoarchitecture. Bioarchitecture 2011;**1**(2):66-68. doi:10.4161/bioa.1.2.15467.
32. Valencia DA, Quinlan ME. Formins. Curr Biol 2021;**31**(10):R517-R522. doi:10.1016/j.cub.2021.02.047.
33. Summers KM, Bush SJ, Davis MR, et al. Fibrillin-1 and asprosin, novel players in metabolic syndrome. Mol Genet Metab 2023;**138**(1):106979. doi:10.1016/j.ymgme.2022.106979.
34. Supek F, Bosnjak M, Skunca N, et al. REVIGO summarizes and visualizes long lists of gene ontology terms. Plos One 2011;**6**(7):e21800. doi:10.1371/journal.pone.0021800.
35. Escuer P, Pisarenco VA, Fernandez-Ruiz AA, et al. The chromosome-scale assembly of the Canary Islands endemic spider Dysdera silvatica (Arachnida, Araneae) sheds light on the origin and genome structure of chemoreceptor gene families in chelicerates. Mol Ecol Resour 2022;**22**(1):375-390. doi:10.1111/1755-0998.13471.
36. Luo J, Ding Y, Peng Z, et al. Molecular diversity and evolutionary trends of cysteine-rich peptides from the venom glands of Chinese spider Heteropoda venatoria. Sci Rep 2021;**11**(1):3211. doi:10.1038/s41598-021-82668-5.
37. Wang Z, Zhu K, Li H, et al. Chromosome-level genome assembly of the black widow spider Latrodectus elegans illuminates composition and evolution of venom and silk proteins. Gigascience 2022;**11**.

doi:10.1093/gigascience/giac049.

38. Zhu B, Jin P, Hou Z, et al. Chromosomal-level genome of a sheet-web spider provides insight into the composition and evolution of venom. Mol Ecol Resour 2022;**22**(6):2333-2348. doi:10.1111/1755-0998.13601.

39. Chen M, Blum D, Engelhard L, et al. Molecular architecture of black widow spider neurotoxins. Nat Commun 2021;**12**(1):6956. doi:10.1038/s41467-021-26562-8.

40. Luddecke T, Herzig V, von Reumont BM, et al. The biology and evolution of spider venoms. Biol Rev Camb Philos Soc 2022;**97**(1):163-178. doi:10.1111/brv.12793.

41. Zhang Z, Carriero N, Zheng D, et al. PseudoPipe: an automated pseudogene identification pipeline. Bioinformatics 2006;**22**(12):1437-9. doi:10.1093/bioinformatics/btl116.

42. Protas ME, Trontelj P, Patel NH. Genetic basis of eye and pigment loss in the cave crustacean, *Asellus aquaticus*. Proceedings of the National Academy of Sciences 2011;**108**(14):5702-5707. doi:doi:10.1073/pnas.1013850108.

43. Gore AV, Tomins KA, Iben J, et al. An epigenetic mechanism for cavefish eye degeneration. Nature Ecology & Evolution. 2018;2(7):1155-1160. doi: 10.1038/s41559-018-0569-4

44. Mojaddidi H, Fernandez FE, Erickson PA, et al. Embryonic origin and genetic basis of cave associated phenotypes in the isopod crustacean Asellus aquaticus. Sci Rep-Uk 2018;**8**(1):16589. doi:10.1038/s41598-018-34405-8.

45. Piatigorsky J. A Genetic Perspective on Eye Evolution: Gene Sharing, Convergence and Parallelism. Evolution: Education and Outreach 2008;**1**(4):403-414. doi:10.1007/s12052-008-0077-0.

46. Krishnan J, Rohner N. Cavefish and the basis for eye loss. Philos Trans R Soc Lond B Biol Sci 2017;**372**(1713). doi:10.1098/rstb.2015.0487.

47. Bolger AM, Lohse M, Usadel B. Trimmomatic: a flexible trimmer for Illumina sequence data. Bioinformatics 2014;**30**(15):2114-20. doi:10.1093/bioinformatics/btu170.

48. Wenger AM, Peluso P, Rowell WJ, et al. Accurate circular consensus long-read sequencing improves variant detection and assembly of a human genome. Nat Biotechnol 2019;**37**(10):1155-1162. doi:10.1038/s41587-019-0217-9.

49. Lu L, Liu X, Huang W, et al. Robust Hi-C Maps of Enhancer-Promoter Interactions Reveal the Function of Non-coding Genome in Neural Development and Diseases. Mol Cell 2020;**79**(3):521-534.e15. doi:10.1016/j.molcel.2020.06.007.

50. Rao SS, Huntley MH, Durand NC, et al. A 3D map of the human genome at kilobase resolution reveals principles of chromatin looping. Cell 2014;**159**(7):1665-80. doi:10.1016/j.cell.2014.11.021.

51. Meyer M, Kircher M. Illumina sequencing library preparation for highly multiplexed target capture and sequencing. Cold Spring Harb Protoc 2010;**2010**(6):pdb.prot5448. doi:10.1101/pdb.prot5448.

52. Durand NC, Shamim MS, Machol I, et al. Juicer Provides a One-Click System for Analyzing Loop-Resolution Hi-C Experiments. Cell Syst 2016;**3**(1):95-8. doi:10.1016/j.cels.2016.07.002.

53. Marcais G, Kingsford C. A fast, lock-free approach for efficient parallel counting of occurrences of k-mers. Bioinformatics 2011;**27**(6):764-70. doi:10.1093/bioinformatics/btr011.

54. Cheng H, Concepcion GT, Feng X, et al. Haplotype-resolved de novo assembly using phased assembly graphs with hifiasm. Nat Methods 2021;**18**(2):170-175. doi:10.1038/s41592-020-01056-5.

55. Ruan J, Li H. Fast and accurate long-read assembly with wtdbg2. Nat Methods 2020;**17**(2):155-158.

doi:10.1038/s41592-019-0669-3.
56. Hu J, Fan J, Sun Z, et al. NextPolish: a fast and efficient genome polishing tool for long-read assembly. Bioinformatics 2020;**36**(7):2253-2255. doi:10.1093/bioinformatics/btz891.
57. Li H. Minimap2: pairwise alignment for nucleotide sequences. Bioinformatics 2018;**34**(18):3094-3100. doi:10.1093/bioinformatics/bty191.
58. Danecek P, Bonfield JK, Liddle J, et al. Twelve years of SAMtools and BCFtools. Gigascience. 2021,10(2):giab008. doi: 10.1093/gigascience/giab008.
59. Dudchenko O, Batra SS, Omer AD, et al. De novo assembly of the Aedes aegypti genome using Hi-C yields chromosome-length scaffolds. Science 2017;**356**(6333):92-95. doi:10.1126/science.aal3327.
60. Dudchenko O, Shamim MS, Batra S, et al. The Juicebox Assembly Tools module facilitates de novo assembly of mammalian genomes with chromosome-length scaffolds for under $1000. Cold Spring Harbor: Cold Spring Harbor Laboratory Press; 2018.
61. Kim D, Paggi JM, Park C, et al. Graph-based genome alignment and genotyping with HISAT2 and HISAT-genotype. Nat Biotechnol 2019;**37**(8):907-915. doi:10.1038/s41587-019-0201-4.
62. Anders S, Pyl PT, Huber W. HTSeq--a Python framework to work with high-throughput sequencing data. Bioinformatics 2015;**31**(2):166-9. doi:10.1093/bioinformatics/btu638.
63. Ritchie ME, Phipson B, Wu D, et al. limma powers differential expression analyses for RNA-sequencing and microarray studies. Nucleic Acids Res 2015;**43**(7):e47. doi:10.1093/nar/gkv007.
64. Flynn JM, Hubley R, Goubert C, et al. RepeatModeler2 for automated genomic discovery of transposable element families. Proc Natl Acad Sci U S A 2020;**117**(17):9451-9457. doi:10.1073/pnas.1921046117.
65. Tarailo-Graovac M, Chen N. Using RepeatMasker to identify repetitive elements in genomic sequences. Curr Protoc Bioinformatics 2009;**Chapter 4**:4.10.1-4.10.14. doi:10.1002/0471250953.bi0410s25.
66. Bruna T, Hoff KJ, Lomsadze A, et al. BRAKER2: automatic eukaryotic genome annotation with GeneMark-EP+ and AUGUSTUS supported by a protein database. NAR Genom Bioinform 2021;**3**(1):lqaa108. doi:10.1093/nargab/lqaa108.
67. Chan PP, Lin BY, Mak AJ, et al. tRNAscan-SE 2.0: improved detection and functional classification of transfer RNA genes. Nucleic Acids Res 2021;**49**(16):9077-9096. doi:10.1093/nar/gkab688.
68. Kalvari I, Nawrocki EP, Ontiveros-Palacios N, et al. Rfam 14: expanded coverage of metagenomic, viral and microRNA families. Nucleic Acids Res 2021;**49**(D1):D192-D200. doi:10.1093/nar/gkaa1047.
69. Nawrocki EP, Eddy SR. Infernal 1.1: 100-fold faster RNA homology searches. Bioinformatics 2013;**29**(22):2933-5. doi:10.1093/bioinformatics/btt509.
70. Yu N, Li J, Bao H, et al. Chromosome-level genome of spider Pardosa pseudoannulata and cuticle protein genes in environmental stresses. Sci Data 2024;**11**(1):121. doi:10.1038/s41597-024-02966-1.
71. Chen S, Zhou Y, Chen Y, et al. fastp: an ultra-fast all-in-one FASTQ preprocessor. Bioinformatics 2018;**34**(17):i884-i890. doi:10.1093/bioinformatics/bty560.
72. Grabherr MG, Haas BJ, Yassour M, et al. Full-length transcriptome assembly from RNA-Seq data without a reference genome. Nat Biotechnol 2011;**29**(7):644-52. doi:10.1038/nbt.1883.
73. Li W, Godzik A. Cd-hit: a fast program for clustering and comparing large sets of protein or nucleotide sequences. Bioinformatics 2006;**22**(13):1658-9. doi:10.1093/bioinformatics/btl158.
74. Emms DM, Kelly S. OrthoFinder: phylogenetic orthology inference for comparative genomics. Genome Biol 2019;**20**(1):238. doi:10.1186/s13059-019-1832-y.

75. Katoh K, Standley DM. MAFFT multiple sequence alignment software version 7: improvements in performance and usability. Mol Biol Evol 2013;**30**(4):772-80. doi:10.1093/molbev/mst010.
76. Camacho C, Coulouris G, Avagyan V, et al. BLAST+: architecture and applications. Bmc Bioinformatics 2009;**10**:421. doi:10.1186/1471-2105-10-421.
77. Minh BQ, Schmidt HA, Chernomor O, et al. IQ-TREE 2: New Models and Efficient Methods for Phylogenetic Inference in the Genomic Era. Mol Biol Evol 2020;**37**(5):1530-1534. doi:10.1093/molbev/msaa015.
78. Fan Z, Wang LY, Xiao L, et al. Lampshade web spider Ectatosticta davidi chromosome-level genome assembly provides evidence for its phylogenetic position. Commun Biol 2023;**6**(1):748. doi:10.1038/s42003-023-05129-x.
79. Tang H, Bowers JE, Wang X, et al. Synteny and collinearity in plant genomes. Science 2008;**320**(5875):486-8. doi:10.1126/science.1153917.
80. Yang Z. PAML 4: phylogenetic analysis by maximum likelihood. Mol Biol Evol 2007;**24**(8):1586-91. doi:10.1093/molbev/msm088.
81. Kosakovsky PS, Poon A, Velazquez R, et al. HyPhy 2.5-A Customizable Platform for Evolutionary Hypothesis Testing Using Phylogenies. Mol Biol Evol 2020;**37**(1):295-299. doi:10.1093/molbev/msz197.
82. Harris RS. Improved pairwise alignment of genomic DNA. Doctor of Philosophy, The Pennsylvania State University, 2007.
83. Kent WJ, Baertsch R, Hinrichs A, et al. Evolution's cauldron: duplication, deletion, and rearrangement in the mouse and human genomes. Proc Natl Acad Sci U S A 2003;**100**(20):11484-9. doi:10.1073/pnas.1932072100.
84. Blanchette M, Kent WJ, Riemer C, et al. Aligning multiple genomic sequences with the threaded blockset aligner. Genome Res 2004;**14**(4):708-15. doi:10.1101/gr.1933104.
85. Hubisz MJ, Pollard KS, Siepel A. PHAST and RPHAST: phylogenetic analysis with space/time models. Brief Bioinform 2011;**12**(1):41-51. doi:10.1093/bib/bbq072.
86. Siepel A, Bejerano G, Pedersen JS, et al. Evolutionarily conserved elements in vertebrate, insect, worm, and yeast genomes. Genome Res 2005;**15**(8):1034-50. doi:10.1101/gr.3715005.
87. Yang H, Wang K. Genomic variant annotation and prioritization with ANNOVAR and wANNOVAR. Nat Protoc 2015;**10**(10):1556-66. doi:10.1038/nprot.2015.105.
88. Wang K, Li M, Hakonarson H. ANNOVAR: functional annotation of genetic variants from high-throughput sequencing data. Nucleic Acids Res 2010;**38**(16):e164. doi:10.1093/nar/gkq603.
89. Pineda SS, Chaumeil PA, Kunert A, et al. ArachnoServer 3.0: an online resource for automated discovery, analysis and annotation of spider toxins. Bioinformatics 2018;**34**(6):1074-1076. doi:10.1093/bioinformatics/btx661.
90. Haney RA, Matte T, Forsyth FS, et al. Alternative Transcription at Venom Genes and Its Role as a Complementary Mechanism for the Generation of Venom Complexity in the Common House Spider. Front Ecol Evol 2019;**7**. doi:10.3389/fevo.2019.00085.
91. Mistry J, Finn RD, Eddy SR, et al. Challenges in homology search: HMMER3 and convergent evolution of coiled-coil regions. Nucleic Acids Res 2013;**41**(12):e121. doi:10.1093/nar/gkt263.
92. Roberts SP, Harrison JF, Dudley R. Allometry of kinematics and energetics in carpenter bees (Xylocopa varipuncta) hovering in variable-density gases. J Exp Biol 2004;**207**(Pt 6):993-1004.

doi:10.1242/jeb.00850.
93. Zhang Y; Li S. Genome of Octonoba sinensis. ScienceDB. 2024. doi.org/10.57760/sciencedb.09166.
94. Zhang Y; Shen Y; Jin P, et al. Supporting data for "A Trade-off in Evolution: The Adaptive Landscape of Spiders without Venom Glands" GigaScience Database. 2024. https://doi.org/10.5524/102549

Figure 1

Click here to access/download;Figure;Fig.1-giga-re.jpg

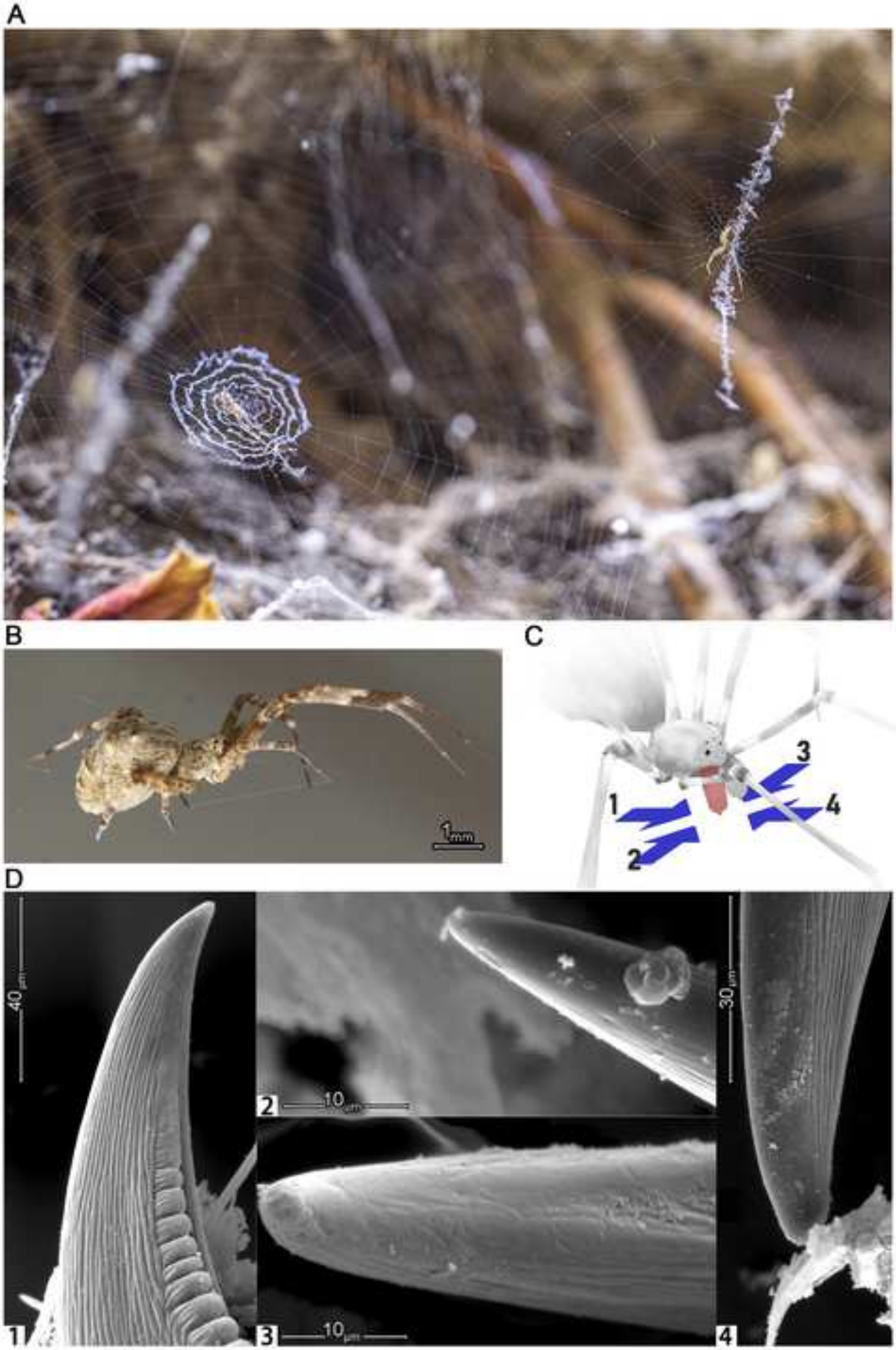

Figure 2

Click here to access/download;Figure;Fig.2-giga-re.jpg

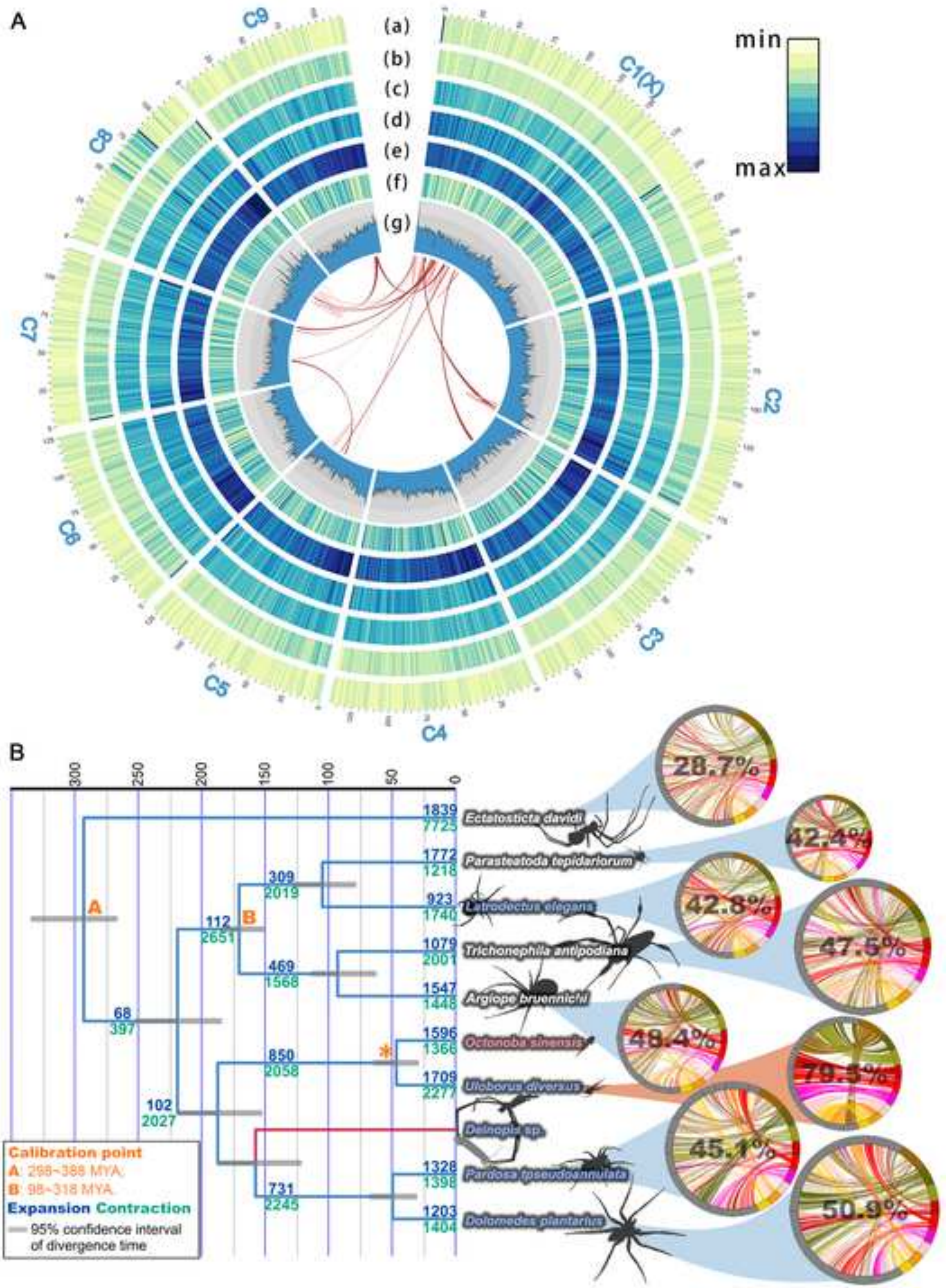

Figure 3

Click here to access/download;Figure;Fig.3-giga-re.jpg

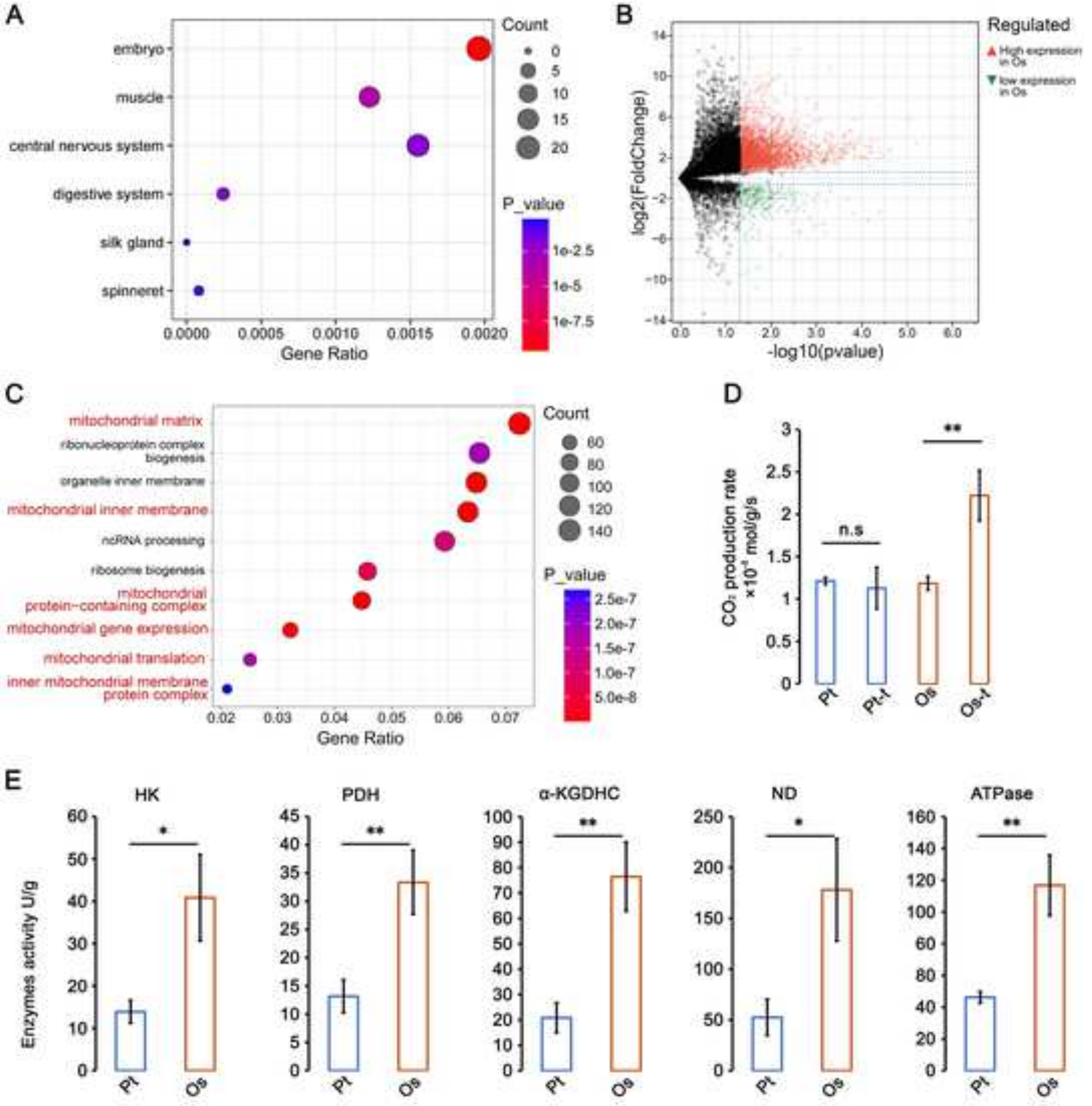

Figure 4

Click here to access/download;Figure;Fig.4-giga-re.jpg

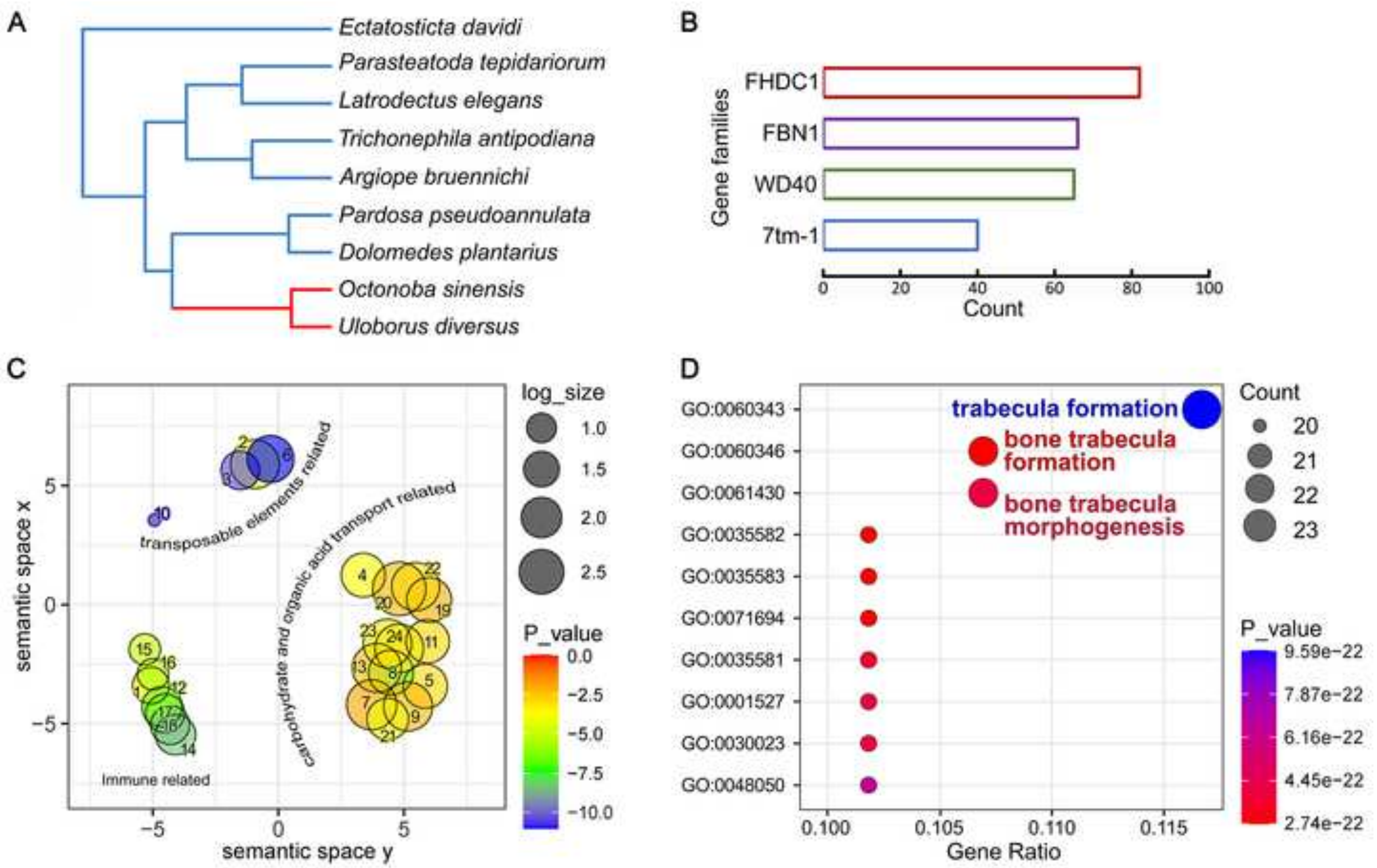

Figure 5

Click here to access/download;Figure;Fig.5-giga-re.jpg

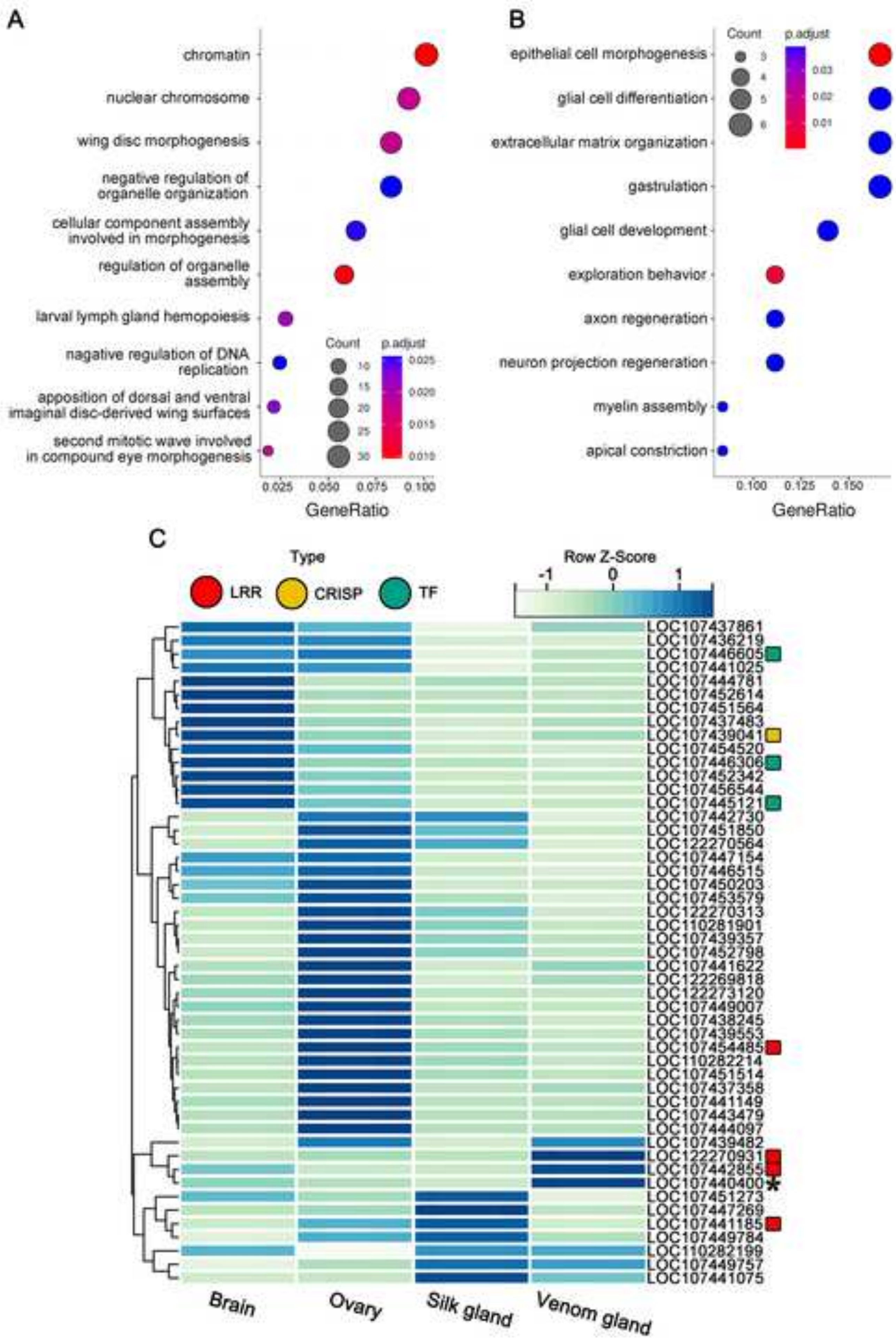

Figure 6 Click here to access/download;Figure;Fig.6-giga-re.jpg

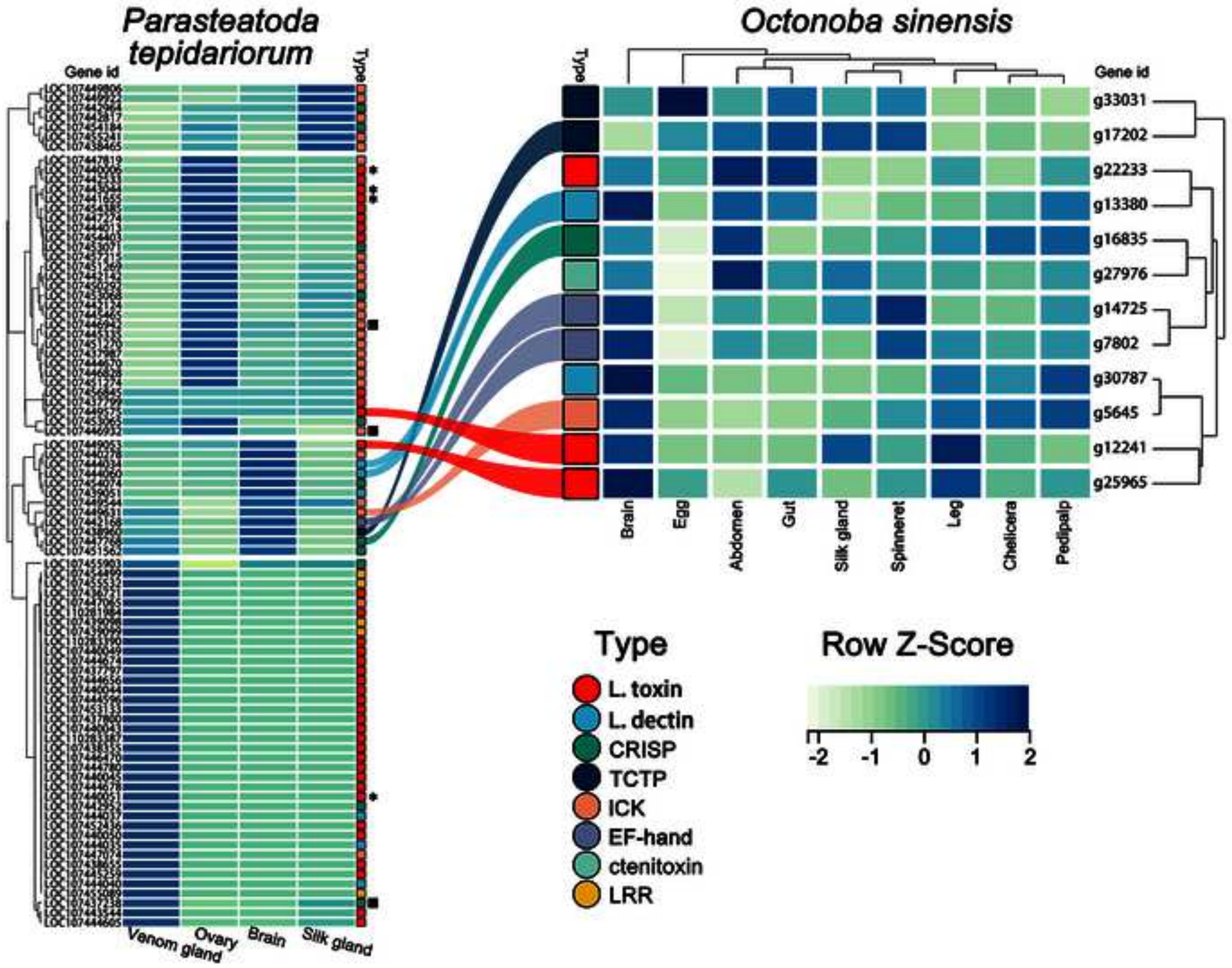

Figure 7

Click here to access/download;Figure;Fig.7-giga-re.jpg

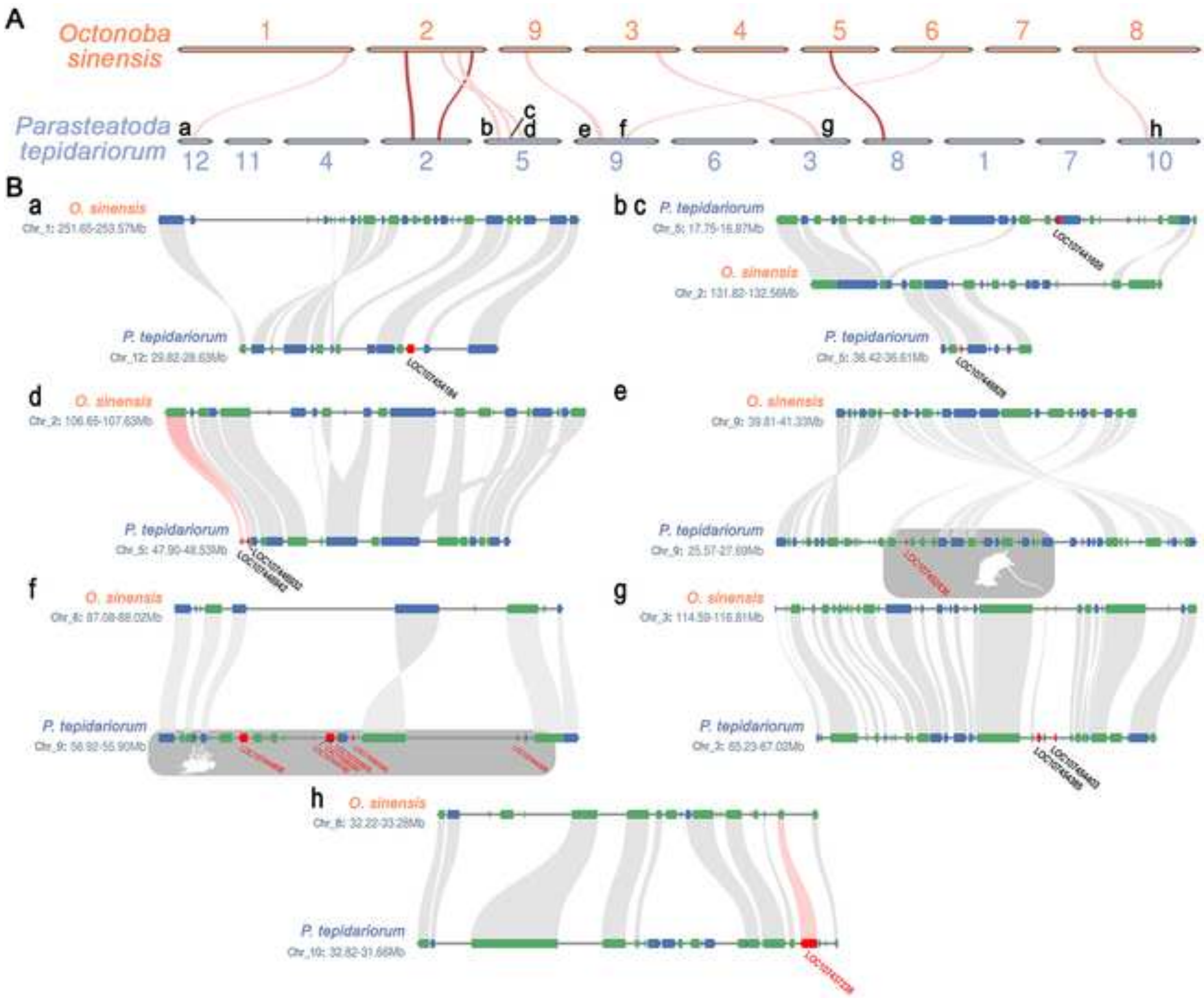

Additional file 1

Click here to access/download
**Supplementary Material**
Additional file 1.mp4

Additional file 2

Click here to access/download
**Supplementary Material**
Additional file 2.mp4

Additional file 3

Click here to access/download

**Supplementary Material**

Additional file 3.mp4

Additional file 4

Click here to access/download
**Supplementary Material**
Additional file 4-giga.xlsx

Additional file 5

Click here to access/download
**Supplementary Material**
Additional file 5-giga.docx

Additional file 6

Click here to access/download
**Supplementary Material**
Additional file 6.fa

Response to reviewers R3
Click here to access/download;Personal Cover;Response to reviewers R3.docx

# Response to reviewers

Dear Editor Zhang

I hope this email finds you well. We have carefully considered all the feedback and have made the necessary revisions to our paper.

We would like to emphasize that the entire review process has been an incredibly enriching experience for us. The insights and suggestions from the reviewers and editor have not only strengthened our research but have also broadened our understanding of the field. We are grateful for the time and effort the reviewers have invested in providing us with such valuable feedback.

Thank you.

Best regards,

Yiming Zhang; Shuqiang Li

Institute of Zoology, Chinese Academy of Sciences

1. Beichen West Road, Chaoyang District

Beijing 100101, P. R. China

Tel: +86-13363636950

Fax: +86-10-64807216

Email: zhangyiming@ioz.ac.cn; lisq@ioz.ac.cn

Editor comments

Please include a point-by-point within the 'Response to Reviewers' box in the submission system. Please ensure you describe additional experiments that were carried out and include a detailed rebuttal of any criticisms or requested revisions that you disagreed with. Please remember to make revisions per Dr. Sandra Correa-Garhwal's comments 34-48 in the second round of review.

>>>**Response:** Thank you for giving us the opportunity to supplement these responses. We have responded to each point individually.

34. Figure S4: The black text in the figure should be added the figure legend. For the green genes, add the species they come from.

>>>**Response:** Thank you for your comments. We have moved the **black text** from the figure to the figure legend and listed the species sources of the reference toxin genes (marked with green color).

"Figure S4: Phylogenetic tree of neurotoxin genes (Latrotoxin). Tips labelled in red font represent *Octonoba sinensis* genes, those labelled in green font represent the reference sequences of different Latrotoxins genes previously studied, others represent *Parasteatoda tepidariorum* genes. Tips labelled in green font come from different species, *Latrodectus tredecimguttatus*: sp|Q25338|LITD_LATTR, sp|P23631|LATA_LATTR, sp|Q02989|LITA_LATTR, sp|Q9XZC0|LCTA_LATTR; *Steatoda grossa*: sp|L7X8P2|LATA_STEGR; *Latrodectus geometricus*: sp|L7XCU0|LATA_LATGE; *Latrodectus h esperus*: sp|P0DJE3|LATA_LATHE; *Latrodectus pallidus*: sp|L7XDS4|LATA_LATPL; *Latrodectus hasselti*: sp|G0LXV8|LATA_LATHA. The red font with black stroke is a similar object below the recognition threshold as an outlier."

35. Lines 281-290: I think is very interesting that there is conservation in the where these genes are in both species regardless of function. The paragraph will benefit from editing because the results are not coming thru. I also suggest that the authors describe all genes that fall under this category, I think that there are only three by looking at Figure 6. Given the small number of instances, it seems odd to only describe one of them. These results should also be added to the discussion: we found these genes, they are expressed (or not)

in XX tissue, and we think the function in octonoba is XX.

>>>**Response:** It is true that there are three pairs of toxin homologs preserved in the syntenic region, but only one of them was highly expressed in the venom glands of *P. tepidariorum*, which we consider a relatively reliable toxin gene in *P. tepidariorum*. The others showed their highest expression in other tissues of *P. tepidariorum* (Figure 6) and may be performing non-toxic functions, so we didn't have a detailed discussion on the two genes mentioned later.

In the revised manuscript, we mentioned the above content and added some discussion at this paragraph.

"By searching for toxin gene homologs in collinearity fragments of *O. sinensis* and *P. tepidariorum*, a particular class of genes was found in *O. sinensis*. These genes are located in the same place as the *P. tepidariorum* toxin gene homologs in the collinearity segment, but they can no longer be identified as toxin genes (below the minimum recognition threshold, see methods) (Figure 7B, red ribbon). There are three pairs of such genes, includes one pair of CRISP genes (g31478~LOC107437238) and two pairs of ICK genes (g6736~LOC107446942 and g6736~LOC107446932). Their expression patterns in *P. tepidariorum* indicate that only the CRISP gene is a reliable toxin gene (Figure 6, black squares). In *O. sinensis*, this CRISP gene (g31478) cannot be unambiguously classified as a homolog of toxin genes due to changes in protein structure (Additional file 4: Table S16). However, compared with other toxin genes which it is difficult to find pseudogenes, the ortholog of this CRISP gene in *O. sinensis* have complete gene structures, and can be expressed in multiple tissues of a venomless spider (Additional file 4: Table S17). These all indicate that this CRISP gene (g31478) must play the role of a non-toxic gene. We believe that this observation suggests a potential functional shift between toxic and non-toxic genes in spiders." (**see lines 292–305**)

We also mentioned relevant content in the **Discussion** section of the article.

"Multi-omics analysis revealed the absence or possible functional shift of toxin genes in *O. sinensis*." (**see lines 330–331**)

"Simultaneously, through analyzing expression patterns and synteny relationships, it was

discovered that toxin genes in venomous spiders are expressed in other tissues of uloborids and may perform non-toxic functions. This observation suggests a potential functional shift between toxic and non-toxic genes in spiders. In addition to toxin genes, the absence of certain genes in uloborids raises concerns. These include protein c-ets-2, CC2D, and …" (**see lines 357–361**)

36. Figure 6 The legend that is inside the figure, starting with the asterisk should be included in the legend and maybe not in the figure. It also refers to itself which is odd)

>>>**Response:** Thank you for your comments. We have moved the corresponding information from the figure to the figure legend.

37. Figure 7. What the red line in A means needs to be included in the legend for panel A. Also, include that the different colors (green vs. blue) in the genes for panel B indicate.

>>>**Response:** Thank you for your comments. The "**red line**" indicates that the original toxin homolog has been retained in the collinearity. The genes with different colors in the **Figure 7B** represent different transcription directions. We have added relevant information in the figure legend.

38. Line 300: add () to A

>>>**Response:** Thank you, we have made the correction.

39. Line 301: add () to A

>>>**Response:** Thank you, we have made the correction.

40. For the methods, include the type of camera that was used to film the pre-wrapping behavior.

>>>**Response:** Thank you for your comments. We have supplemented this information. The text is taken as follows:

"We recorded a series of videos to observe the predation behavior, using Logitech StreamCam 960-001282." **(lines 386–387)**

41. Line 393 Additional file 6 has no information related to tissue samples

>>>**Response:** Thank you for your comments. The tissue sample information involved in **Additional file 6** is in the **Section 5** of **Methods (see lines 428–431)**. The purpose of **Additional file 6** is to demonstrate two genes (Figure 7B, genes linked with red ribbon) in the *O. sinensis* have indeed been expressed. We have removed other genes in the new modification, leaving only the two required genes. We have renamed the table as **Table S17** and placed it in **Additional file 4**.

42. Line 400: replace "have been" with were

>>>**Response:** Thank you, we have made the correction.

43. Line 406 spell out 9. Add "of" after Gb

>>>**Response:** Thank you, we have made the correction.

44. Section 7 - was this methodology used for figure s4? How was that tree generated?

>>>**Response:** Thank you, the phylogenetic tree of latrotoxin homologs (Figure S4) was reconstructed using the neighbor-joining method, following alignment of the full-length protein sequences via Mafft. We have supplemented this information in this **Section**.

45. How were the cluster analyses done?

>>>**Response:** Thank you for your comments. One-to-one ortholog identification among ten species (Figure 2B) was performed using the RBH method by blastp. *O. sinensis* was used as a reference species. Finally, 5,848 RBH clusters (Additional file 4: Table S19) were retained for analysis. We have supplemented this information. **(lines 484–486)**

46. Line 456 spell out HCEs

>>>**Response:** Thank you, we have made the correction. (**see line 502)**

47. Line 468: instead of "previous databases" explain what those are. Downloaded sequences from multiple sources (NCBI/Arachnoserver?) that includes multiple species? Or includes venom components from all spiders and other animals?

>>>**Response:** Thank you, we have supplemented this information. This information comes from previous studies, and we have listed the articles published in these studies as references and added a brief description in the manuscript.

“Based on ArachnoServer 3.0 [89], a specialized spider venom database, and integrating toxin protein sequences obtained from other toxin research of spiders [37, 38, 90], we have compiled a new reference dataset.” **(see lines 514–516)**

48. Availability of data: the NCBI code given is not functional and there is an empty parenthesis for the gigaDB Digital Repository.

>>>**Response:** Thanks for your suggestion, the relevant data has been uploaded to the FTP server provided by GigaDB and will be made public after the article is published. At that time, there will be a valid link filled in the current parentheses. At this stage, you can obtain the corresponding information through the FTP link.

We have provided a new available BioProject Accession number (PRJNA1018860)

In addition, we have added a public location for sequencing data in SRR, which is ScienceDB (doi.org/10.57760/sciencedb.09166).

Reviewer #2:

I believe the manuscript has shown improvement since the last revision and has effectively addressed most of my concerns and comments. However, I think the discussion section still requires further refinement to convey the main message clearly. For instance, consolidating the concluding remarks into the final paragraph and keeping all conclusions within that paragraph would enhance coherence.

>>>**Response:** I would like to express my sincere gratitude for your assistance and support throughout the entire process. We have learned a great deal from your feedback and suggestions. Thank you for your valuable contributions.

We have consolidated all concluding remarks into the last paragraph of the discussion.

"For predators, there exists a pervasive evolutionary trade-off between chemical and physical attack strategies. Previous research has frequently favored the exploration of chemical strategies, particularly venom. However, against the backdrop of nearly all spiders being toxic predators, our study delves into the genetic basis underlying the alternative choice in this trade-off. Unsurprisingly, reliable toxin gene was not identified in Uloboridae, but these adaptive evolutions ranging from muscle to aerobic respiration and then to supply of energy substances, provide strong support for the exceptional physical endurance demands of this group, and compensate for their decreased adaptability due to the absence of venom glands. Furthermore, some development-related gene and element deletions were observed in uloborids. Although the association between these deletions and the absence of spider venom glands remains unclear, they still hold potential for exploring the evolutionary mechanisms underlying this phenomenon."

**(see lines 366–376)**

Please ensure that numbers under ten are spelled out. Additionally, after "protocol" in line 520, add a period. Include the number for the GigaDB Digital Repository. Furthermore, review the references for format consistency. For example, Reference 50 has the year in bold instead of the issue, and Reference 79 is written in all caps.

>>>**Response:** Thank you for your comments. We searched the entire text and spelled out the numbers under ten in the manuscript. Except for some special positions, such as

such as “k > 1” in **line 496**

There is a period missing at the end of the main text (after "protocol"). Thank you for your reminder. We have made the necessary changes here.

For the access address of GigaDB, currently there is only an FTP server connection method available for reviewers to access. I have communicated with the GigaDB office and they said that the corresponding link will be provided when the article is published.

We have thoroughly organized the references. The reference 50 you mentioned has the same volume number as the publication year, so the reference management APP automatically generate it in bold **(see reference 51)**. Reference 79, we have made the correction. **(see reference 82)**.
