# Supplementary material for: A trade-off in evolution: the adaptive landscape of spiders without venom glands
Source: Gigascience. 2024 Aug 5;13:giae048. doi: 10.1093/gigascience/giae048 (PMC11299198; doi:10.1093/gigascience/giae048)
Supplement: giae048_GIGA-D-23-00275_Revision_1 [file giae048_giga-d-23-00275_revision_1.pdf]

GigaScience

A Trade-off in Evolution: The Adaptive Landscape of Spiders without Venom Glands

--Manuscript Draft--

**Manuscript Number:**	GIGA-D-23-00275R1
**Full Title:**	A Trade-off in Evolution: The Adaptive Landscape of Spiders without Venom Glands
**Article Type:**	Research
**Funding Information:**	Strategic Priority Research Program of Chinese Academy of Sciences (XDB31000000) — professor Shuqiang Li
**Abstract:**	Background: Venom glands play a key role in the predation and defense strategies of almost all spider groups. However, the spider family Uloboridae lacks venom glands and has evolved an adaptive strategy: They excessively wrap their prey directly with spider silk instead of paralyzing it first with toxins. This shift of survival strategy is very fascinating, but the genetic underpinnings behind it are poorly understood.<br>Results: Spanning multiple spider groups, we conducted multi-omics analyses on Octonoba sinensis, and described the adaptive evolution of the Uloboridae family at the genome level. We observed the coding genes of myosin and twitchin in muscles are under positive selection, energy metabolism functions are enhanced, and gene families related to tracheal development and tissue mechanical strength are expanded or emerged, all of which are related to the unique anatomical structure and predatory behavior of spiders in the family Uloboridae. In addition, we also scanned the elements which are absent or under relaxed purifying selection, as well as toxin gene homologs in the genomes of two species in this family. The results show that the absence of regions and regions under relaxed selection in these spiders' genomes are concentrated in areas related to development and neuro-system. The search for toxin homologs also confirms that there are no toxin coding genes available for hunting in the genome of this group.<br>Conclusions: This study demonstrates the trade-off between different predation strategies in spiders, either using venom or physical energy and provides insights into the possible mechanism underlying this trade-off. Venomless spiders need to mobilize multiple developmental and metabolic pathways related to motor function and limb mechanical strength to cover the decline in adaptability caused by the absence of venom glands.
**Corresponding Author:**	Shuqiang Li, Ph.D.<br>Institute of Zoology Chinese Academy of Sciences<br>Beijing, CHINA
**Corresponding Author Secondary Information:**	
**Corresponding Author's Institution:**	Institute of Zoology Chinese Academy of Sciences
**Corresponding Author's Secondary Institution:**	
**First Author:**	Yiming Zhang
**First Author Secondary Information:**	
**Order of Authors:**	Yiming Zhang
	Yunxiao Shen
	Bingyue Zhu
	Pengyu Jin
	Yejie Lin
	Tongyao Jiang
	Xianting Huang
	Yang Wang

Powered by Editorial Manager® and ProduXion Manager® from Aries Systems Corporation

	Shuqiang Li, Ph.D.
**Order of Authors Secondary Information:**	
**Response to Reviewers:**	Response to reviewers<br>Dear Zhang<br>Thanks very much for handling our manuscript (GIGA-D-23-00275) submitted to GigaScience. We greatly appreciate the profound and constructive comments from you and the two reviewers, which are very helpful to improve our manuscript. We have been able to incorporate changes to reflect the suggestions provided by the reviewers. all he changes are highlighted within the manuscript. We believed that we have addressed all the concerns from the reviewers. A point-by-point response to reviewers' comments is enclosed.<br><br>Sincerely,<br>Yiming Zhang; Shuqiang Li<br>Institute of Zoology, Chinese Academy of Sciences<br>1. Beichen West Road, Chaoyang District<br>Beijing 100101, P. R. China<br>Tel: +86-10-64807216<br>Fax: +86-10-64807216<br>Email: zhangyiming@ioz.ac.cn; lisq@ioz.ac.cn<br><br><br>Editor comments<br>As the reviewer mentioned that the genome of Uloborus diversus should be included in the manuscript.<br>>>>Response: Thanks for your suggestions. We added this genome to the new analysis and supplemented the annotation, increasing the number of coding genes with transcriptome support from 15750 to 19036. Subsequently, we also added some genomes proposed by reviewers to the new analysis and conducted all downstream analyses based on these data.<br><br><br><br>Reviewer #1:<br>Zhang et al. provide a chromosome level assembly of Octonoba sinensis, a member of the one and only family of spiders that does not possess venom glands. The sequence and annotation is highly valuable for arachnologists and arthropod biologists more generally. The manuscript also provides some interesting analyses, focusing on potential gene loss associated with loss of venom glands. However, I have concerns that need to be addressed prior to publication.<br>>>>Response: Thank you for your positive assessment of our work. In this revision, we have strengthened the exploration of the trade-offs between motor function and the absence of venom glands. In addition to some highlights in the original manuscript, new discoveries have also emerged under a new comparative system. These results provide a more comprehensive characterization of the adaptive evolution of this group under the absence of venom glands.<br>1. First, the closest relative included in the gene family analyses, Stegodyphus dumicola (Eresidae), is quite distantly related from Uloboridae. Thus, inferences about gene loss or positive selection associated with the loss of venom glands are quite difficult to make with confidence. At a minimum, the authors need to clarify the lack of inclusion of any other members of the UDOH grade. As written (lines 180-183), the authors insinuate that these close relatives are included in the analysis of gene loss and positive/relaxed selection. This is not true. Although, there are no genomes currently available for members of Deinopidae, Oecobiidae, Hersiliidae, there are transcriptomes. The authors could consider an additional analysis using these transcriptomes to track gene loss.<br>>>>Response: Thanks for your suggestions. Based on your suggestion, we assembled the transcriptome of an Ogre-faced spider (Deinopis sp.), and obtained a total protein Busco score of 93.1% (see Fig. 2B, 3A and Methods: 6. Genome annotation). We used this portion of data to enrich selection pressure analysis and assist in searching for specific gene deletions and emergence.<br>2. Related to the above comment, a high quality chromosome-level assembly is available for Uloborus diversus. I strongly recommend including Uloborus in synteny

and gene family analyses. As another uloborid species, its inclusion would bolster inferences about gene loss in Uloboridae or positive selection/relaxed selection in Uloboridae, and not just in Octonoba.
>>>Response: Thanks for your comments. We added this genome to the new analysis (see Fig. 2B, 3A) and supplemented the annotation, increasing the number of coding genes with transcriptome support from 15750 to 19036. Subsequently, we also added some genomes proposed by reviewers to the new analysis and conducted all downstream analyses based on these data.
3. Related to above, there are three genomes available for RTA clade species in NCBI. It doesn't look like there are associated publications yet, but the authors might consider inclusion to break up the evolutionary distance between Uloboridae and other species currently in the analysis; if the authors are set on only using species with whole genomes.
>>>Response: Based on your suggestion, we have annotated the Pardosa pseudoannulata and Dolomedes plantarius genomes, and the total protein Busco scores reached 93.6% and 95.0%, respectively. In addition, we replaced the Dysdera silvatica genome with the newly published Ectatosticta davidi genome as the basal group, and re-annotated the Latrodectus elegans genome, resulting in an increase in its protein Busco score from 63.7% to 96.5%. In the end, all of these genomes have a protein Busco score of over 90% (Additional file 4: Table S2), and a more suitable evolutionary tree was obtained (see Fig. 2B, 3A).
4. For positive selection analyses, is finding metabolic genes unusual? E.g. if the same analysis was done moving a species with venom glands to the target would metabolic genes still arise? Perhaps a different set of genes, but could be something more general to arachnids/arthropods?
>>>Response: Based on your suggestion, using the new dataset mentioned above, we conducted a new positive selection analysis using the nodes of the Uloboridae family, where the O. sinensis and U. diversus genomes are located, as the foreground branch. Unfortunately, the new significant positive selection gene set does not support enrichment in energy metabolism pathways (see Results: 4. Genes under positive selection and energy metabolism in muscle). However, in comparative transcriptome analysis of legs of different species, genes highly expressed in the O. sinensis can be significantly enriched in mitochondrial related GO entries (Fig. 3C).
5. The relevance of the "absent" regions and regions under relaxed selection wasn't clear. Was there a specific hypothesis being tested? E.g. that toxin genes are more likely to be lost or under relaxed selection? Clarify.
>>>Response: Thanks for your comments. We speculate that due to the absence of venom glands, the genes or functional regions specifically involved in the venom gland system in the Uloboridae family may be subjected to relaxed purifying selection or gradually lost from genome. In order to obtain this information, Highly-Conserved Elements (HCEs), absent genes and genes under selective relaxation in the O. sinensis and U. diversus genome were searched and identified. We have added the relevant description to the "Results: 6. Absent regions and genes under relaxed purifying selection" to make it clearer.
6. Similarly, the point of the SNP analysis wasn't clear. Was there a driving hypothesis? What is learned from the analysis?
>>>Response: Thanks for your comments. The distribution of SNPs may reflect information from recent evolutionary processes or divergence characteristics between closely related lineage. It may not properly reflect the evolution information on a larger time scale (such as in this study) (doi: 10.1093/gbe/evz128). Therefore, we have deleted this section in the revised manuscript.
7. How were venom specific modules identified in Parasteatoda? If the authors generated their own RNA seq for Parasteatoda, please include accessions.
>>>Response: Thank you for pointing this out. This “venom specific modules” was the result of our previous work (doi: 10.1186/s12915-023-01581-7). It was obtained through weighted correlation network analysis using transcriptome data from different tissues, and the relevant results have been published. Here, we directly cite this result (see reference 29).
In the revised manuscript, we have generated transcriptome data for legs from Parasteatoda, while remaining transcriptome data of the Parasteatoda are sourced from the aforementioned references. We have provided clarification in the revised manuscript (see Methods: 5. RNA extraction and sequencing).
8. I'm not convinced latrotoxins exist outside of Theridiidae. Is it possible the significant BLAST alignments are based on ankyrin repeats found in latrotoxins and other types of

genes? Please provide more evidence in support of latrotoxins in distant relatives of Latrodectus.
>>>Response: Thank you for your reminder. You have raised an important point here. The toxin gene homologs we obtained through our search were all based on the results of blast analysis using the spider toxin database annotated by previous researchers.
In the revised manuscript, we also established the hidden Markov models (HMM) for different types of toxin proteins based on the database, and further confirmed the results obtained from blastp using HMMER v 3.3. The confirmation of any toxin protein requires strict toxicology or virulence testing. In the revised manuscript, we refer to the target gene obtained through our search as a toxin gene homolog. Based on this, we have also revised some of the less rigorous viewpoints in the discussion. Thank you again for your prompt.
9. Language and readability were mostly fine, but the manuscript could use editing in many places-too many for me to enumerate. One item that jumped out was the use of "losed" for "lost" in some figures. Please correct and have someone read the whole manuscript carefully.
>>>Response: Thank you, we will do our best to review and revise the manuscript.

Reviewer #2:
The manuscript titled "A Trade-off in Evolution: The Adaptive Landscape of Spiders without Venom Glands" described the a comprehensive study of the spider Octonoba sinensis to identify pathways related to metabolism and trachea development that could help explain energy requirements by the spider's extensive wrapping behavior. The manuscript also explores genetic footprint of venom components still present in the venom less spider. The manuscript has a lot of exciting new data, I find the metabolic and trachea genetics very interesting to specialist as well as the general public. I do however have some issues with the venom sections that in my view need to be addressed.
>>>Response: We sincerely appreciate all your valuable comments and suggestions, which helped us in improving the quality of the manuscript. Below is our point-by-point response to each comment.
1. It is worth noting that Uloboridae is not the only spider family to not have venom glands. Holarchaea is another family that has also been described as not having venom glands. This should be mentioned/addressed.
>>>Response: Thank you for your reminder. We have added relevant descriptions in the introduction section (see line 40–44). The text is taken as follows:
“However, as an important means of hunting and defense, the toxin system gives spiders an outstanding advantage in environmental suitability and has allowed them to spread throughout the world. Of course, there are always exceptions, some outliers are believed to be lacking venom glands. Currently, known spiders without venom grands include the Holarchaea genus (2 species)[2–4] and the entire family Uloboridae, with the latter being the most prosperous group of them [5].”
2. I have some issues with Section 7 of the manuscript: "Differential retention of major toxin genes in O. sinensis". Knowing that the family does not have venom glands and has not many millions of years it is not acceptable for the authors to refer to the genes they find in O. sinensis with homology to known venom components as toxin genes. This will lead to many misinterpretations although admittedly it sounds a lot catchier. Moreover, the families that were identified while all are known to be venom components, not all are toxins. For example, the Translationally Controlled Tumor Protein (TCTP) a histamine-releasing factor acts together with Together the cysteine-rich secretory protein (CRISP) family, contributing to inflammatory responses (Sade et al., 2012; Boia-ferreira et al., 2019; Justa et al., 2020). Given this, the authors will need to tone down the claim of having found toxin in a spider that does not have venom glands. This is not surprising assuming a secondarily loss in the family (the most parsimonious interpretation). I believe this also leads to some misinterpretations of the results; it is my understanding that most venom components are hypothesized to have evolved from ancestor proteins that had normal physiological functions that were then recruited. This information is not concluded from the current manuscript, there is a large body of evidence that supports the evolution of venom proteins via processes such as gene duplication followed by subfunctionalization and/or neofunctionalization, horizontal gene transfer, single gene co-option, etc. It follows that expression of venom homologs in the brain region should not come as a surprise since a lot of what are refer to as toxins are thought to be recruited from the neuropeptides, molecules such

as latrodectins (McCowan & Garb 2014). I suggest a major modification of the results/discussion section having more emphasis on the metabolic data and how this can potentially be a trade-off because as written, the manuscript does not describes a trade-off between wrapping and lack of venom.
>>>Response: Thank you for your comments, and we fully accept your suggestion. The toxin gene homologs we obtained through our search were all based on the results of blast analysis using the spider toxin database annotated by previous researchers.
In the revised manuscript, we also established the hidden Markov models (HMM) for different types of toxin proteins based on the database, and further confirmed the results obtained from blastp using HMMER v 3.3. The confirmation of any toxin protein requires strict toxicology or virulence testing. In the revised manuscript, we refer to the target gene obtained through our search as “toxin gene homolog”. Based on our analysis results, we ultimately believe that the toxin genes used for hunting in Uloboridae have been lost, and changed the Result section 7 to “Deficiency of toxin genes in O. sinensis”. We have also deleted some of the less rigorous viewpoints in the Discussion. Thank you again for your prompt.

3. The reason why P. tepidarioriom was used as a reference comparison needs to be stated somewhere. I think it is somewhat arbitrary and I personally would like to hear the authors' reasons. Also why U. diversus, another Uloboridae was not included in the study is beyond me. This species also has a chromosome level genome and could add to the story.
>>>Response: P. tepidarioriom is a globally distributed species and also a type species of spider (10.1186/s12915-017-0399-x; 10.1186/s13227-015-0011-9; 10.1093/molbev/msad239; 10.1016/j.dib.2018.05.106). It has the best protein annotation currently available in spiders and a good genome assembly at the chromosome level. In addition, it has rich online data and annotation resources in major databases such as KEGG and GProfiler. Therefore, using it as a control species can obtain a more comprehensive evolutionary landscape. Moreover, P. tepidarioriom have relatively small differences in body size, ecological niches, and hunting targets compared to O. sinensis. Therefore, considering these aspects, the two species also have comparative value. We have added relevant descriptions in the revision, and the screenshot is as follows:
Indeed, U. diversus is a very important species for the scientific issues involved in this study. In this revision, we have incorporated this important genome into our analysis system (see Fig. 2B, 3A) and supplemented the annotation, increasing the number of coding genes with transcriptome support from 15750 to 19036. Subsequently, we also added some genomes proposed by reviewers to the new analysis and conducted all downstream analyses based on these data.
4. L45-47: I am not sure I understand the meaning of these sentences and I suggest their removal.
>>>Response: We have deleted these sentences according your suggestion.
5. L51-54: There are many spider families that have well-developed trachea with branches extended into their prosoma, such as salticidae and Cybaeidae. To make the case more compelling for why it is particularly important for Uloboridae to have this adaptation I suggest the authors include more detrain about the greater need for energy in the limbs or greater metabolic input and thus there could be a greater presence of mitochondria in the legs or upregulation of genes related to energy metabolism. If this is not expanded, it is unclear why O. sinensis is indeed a good model for this study.
>>>Response: Thank you for your suggestions. Other groups may also require leg strength, but many have abandoned webs in predatory activities. Uloboridae is all web-building spiders, so we pay more attention to the compensation for the loss of venom glands in web-building spiders, and did not pay special attention to other hunting type groups. Comparative transcriptome analysis of motor organs is indeed crucial, and we have included this work in our revised manuscript. Excerpt as follows:
“In order to further explore the evolution in motor function of Uloboridae family, we compared the transcriptome data of the legs between O. sinensis and other species. We used the model species: P. tepidariorum, which is also a web-building spider, as a control. Under consistent standardized conditions, the results showed that a large number of genes were differentially expressed (Figure 3B). Compared to P. tepidariorum, genes higher expression in the O. sinensis legs were most enriched in the mitochondrial matrix, and, other GO terms related to mitochondria also detected

Powered by Editorial Manager® and ProduXion Manager® from Aries Systems Corporation

high enrichment (Figure 4C). This result suggests that at least in the legs, O. sinensis require greater energy consumption compared to typical web-building spiders.”
6. L55: Do not start a sentence with a species name.
>>>Response: Thank you for your reminder. We have made the corresponding modifications (see line 58).
7. L76-80: Uloborid spiders have been described as lacking both venom glands and openings on the fangs for the delivery of venom (Opell 1979; Weng et al. 2006). It is not surprise that you did not observe fang openings in O. sinensis fangs. Therefore, I suggest the authors change the wording from 'prove' to further providing evidence that uloborids are not equipped to deliver venom.
>>>Response: Thank you for your suggestions, we have made the corresponding modifications (see line 83–86).
8. L 94: Not sure "as a percentage of … 98.11%" is needed.
>>>Response: Thank you for your comments, we had anticipated that someone would pay attention to this type of information, now we have moved it to the Additional file (see Results: 2. Observation of behavior and fangs).
9. L100: change to 24,579 and 24,563
>>>Response: Thank you for your suggestions, we have made the corresponding modifications (see line 104–105).
10. L101: "in at least one the following databases: "
>>>Response: Thank you for your suggestions, we have made the corresponding modifications (see line 105).
11. L250-253: I agree that it could be possible that these genes were removed from the genome but it is hard to tell from the current data if this was quick or not after selective pressures changed.
>>>Response: Thank you for your reminder. We have reorganized this section (Section 7) based on the new analysis results. The expression you mentioned here has been rewritten and taken as follows:
“Compared with other genes which it is difficult to find pseudogenes, this gene has complete gene structures and CDS regions, transcriptome data also show that it can be normally transcribed into mRNA (Additional file 6). We speculate that this gene (g31478) may plays a role as a non-toxic gene in O. sinensis.”
12. L297-298: While I do not agree with the statement, at the very least the authors need to provide a lot more information to back up this claim.
>>>Response: Thank you for your reminder. This section of the discussion is indeed too radical and lacks research support. We have reorganized the language and removed the expressions about “preadaptation” and “extraction”.
13. L139-140: Sentence is confusing as written and should be rewritten or deleted.
>>>Response: Thank you for your comments. In the revision, the analysis results under the new background differ from the original manuscript, and this section has been rewritten. We deliberately avoided such issues during the revision process.
14. L324: Where the samples use males or females? Mature or immature? This will be important for expression levels. Why were the spiders starved for a week?
>>>Response: Thank you for your reminder. We have added detailed descriptions of this section in the revision. Excerpt as follows:
“To minimize contamination of intestinal DNA as much as possible, all samples were starvation reared for more than one week at room temperature. Genome DNA for both short and long read sequencing were isolated from the cephalothoraxes of adult female spiders using the Qiagen Blood & Cell Culture DNA Kit (QIAGEN, Hilden, Germany).”
15. L338: what type of tissue was used to generate the genome? Whole spiders?
>>>Response: Thank you for your reminder. We have added detailed descriptions of this section in the revision (see Methods: 1. Sample collection and DNA extraction).
16. L349: experiments "were" conducted.
>>>Response: Thank you for your reminder. We checked for similar errors to the best of our ability.
17. L372: BUSCO should be spelled out, explained and cited the first time is used.
>>>Response: Thank you for your reminder. The Benchmarking Universal Single-Copy Orthologs (BUSCO) method first appeared in the Results: 2. Genome assembly and annotation, and in the revision, we spelled out its full name and cited it (see line 95, 96).

18. L375: what do you mead by spinneret? If you dissected the silk glands out (all the silk glands out) then you not have any tissue other than connective and/or fat attached

	the spinneret. This should be clarified. If the authors took out the larger glands and left the smaller ones behind then the names should reflect that. Are there any images of the tissues used? I will be curious to see what part of the brain and the gut was used for example. Whether the spiders where male or female, at what stage and how many where used should be included for both species.<br>>>>Response: Thank you for your comments. Our approach to dissecting spider spinnerets is similar to that of other appendages. It is to use tweezers to separate it from the body and remove visible internal organs as much as possible. However, the spinner was too small to be completely cleaned and may have a small portion of the silk gland.<br>We have not saved official images. We shoot a few videos and photos to demonstrate the anatomical process, mainly highlighting the anatomy of the intestine and brain. This information will be uploaded to the gigaDB.<br>19. L396: Why was the Uloboridae Uloborus diversus not included in this study? To my knowledge it is also a chromosome level assembly and would have made a stranger case for the conclusions.<br>>>>Response: Thank you for your comments. We have addressed this section in our response to your previous comments. Here we would like to point out that in addition to the Uloborus diversus, we also added two species from RTA clade (Pardosa pseudoannulata and Dolomedes plantarius), replaced the Dysdera silvatica with a better quality genome of Ectatosticta davidi, and removed the less important Hylyphantes graminicola and Stegodyphus mimosarum. In the end, all of these genomes have a protein Busco score of over 90%, and a more suitable evolutionary tree was obtained (see Fig. 2B).<br>20. The manuscript contains both Fig and Figure. For consistency only use one.<br>>>>Response: Thank you for your reminder. We checked for similar errors to the best of our ability.<br>21. Figure 1 suggestions: B- Adult male or female? Move the scale bar to bottom right. C- I find this section to be very confusing and should perhaps be its own figure. The numbering inside the circles make it hard to understand to me. I suggest a different organization for this figure.<br>>>>Response: Thank you for your reminder. We reorganized the Figure 1 and placed the scale bar in channel B in the bottom right corner of that channel; We have added a separate channel. Used to describe the shooting angle in SEM photographs (see Fig. 1).
**Additional Information:**	
**Question**	**Response**
Are you submitting this manuscript to a special series or article collection?	No
**Experimental design and statistics**<br><br>Full details of the experimental design and statistical methods used should be given in the Methods section, as detailed in our Minimum Standards Reporting Checklist. Information essential to interpreting the data presented should be made available in the figure legends.<br><br>Have you included all the information requested in your manuscript?	Yes
**Resources**	Yes

Powered by Editorial Manager® and ProduXion Manager® from Aries Systems Corporation

A description of all resources used, including antibodies, cell lines, animals and software tools, with enough information to allow them to be uniquely identified, should be included in the Methods section. Authors are strongly encouraged to cite Research Resource Identifiers (RRIDs) for antibodies, model organisms and tools, where possible.<br><br>Have you included the information requested as detailed in our Minimum Standards Reporting Checklist?	
**Availability of data and materials**<br><br>All datasets and code on which the conclusions of the paper rely must be either included in your submission or deposited in publicly available repositories (where available and ethically appropriate), referencing such data using a unique identifier in the references and in the "Availability of Data and Materials" section of your manuscript.<br><br>Have you have met the above requirement as detailed in our Minimum Standards Reporting Checklist?	Yes

Manuscript Click here to access/download;Manuscript;giga-1.17-review.docx

A Trade-off in Evolution: The Adaptive Landscape of Spiders without Venom Glands

Yiming Zhang†[1,2,3], Yunxiao Shen†[1,3], Bingyue Zhu[1,3], Pengyu Jin[1], Yejie Lin[2], Tongyao Jiang[1,3], Xianting Huang[1,3], Yang Wang[1,3] and Shuqiang Li[1]*

[1]Key Laboratory of Zoological Systematics and Evolution, Institute of Zoology, Chinese Academy of Sciences, Beijing 100101, China

[2]Hebei Key Laboratory of Animal Diversity, College of Life Sciences, Langfang Normal University, Langfang 065000, China

[3]University of Chinese Academy of Sciences, Beijing 101408, China

† Yiming Zhang and Yunxiao Shen contributed equally to this work.

*Corresponding author: E-mail: lisq@ioz.ac.cn

Abstract

Background: Venom glands play a key role in the predation and defense strategies of almost all spider groups. However, the spider family Uloboridae lacks venom glands and has evolved an adaptive strategy: They excessively wrap their prey directly with spider silk instead of paralyzing it first with toxins. This shift of survival strategy is very fascinating, but the genetic underpinnings behind it are poorly understood.

Results: Spanning multiple spider groups, we conducted multi-omics analyses on *Octonoba sinensis*, and described the adaptive evolution of the Uloboridae family at the genome level. We observed the coding genes of myosin and twitchin in muscles are under positive selection, energy metabolism functions are enhanced, and gene families related to tracheal development and tissue mechanical strength are expanded or emerged, all of which are related to the unique anatomical structure and predatory behavior of spiders in the family Uloboridae. In addition, we also scanned the elements which are absent or under relaxed purifying selection, as well as toxin gene homologs in the genomes of two species in this family. The results show that the absence of regions and regions under relaxed selection in these spiders' genomes are concentrated in areas related to development and neuro-system. The search

for toxin homologs also confirms that there are no toxin coding genes available for hunting in the genome of this group.

Conclusions: This study demonstrates the trade-off between different predation strategies in spiders, either using venom or physical energy and provides insights into the possible mechanism underlying this trade-off. Venomless spiders need to mobilize multiple developmental and metabolic pathways related to motor function and limb mechanical strength to cover the decline in adaptability caused by the absence of venom glands.

Keywords: Venom gland deficient, Adaptive evolution, Genome, *Octonoba sinensis*

Introduction

"Venomous" is a common way that people perceive spiders (Araneae). The toxic, painful and even fatal bite is always frightening. Indeed, almost all spiders are venomous. In the earliest divergent suborder Mesothelae, they already had fangs that could inject venom [1], and some highly toxic species make spiders even more notorious. However, as an important means of hunting and defense, the toxin system gives spiders an outstanding advantage in environmental suitability and has allowed them to spread throughout the world. Of course, there are always exceptions, and some outliers are believed to be lacking venom glands. Currently, known spiders without venom grands include *Holarchaea* (2 species)[2–4] and the entire family Uloboridae, with the latter being the most prosperous group [5]. Compared to *Holarchaea*, which has a smaller body size, fewer species, and limited distribution, the family Uloboridae provides a satisfactory model for us to study the evolution of an important synapomorphy of spiders and the adaptation strategy changes brought about by the loss of important functional traits.

Due to the absence of venom glands, the predation methods of these spiders are also relatively specialized. Observations of the predatory behavior of this group have found that, unlike other spider species that rely on instantaneous explosive force to subdue their prey, the long-time and high-intensity wrapping of prey is very common [6–8]. However, this strategy inevitably incurs a higher energy output

burden. Previous anatomical records indicate that spiders in the family Uloboridae have well-developed trachea, and many branches of the trachea extend into the prosoma and appendages [9, 10]. Unfortunately, there is no relevant research that can link these adaptive characteristics to the venom gland deficiency of this group.

The spider *Octonoba sinensi* belongs to the family Uloboridae. Their body size is relatively larger than other members of the family [11], and this species is widely distributed in East Asia, Southeast Asia and North America [2]. Its habitat is close to human buildings, and the populations are large, so they can be easily collected in cities (Figure 1AB). These aforementioned characteristics make it a model species of the Uloboridae to study the biological characteristics of this family.

In this study, we generated a chromosome-level genome assembly of *O. sinensis*, and high-quality annotation was performed on the genomes of *Pardosa pseudoannulata* and *Dolomedes plantarius*, as well as optimization of annotation for the genomes of *Uloborus diversus* and *Latrodectus elegans*. By leveraging multi-omics datasets from various spider species, we explored the molecular basis of the unique adaptive strategies in the family Uloboridae. In the selection pressure analysis, we found that some important genes related to muscle movement have undergone significant positive selection. In gene family analysis, we observed that with the absence or relaxation of toxins and developmental related genes, gene families related to tracheal development, skeletal development, and tissue force-bearing structures have significantly expanded or emerged in the genomes of this group. Moreover, energy metabolism related genes exhibit high expression, and enzyme activity in related pathways is significantly increased. These all provide a plausible explanation for the improvement in respiratory efficiency and the well-developed tracheal system observed in its anatomy.

Results

1. Observation of behavior and fangs

Through our observations, like other species in the family Uloboridae, *O. sinensis* only arrest their prey through extensive silk wrapping. The time usually exceeds 3 minutes (sometimes even 8–9 minutes), during which there may be several brief breaks (Additional file 1–3). This time is much higher than the

previously recorded wrapping time of all species of group Araneoidea (9.7 ± 3.0 seconds for small prey, 26 ± 42 seconds for big prey) [12].

In addition, for the first time, we examined the fangs of *O. sinensis* from multiple angles with a Scanning Electron Microscope (SEM). Generally speaking, if a species has venom glands, a channel opening for injecting venom should be found on the fangs [1, 13]. We did not observe this in *O. sinensis*, which further providing evidence that Uloborids are not equipped to deliver venom (Figure 1C, D).

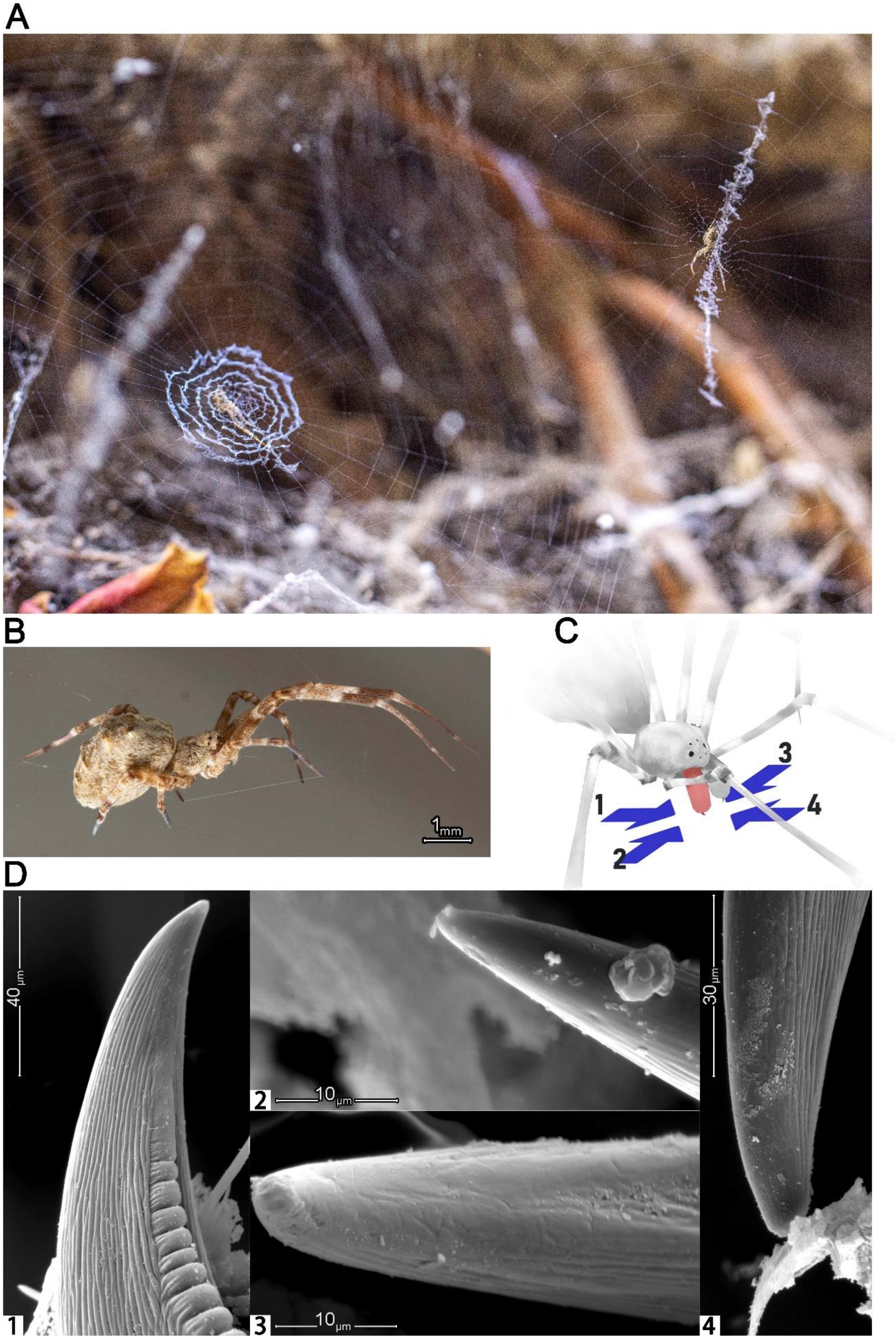
A
B
1mm
C
1
2
3
4
D
40µm
10µm
30µm
1
2
3
4

Figure 1: Observation of *Octonoba sinensis*. (A) Two specimens of *O. sinensis* on their orb-webs in their natural environment. (B) Adult female of *O. sinensis*. (C) The perspective diagram in channel D, with arrows numbered 1–4 indicating the shooting angles of the four images in channel D. (D) Scanning electron microscope image of *O. sinensis* fangs.

2. Genome assembly and annotation

We assembled an *O. sinensis* genome of 1.34 Gb, which is slightly smaller than the prediction of 1.47 Gb based on Illumina data (Additional file 4: Table S1). Average GC content is 32.57%, N50 value is 139.92 Mb. A total of 20 scaffolds were obtained, of which more than 99.9% of the sequences were loaded onto 9 scaffolds that reached the chromosome level (Figure 2A), which was consistent with the previous karyotype analysis of *O. sinensis* [14]. The Benchmarking Universal Single-Copy Orthologs (BUSCO) score is 95.3% [15, 16] in arachnida_odb10, (Additional file 4: Table S2).

In the annotation of repetitive sequences of the genome, we found that the proportion of repetitive regions was 55.08%, and the most recognizable element was DNA transposons, which accounted for 18.5% of the genome (Additional file 4: Table S3, Additional file 5: Figure S1). In other spider genomes, most species are dominated by DNA transposons [17–19]. In our assembly, 24579 coding genes were annotated, and 24563 genes have obtained effective functional annotation in at least one of the following databases: NCBI-Nr (http://www.ncbi.nlm.nih.gov), Swiss-Prot (http://www.uniprot.org/) or EggNOG v5.0 databases (http://eggnog5.embl.de/) [20]. The chromosome loading rate of coding genes is 99.09%. The BUSCO assessment of protein level is 94.8% (Additional file 4: Table S2).

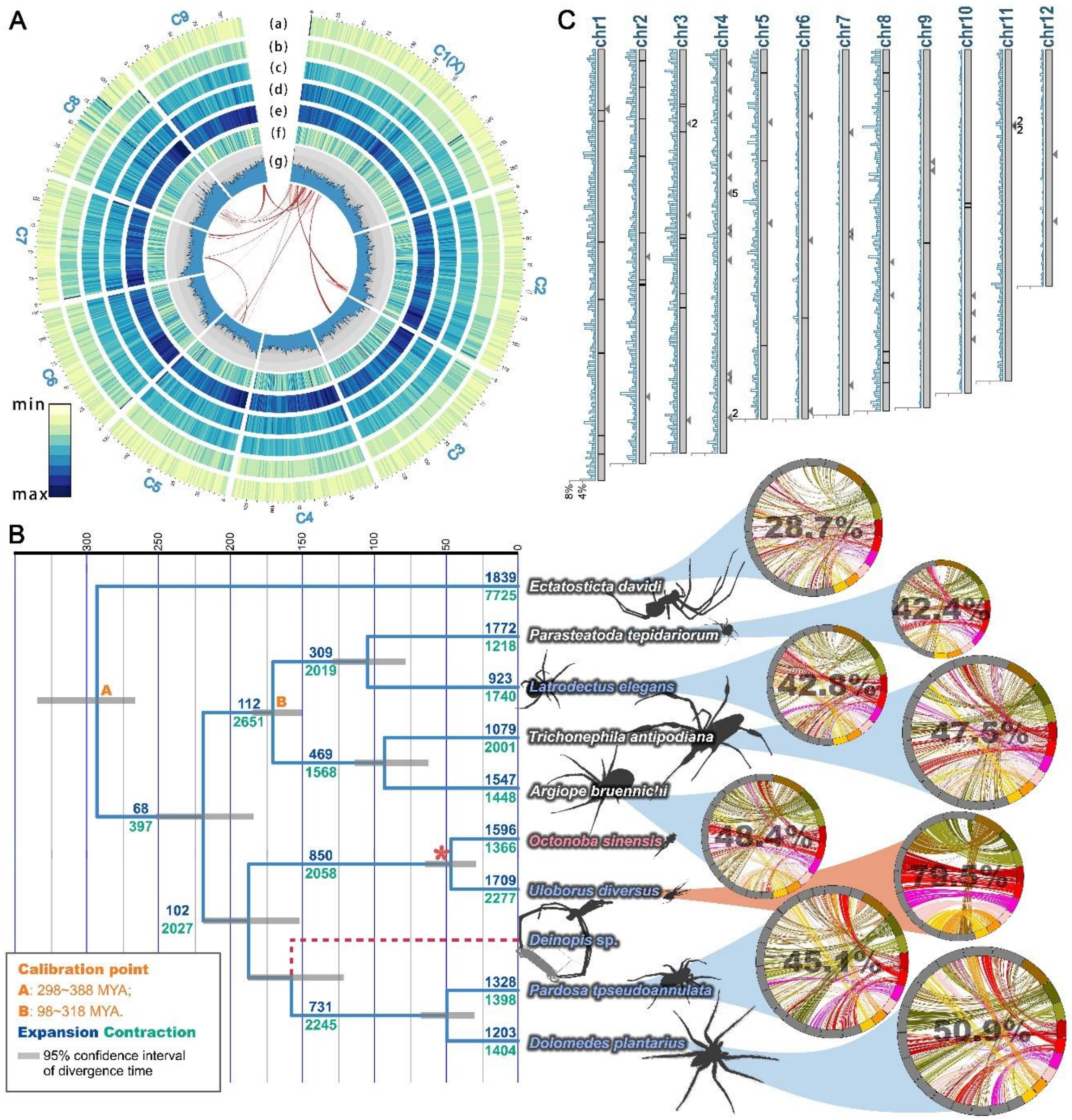

Figure 2: Genome and comparative genomic analysis results from *Octonoba sinensis*. (A) Assembly and structural annotation of the *O. sinensis* genome. The distribution of different elements is marked with lowercase letters from the outside to the inside in the circle diagram, a: gene; b: SINE, c: LINE, d: LTR; e: DNA transposon; f: Highly-Conserved Elements (HCEs); g: GC content. The inner lines of the circle graph are collineation, dark red lines are collineation between chromosomes, pink lines represent collineation within the chromosome. (B) Phylogeny of multiple spider species. All nodes received 100% bootstrap support. We have provided new annotated genomes marked in light blue

font, and new genome assemblies and annotations marked in light red font. The 95% confidence interval of the divergence time is represented by the gray strip on the phylogeny, and the number of expanded and contracted gene families at each node is represented by light blue and light green numbers respectively. The asterisk indicates the foreground branch in the selection pressure analysis. The branch with only transcriptome data cannot deploy CAFE analysis and is highlighted by a red dashed line. The collinearity between *O. sinensis* and other spiders is represented by circle diagrams. The proportion of collinearity segments in the *O. sinensis* genome is indicated in bold percentages. (C) Distribution of all Highly-Conserved Elements (HCEs) in *Parasteatoda tepidariorum* and missing HCEs and genes in *O. sinensis*. The blue bar chart represents the distribution of HCEs, decimals on the ruler represent the proportion of HCEs; the black band represents HCEs with specific deletions in *O. sinensis*; the gray arrow indicates the location of genes with specific deletions in *O. sinensis*. If the number of missing genes at the location is greater than 1, it is indicated with a number.

3. Divergence time estimation and synteny analysis

Genomes of model species — the house spider (*Parasteatoda tepidariorum*), *O. sinensis*, *U. diversus* and seven other representative species (Additional file 4: Table S4) were selected for homologs gene identification (see methods). A total of 1560 single copy gene families shared by all species were identified and used to construct the phylogenetic tree (Figure 2B). All nodes have 100% bootstrap support, and the topological structure and the divergence time of each node prediction are similar to those of previous studies [21–23].

Among the species involved in the above analysis, genomes with chromosome-level assemblies were selected for synteny analysis with *O. sinensis* (Additional file 4: Table S4). There is a trend that the closer the relationship, the stronger the collinearity (Figure 2B).

4. Genes under positive selection and energy metabolism in muscle

In the selection pressure analysis, at the node of the Uloboridae (Figure 2B), 401 genes were under positive selection (Additional file 4: Table S5). Although these genes did not achieve effective GO

enrichment (p. adjust < 0.05), we found that there is tissue preference in the expression of some positive selection genes (PSGs), and, in *O. sinensis*, these genes have the highest enrichment in embryos and muscles (Figure 3A). It is worth noting that these PSGs in muscle tissue include *myosin* (gene ID: g27351), an important component of muscle fibers, and *twitchin* (gene ID: g8872), a key regulator of muscle movement.

To further explore the evolution in motor function of Uloboridae, we compared the transcriptome data of the legs between *O. sinensis* and other species. We used the model species *P. tepidariorum*, which is also a web-building spider, as a control. Under consistent standardized conditions, the results showed that a large number of genes were differentially expressed (Figure 3B, Additional file 4: Table S6). Compared to *P. tepidariorum*, genes higher expression in the *O. sinensis* legs were most enriched in the mitochondrial matrix, and, other GO terms related to mitochondria also detected high enrichment (Figure 3C). This result suggests that at least in the legs, *O. sinensis* requires greater energy consumption compared to typical web-building spiders. On this basis, we examined the activities of several key enzymes in the mitochondria and involved in energy metabolism, including hexokinase (HK), pyruvate dehydrogenase (PDH), α-ketoglutarate dehydrogenase (α-KGDHC), NADH dehydrogenase (ND), and ATP synthase (ATPase). Our results revealed that the activity levels of these five enzymes in the body of *O. sinensis* were higher compared to those in *P. tepidariorum* (Figure 3E). Furthermore, while there was no significant difference in CO_2 production rates between *O. sinensis* and *P. tepidariorum* in a resting state, *O. sinensis* exhibited significantly higher rates under fatigue treatment (Figure 3D). These findings suggest that the evolution related to energy metabolism in the motor organs may be a key factor in the sustained output power of species in the Uloboridae family.

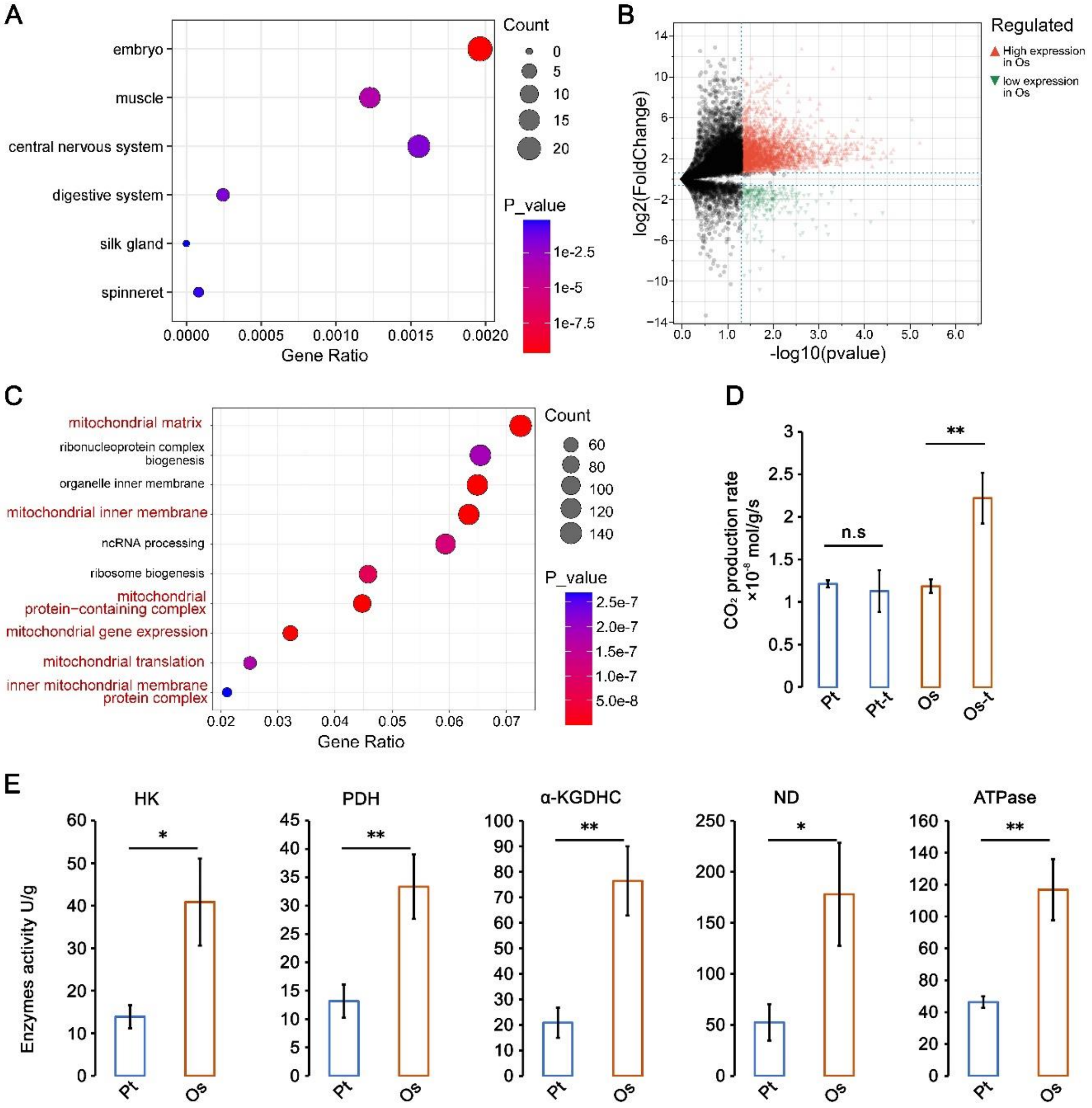

Figure 3: Positive selection signals related to energy metabolism. (A) tissue enrichment of genes under positive selection. (B) Differential expression analysis of the legs of *Parasteatoda tepidariorum* and *Octonoba sinensis* (C) GO enrichment of genes that are significantly overexpressed in *Octonoba sinensis* legs. GO terms related to the mitochondrion are highlighted in red. (D–E) Metabolic rate measurement (D) and enzyme activity (E) of hexokinase (HK), pyruvate dehydrogenase (PDH), alpha-ketoglutarate dehydrogenase complex (α-KGDHC), NADH dehydrogenase (ND) and ATP synthase (ATPase). Pt: *P. tepidariorum*, Os: *O. sinensis*, -t: under fatigue treatment. Significant differences are

denoted by *P < 0.05, **P < 0.01, and n.s., not significant.

5. Expanded and new emergent gene families

To compare the genomic differences between Uloboridae and other spiders, expanded and new emergent gene families were identified. Results indicate that 123 gene families have undergone significant expansion at the ancestral node of the Uloboridae (Figure 4A). We extracted members of these gene families from the *O. sinensis* genome and found that members from four superfamilies were the most common in the annotation results, and they were: FH2 domain containing 1 (FHDC1), FBN1, WD40 repeat proteins (WD40), and seven-(pass)-transmembrane domain receptors 1 (7tm_1) (Figure 4B, Additional file 4: Table S7). Interestingly, studies have shown that FHDC1 proteins not only play a crucial role in the development of the tracheal system in fruit flies [24] but also have significant implications in muscle movement [25, 26]. In addition, the preproprotein of FBN1 is proteolytically processed to generate two proteins including the extracellular matrix component fibrillin-1 and the protein hormone asprosin. Fibrillin-1 is an extracellular matrix glycoprotein that serves as a structural component of calcium-binding microfibrils. These microfibrils provide force-bearing structural support in elastic and nonelastic connective tissue throughout the body. Asprosin has been shown to regulate glucose homeostasis [27]. Apart from the four superfamilies mentioned above, we have deployed GO enrichment analysis of the other expanded gene families. REVIGO [28] results show that these annotated genes were mainly enriched in the transport of carbohydrates and organic acids, the immune system, and the functions related to transposable elements (Figure 4C).
Two-hundred-sixty-nine new emergent gene families have been identified in both *O. sinensis* and *U. diversus*, with a total of 658 members in the *O. sinensis* genome (Additional file 4: Table S8). GO enrichment results showed that these genes are more enriched in GO terms that are related to bone trabecular development (Figure 4D).

Generally speaking, spiders lack endurance, and the vast majority of species rely on instantaneous explosive power for hunting [13]. However, species of Uloboridae are able to exercise intensely for nearly an hour [8]. Our results indicate that genes related to tracheal development, skeletal

development, tissue force-bearing structures and energy metabolism have significantly expanded or emerged in the genome of Uloboridae. We believe that the evolution of these aspects is highly likely to be related to their increased endurance.

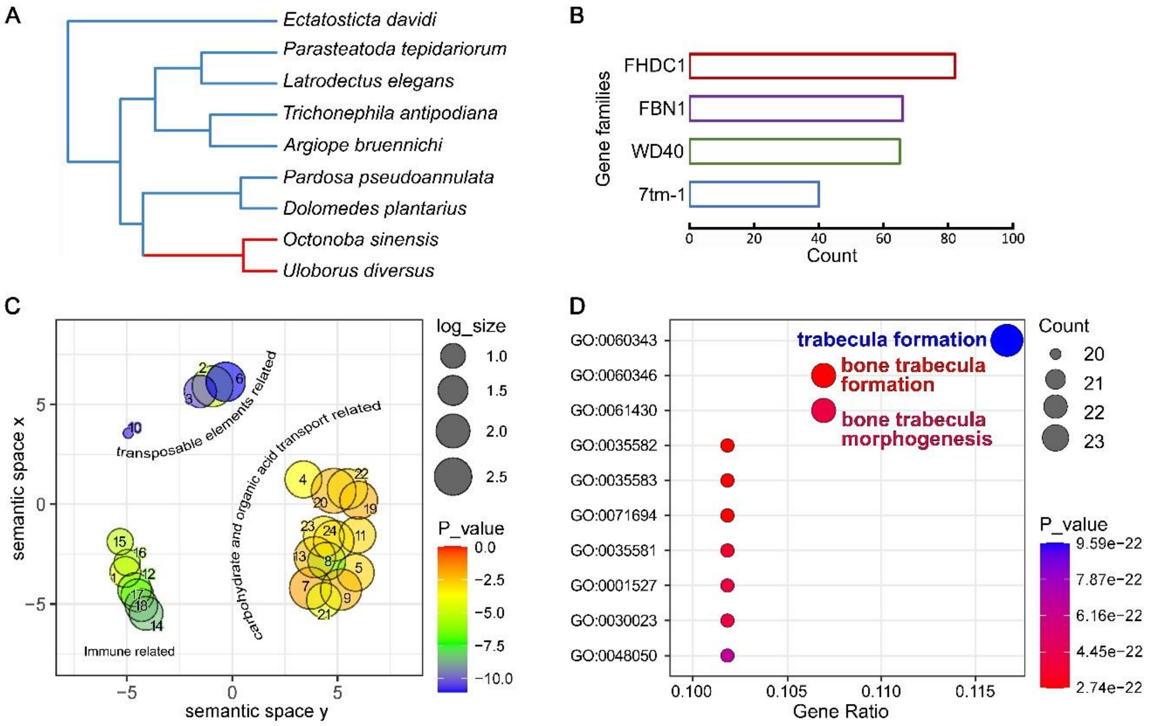

Figure 4. Expanded and new emergent gene families. (A) Phylogeny for calculating the expansion and contraction of gene families. Uloboridae branches are in red. (B) The four superfamilies with the highest number of clearly annotated genes. (C) REVIGO plot of GO enrichment results for the other expanded gene families, excluding four superfamilies. The numbers in the figure refers to different GO entries: 1-response to molecules of bacterial origin, 2-DNA recombination, 3-DNA transposition, 4-receptor-mediated endocytosis, 5-carbohydrate transport, 6-DNA integration, 7-organic anion transport, 8-sialic acid transport, 9-organic acid transport, 10-transposition, 11-carbohydrate transmembrane transport, 12-response to type II interferon, 13-carboxylic acid transport, 14-response to interleukin-4, 15-cellular response to biotic stimulus, 16-cellular response to molecule of bacterial origin, 17-cellular response to type II interferon, 18-cellular response to interleukin-4, 19-monoatomic anion transmembrane transport, 20-inorganic cation transmembrane transport, 21-carbohydrate

derivative transport, 22-proton transmembrane transport, 23-organic acid transmembrane transport, 24-carboxylic acid transmembrane transport. (D) GO enrichment of new emergent gene families in Uloboridae.

6. Absent regions and genes under relaxed purifying selection

Due to the absence of venom glands, the genes or functional regions specifically involved in the venom gland system in Uloboridae may be subjected to relaxed purifying selection or gradually lost from the genome. To obtain this information, spanning nine spider genomes (Figure 3A), Highly-Conserved Elements (HCEs) were searched and the sites with Uloboridae-specific deletions among them were identified (Figure 1C, Additional file 4: Table S9 S10). In addition, absent genes and genes under selective relaxation in the *O. sinensis* and *U. diversus* genomes were analyzed against the background of species with venom glands (Figure 2B, Additional file 4: Table S11 S12). We conducted functional enrichment on the above results and found that the biggest difference between Uloboridae and background species comes from the development, especially the neuro-development, related gene family (Figure 5A–B).

In the homologs missing from Uloboridae, we found that 5 genes belong to two toxin related gene families (LRR and CRISP), as well as three transcription factors. It is worth noting that the expression patterns of the homologs of two LRR genes (LOC122270931, LOC107442855) in *P. tepidariorum* indicate their highest expression in venom glands, although neither of them has been annotated as homologs to known toxin genes (Figure 5C). Three transcription factors belong to important components that activate transcription: “protein c-ets-2”; the “cooled coil and C2 domain containing protein” that regulates neurotransmitter expression; and the component of the STAGA complex: “ataxin-7”.

In addition, We referred to the venom gland specific expression module of *P. tepidariorum* obtained in a previous study (n = 1088, Additional file 4: Table S13) [29] and found that only one gene (LOC107440400), which is specifically lost in Uloboridae, intersects with this module. This gene is a SOBP (Sine Oculis-Binding Protein Homolog) gene, and the protein encoded by this gene is involved

in development of the cochlea, and genetic defects are also related to intellectual disability (Figure 5C).

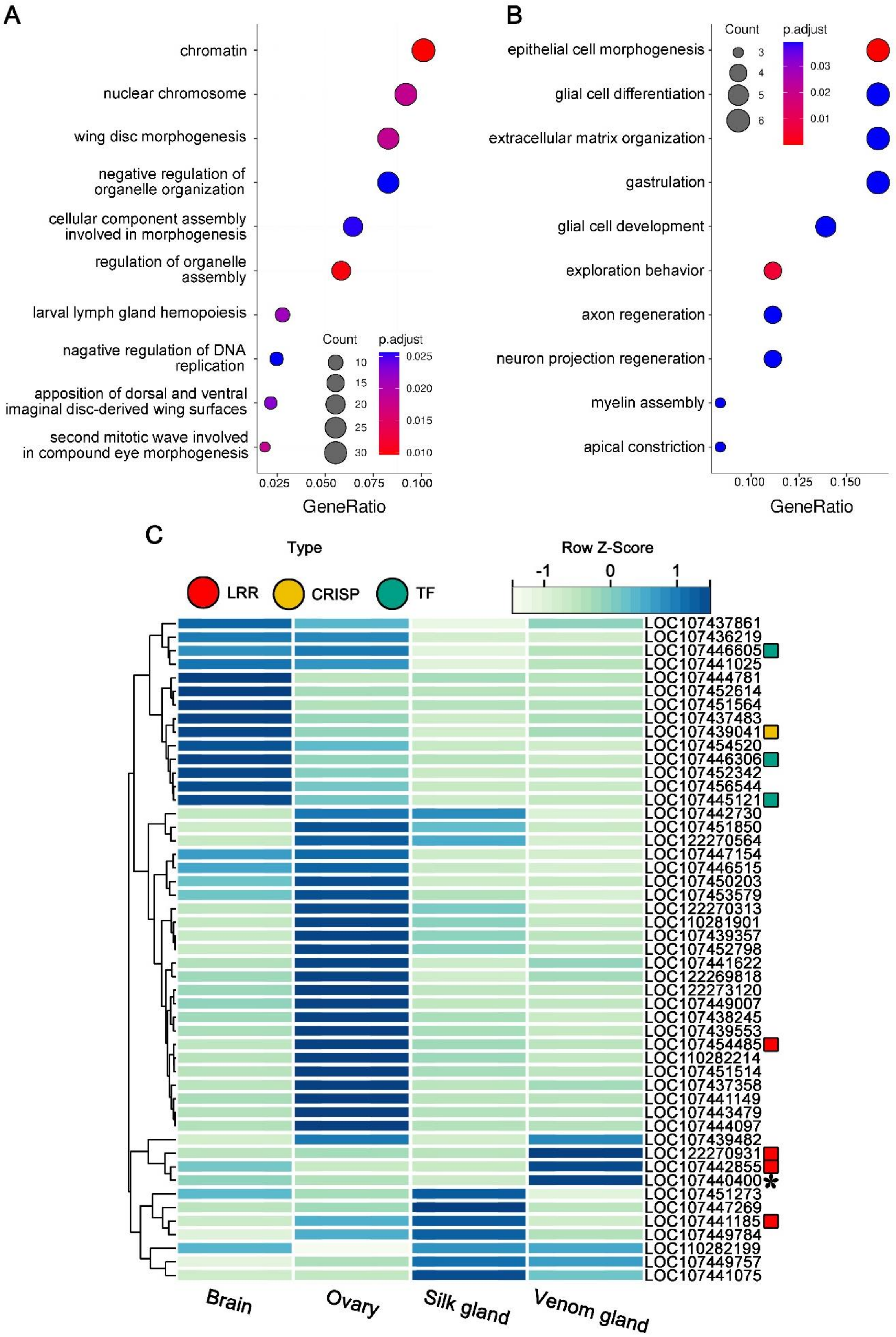

A
chromatin
nuclear chromosome
wing disc morphogenesis
negative regulation of organelle organization
cellular component assembly involved in morphogenesis
regulation of organelle assembly
larval lymph gland hemopoiesis
nagative regulation of DNA replication
apposition of dorsal and ventral imaginal disc-derived wing surfaces
second mitotic wave involved in compound eye morphogenesis
Count
10
15
20
25
30
p.adjust
0.025
0.020
0.015
0.010
0.025 0.050 0.075 0.100
GeneRatio
B
epithelial cell morphogenesis
glial cell differentiation
extracellular matrix organization
gastrulation
glial cell development
exploration behavior
axon regeneration
neuron projection regeneration
myelin assembly
apical constriction
Count
3
4
5
6
p.adjust
0.03
0.02
0.01
0.100 0.125 0.150
GeneRatio
C
Type
LRR
CRISP
TF
Row Z-Score
-1 0 1
LOC107437861
LOC107436219
LOC107446605
LOC107441025
LOC107444781
LOC107452614
LOC107451564
LOC107437483
LOC107439041
LOC107454520
LOC107446306
LOC107452342
LOC107456544
LOC107445121
LOC107442730
LOC107451850
LOC122270564
LOC107447154
LOC107446515
LOC107450203
LOC107453579
LOC122270313
LOC110281901
LOC107439357
LOC107452798
LOC107441622
LOC122269818
LOC122273120
LOC107449007
LOC107438245
LOC107439553
LOC107454485
LOC110282214
LOC107451514
LOC107437358
LOC107441149
LOC107443479
LOC107444097
LOC107439482
LOC122270931
LOC107442855
LOC107440400
LOC107451273
LOC107447269
LOC107441185
LOC107449784
LOC110282199
LOC107449757
LOC107441075
Brain
Ovary
Silk gland
Venom gland

Figure 5: Absent regions and regions under relaxed selection. (A) GO enrichment of genes under relaxed selection. (B) GO enrichment of genes related to missing Highly-Conserved Elements (HCEs). (C) Expression patterns in *Parasteatoda tepidariorum* of genes that are missing in both the *Octonoba sinensi* and *Uloborus diversus* genome. Different colored markers highlight different types of genes, while the asterisk indicates the gene specifically missing from the *P. tepidariorum* venom gland specific expression module.

7. Deficiency of toxin genes in *O. sinensis*

To search for toxin genes, we integrated the results of previous studies and established a comprehensive toxin protein database (Additional file 7) and screened toxin gene homologs with the same threshold in different species. In the *O. sinensis* and *U. diversus* genomes, we have identified 12 and 11 homologs, respectively, that have similar structures to members of six major toxin or venom component gene families (Latrotoxin, Latrodectin, CRISP, ICK, TCTP, EF-hand and ctenitoxin). Our result indicates that the number of toxin related homologs in Uloboridae is indeed relatively small [30–33], but still higher than that in some venomous spiders, such as *Ectatosticta davidi* (Additional file 4: Table S14). However, by characterizing the expression positions of these *O. sinensis* homologs in *P. tepidariorum*, we found that the toxin related homologs of *O. sinensis* were not found to be highly expressed in venom glands (Figure 6, Additional file 6). Latrotoxin, a class of toxins that have gained a lot of attention, some of which have strong toxicity to vertebrates [34]. In our results, three genes in *O. sinensis* are homologs to this family. However, in the phylogenetic analysis, we found that these homologs in *O. sinensis* are not clustered on the same branch as the reported Latrotoxin (Additional file 5: Figure S2). There is a hypothesis regarding the evolution of venom components that ancestors of toxin proteins were originally proteins with normal physiological functions which were recruited in venom glands to play the role of venom components [35], and our results also support this viewpoint.

To further explore the evolutionary processes of *O. sinensis* toxin genes in the absence of venom glands, we conducted a pseudogene search on non-coding regions of the *O. sinensis* genome, but traditional

search methods did not identify pseudogenes (blastn, E-value 10^{-5}, matching length 50 bp) [36]. However, we did find more traces of toxin gene homologs in the blastx search through the protein sequence in the toxin genes database. These results include 48 different genomic regions, but only one of which had an effective hit with the relatively reliable toxin gene of *P. tepidariorum* (LOC107440051) (Additional file 4: Table S15, Figure 6).

By searching for toxin gene homologs in collinearity fragments of *O. sinensis* and *P. tepidariorum*, a particular class of genes was found in *O. sinensis*. These genes are located in the same place as the *P. tepidariorum* toxin gene homologs in the collinearity segment, but they can no longer be identified as toxin genes (below the minimum recognition threshold, see methods) (Figure 7B, red ribbon). This includes a pair of CRISP genes, which is highly expressed in the venom glands of *P. tepidariorum* (LOC107437238). However, in *O. sinensis* (g31478), this gene only has one CAP region remaining and cannot been identified as a homolog of toxin genes (Additional file 4: Table S16). Compared with other genes in which it is difficult to find pseudogenes, this gene has complete gene structures and CDS regions. Transcriptome data also show that it can be normally transcribed into mRNA (Additional file 6). We speculate that this gene (g31478) may play a role as a non-toxic gene in *O. sinensis*.

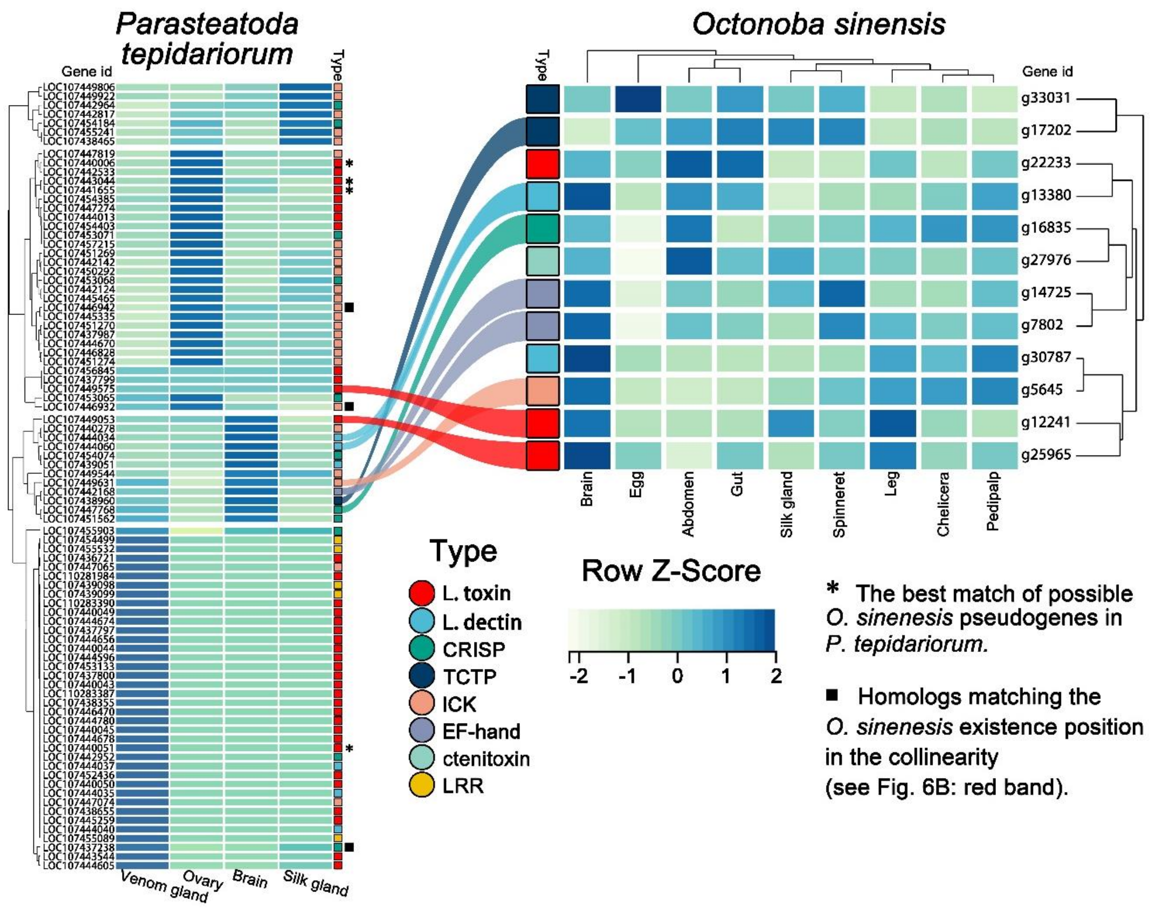

Figure 6. The expression patterns of toxin homolog coding genes in *Octonoba sinensis* and *Parasteatoda tepidariorum*. The ribbon connects the homologs genes. The black squares and asterisks indicate genes found in *P. tepidariorum* that are related to traces of the *O. sinensis* genome.

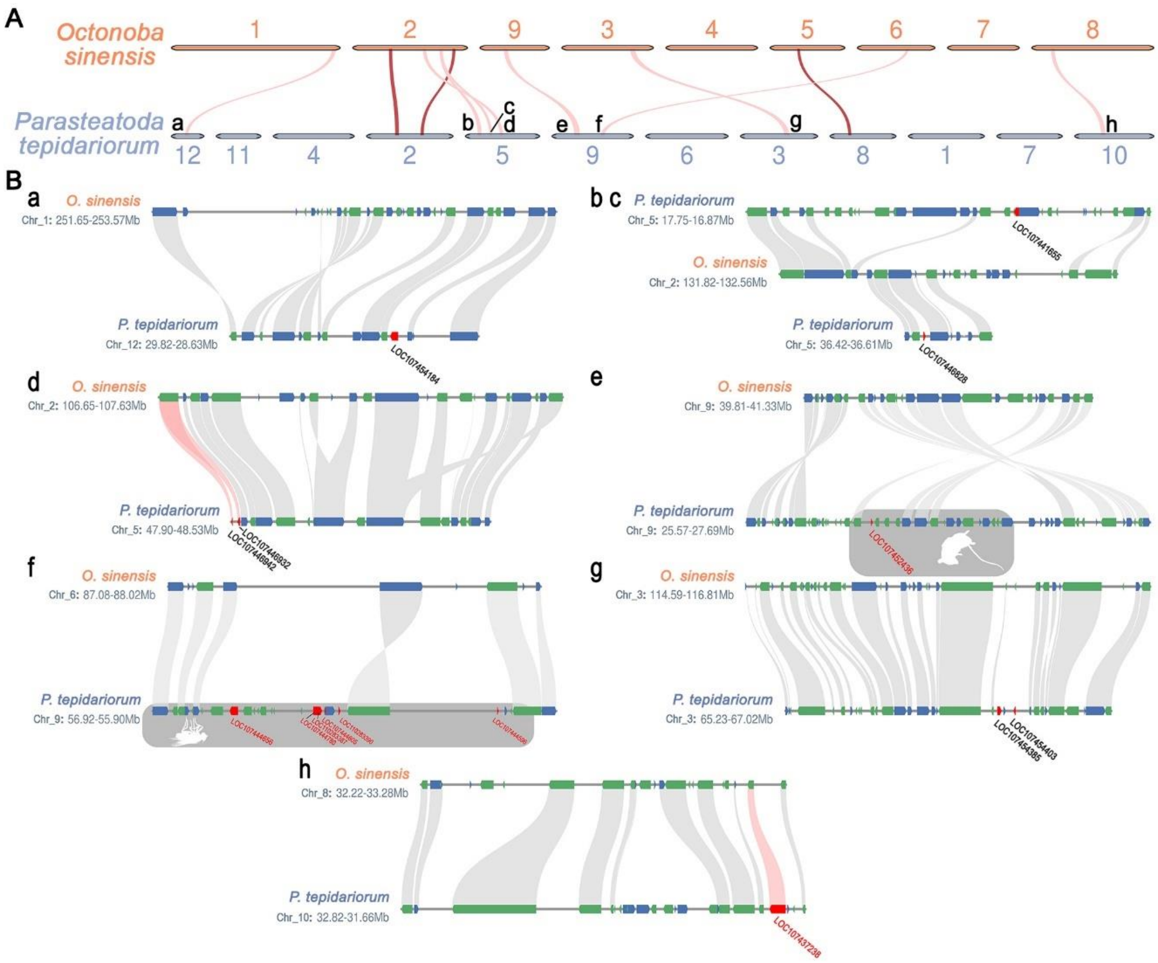

Figure 7. Collinearity containing toxin homologs between *Octonoba sinensis* and *Parasteatoda tepidariorum*. (A) Collinearity of all toxin homologs. If the toxin homologs were lost in *O. sinensis*, the collinearity fragments are represented by a pink ribbon. (B) Gene correspondence in each collinearity fragment where toxin gene deletion occurs in A. Each lowercase letter corresponds to the collinearity fragments represented by the same letter in A; the toxin gene names in *P. tepidariorum* are listed, and the red gene names highlight the toxin genes that are highly expressed in the venom glands. The toxin genes in the gray background belong to the Latrotoxin gene family, and different silhouettes indicate the type of toxin and the target group of toxicity. Mouse = vertebrates (alpha-latrotoxin), Fly = insects (delta-latrotoxin); the red ribbons indicate that the collinearity of the linked genes is extremely weak, below the threshold (see methods).

Discussion

During the process of evolution, the loss of important organs is caused by a series of developmental changes. Typical examples include various species of eyeless organisms [37–41], as well as limb degeneration in some quadrupeds [42–44]. We summarized the specific differences in the genome of *O. sinensis* and *U. diversus* relative to the background and found that many genes related to absent regions and regions under relaxed selection were enriched in developmental GO terms (Fig 3A–D), which was consistent with previous studies [37–44]. Simultaneously, using venom or controlling venom glands is a complex neural activity [45–47]. Our results show that the enrichment of GO terms related to specialized neuro-development suggests that some of these genes may be involved in the development of the nervous control system of venom glands.

Although the web-building behavior of spiders is well known, the venom system seems more important than the former. Using webs for predation occurred at least 50 million years (MYs) after the use of venom glands in hunting [22]. Moreover, among the youngest and diverse Retrolateral Tibial Apophysis (RTA) clade, the vast majority of spiders abandoned webs for predatory activities. This resulted in most extant spiders relying solely on the injection of toxins to immobilize their prey [48, 49]. From published data, species without venom glands are rare, and all spider species without venom glands are adept at relying on webs for predation [1, 13], this seems to be a trade-off.

For burrowing or wandering spiders, they usually subdue their prey with venom. However, within the orb-weaving spiders (Araneoidea), many species first use entanglement to restrain large prey and then use venom [12]. Although entanglement is extremely crude compared to the behavior of Uloboridae species, this predatory strategy may lessen selective pressure for the use of venom. In Uloboridae, the behavior of wrapping prey is highly specialized, and the high-intensity and lengthy physical output requires extremely high endurance. Previous studies have shown that the entire process can last from a few minutes to nearly an hour, and the spider silk used can reach over a hundred meters [6–8].

For predators, there is a widespread evolutionary cost trade-off between chemical and physical attack strategies, and previous research has often focused more on the former, such as venom. However, in the background of almost all spiders being toxic predators, we have described the genetic underpinnings for the alternative choice in this trade-off. In this study, no reliable toxin gene was identified in the *O.*

sinensis genome, and some homologs of toxin genes that we identified expressed in other body organs may have performed some non-toxin functions. However, the adaptive evolution in Uloboridae that is related to the respiratory system, motor system, and energy metabolism, may cover the decline in adaptability caused by the absence of venom glands.

Methods

1. Sample collection and DNA extraction

Live *O. sinensis* were field-captured from Olympic Park, Chaoyang District, Beijing, China. To minimize the contamination of impurities in the digestive tract as much as possible, all samples were starvation reared for more than one week at room temperature. Genome DNA for both short and long read sequencing were isolated from the cephalothoraxes of adult female spiders using the Qiagen Blood & Cell Culture DNA Kit (QIAGEN, Hilden, Germany).

2. Observation of predation behavior and examination of fangs

We recorded a series of videos to observe the predation behavior. Wrapping duration was timed. If the spider stopped for more than 3 seconds, the timing also stopped, but would continue if it began again. If the spider begins to soak (*O. sinensis* will emit liquid onto their prey before eating) or leave the prey, the video was terminated.

The dissected spider fangs were pasted onto a copper substrate at different angles, dried with a CO_2 critical point drying method, coated with gold, and then observed with the SEM (model: FEI Quanta 450).

3. Genome sequencing and genome size estimation

Short-insert (300–400 bp) libraries of *O. sinensis* were sequenced with the MGISEQ-2000 platform using paired-ends (PE) reads of 150 bp (BGI, Shenzhen, China). To remove low-quality reads and adapters, raw reads were trimmed by Trimmomatic v0.39 [50]. A total of 259.82 Gb of clean data were obtained for *O. sinensis* for survey analysis and assembly correction.

For long read sequencing, 'SMRTbell' (double-stranded DNA template capped by hairpin loops at both ends) libraries were constructed according to the standard protocol of PacBio using 15 kb of preparation

solution (PacBio, California, USA). The high-fidelity (HiFi) libraries were sequenced on three SMRT cells on the PacBio Sequel II system in Circular Consensus Sequencing (CCS) mode at Novogene Technology Co. and generated 67.18 Gb HiFi data (3,967,026 reads) total [51].

To further improve the continuity of the assembled genomes, chromosome conformation capture (Hi-C) experiments were conducted [52]. Hi-C libraries were prepared following a published protocol with minor modifications [53]. For cross-linking, samples were fixed with 1% formaldehyde. The cross-linked DNA was digested with MboI restriction endonuclease and marked with biotin-14-dCTP to remove non-ligated DNA fragments. The ligated DNA was extracted with a QIAamp DNA Mini Kit (QIAGEN). The purified DNA was then sheared to ~350 bp fragments and followed by a standard Illumina library preparation protocol [54]. Hi-C sequencing of *O. sinensis* was conducted on the MGISEQ-2000 platform with PE 150 bp (BGI, Shenzhen, China). We then filtered the raw reads using Juicer v1.6.2 [55] to remove low-quality reads and adapters, yielding 201.99 Gb of clean data.

Before *de novo* assembly, we estimated the genome size of this species. Using the Illumina data, Jellyfish (v2.1.3) [56] was employed to calculate the frequency of each K-mer (k = 17~31). Then, the genome size of *O. sinensis* was estimated using a method based on K-mer distribution.

4. *De novo* Genome assembly and quality assessment

PacBio reads were first assembled using two *de novo* assemblers: hifiasm v0.15.2 [57] and wtdbg2 v2.5 [58]. The best assembly was selected according to the optimal continuity and completeness. The final version of contigs was polished with Racon v1.4.17 for three rounds (https://github.com/isovic/racon) based on long reads, and NextPolish v1.4.0 [59] using short reads. Contig level genome completeness assessment was performed using BUSCO v5.2.2 [15, 16]. Genome consistency assessment was evaluated by mapping the short reads to the genome with Minimap2 v2.24-r1122 [60] and Samtools v1.10 [61].

We used Hi-C-based proximity-guided assembly to generate chromosomal level genome assemblies for *O. sinensis*. Hi-C library sequencing data were mapped to the contig level genome using Juicer. The 3D-DNA v180922 [62] pipeline was executed to construct the chromosomes and correct the errors. We further performed correction with Juicebox Assembly Tools v1.11.08 [63]. The completeness of the

chromosomal level assembly was assessed by BUSCO.

5. RNA extraction and sequencing

Different tissue samples (spinneret, leg, brain, gut, silk gland, pedipalp, chelicera and abdomen) (Additional file 6) from adult female *O. sinensis* and eggs (development stage undetermined) were dissected for total RNA extraction using an RNAsimple Total RNA kit (TIANGEN, Beijing, China). The RNA-seq libraries were constructed with insert sizes of ~150 bp and sequenced on the NovaSeq 6000 platform. We produced ~6 Gb of data per sample. Low-quality reads, reads with adapters, and unknown bases were filtered using Trimmomatic.

In addition, legs from adult female *P. tepidariorum* were subjected to transcriptome analysis according to the above process, and transcriptome data from four tissue transcriptomes (PRJNA934108, including brain, ovary, silk gland and venom gland) have been downloaded from the Sequence Read Archive (SRA) database.

6. Genome annotation

The RepeatModeler v2.0.2 [64] and RepeatMasker v4.1.2-p1 [65] pipelines were used to annotate repetitive sequences in genome.

Gene annotation was based on the braker v2.1.6 [66] pipeline, which combines the whole protein sequences of the 9 species in this study (Additional file 4: Table S4) and more than 320 Gb multi tissue transcriptome data for comprehensive annotation.

Gene function annotation is based on NCBI-Nr (http://www.ncbi.nlm.nih.gov), Swiss-Prot (http://www.uniprot.org/) and EggNOG v5.0 (http://eggnog5.embl.de/) databases. The tRNA was predicted using the program tRNAscan-SE v2.09 [67]. Other non-coding RNAs were annotated with the Rfam v14.8 [68] database through infernal v1.1.4 [69].

Among the other species involved in this study, *P. pseudoannulata* and *D. plantarius* only have assembled sequence data currently. The genome of *L. elegans* has high assembly quality, but the protein BUSCO score is only 63.7%; *U. diversus* only has 15750 annotated protein coding genes, significantly less than other spider species. To obtain more reliable results for downstream analysis, we annotated the genomes of the aforementioned species based on transcriptome data. For *P. pseudoannulata*, *D.*

plantarius and *L. elegans*, we used our own annotations, and for *U. diversus*, we used our annotation to supplement the original one. All annotation strategies are based on transcriptome data from the SRA (Additional file 4: Table S17) database and carried out through the TransDecoder pipelines (v5.5.0, https://github.com/TransDecoder). We also added *Deinopis* sp. of Deinopidae, and assembled all of its proteins sequence using transcriptome data (DRR297048), quality control was done using fastp version 0.21.0 [70]. De novo assemblies were done using Trinity-v2.11.0 [71] under default settings. ORF prediction was done using TransDecoder. Redundancy reduction was done with CD-HIT version 4.8.1 (-c 0.98 -n 10) [72]. All the newly provided annotations mentioned above have a protein BUSCO score of over 90% (Additional file 4: Table S2).

7. Orthologous gene identification, phylogenetic and synteny analysis

We used OrthoFinder v2.5.4 [73] to analyze the annotation information of species in this study (Additional file 4: Table S5). In the pipeline, Mafft v7.453 [73] was used to perform multiple sequence alignment, blastp v2.9.0+ [75] was used to perform sequence searches, and the phylogenetic tree was constructed using IQ-TREE v2.2.0 [76]. The calibration points are from fossil specimens [21]. The analysis was run twice, once to calculate the divergence time of different species, and the second to calculate the expansion and contraction of gene families. The latter did not include *Deinopis* sp. (Figure 3A).

Collinearity analysis was conducted between *O. sinensis* and other species with chromosome level genomes (*T. antipodiana*, *A. bruennichi*, *L. elegans*, *U. diversus, P. pseudoannulata*, *D. plantarius* and *E. davidi*.) using the MCscan pipeline (Python version) in the jcvi toolkit [77].

8. Test for selection pressure and gene family expansions/contractions

For all species (Figure 2B), we used the *O. sinensis* genome as a reference to extract the Reciprocal Best Hits (RBH) based on blastp. Finally, 5848 RBH clusters (Additional file 4: Table S18) were retained for analysis.

To scan for genes under positive selection in *O. sinensis*, RBH clusters were used for the branch site model analysis using CODEML in PAML v4.9j toolkit [78]. Each gene family sets *O. sinensis* and *U. diversus* as foreground branches (Figure 4A). “Model A” and “Model A-null” models were compared.

"Model A" assumes that the selection pressure of foreground branches is greater than that of background branches, and "Model A-null" is an alternative hypothesis.

To statistically test which genes of Uloboridae are under relaxed purifying selection, we used RELAX in the HYPHY v2.5.2 [79] toolkit to infer the free relaxation parameter k at the node of *O. sinensis* and *U. diversus* branches for genes shared by all species in the Phylogeny of Figure 2B (Additional file 4: Table S4). The relaxation parameter k is an exponent for selection parameters between the foreground and the background branches. A $k > 1$ suggests selection is more intensified in the foreground branch vs. the background branch and vice versa.

For gene family expansions/contractions, mcmctree in the PAML v4.9j toolkit was used to estimate the divergence time of each node in the phylogenetic tree of OrthoFinder pipeline results (without *Deinopis* sp.) (Figure 4A). Next, we used CAFE v4.2 [80] under the default parameters to analyze the gene family expansions and contractions of eight spider species.

9. Identification of HCEs

To identify the HCEs, we initially generated pairwise sequence alignments across all eight spider genomes of the eight species (Figure 4A) with LASTZ v1.04.15 [81] and chainNet [82], using the *P. tepidariorum* genome as the reference. We then used MULTIZ v11.2 [83] to combine the pairwise alignments into multiple sequence alignments. Subsequently, we ran phyloFit in the PHAST package [84] with the topology from OrthoFinder to estimate the neutral ('nonconserved') model based on fourfold degenerate sites. With the nonconserved model as input, we ran phastCons [85] to estimate conserved models with its intrinsic function and predicted the HCEs.

The distribution of HCEs in exons, introns, 2000 bp upstream and 2000 bp downstream of genes and intergenic regions was summarized with Annovar (https://annovar.openbioinformatics.org/) [86, 87] based on the genome annotation information of *O. sinensis* or *P. tepidariorum*.

10. Homologs of toxin gene family identification and analysis

Previous datasets were used as references (Additional file 7) [32, 88–90], and we searched for target genes using blastp v2.9.0+ (E-value less than 1e-10, matching length greater than 70% of the reference sequence, and hit area mismatch less than 30%). We also established hidden Markov models (HMM)

for different types of toxin proteins based on the database, and further confirmed the results obtained from blastp using HMMER v 3.3 [91]. For the search of hidden toxin-related pseudogenes in *O. sinensis*, we referred to the identification criteria of human genome pseudogenes [36], and used blastn v2.9.0+ and blastx v2.9.0+ to search for candidates.

11. Physiological index measurement

Assays were performed as described previously [92]. CO_2 production rate was used as a proxy for metabolic rate (MR). The assays were conducted in a closed-circuit system with a volume of 73.3 ml at a temperature of 25 °C, a pressure of 100.6 kPa, and a flow rate of 110 ml/min. MR was calculated as the amount of CO_2 produced per gram of body mass per second, using the equation MR = MCO_2/T/body mass, where MCO_2 represents the amount of oxygen substance (in mol). To put spiders into a state of fatigue, we stimulated the spider's legs with a dissecting needle and kept it in a high-intensity state of exercise for ten minutes.

Enzyme activity was measured using the corresponding reagent kit (Wuhan Mosak Biotechnology Co., Ltd. KT50129, KT50577, KT42310, KT41589, KT87867). Female spider individuals in a resting state were fixed in liquid nitrogen after weighing and stored at -80 °C. Prior to testing, homogenize the samples were homogenized and diluted to 500 µl as the test solution following the supplier's protocol

Additional Files

Additional file 1–3: Hunting video.

Additional file 4: Table S1. Genome survey prediction of *Octonoba sinensis*. **Table S2.** Genome assembly. **Table S3.** Repeat sequences of *Octonoba sinensis* genome. **Table S4.** Transcriptome assembly and genomes used in this study. **Table S5.** Genes under positive selection pressure on the node of the family Uloboridae. **Table S6.** Transcriptome differential expression analysis of the legs of *Parasteatoda tepidariorum* and *Octonoba sinensis*. **Table S7.** Genes list of significantly expanded gene families in *Octonoba sinensis*. **Table S8.** New emergent gene family members in the *Octonoba sinensis* genome. **Table S9.** All HCEs, using the *Parasteatoda tepidariorum* genome as a reference. **Table S10.** Missing HCEs in *Octonoba sinensis* and *Uloborus diversus*. **Table S11.** Specific missing orthologous

groups in *Octonoba sinensis* and *Uloborus diversus*. **Table S12.** Genes under relaxed selection pressure in Uloboridae. **Table S13.** *Parasteatoda tepidariorum* venom gland-specific expression module. **Table S14.** Quantitative distribution of different toxin homologs across species. **Table S15.** "Pseudogene" blastx. **Table S16.** Protein domain search **Table S17.** The SRA data used for genome annotation. **Table S18.** Clusters of Reciprocal Best Hits.

Additional file 5: Figure S1. Recognizable elements of the *Octonoba sinensis* genome. **Figure S2.** Phylogenetic tree of homologs of neurotoxin genes (Latrotoxin).

Additional file 6: Expression Matrix of *Octonoba sinensis*.

Additional file 7: Dataset of toxin reference genes.

Acknowledgements

We are grateful to Prof. Zhonge Hou and Assis. Prof. Fengyuan Li for academic suggestions. We thank Wei Wang at Guangxi Normal University for her suggestions on anatomical techniques. Sincere thanks to Prof. Nadia Ayoub at W&L and Dr. Sandra Correa-Garhwal at American Museum of Natural History for their careful review and valuable suggestions on this article. Finally, Y.M.Z. wants to thank Lingling Liu, and in particular, for the invaluable support over the years.

Authors' contributions

S.Q.L. and Y.M.Z. conceived and designed the project. Y.X.S. and Y.M.Z. finished the genome assembly and annotation. B.Y.Z., P.Y.J., Y.X.S., and Y.M.Z executed the comparison analysis. Y.J.L. identified the species and provided the spider pictures used in the article. T.Y.J assembled and annotated transcriptome data of *Deinopis* sp. Y.W. recorded the original hunting videos, and Y.M.Z. edited the videos. X.T.H. sent a new transcriptome for testing and analysis. All authors participated in the discussion. The authors read and approved the final manuscript.

Funding

This study was supported by the Strategic Priority Research Program of the Chinese Academy of Sciences (XDB31000000).

Availability of data and materials

All data generated or analyzed during this study are included in this article, its supplementary information files, and publicly available repositories. Original sequencing data has been uploaded to the NCBI database (PRJNA1019401). New chromosome-level genome assemblies are deposited in the gigaDB Digital Repository ().

Declarations

Ethics approval and consent to participate

Not applicable.

Consent for publication

Not applicable.

Competing interests

The authors declare that they have no competing interests.

References:

[1] Foelix RF, Erb B. Mesothelae have venom glands., 2010.

[2] NMBE - World Spider Catalog., vol. 2023.

[3] Forster RR, Platnick NI. A review of the archaeid spiders and their relatives, with notes on the limits of the superfamily Palpimanoidea (Arachnida, Araneae). Bulletin of the AMNH ; v. 178, article 1., 1984.

[4] Rix MG. A Review of the Tasmanian Species of Pararchaeidae and Holarchaeidae (Arachnida, Araneae). The Journal of Arachnology 2005;33:135.

[5] Opell BD. Revision of the genera and tropical American species of the spider family Uloboridae. Bulletin of the Museum of Comparative Zoology at Harvard College 1979;148:443.

[6] Weng JL, Barrantes G, Eberhard WG. Feeding by Philoponella vicina (Araneae, Uloboridae) and how uloborid spiders lost their venom glands. CAN J ZOOL 2006;84:1752.

[7] Eberhard WG, Barrantes G, Weng JL. Tie them up tight: wrapping by Philoponella vicina spiders breaks, compresses and sometimes kills their prey. SCI NAT-HEIDELBERG 2006;93:251.

[8] LUBIN YD. Web buiding and prey capture in the Uloboridae. Spiders:Webs, Behavior, and Evolution. 1986:132.

[9] Opell BD. The relationship of book lung and tracheal systems in the spider family uloboridae. J MORPHOL 1990;206:211.

[10] Opell BD. The respiratory complementarity of spider book lung and tracheal systems. J MORPHOL 1998;236:57.

[11] de Plancy VC. Arachnides recueillis aux environs de Pékin. In: SIMON E, edito. Annales de la Société Entomologique de France. Saint-Germain, 1880.

[12] Robinson MH, Olazarri J. Units of behavior and complex sequences in the predatory behavior of Argiope argentata (Fabricius): (Araneae: Araneidae)., 1971.

[13] Rainer F. Biology of Spiders: Oxford university press, 2011.

[14] Wang X, Wang Y, Yang Z, Cui S. On the Karyotype of Octonnba sinensis. Journal of Hebei Normal University (Natural Science) 1997:423.

[15] Seppey M, Manni M, Zdobnov EM. BUSCO: Assessing Genome Assembly and Annotation Completeness. Methods Mol Biol 2019;1962:227.

[16] Simao FA, Waterhouse RM, Ioannidis P, Kriventseva EV, Zdobnov EM. BUSCO: assessing genome assembly and annotation completeness with single-copy orthologs. BIOINFORMATICS 2015;31:3210.

[17] Hu W, Jia A, Ma S, Zhang G, Wei Z, Lu F, Luo Y, Zhang Z, Sun J, Yang T, Xia T, Li Q, Yao T, Zheng J, Jiang Z, Xu Z, Xia Q, Wang Y. A molecular atlas reveals the tri-sectional spinning mechanism of spider dragline silk. NAT COMMUN 2023;14.

[18] Fan Z, Yuan T, Liu P, Wang L, Jin J, Zhang F, Zhang Z. A chromosome-level genome of the spiderTrichonephila antipodiana reveals the genetic basis of its polyphagy and evidence of an ancient whole-genome duplication event. GIGASCIENCE 2021;10.

[19] Liu S, Aagaard A, Bechsgaard J, Bilde T. DNA Methylation Patterns in the Social Spider, Stegodyphus dumicola. GENES-BASEL 2019;10:137.

[20] Huerta-Cepas J, Szklarczyk D, Heller D, Hernandez-Plaza A, Forslund SK, Cook H, Mende DR, Letunic I, Rattei T, Jensen LJ, von Mering C, Bork P. eggNOG 5.0: a hierarchical, functionally and phylogenetically annotated orthology resource based on 5090 organisms and 2502 viruses. NUCLEIC ACIDS RES 2019;47:D309.

[21] Magalhaes I, Azevedo G, Michalik P, Ramirez MJ. The fossil record of spiders revisited: implications for calibrating trees and evidence for a major faunal turnover since the Mesozoic. Biol Rev Camb Philos Soc 2019.

[22] Shao L, Zhao Z, Li S. Is phenotypic evolution affected by spiders' construction behaviors? SYST BIOL 2022.

[23] Wheeler WC, Coddington JA, Crowley LM, Dimitrov D, Goloboff PA, Griswold CE, Hormiga G, Prendini L, Ramirez MJ, Sierwald P, Almeida-Silva L, Alvarez-Padilla F, Arnedo MA, Benavides SL, Benjamin SP, Bond JE, Grismado CJ, Hasan E, Hedin M, Izquierdo MA, Labarque FM, Ledford J, Lopardo L, Maddison WP, Miller JA, Piacentini LN, Platnick NI, Polotow D, Silva-Davila D, Scharff N, Szuts T, Ubick D, Vink CJ, Wood HM, Zhang J. The spider tree of life: phylogeny of Araneae based on target-gene analyses from an extensive taxon sampling. CLADISTICS 2017;33:574.

[24] Matusek T, Djiane A, Jankovics F, Brunner D, Mlodzik M, Mihaly J. The Drosophila formin DAAM regulates the tracheal cuticle pattern through organizing the actin cytoskeleton. DEVELOPMENT 2006;133:957.

[25] Iskratsch T, Ehler E. Formin-g muscle cytoarchitecture. Bioarchitecture 2011;1:66.

[26] Valencia DA, Quinlan ME. Formins. CURR BIOL 2021;31:R517.

[27] Summers KM, Bush SJ, Davis MR, Hume DA, Keshvari S, West JA. Fibrillin-1 and asprosin, novel players in metabolic syndrome. MOL GENET METAB 2023;138:106979.

[28] Supek F, Bosnjak M, Skunca N, Smuc T. REVIGO summarizes and visualizes long lists of gene ontology terms. PLOS ONE 2011;6:e21800.

[29] Zhu B, Jin P, Zhang Y, Shen Y, Wang W, Li S. Genomic and transcriptomic analyses support a silk gland origin of spider venom glands. BMC BIOL 2023;21.

[30] Luo J, Ding Y, Peng Z, Chen K, Zhang X, Xiao T, Chen J. Molecular diversity and evolutionary trends of cysteine-rich peptides from the venom glands of Chinese spider Heteropoda venatoria. Sci Rep 2021;11:3211.

[31] Wang Z, Zhu K, Li H, Gao L, Huang H, Ren Y, Xiang H. Chromosome-level genome assembly of the black widow spider Latrodectus elegans illuminates composition and evolution of venom and silk proteins. GIGASCIENCE 2022;11.

[32] Zhu B, Jin P, Hou Z, Li J, Wei S, Li S. Chromosomal-level genome of a sheet-web spider provides insight into the composition and evolution of venom. MOL ECOL RESOUR 2022;22:2333.

[33] Escuer P, Pisarenco VA, Fernandez-Ruiz AA, Vizueta J, Sanchez-Herrero JF, Arnedo MA, Sanchez-Gracia A, Rozas J. The chromosome-scale assembly of the Canary Islands endemic spider Dysdera silvatica (Arachnida, Araneae) sheds light on the origin and genome structure of chemoreceptor gene families in chelicerates. MOL ECOL RESOUR 2022;22:375.

[34] Chen M, Blum D, Engelhard L, Raunser S, Wagner R, Gatsogiannis C. Molecular architecture of black widow spider neurotoxins. NAT COMMUN 2021;12:6956.

[35] Luddecke T, Herzig V, von Reumont BM, Vilcinskas A. The biology and evolution of spider venoms. Biol Rev Camb Philos Soc 2022;97:163.

[36] Zhang Z, Carriero N, Zheng D, Karro J, Harrison PM, Gerstein M. PseudoPipe: an automated pseudogene identification pipeline. BIOINFORMATICS 2006;22:1437.

[37] Protas ME, Trontelj P, Patel NH. Genetic basis of eye and pigment loss in the cave crustacean, *Asellus aquaticus*. Proceedings of the National Academy of Sciences 2011;108:5702.

[38] Gore AV, Tomins KA, Iben J, Ma L, Castranova D, Davis A, Parkhurst A, Jeffery WR, Weinstein BM. An epigenetic mechanism for cavefish eye degeneration. bioRxiv 2017:199018.

[39] Mojaddidi H, Fernandez FE, Erickson PA, Protas ME. Embryonic origin and genetic basis of cave associated phenotypes in the isopod crustacean Asellus aquaticus. SCI REP-UK 2018;8:16589.

[40] Piatigorsky J. A Genetic Perspective on Eye Evolution: Gene Sharing, Convergence and Parallelism. Evolution: Education and Outreach 2008;1:403.

[41] Krishnan J, Rohner N. Cavefish and the basis for eye loss. Philos Trans R Soc Lond B Biol Sci 2017;372.

[42] Saxena A, Cooper KL. Diversification of the vertebrate limb: sequencing the events. CURR OPIN GENET DEV 2021;69:42.

[43] Yi H. How Snakes Came to Slither. SCI AM 2017;318:70.

[44] Mann A, Pardo JD, Maddin HC. Snake-like limb loss in a Carboniferous amniote. NAT ECOL EVOL 2022;6:614.

[45] Morgenstern D, King GF. The venom optimization hypothesis revisited. TOXICON 2013;63:120.

[46] Bordon K, Cologna CT, Fornari-Baldo EC, Pinheiro-Junior EL, Cerni FA, Amorim FG, Anjolette F, Cordeiro FA, Wiezel GA, Cardoso IA, Ferreira IG, de Oliveira IS, Boldrini-Franca J, Pucca MB, Baldo MA, Arantes EC. From Animal Poisons and Venoms to Medicines: Achievements, Challenges and Perspectives in Drug Discovery. FRONT PHARMACOL 2020;11:1132.

[47] Utkin YN. Animal venom studies: Current benefits and future developments. World J Biol Chem 2015;6:28.

[48] Shao L, Li S. Early Cretaceous greenhouse pumped higher taxa diversification in spiders. MOL PHYLOGENET EVOL 2018;127:146.
[49] Rainer F, Bruno E. Mesothelae have venom glands. The Journal of Arachnology 2010;38:596.
[50] Bolger AM, Lohse M, Usadel B. Trimmomatic: a flexible trimmer for Illumina sequence data. BIOINFORMATICS 2014;30:2114.
[51] Wenger AM, Peluso P, Rowell WJ, Chang PC, Hall RJ, Concepcion GT, Ebler J, Fungtammasan A, Kolesnikov A, Olson ND, Topfer A, Alonge M, Mahmoud M, Qian Y, Chin CS, Phillippy AM, Schatz MC, Myers G, DePristo MA, Ruan J, Marschall T, Sedlazeck FJ, Zook JM, Li H, Koren S, Carroll A, Rank DR, Hunkapiller MW. Accurate circular consensus long-read sequencing improves variant detection and assembly of a human genome. NAT BIOTECHNOL 2019;37:1155.
[52] Lu L, Liu X, Huang W, Giusti-Rodríguez P, Cui J, Zhang S, Xu W, Wen Z, Ma S, Rosen JD, Xu Z, Bartels CF, Kawaguchi R, Hu M, Scacheri PC, Rong Z, Li Y, Sullivan PF, Song H, Ming G, Li Y, Jin F. Robust Hi-C Maps of Enhancer-Promoter Interactions Reveal the Function of Non-coding Genome in Neural Development and Diseases. MOL CELL 2020;79:521.
[53] Rao SS, Huntley MH, Durand NC, Stamenova EK, Bochkov ID, Robinson JT, Sanborn AL, Machol I, Omer AD, Lander ES, Aiden EL. A 3D map of the human genome at kilobase resolution reveals principles of chromatin looping. CELL 2014;159:1665.
[54] Meyer M, Kircher M. Illumina sequencing library preparation for highly multiplexed target capture and sequencing. Cold Spring Harb Protoc 2010;2010:t5448.
[55] Durand NC, Shamim MS, Machol I, Rao SS, Huntley MH, Lander ES, Aiden EL. Juicer Provides a One-Click System for Analyzing Loop-Resolution Hi-C Experiments. CELL SYST 2016;3:95.
[56] Marcais G, Kingsford C. A fast, lock-free approach for efficient parallel counting of occurrences of k-mers. BIOINFORMATICS 2011;27:764.
[57] Cheng H, Concepcion GT, Feng X, Zhang H, Li H. Haplotype-resolved de novo assembly using phased assembly graphs with hifiasm. NAT METHODS 2021;18:170.
[58] Ruan J, Li H. Fast and accurate long-read assembly with wtdbg2. NAT METHODS 2020;17:155.
[59] Hu J, Fan J, Sun Z, Liu S. NextPolish: a fast and efficient genome polishing tool for long-read assembly. BIOINFORMATICS 2020;36:2253.
[60] Li H. Minimap2: pairwise alignment for nucleotide sequences. BIOINFORMATICS 2018;34:3094.
[61] Li H, Handsaker B, Wysoker A, Fennell T, Ruan J, Homer N, Marth G, Abecasis G, Durbin R. The Sequence Alignment/Map format and SAMtools. BIOINFORMATICS 2009;25:2078.
[62] Dudchenko O, Batra SS, Omer AD, Nyquist SK, Hoeger M, Durand NC, Shamim MS, Machol I, Lander ES, Aiden AP, Aiden EL. De novo assembly of the Aedes aegypti genome using Hi-C yields chromosome-length scaffolds. SCIENCE 2017;356:92.
[63] Dudchenko O, Shamim MS, Batra S, Durand NC, Musial NT, Mostofa R, Pham M, St Hilaire BG, Yao W, Stamenova E, Hoeger M, Nyquist SK, Korchina V, Pletch K, Flanagan JP, Tomaszewicz A, Mcaloose D, Cynthia PRE, Novak BJ, Omer AD, Aiden EL. The Juicebox Assembly Tools module facilitates de novo assembly of mammalian genomes with chromosome-length scaffolds for under $1000. Cold Spring Harbor: Cold Spring Harbor Laboratory Press, 2018.
[64] Flynn JM, Hubley R, Goubert C, Rosen J, Clark AG, Feschotte C, Smit AF. RepeatModeler2 for automated genomic discovery of transposable element families. Proc Natl Acad Sci U S A 2020;117:9451.

[65] Tarailo-Graovac M, Chen N. Using RepeatMasker to identify repetitive elements in genomic sequences. Curr Protoc Bioinformatics 2009;Chapter 4:4.
[66] Bruna T, Hoff KJ, Lomsadze A, Stanke M, Borodovsky M. BRAKER2: automatic eukaryotic genome annotation with GeneMark-EP+ and AUGUSTUS supported by a protein database. NAR Genom Bioinform 2021;3:a108.
[67] Chan PP, Lin BY, Mak AJ, Lowe TM. tRNAscan-SE 2.0: improved detection and functional classification of transfer RNA genes. NUCLEIC ACIDS RES 2021;49:9077.
[68] Kalvari I, Nawrocki EP, Ontiveros-Palacios N, Argasinska J, Lamkiewicz K, Marz M, Griffiths-Jones S, Toffano-Nioche C, Gautheret D, Weinberg Z, Rivas E, Eddy SR, Finn RD, Bateman A, Petrov AI. Rfam 14: expanded coverage of metagenomic, viral and microRNA families. NUCLEIC ACIDS RES 2021;49:D192.
[69] Nawrocki EP, Eddy SR. Infernal 1.1: 100-fold faster RNA homology searches. BIOINFORMATICS 2013;29:2933.
[70] Chen S, Zhou Y, Chen Y, Gu J. fastp: an ultra-fast all-in-one FASTQ preprocessor. BIOINFORMATICS 2018;34:i884.
[71] Grabherr MG, Haas BJ, Yassour M, Levin JZ, Thompson DA, Amit I, Adiconis X, Fan L, Raychowdhury R, Zeng Q, Chen Z, Mauceli E, Hacohen N, Gnirke A, Rhind N, di Palma F, Birren BW, Nusbaum C, Lindblad-Toh K, Friedman N, Regev A. Full-length transcriptome assembly from RNA-Seq data without a reference genome. NAT BIOTECHNOL 2011;29:644.
[72] Li W, Godzik A. Cd-hit: a fast program for clustering and comparing large sets of protein or nucleotide sequences. BIOINFORMATICS 2006;22:1658.
[73] Emms DM, Kelly S. OrthoFinder: phylogenetic orthology inference for comparative genomics. GENOME BIOL 2019;20:238.
[74] Katoh K, Standley DM. MAFFT multiple sequence alignment software version 7: improvements in performance and usability. MOL BIOL EVOL 2013;30:772.
[75] Camacho C, Coulouris G, Avagyan V, Ma N, Papadopoulos J, Bealer K, Madden TL. BLAST+: architecture and applications. BMC BIOINFORMATICS 2009;10:421.
[76] Minh BQ, Schmidt HA, Chernomor O, Schrempf D, Woodhams MD, von Haeseler A, Lanfear R. IQ-TREE 2: New Models and Efficient Methods for Phylogenetic Inference in the Genomic Era. MOL BIOL EVOL 2020;37:1530.
[77] Tang H, Bowers JE, Wang X, Ming R, Alam M, Paterson AH. Synteny and collinearity in plant genomes. SCIENCE 2008;320:486.
[78] Yang Z. PAML 4: phylogenetic analysis by maximum likelihood. MOL BIOL EVOL 2007;24:1586.
[79] Kosakovsky PS, Poon A, Velazquez R, Weaver S, Hepler NL, Murrell B, Shank SD, Magalis BR, Bouvier D, Nekrutenko A, Wisotsky S, Spielman SJ, Frost S, Muse SV. HyPhy 2.5-A Customizable Platform for Evolutionary Hypothesis Testing Using Phylogenies. MOL BIOL EVOL 2020;37:295.
[80] De Bie T, Cristianini N, Demuth JP, Hahn MW. CAFE: a computational tool for the study of gene family evolution. BIOINFORMATICS 2006;22:1269.
[81] Harris RS. IMPROVED PAIRWISE ALIGNMENT OF GENOMIC DNA., vol. Doctor of Philosophy: The Pennsylvania State University, 2007.
[82] Kent WJ, Baertsch R, Hinrichs A, Miller W, Haussler D. Evolution's cauldron: duplication, deletion,

and rearrangement in the mouse and human genomes. Proc Natl Acad Sci U S A 2003;100:11484.

[83] Blanchette M, Kent WJ, Riemer C, Elnitski L, Smit AF, Roskin KM, Baertsch R, Rosenbloom K, Clawson H, Green ED, Haussler D, Miller W. Aligning multiple genomic sequences with the threaded blockset aligner. GENOME RES 2004;14:708.

[84] Hubisz MJ, Pollard KS, Siepel A. PHAST and RPHAST: phylogenetic analysis with space/time models. BRIEF BIOINFORM 2011;12:41.

[85] Siepel A, Bejerano G, Pedersen JS, Hinrichs AS, Hou M, Rosenbloom K, Clawson H, Spieth J, Hillier LW, Richards S, Weinstock GM, Wilson RK, Gibbs RA, Kent WJ, Miller W, Haussler D. Evolutionarily conserved elements in vertebrate, insect, worm, and yeast genomes. GENOME RES 2005;15:1034.

[86] Yang H, Wang K. Genomic variant annotation and prioritization with ANNOVAR and wANNOVAR. NAT PROTOC 2015;10:1556.

[87] Wang K, Li M, Hakonarson H. ANNOVAR: functional annotation of genetic variants from high-throughput sequencing data. NUCLEIC ACIDS RES 2010;38:e164.

[88] Haney RA, Matte T, Forsyth FS, Garb JE. Alternative Transcription at Venom Genes and Its Role as a Complementary Mechanism for the Generation of Venom Complexity in the Common House Spider. Front Ecol Evol 2019;7.

[89] Pineda SS, Chaumeil PA, Kunert A, Kaas Q, Thang M, Le L, Nuhn M, Herzig V, Saez NJ, Cristofori-Armstrong B, Anangi R, Senff S, Gorse D, King GF. ArachnoServer 3.0: an online resource for automated discovery, analysis and annotation of spider toxins. BIOINFORMATICS 2018;34:1074.

[90] Wang Z, Zhu K, Li H, Gao L, Huang H, Ren Y, Xiang H. Chromosome-level genome assembly of the black widow spider Latrodectus elegans illuminates composition and evolution of venom and silk proteins. GIGASCIENCE 2022;11.

[91] Mistry J, Finn RD, Eddy SR, Bateman A, Punta M. Challenges in homology search: HMMER3 and convergent evolution of coiled-coil regions. NUCLEIC ACIDS RES 2013;41:e121.

[92] Roberts SP, Harrison JF, Dudley R. Allometry of kinematics and energetics in carpenter bees (Xylocopa varipuncta) hovering in variable-density gases. J EXP BIOL 2004;207:993.

Figure1 Click here to access/download;Figure;Fig.1-giga.jpg

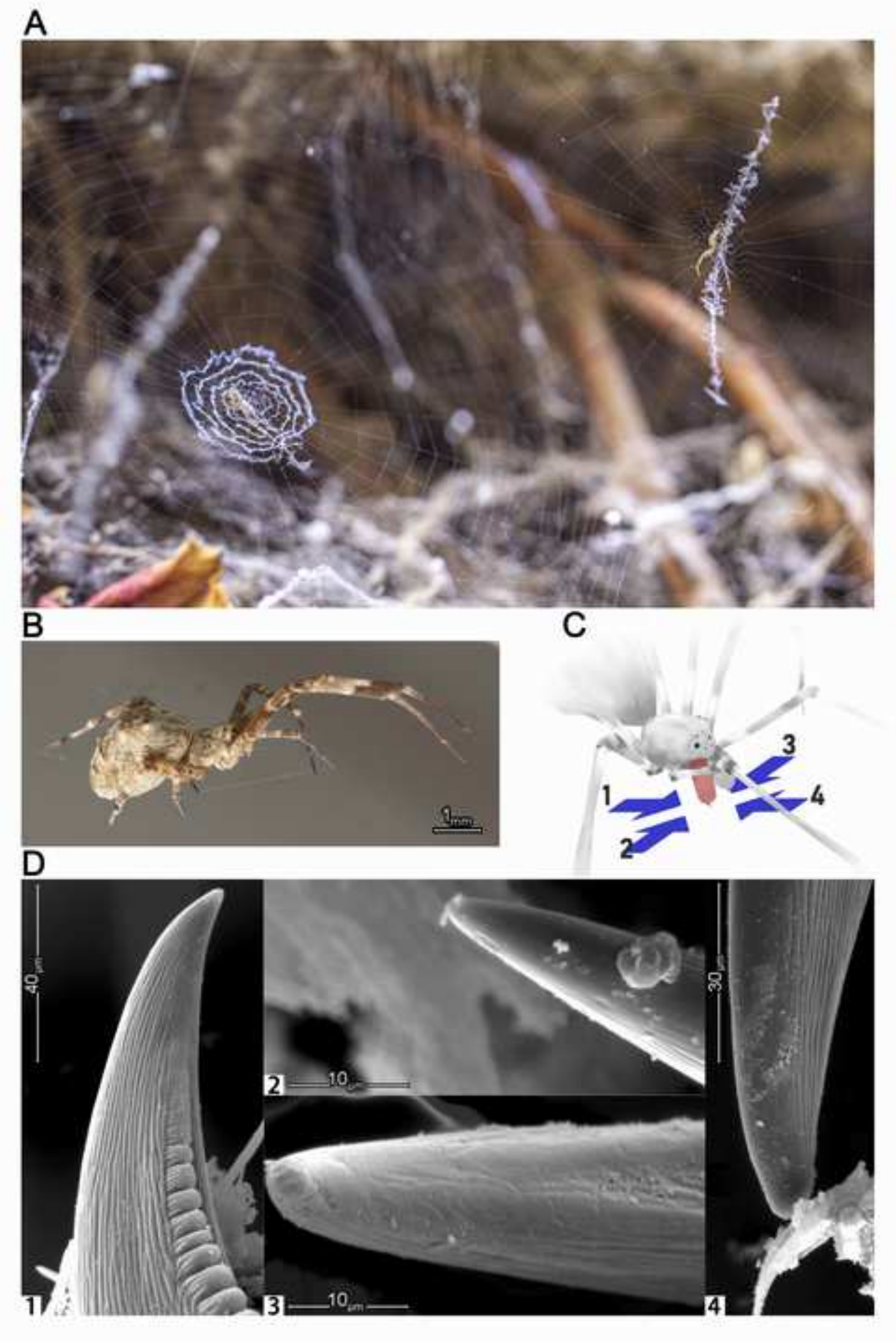

Figure2

Click here to access/download;Figure;Fig.2-giga.jpg

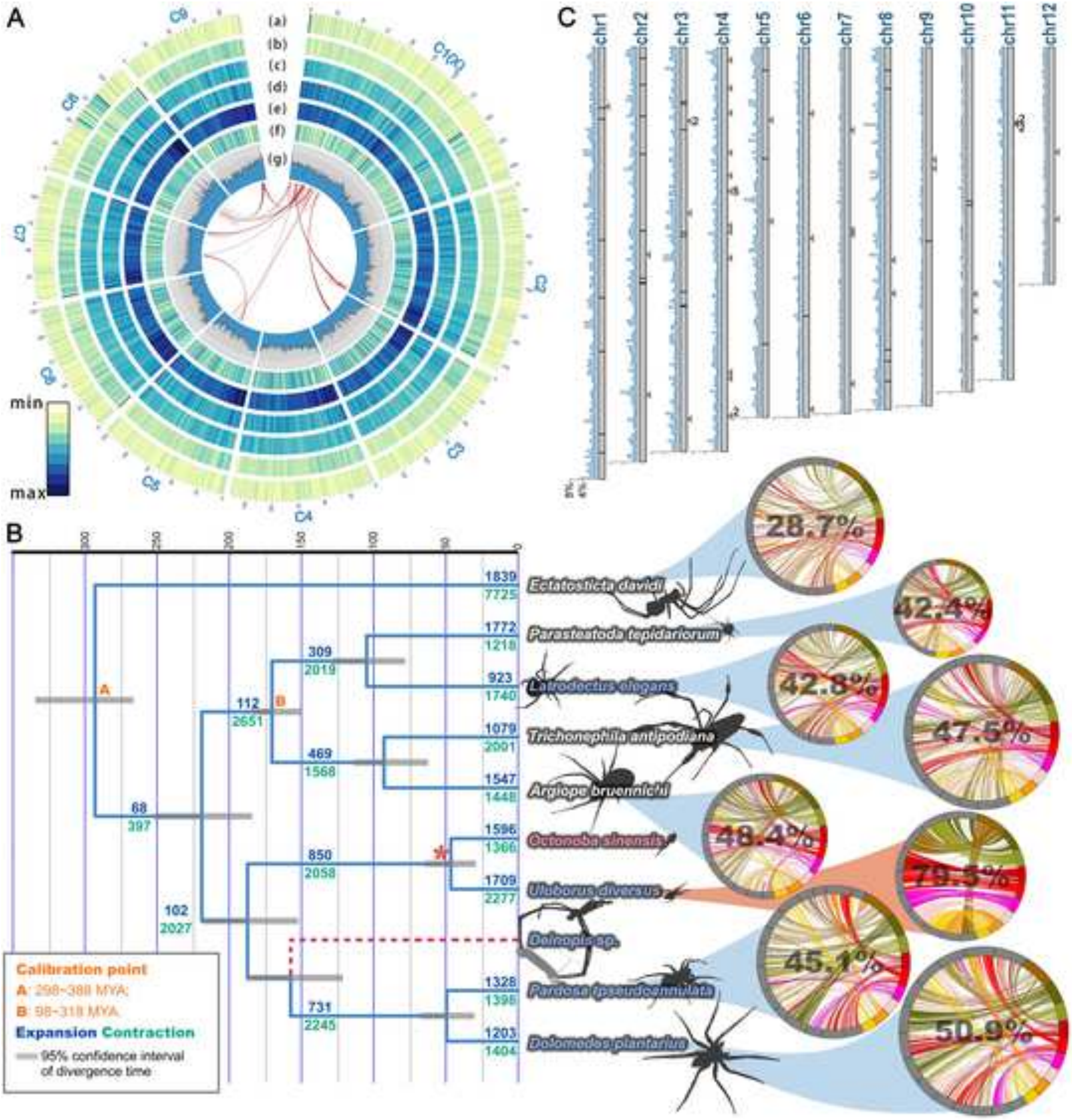

Figure3 Click here to access/download;Figure;Fig.3-giga.jpg

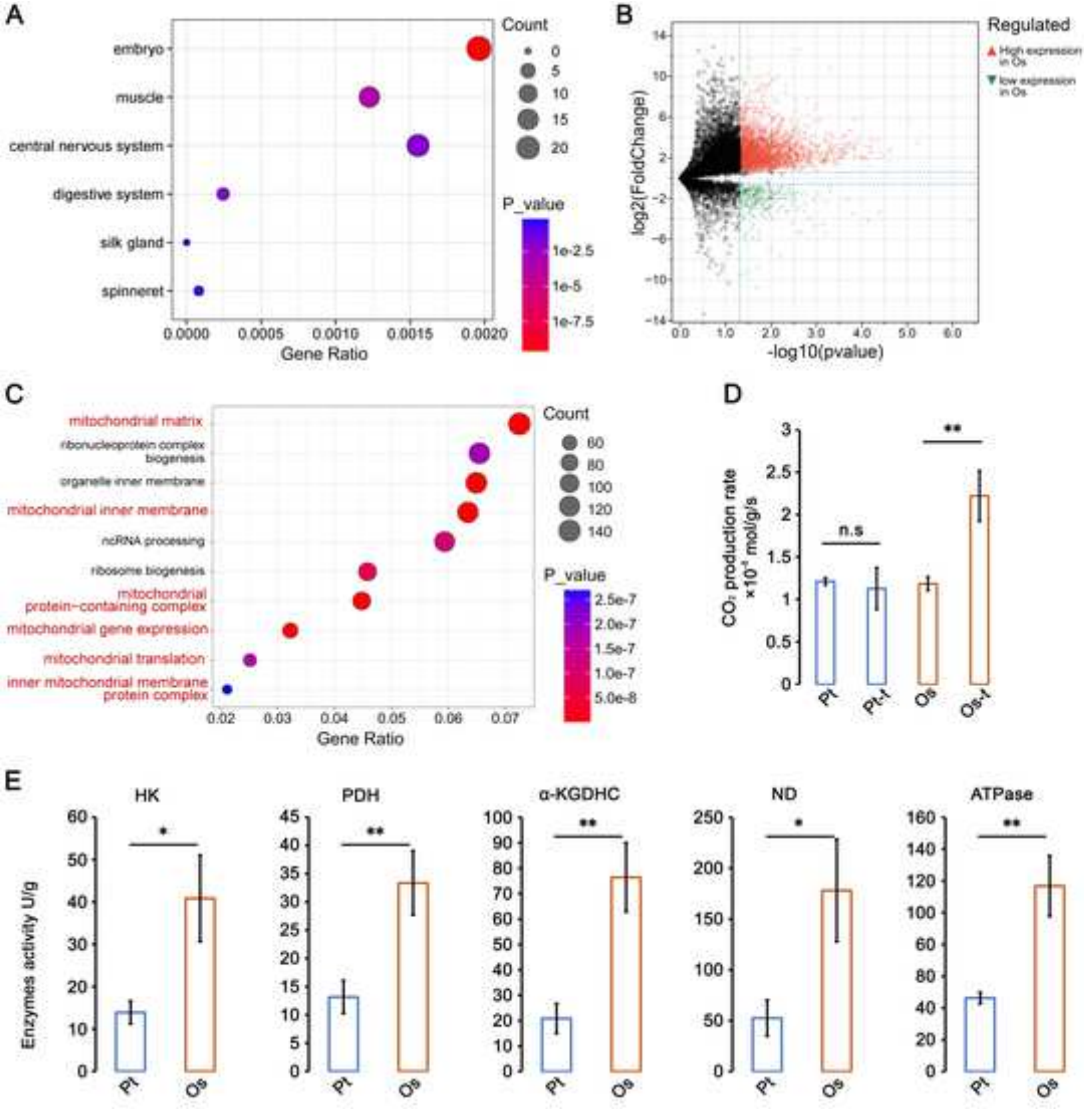

Figure4

Click here to access/download;Figure;Fig.4-giga.jpg

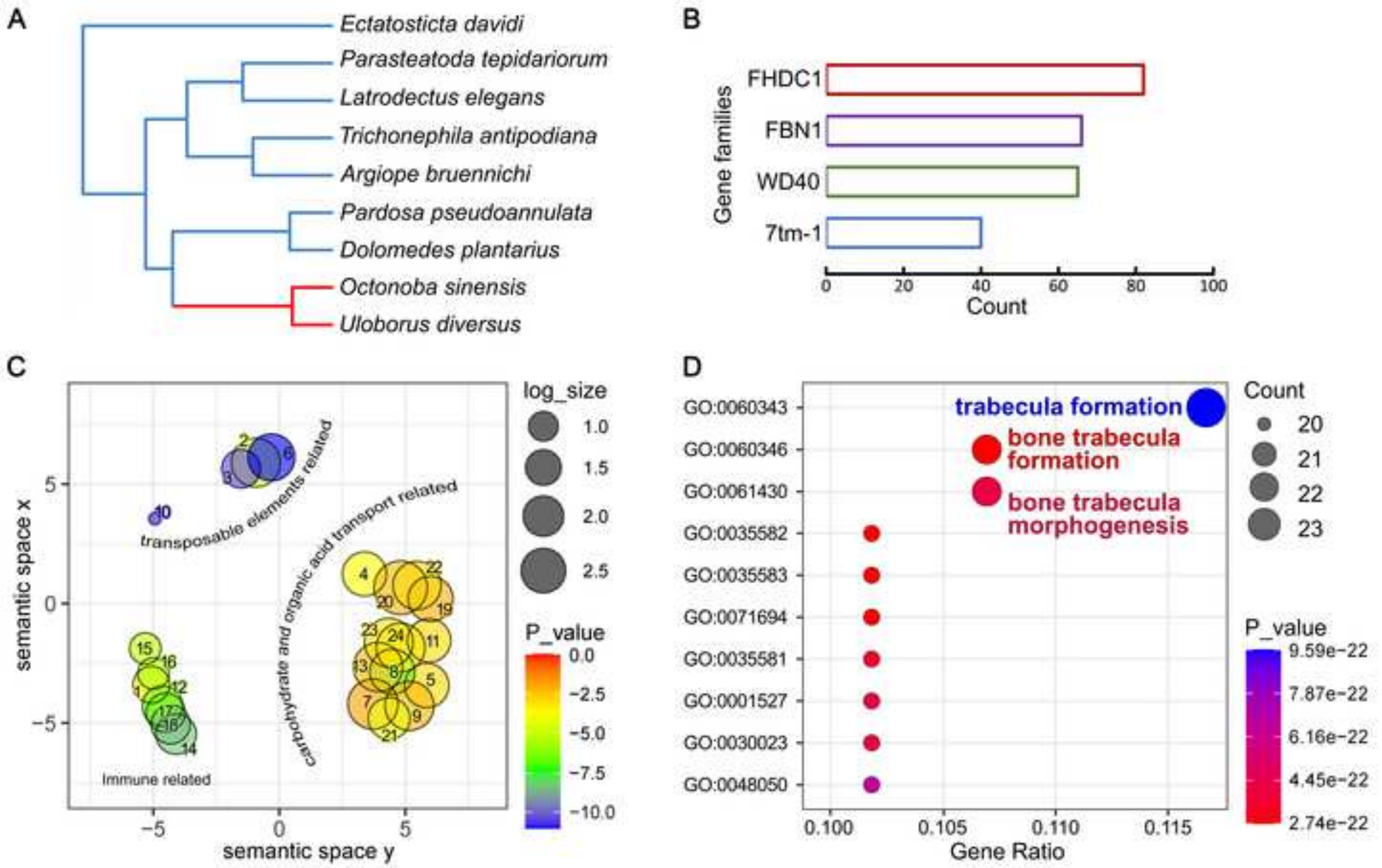

Figure5

Click here to access/download;Figure;Fig.5-giga.jpg

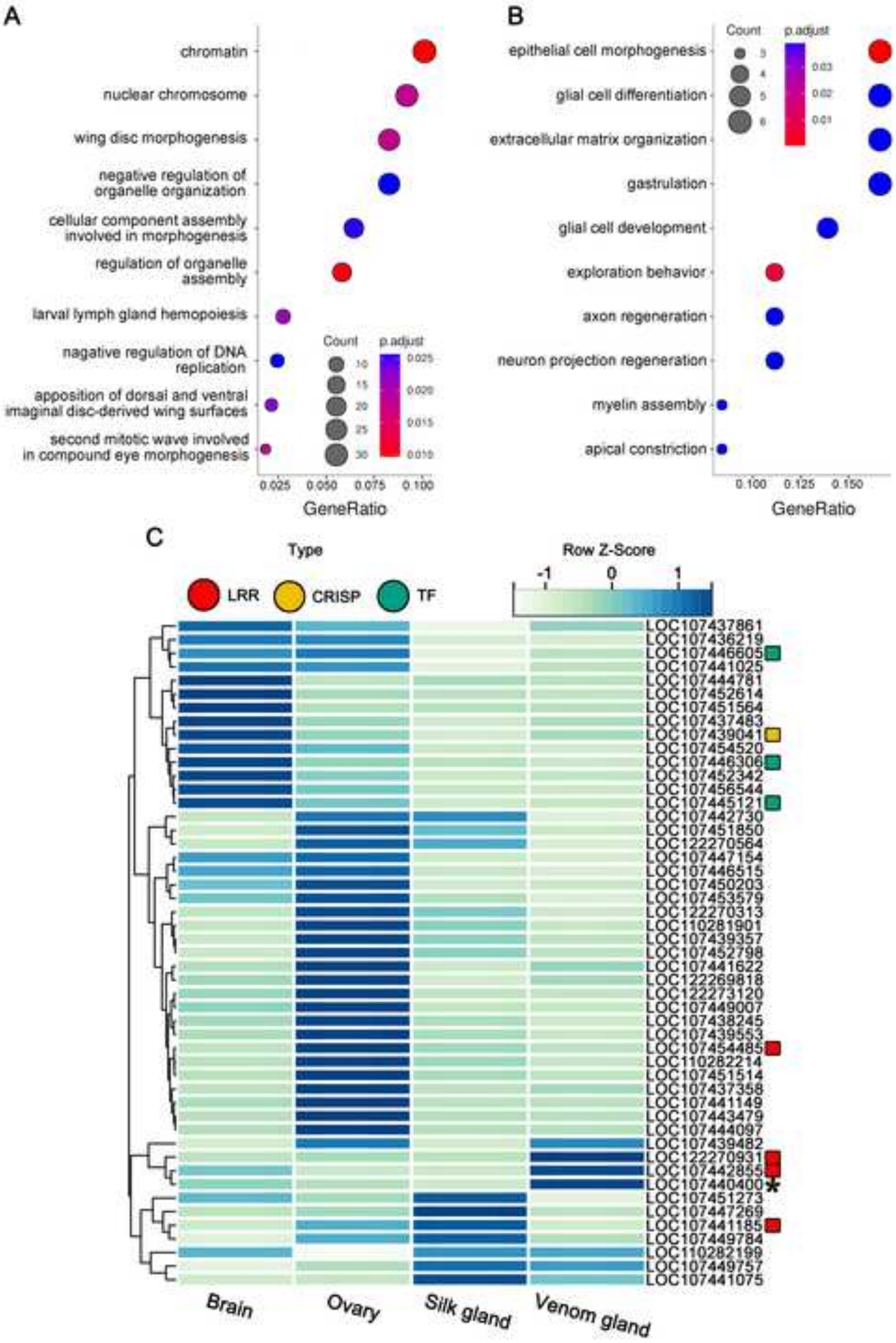

Figure6

Click here to access/download;Figure;Fig.6-giga.jpg

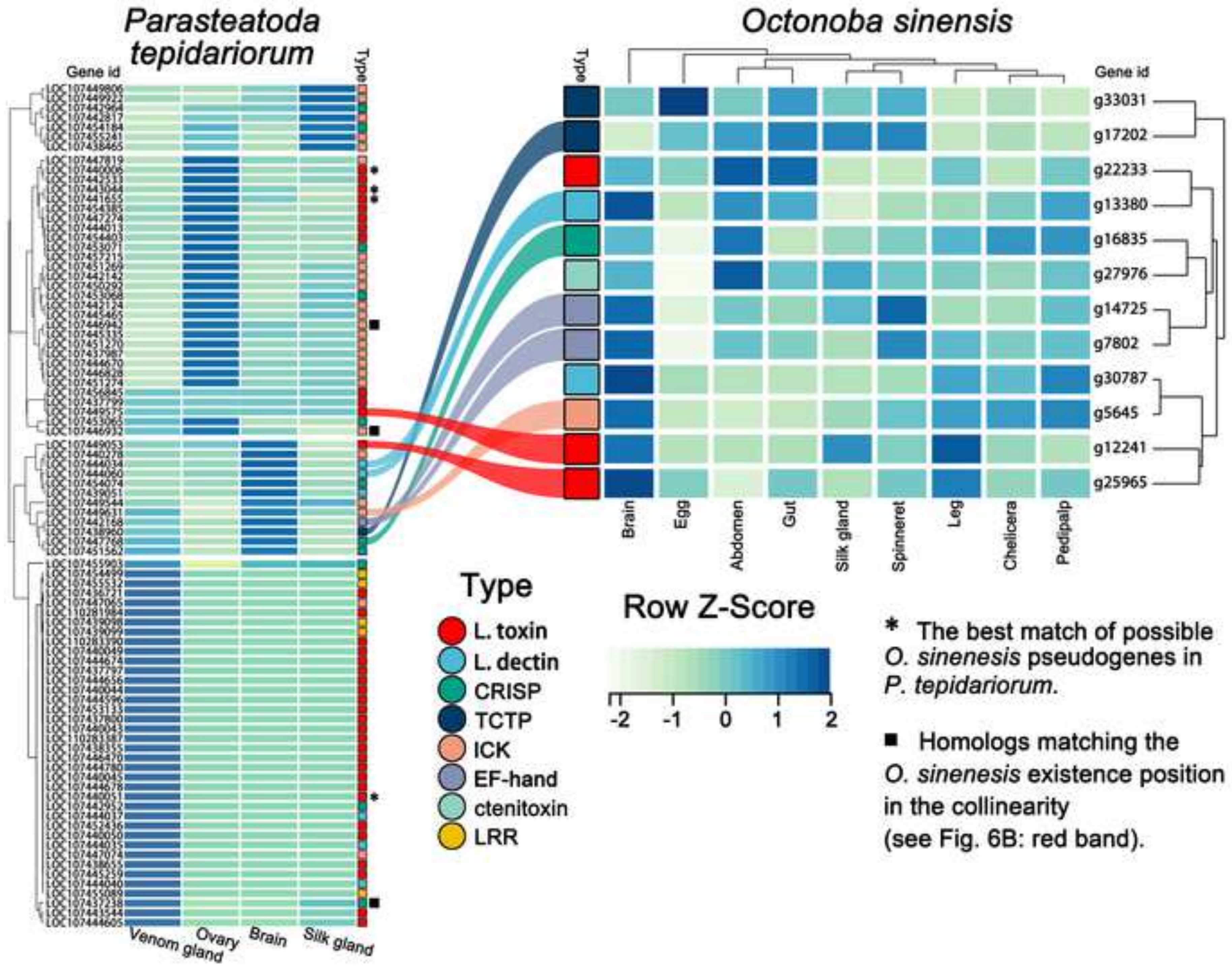

Figure7

Click here to access/download;Figure;Fig.7-giga.jpg

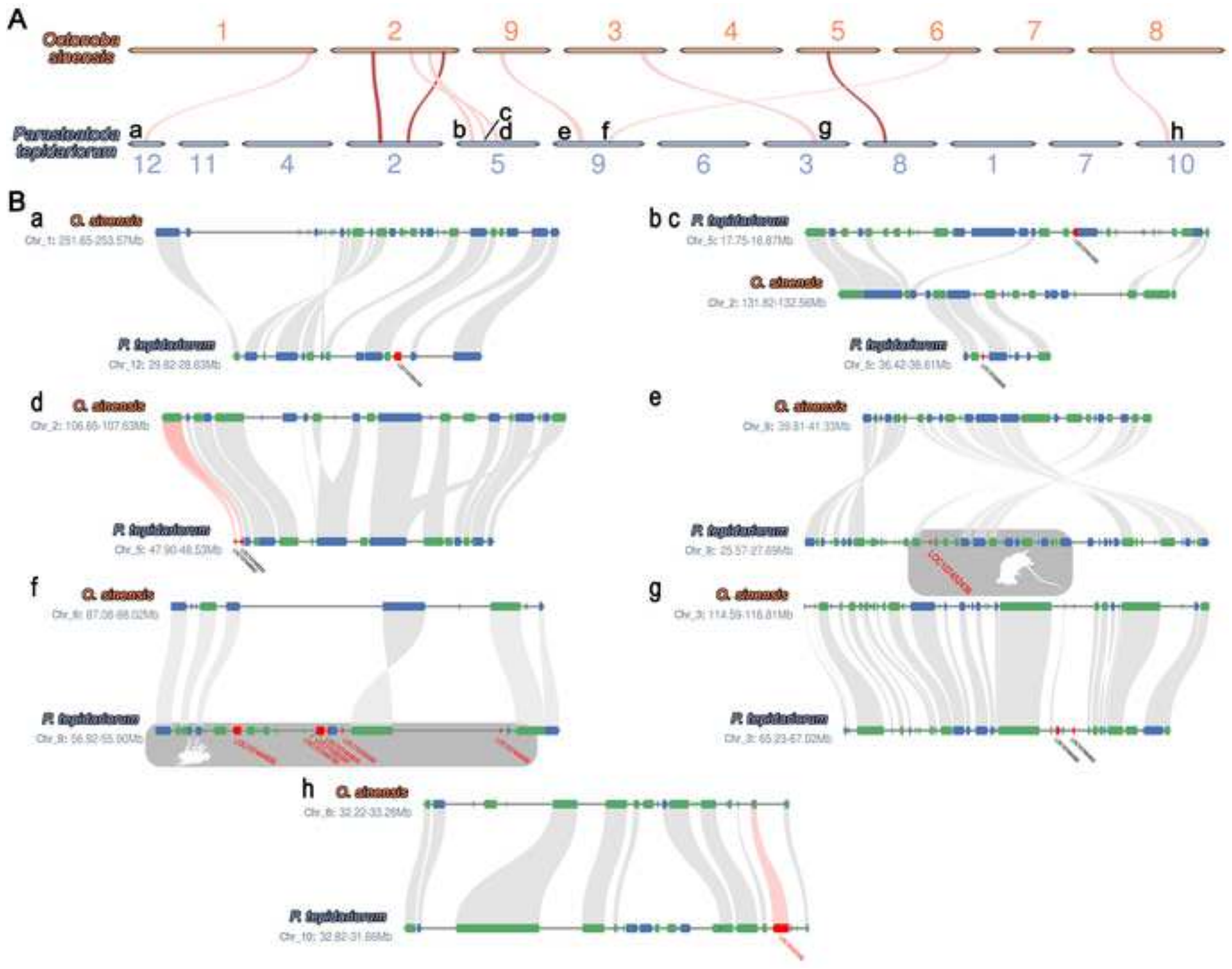

Additional file 1

Click here to access/download
Supplementary Material
Additional file 1.mp4

Additional file 2

Click here to access/download
Supplementary Material
Additional file 2.mp4

Additional file 3

Click here to access/download
Supplementary Material
Additional file 3.mp4

Additional file4

Click here to access/download
Supplementary Material
Additional file 4-giga.xlsx

Additional file5

Click here to access/download
Supplementary Material
Additional file 5-giga.docx

Additional file6

Click here to access/download
Supplementary Material
Additional file 6-giga.xls

Personal Cover Click here to access/download;Personal Cover;Cover letter lfy.doc

中國科學院動物研究所
INSTITUTE OF ZOOLOGY,CHINESE ACADEMY OF SCIENCES

August 31th, 2023

Scott Edmunds, Ph.D.
Editors-in-Chief, GigaScience
GigaScience Press

Dear Editors:

We are pleased to submit our manuscript entitled "A Trade-off in Evolution: The Adaptive Landscape of Spiders without Venom Glands" for consideration in *GigaScience*.

The evolution of traits in a trade-off fashion propounded by Charles Darwin in The Origin of Species is a very important subject. However, our understanding of trade-offs in evolutionary biology remains poor. Uloboridae is one of the venom gland deficient of spiders. Trade-offs between lack of venom glands and a laborious prey wrapping technique in *Octonoba sinensis* (Araneae, Uloboridae) provide us a classic example to understand the trade-off strategy.

Genome sequences from *O. sinensis* reveal that the evolution of energy metabolism pathways is the key factor in their sustained power output. Comparative genomics and transcriptomics suggest that the absence of regions and regions under relaxed selection in *O. sinensis* are concentrated in the field of development, and the number of toxin genes expressed in *O. sinensis* are less than in most spider species.

We combined multi-omics and functional approaches to uncover the evolution of adaptation strategies in the Uloboridae and provide insights into the mechanism underlying this trade-off between different predation strategies. Our study shows that building the organisms is the inter-dependence between sets of traits when one part modified through natural selection, other parts become varied.

Neither the manuscript nor any part of it has been published or is under consideration for review or publication elsewhere. All authors have directly participated in the planning, execution, or analysis of this study, and have read and approved the final version submitted. There is no conflict of interest related to the manuscript.

We believe that this work is well-suited for the broad readership of *GigaScience*. Thank you for receiving our manuscript and considering it for review. We greatly appreciate your time and look forward to your response.

Yours sincerely,

Shuqiang Li, Ph.D.

Professor, Key Laboratory of Zoological Systematics and Evolution

Institute of Zoology, Chinese Academy of Sciences

Beijing 100101, China

Response to reviewers Click here to access/download;Personal Cover;Response to reviewers.docx

Response to reviewers

Dear Zhang

Thanks very much for handling our manuscript (GIGA-D-23-00275) submitted to *GigaScience*. We greatly appreciate the profound and constructive comments from you and the two reviewers, which are very helpful to improve our manuscript. We have been able to incorporate changes to reflect the suggestions provided by the reviewers. all he changes are highlighted within the manuscript. We believed that we have addressed all the concerns from the reviewers. A point-by-point response to reviewers' comments is enclosed.

Sincerely,

Yiming Zhang; Shuqiang Li

Institute of Zoology, Chinese Academy of Sciences

1. Beichen West Road, Chaoyang District

Beijing 100101, P. R. China

Tel: +86-10-64807216

Fax: +86-10-64807216

Email: zhangyiming@ioz.ac.cn; lisq@ioz.ac.cn

Editor comments

As the reviewer mentioned that the genome of Uloborus diversus should be included in the manuscript.

>>>**Response:** Thanks for your suggestions. We added this genome to the new analysis and supplemented the annotation, increasing the number of coding genes with transcriptome support from 15750 to 19036. Subsequently, we also added some genomes proposed by reviewers to the new analysis and conducted all downstream analyses based on these data.

Reviewer #1:

Zhang et al. provide a chromosome level assembly of *Octonoba sinensis*, a member of the one and only family of spiders that does not possess venom glands. The sequence and annotation is highly valuable for arachnologists and arthropod biologists more generally. The manuscript also provides some interesting analyses, focusing on potential gene loss associated with loss of venom glands. However, I have concerns that need to be addressed prior to publication.

>>>**Response:** Thank you for your positive assessment of our work. In this revision, we have strengthened the exploration of the trade-offs between motor function and the absence of venom glands. In addition to some highlights in the original manuscript, new discoveries have also emerged under a new comparative system. These results provide a more comprehensive characterization of the adaptive evolution of this group under the absence of venom glands.

1. First, the closest relative included in the gene family analyses, *Stegodyphus dumicola* (Eresidae), is quite distantly related from Uloboridae. Thus, inferences about gene loss or positive selection associated with the loss of venom glands are quite difficult to make with confidence. At a minimum, the authors need to clarify the lack of inclusion of any other members of the UDOH grade. As written (lines 180-183), the authors insinuate that these close relatives are included in the analysis of gene loss and positive/relaxed selection. This is not true. Although, there are no genomes currently available for members of Deinopidae, Oecobiidae, Hersiliidae, there are transcriptomes. The authors could consider an additional analysis using these transcriptomes to track gene loss.

>>>**Response:** Thanks for your suggestions. Based on your suggestion, we assembled the transcriptome of an Ogre-faced spider (*Deinopis* sp.), and obtained a total protein Busco score of 93.1% (**see Fig. 2B, 3A and Methods: 6. Genome annotation**). We used this portion of data to enrich selection pressure analysis and assist in searching for specific gene deletions and emergence.

2. Related to the above comment, a high quality chromosome-level assembly is available for *Uloborus diversus*. I strongly recommend including Uloborus in synteny and gene family analyses. As another uloborid species, its inclusion would bolster inferences about gene loss in Uloboridae or positive selection/relaxed selection in Uloboridae, and not just in Octonoba.

>>>**Response:** Thanks for your comments. We added this genome to the new analysis (**see Fig. 2B, 3A**) and supplemented the annotation, increasing the number of coding genes with transcriptome support from 15750 to 19036. Subsequently, we also added some genomes proposed by reviewers to the new analysis and conducted all downstream analyses based on these data.

3. Related to above, there are three genomes available for RTA clade species in NCBI. It doesn't look like there are associated publications yet, but the authors might consider inclusion to break up the evolutionary distance between Uloboridae and other species currently in the analysis; if the authors are set on only using species with whole genomes.

>>>**Response:** Based on your suggestion, we have annotated the *Pardosa pseudoannulata* and *Dolomedes plantarius* genomes, and the total protein Busco scores reached 93.6% and 95.0%, respectively. In addition, we replaced the *Dysdera silvatica* genome with the newly published *Ectatosticta davidi* genome as the basal group, and re-annotated the *Latrodectus elegans* genome, resulting in an increase in its protein Busco score from 63.7% to 96.5%. In the end, all of these genomes have a protein Busco score of over 90% (**Additional file 4: Table S2**), and a more suitable evolutionary tree was obtained (**see Fig. 2B, 3A**).

4. For positive selection analyses, is finding metabolic genes unusual? E.g. if the same analysis was done moving a species with venom glands to the target would metabolic genes still arise? Perhaps a different set of genes, but could be something more general to arachnids/arthropods?

>>>**Response:** Based on your suggestion, using the new dataset mentioned above, we conducted a new positive selection analysis using the nodes of the Uloboridae family, where the *O. sinensis* and *U. diversus* genomes are located, as the foreground branch. Unfortunately, the new significant positive selection gene set does not support enrichment

in energy metabolism pathways (**see Results: 4. Genes under positive selection and energy metabolism in muscle**). However, in comparative transcriptome analysis of legs of different species, genes highly expressed in the *O. sinensis* can be significantly enriched in mitochondrial related GO entries (**Fig. 3C**).

5. The relevance of the "absent" regions and regions under relaxed selection wasn't clear. Was there a specific hypothesis being tested? E.g. that toxin genes are more likely to be lost or under relaxed selection? Clarify.

>>>**Response:** Thanks for your comments. We speculate that due to the absence of venom glands, the genes or functional regions specifically involved in the venom gland system in the Uloboridae family may be subjected to relaxed purifying selection or gradually lost from genome. In order to obtain this information, Highly-Conserved Elements (HCEs), absent genes and genes under selective relaxation in the *O. sinensis* and *U. diversus* genome were searched and identified. We have added the relevant description to the "**Results: 6. Absent regions and genes under relaxed purifying selection**" to make it clearer.

6. Similarly, the point of the SNP analysis wasn't clear. Was there a driving hypothesis? What is learned from the analysis?

>>>**Response:** Thanks for your comments. The distribution of SNPs may reflect information from recent evolutionary processes or divergence characteristics between closely related lineage. It may not properly reflect the evolution information on a larger time scale (such as in this study) (doi: 10.1093/gbe/evz128). Therefore, we have deleted this section in the revised manuscript.

7. How were venom specific modules identified in Parasteatoda? If the authors generated their own RNA seq for Parasteatoda, please include accessions.

>>>**Response:** Thank you for pointing this out. This “venom specific modules” was the result of our previous work (doi: 10.1186/s12915-023-01581-7). It was obtained through weighted correlation network analysis using transcriptome data from different tissues, and the relevant results have been published. Here, we directly cite this result (**see reference 29**).

In the revised manuscript, we have generated transcriptome data for legs from Parasteatoda, while remaining transcriptome data of the Parasteatoda are sourced from the aforementioned references. We have provided clarification in the revised manuscript (**see Methods: 5. RNA extraction and sequencing**).

8. I'm not convinced latrotoxins exist outside of Theridiidae. Is it possible the significant BLAST alignments are based on ankyrin repeats found in latrotoxins and other types of genes? Please provide more evidence in support of latrotoxins in distant relatives of Latrodectus.

>>>**Response:** Thank you for your reminder. You have raised an important point here. The toxin gene homologs we obtained through our search were all based on the results of blast analysis using the spider toxin database annotated by previous researchers.

In the revised manuscript, we also established the hidden Markov models (HMM) for different types of toxin proteins based on the database, and further confirmed the results obtained from blastp using HMMER v 3.3. The confirmation of any toxin protein requires strict toxicology or virulence testing. In the revised manuscript, we refer to the target gene obtained through our search as a toxin gene homolog. Based on this, we have also revised some of the less rigorous viewpoints in the discussion. Thank you again for your prompt.

9. Language and readability were mostly fine, but the manuscript could use editing in many places-too many for me to enumerate. One item that jumped out was the use of "losed" for "lost" in some figures. Please correct and have someone read the whole manuscript carefully.

>>>**Response:** Thank you, we will do our best to review and revise the manuscript.

Reviewer #2:

The manuscript titled "A Trade-off in Evolution: The Adaptive Landscape of Spiders without Venom Glands" described the a comprehensive study of the spider *Octonoba sinensis* to identify pathways related to metabolism and trachea development that could help explain energy requirements by the spider's extensive wrapping behavior. The manuscript also explores genetic footprint of venom components still present in the venom less spider. The manuscript has a lot of exciting new data, I find the metabolic and trachea genetics very interesting to specialist as well as the general public. I do however have some issues with the venom sections that in my view need to be addressed.

>>>**Response:** We sincerely appreciate all your valuable comments and suggestions, which helped us in improving the quality of the manuscript. Below is our point-by-point response to each comment.

1. It is worth noting that Uloboridae is not the only spider family to not have venom glands. Holarchaea is another family that has also been described as not having venom glands. This should be mentioned/addressed.

>>>**Response:** Thank you for your reminder. We have added relevant descriptions in the introduction section (**see line 40–44**). The text is taken as follows:

"However, as an important means of hunting and defense, the toxin system gives spiders an outstanding advantage in environmental suitability and has allowed them to spread throughout the world. Of course, there are always exceptions, some outliers are believed to be lacking venom glands. Currently, known spiders without venom grands include the *Holarchaea* genus (2 species)[2–4] and the entire family Uloboridae, with the latter being the most prosperous group of them [5]."

2. I have some issues with Section 7 of the manuscript: "Differential retention of major toxin genes in O. sinensis". Knowing that the family does not have venom glands and has not many millions of years it is not acceptable for the authors to refer to the genes they find in O. sinensis with homology to known venom components as toxin genes. This will lead to many misinterpretations although admittedly it sounds a lot catchier. Moreover, the families that were identified while all are known to be venom components, not all are toxins.

For example, the Translationally Controlled Tumor Protein (TCTP) a histamine-releasing factor acts together with Together the cysteine-rich secretory protein (CRISP) family, contributing to inflammatory responses (Sade et al., 2012; Boia-ferreira et al., 2019; Justa et al., 2020). Given this, the authors will need to tone down the claim of having found toxin in a spider that does not have venom glands. This is not surprising assuming a secondarily loss in the family (the most parsimonious interpretation). I believe this also leads to some misinterpretations of the results; it is my understanding that most venom components are hypothesized to have evolved from ancestor proteins that had normal physiological functions that were then recruited. This information is not concluded from the current manuscript, there is a large body of evidence that supports the evolution of venom proteins via processes such as gene duplication followed by subfunctionalization and/or neofunctionalization, horizontal gene transfer, single gene co-option, etc. It follows that expression of venom homologs in the brain region should not come as a surprise since a lot of what are refer to as toxins are thought to be recruited from the neuropeptides, molecules such as latrodectins (McCowan & Garb 2014). I suggest a major modification of the results/discussion section having more emphasis on the metabolic data and how this can potentially be a trade-off because as written, the manuscript does not describes a trade-off between wrapping and lack of venom.

>>>**Response:** Thank you for your comments, and we fully accept your suggestion. The toxin gene homologs we obtained through our search were all based on the results of blast analysis using the spider toxin database annotated by previous researchers.

In the revised manuscript, we also established the hidden Markov models (HMM) for different types of toxin proteins based on the database, and further confirmed the results obtained from blastp using HMMER v 3.3. The confirmation of any toxin protein requires strict toxicology or virulence testing. In the revised manuscript, we refer to the target gene obtained through our search as “toxin gene homolog”. Based on our analysis results, we ultimately believe that the toxin genes used for hunting in Uloboridae have been lost, and changed the **Result** section 7 to “**Deficiency of toxin genes in O. sinensis**”. We have also deleted some of the less rigorous viewpoints in the **Discussion**. Thank you again for your prompt.

3. The reason why *P. tepidariorium* was used as a reference comparison needs to be stated somewhere. I think it is somewhat arbitrary and I personally would like to hear the authors' reasons. Also why *U. diversus*, another Uloboridae was not included in the study is beyond me. This species also has a chromosome level genome and could add to the story.

>>>**Response:** *P. tepidariorium* is a globally distributed species and also a type species of spider (10.1186/s12915-017-0399-x; 10.1186/s13227-015-0011-9; 10.1093/molbev/msad239; 10.1016/j.dib.2018.05.106). It has the best protein annotation currently available in spiders and a good genome assembly at the chromosome level. In addition, it has rich online data and annotation resources in major databases such as KEGG and GProfiler. Therefore, using it as a control species can obtain a more comprehensive evolutionary landscape. Moreover, *P. tepidariorium* have relatively small differences in body size, ecological niches, and hunting targets compared to *O. sinensis*. Therefore, considering these aspects, the two species also have comparative value. We have added relevant descriptions in the revision, and the screenshot is as follows:

Indeed, *U. diversus* is a very important species for the scientific issues involved in this study. In this revision, we have incorporated this important genome into our analysis system (**see Fig. 2B, 3A**) and supplemented the annotation, increasing the number of coding genes with transcriptome support from 15750 to 19036. Subsequently, we also added some genomes proposed by reviewers to the new analysis and conducted all downstream analyses based on these data.

4. L45-47: I am not sure I understand the meaning of these sentences and I suggest their removal.

>>>**Response:** We have deleted these sentences according your suggestion.

5. L51-54: There are many spider families that have well-developed trachea with branches extended into their prosoma, such as salticidae and Cybaeidae. To make the case more compelling for why it is particularly important for Uloboridae to have this adaptation I suggest the authors include more detrain about the greater need for energy in the limbs or greater metabolic input and thus there could be a greater presence of mitochondria in the

legs or upregulation of genes related to energy metabolism. If this is not expanded, it is unclear why *O. sinensis* is indeed a good model for this study.

>>>**Response:** Thank you for your suggestions. Other groups may also require leg strength, but many have abandoned webs in predatory activities. Uloboridae is all web-building spiders, so we pay more attention to the compensation for the loss of venom glands in web-building spiders, and did not pay special attention to other hunting type groups. Comparative transcriptome analysis of motor organs is indeed crucial, and we have included this work in our revised manuscript. Excerpt as follows:

"In order to further explore the evolution in motor function of Uloboridae family, we compared the transcriptome data of the legs between *O. sinensis* and other species. We used the model species: *P. tepidariorum*, which is also a web-building spider, as a control. Under consistent standardized conditions, the results showed that a large number of genes were differentially expressed (Figure 3B). Compared to *P. tepidariorum*, genes higher expression in the *O. sinensis* legs were most enriched in the mitochondrial matrix, and, other GO terms related to mitochondria also detected high enrichment (Figure 4C). This result suggests that at least in the legs, *O. sinensis* require greater energy consumption compared to typical web-building spiders."

6. L55: Do not start a sentence with a species name.

>>>**Response:** Thank you for your reminder. We have made the corresponding modifications (**see line 58**).

7. L76-80: Uloborid spiders have been described as lacking both venom glands and openings on the fangs for the delivery of venom (Opell 1979; Weng et al. 2006). It is not surprise that you did not observe fang openings in *O. sinensis* fangs. Therefore, I suggest the authors change the wording from 'prove' to further providing evidence that uloborids are not equipped to deliver venom.

>>>**Response:** Thank you for your suggestions, we have made the corresponding modifications (**see line 83–86**).

8. L 94: Not sure "as a percentage of … 98.11%" is needed.

>>>**Response:** Thank you for your comments, we had anticipated that someone would pay attention to this type of information, now we have moved it to the Additional file (**see Results: 2. Observation of behavior and fangs**).

9. L100: change to 24,579 and 24,563

>>>**Response:** Thank you for your suggestions, we have made the corresponding modifications (**see line 104–105**).

10. L101: "in at least one the following databases: "

>>>**Response:** Thank you for your suggestions, we have made the corresponding modifications (**see line 105**).

11. L250-253: I agree that it could be possible that these genes were removed from the genome but it is hard to tell from the current data if this was quick or not after selective pressures changed.

>>>**Response:** Thank you for your reminder. We have reorganized this section (Section 7) based on the new analysis results. The expression you mentioned here has been rewritten and taken as follows:

"Compared with other genes which it is difficult to find pseudogenes, this gene has complete gene structures and CDS regions, transcriptome data also show that it can be normally transcribed into mRNA (Additional file 6). We speculate that this gene (g31478) may plays a role as a non-toxic gene in *O. sinensis*."

12. L297-298: While I do not agree with the statement, at the very least the authors need to provide a lot more information to back up this claim.

>>>**Response:** Thank you for your reminder. This section of the discussion is indeed too radical and lacks research support. We have reorganized the language and removed the expressions about "preadaptation" and "extraction".

13. L139-140: Sentence is confusing as written and should be rewritten or deleted.

>>>**Response:** Thank you for your comments. In the revision, the analysis results under the new background differ from the original manuscript, and this section has been rewritten.

We deliberately avoided such issues during the revision process.

14. L324: Where the samples use males or females? Mature or immature? This will be important for expression levels. Why were the spiders starved for a week?

>>>**Response:** Thank you for your reminder. We have added detailed descriptions of this section in the revision. Excerpt as follows:

“To minimize contamination of intestinal DNA as much as possible, all samples were starvation reared for more than one week at room temperature. Genome DNA for both short and long read sequencing were isolated from the **cephalothoraxes** of **adult female** spiders using the Qiagen Blood & Cell Culture DNA Kit (QIAGEN, Hilden, Germany).”

15. L338: what type of tissue was used to generate the genome? Whole spiders?

>>>**Response:** Thank you for your reminder. We have added detailed descriptions of this section in the revision (**see Methods: 1. Sample collection and DNA extraction**).

16. L349: experiments "were" conducted.

>>>**Response:** Thank you for your reminder. We checked for similar errors to the best of our ability.

17. L372: BUSCO should be spelled out, explained and cited the first time is used.

>>>**Response:** Thank you for your reminder. The Benchmarking Universal Single-Copy Orthologs (BUSCO) method first appeared in the **Results: 2. Genome assembly and annotation**, and in the revision, we spelled out its full name and cited it (see line 95, 96).

18. L375: what do you mead by spinneret? If you dissected the silk glands out (all the silk glands out) then you not have any tissue other than connective and/or fat attached the spinneret. This should be clarified. If the authors took out the larger glands and left the smaller ones behind then the names should reflect that. Are there any images of the tissues used? I will be curious to see what part of the brain and the gut was used for example. Whether the spiders where male or female, at what stage and how many where used should be included for both species.

>>>**Response:** Thank you for your comments. Our approach to dissecting spider spinnerets is similar to that of other appendages. It is to use tweezers to separate it from the body and remove visible internal organs as much as possible. However, the spinner was too small to be completely cleaned and may have a small portion of the silk gland.

We have not saved official images. We shoot a few videos and photos to demonstrate the anatomical process, mainly highlighting the anatomy of the intestine and brain. This information will be uploaded to the gigaDB.

19. L396: Why was the Uloboridae *Uloborus diversus* not included in this study? To my knowledge it is also a chromosome level assembly and would have made a stranger case for the conclusions.

>>>**Response:** Thank you for your comments. We have addressed this section in our response to your previous comments. Here we would like to point out that in addition to the *Uloborus diversus*, we also added two species from RTA clade (*Pardosa pseudoannulata* and *Dolomedes plantarius*), replaced the *Dysdera silvatica* with a better quality genome of *Ectatosticta davidi*, and removed the less important *Hylyphantes graminicola* and *Stegodyphus mimosarum*. In the end, all of these genomes have a protein Busco score of over 90%, and a more suitable evolutionary tree was obtained (**see Fig. 2B**).

20. The manuscript contains both Fig and Figure. For consistency only use one.

>>>**Response:** Thank you for your reminder. We checked for similar errors to the best of our ability.

21. Figure 1 suggestions: B- Adult male or female? Move the scale bar to bottom right. C- I find this section to be very confusing and should perhaps be its own figure. The numbering inside the circles make it hard to understand to me. I suggest a different organization for this figure.

>>>**Response:** Thank you for your reminder. We reorganized the Figure 1 and placed the scale bar in channel B in the bottom right corner of that channel; We have added a separate channel. Used to describe the shooting angle in SEM photographs (**see Fig. 1**).
