# Supplementary material for: A trade-off in evolution: the adaptive landscape of spiders without venom glands
Source: Gigascience. 2024 Aug 5;13:giae048. doi: 10.1093/gigascience/giae048 (PMC11299198; doi:10.1093/gigascience/giae048)
Supplement: giae048_GIGA-D-23-00275_Revision_2 [file giae048_giga-d-23-00275_revision_2.pdf]

GigaScience

A Trade-off in Evolution: The Adaptive Landscape of Spiders without Venom Glands

--Manuscript Draft--

**Manuscript Number:**	GIGA-D-23-00275R2
**Full Title:**	A Trade-off in Evolution: The Adaptive Landscape of Spiders without Venom Glands
**Article Type:**	Research
**Funding Information:**	Strategic Priority Research Program of Chinese Academy of Sciences (XDB31000000) — professor Shuqiang Li<br>the Program of National Natural Sciences Foundation of China (NSFC–32170447) — Dr Zhe Zhao<br>the Program of National Natural Sciences Foundation of China (NSFC–32370490) — Dr Zhe Zhao
**Abstract:**	Background: Venom glands play a key role in the predation and defense strategies of almost all spider groups. However, the spider family Uloboridae lacks venom glands and has evolved an adaptive strategy: They excessively wrap their prey directly with spider silk instead of paralyzing it first with toxins. This shift of survival strategy is very fascinating, but the genetic underpinnings behind it are poorly understood.<br>Results: Spanning multiple spider groups, we conducted multi-omics analyses on Octonoba sinensis, and described the adaptive evolution of the Uloboridae family at the genome level. We observed the coding genes of myosin and twitchin in muscles are under positive selection, energy metabolism functions are enhanced, and gene families related to tracheal development and tissue mechanical strength are expanded or emerged, all of which are related to the unique anatomical structure and predatory behavior of spiders in the family Uloboridae. In addition, we also scanned the elements which are absent or under relaxed purifying selection, as well as toxin gene homologs in the genomes of two species in this family. The results show that the absence of regions and regions under relaxed selection in these spiders' genomes are concentrated in areas related to development and neuro-system. The search for toxin homologs also confirms that there are no toxin coding genes available for hunting in the genome of this group.<br>Conclusions: This study demonstrates the trade-off between different predation strategies in spiders, either using venom or physical energy and provides insights into the possible mechanism underlying this trade-off. Venomless spiders need to mobilize multiple developmental and metabolic pathways related to motor function and limb mechanical strength to cover the decline in adaptability caused by the absence of venom glands.
**Corresponding Author:**	Shuqiang Li, Ph.D.<br>Institute of Zoology Chinese Academy of Sciences<br>Beijing, CHINA
**Corresponding Author Secondary Information:**	
**Corresponding Author's Institution:**	Institute of Zoology Chinese Academy of Sciences
**Corresponding Author's Secondary Institution:**	
**First Author:**	Yiming Zhang
**First Author Secondary Information:**	
**Order of Authors:**	Yiming Zhang<br>Yunxiao Shen<br>Pengyu Jin, Ph.D.<br>Bingyue Zhu, Ph.D.

Powered by Editorial Manager® and ProduXion Manager® from Aries Systems Corporation

	Yejie Lin
	Tongyao Jiang
	Xianting Huang
	Yang Wang
	Zhe Zhao, Ph.D.
	Shuqiang Li, Ph.D.
**Order of Authors Secondary Information:**	

Response to Reviewers:

Response to reviewers
Dear Zhang

Thanks again for the careful handling of our manuscript (GIGA-D-23-00275) submitted to GigaScience. Your profound and constructive feedback, along with the insightful comments from the Dr. Nadia Ayoub and Dr. Sandra Correa-Garhwal, has been invaluable in enhancing the quality of our work.
We have diligently reviewed the comments and suggestions made by the reviewers and have made every effort to incorporate the necessary changes. All modifications made to the manuscript are clearly highlighted, allowing for a comparison with the previous version. We believe that we have addressed all the concerns raised by the reviewers.
Enclosed is a point-by-point response to the reviewers' comments, detailing the changes made and explaining how each suggestion has been incorporated into the revised manuscript. We hope that this comprehensive response, coupled with the revised manuscript, will meet your expectations and further strengthen the scientific impact of our work.
Once again, we appreciate your dedication and commitment to ensuring the highest quality of publications in GigaScience. We look forward to your further guidance and feedback on our revised manuscript.

Thank you.
Best regards,

Yiming Zhang; Shuqiang Li
Institute of Zoology, Chinese Academy of Sciences
1. Beichen West Road, Chaoyang District
Beijing 100101, P. R. China
Tel: +86-13363636950
Fax: +86-10-64807216
Email: zhangyiming@ioz.ac.cn; lisq@ioz.ac.cn

Reviewer #1:
The revised manuscript addresses most of my initial concerns and is much better overall! I'm not totally convinced of a trade-off between venom and web production (or do y'all mean the tradeoff is with excessive physical predation???). Perhaps soften this language? Clarify in discussion. Some additional clarifications are needed prior to publication.
>>>Response: We are once again deeply grateful for your insightful comments and suggestions, which have been instrumental in further enhancing the quality of our manuscript.
The "evolutionary trade-off between the loss of venom glands and the enhancement of physical attack strategies" is exactly what we want to express. To avoid ambiguity in the expression, we have removed the third paragraph in the Discussion, which is "Although the web-building behavior…. this seems to be a trade-off".
In the revised Introduction, we presented the chemical attack properties of venom and emphasized the physical attack properties of "prey-wrapping". We also discussed the genomic changes related to muscle, aerobic respiration and metabolism of energy substances, in the Discussion. Please refer to Introduction lines 52–60 and Discussion lines 324–362 of the revised manuscript for specific modifications.
1. The gene expression analyses are never explained - how was expression level determined - e.g. read mapping using what? What program(s) with what parameters

used to compare tissues within species? What constitutes a differentially expressed gene? What methods were used to compare expression patterns between species?
>>>Response: Thanks for your comments. We have added this section in the Methods (see lines 432–439, Methods: 5. RNA extraction, sequencing and expression analysis), the text is taken as follows:
"Clean reads were aligned to the genome using Hisat2 [60], followed by the quantification of all samples with HTSeq [61] to determine the count value. Subsequently, TPMs were derived through automated scripts."
The "automation scripts" mentioned in the above modifications have been uploaded to the FTP server provided by GigaDB.
"In the differential expression analysis across two species, only Reciprocal Best Hits (RBH) genes were extracted for quantification. To facilitate comparison, ortholog (RBH) gene IDs of O. sinensis were replaced by P. tepidariorum gene IDs for comparison and figure illustration. Finally, differential expression analysis was conducted by R package limma [62]."
For the threshold for identifying differentially expressed genes, we have added relevant descriptions in the caption of Figure 3 (lines 176–177), as recorded below:
"(only Reciprocal Best Hits genes were considered, Fold-change > 1.5, $p < 0.05$)"
In addition, we also used stricter screening conditions (Fold-change > 4, $p < 0.05$) to search for differentially expressed genes, and we created Figure S2 in Additional file 5 using the relevant results, which is similar to Figure 3C.
2. In Figure 3B, is expression analysis for both P. tepidariorum and O. sinensis? Is it comparison of the two? For Figure 3C, is the GO enrichment for genes significantly overexpressed in leg tissues of O. sinensis relative to some other O. sinensis tissue, or relative to P. tepidariorum legs?
>>>Response: Thanks for your comments. Yes, the differential expression analysis in Figure 3B is a comparison between the legs of two species. The revised description in the manuscript is copied as follows:
We have added relevant descriptions in the Figure 3 legends (lines 175–179)
"(B) Differential expression analysis of homologous genes in the legs of Parasteatoda tepidariorum and Octonoba sinensis (only Reciprocal Best Hits genes were considered, Fold-change > 1.5, $p < 0.05$). Genes exhibiting higher expression levels in O. sinensis legs are designated with red upper triangles, whereas those with higher expression in P. tepidariorum legs are designated with green lower triangles."
For Figure 3C, relevant descriptions were added:
Results, 4. Genes under positive selection and energy metabolism in muscle (lines 160–162)
"Compared to the legs of P. tepidariorum, genes with higher expression in the legs of O. sinensis were most enriched in the mitochondrial matrix, meanwhile significant enrichment was also observed in other GO terms pertaining to mitochondria (Figure 3C, Figure S2)."
we have also added relevant content to the caption, the text is taken as follows (lines 179–181):
"(C) GO terms enrichment analysis of genes expressed at higher levels (Fold-change > 1.5, $p < 0.05$) in O. sinensis legs relative to those in P. tepidariorum legs. GO terms related to the mitochondrion are highlighted in red."
3. Is it possible to access all these new annotations? Would be great to point to their public location.
>>>Response: Thanks for your suggestion, the relevant data has been uploaded to the FTP server provided by GigaDB and will be made public after the article is published. I will further confirm with the administrator of the GigaDB that you can obtain this data at this stage of the process.
We have added a public location for sequencing data in SRR, which is ScienceDB (doi.org/10.57760/sciencedb.09166)
4. Why were reciprocal best blast groups used for scans of positive selection rather than the Orthofinder families?
>>>Response: Thanks for your comments. Both the RBH and Orthofinder were used to search for one-to-one orthologs across species, RBH method can obtain more orthologous genes (see Table S19). Therefore, we conducted a positive selection analysis using the reciprocal best blast groups.
5. Table S4 - what is the source of P.tepidariorum? D. spinosa?
>>>Response: Thanks for your comments. We have supplemented this information, as recorded below:
"

Powered by Editorial Manager® and ProduXion Manager® from Aries Systems Corporation

	SpeciesAssembly version (source)SourceAssembly level<br>Parasteatoda<br>tepidariorum10.11922/sciencedb.o00019.00014SciencedbChromosome<br>Deinopis sp.This studyTranscriptome assembly<br>”<br>6. Table S5 - there are no entries under O. sinensis. Should be, right?!<br>>>>Response: Thanks for your reminder. We have added this section.<br>7. Table S6 - is the lfc between P.tep and O.sin? How was this calculated? (see above). Is the p-value shown or the FDR? How were the numbers in Table S6 generated?<br>>>>Response: Thanks for your comments. Yes, this is a differential expression analysis between two species, where lfc represents log2 fold change, and the p-value was displayed in the table.<br>The calculation method was described in detail in the Methods section (lines 432–439). We used P. tepidariorum as a control, and homologous genes were also characterized by gene IDs in P. tepidariorum. A positive value of log2(Fold Change) indicates that the expression level of this gene in O. sinensis is higher than that in P. tepidariorum, while a negative value indicates that the expression level of this gene in O. sinensis is lower than that in P. tepidariorum.<br>8. Table S7 - gene families or genes?<br>>>>Response: Thank you for your reminder. We have reorganized the table according to the attribution of gene families.<br>9. Table S8 - expanded gene families?<br>>>>Response: Thank you, we have reorganized the table according to the attribution of gene families.<br>10. S16 - I don't know what this table provides.<br>>>>Response: Thanks for your comments. This table presents the alterations in the quantity of structural domains observed in a pair of homologous genes, as detailed in the manuscript (lines 298–300). To enhance clarity and facilitate comprehension, we have included a descriptive header for each column.<br>11. Table S8 - Asianopis shows up but was never mentioned in text.<br>>>>Response: Thank you for your reminder. This is an oversight, we found it in Table 18, it should be Deinopis sp., we have corrected it.<br>12. Additional file 6 - Expression matrix - are these TPM values or something else? Is the * in this table the same as the Toxin gene expression matrix?<br>>>>Response: Thanks for your comments. The previous table was a TPM value expression matrix of genes in different O. sinensis tissues, and there was an error in the title, which should be “Figure 7”. Its function is only to demonstrate that two genes in the O. sinensis in Figure 7B(h, linked with red ribbon) do indeed have expression levels. We have removed other genes in the new modification, leaving only the two required genes. We have renamed the table as Table S17 and placed it in Additional file 4.<br>13.Some typos:<br>>>>Response: Thanks for your comments. We have made modifications according to the comments.<br>a.Line 42: delete However,<br>>>> Response: See line 42. We have delete “However”<br>b.Line 59: sinensis needs an s on the end<br>>>> Response: See line 64. “The spider Octonoba sinensis belongs to the family Uloboridae.”<br>c.Line 87: change providing to provides<br>>>> Response: See line 93. “which further provides evidence that uloborids are not equipped to deliver venom (Figure 1C, D).”<br>d.Line 242: lower case we<br>>>>Response: See line 253. “In addition, we referred to the venom gland specific expression module”<br>e.Line 267-268: incomplete sentence<br>>>>Response: Thanks for your comments. We have reorganized the language of this entire piece of content, with the following excerpts (lines 278–280):<br>“Given that O. sinensis had three latrotoxin homologs out of 12 venom components (highest category, Table S14) and latrotoxins are not known outside of Theridiidae, a phylogenetic analysis of this gene family has been conducted.”<br>f.Line 316: Should there be a "lost" between "some of these" and "genes may be"?<br>>>>Response: Thanks for your comments (f–g). Based on the feedback of you and

	another reviewer, this section has been removed. Please refer to the first reply provided to you for details (Discussion lines 324–357).<br>g.Line 320: are the RTA clade really the youngest group of spiders?<br>>>>Response: Thanks for your comments (f–g). Based on the feedback of you and another reviewer, this section has been removed. Please refer to the first reply provided to you for details (Discussion lines 324–357).<br><br>Reviewer #2:<br>Zhang et al provide a multi-omics analyses on Octonoba sinensis (Uloboridae) and describe the genetics that could explain an adaptive response to the lack of venom in the group. The revised version of the manuscript is much improved with new data and new conclusions. However, I still have some concerns that need to be addressed before its publication.<br>>>>Response: We are once again deeply grateful for your insightful comments and suggestions, which have been instrumental in further enhancing the quality of our manuscript. Below, you will find our comprehensive response to each of your comments, addressing them point by point.<br>1. While the addition of Deinopis transcriptome to help with the gene family analyses is fine, this information needs to be included. For example, there is no mention in Figure 1 what the red dotted line means that leads to Deinopis and why it does not have a circle diagram. Table S4 does not have the NCBI info and Table S18 is labeled as Asianopis and not Deinopis. Given that the authors have added other genomes from RTA spiders I am not sure how much this transcriptome is added since it was not included in other analyses in the study. At this point, if it is not adding relevant information to the story, I would recommend removal. It almost looks like a last minute add on.<br>>>>Response: Thank you for your comments. Only one transcriptome is added. The transcriptome assembly of Deinopis sp. has served as an important resource of the manuscript, including the establishment of phylogenetic tree in Figure 2B, the formation of reciprocal best hit (RBH) gene sets, the analysis of selection pressure, and the quest for newly emerging as well as missing genes within the Uloboridae family. However, due to limitations in determining the copy number of homologous genes in the transcriptome assembly and the absence of contiguous genomic fragments, the transcriptome assembly of this spider was excluded from the investigation of gene family expansion and contraction, as well as the analysis of genome Highly-Conserved Elements (HCEs).<br>The RNA-seq data for transcriptome assembly is described in the Methods: 6. Genome annotation (lines 457–461)<br>To enhance clarity and readability, I have made several adjustments in the revised manuscript.<br>Lines 144–146: (The “red dashed line” in the revised manuscript has been changed to “red branch”)<br>“Because Deinopis sp. lacks comprehensive genomic data and is limited to transcriptome information, this species was not included in the synteny analysis (red branch, Figure 2B).”<br>Lines 469–470:<br>“The latter did not include Deinopis sp., as the only transcriptome data cannot determine the number of gene copies.”<br>Furthermore, unless explicitly stated that Deinopis sp. data was not utilized, we have incorporated the transcriptome assembly results of Deinopis sp. into various other analytical frameworks.<br>Regarding the entry pertaining to the Asianopis in Table S18 (Table S19 in the Revised version), it is indeed an error. The intended Asianopis should be Deinopis sp., and we have rectified this mistake in the revised version of the manuscript.<br>We have supplemented this information (Additional file 4: Table S4). The text is taken as follows:<br>“<br>SpeciesAssembly version (source)SourceAssembly level<br>…<br>Parasteatoda tepidariorum10.11922/sciencedb.o00019.00014SciencedbChromosome<br>…<br>Deinopis sp.This studyTranscriptome assembly<br>”

2. Figure 2C seems to be out of order to me. While reading it was hard to understand why it was there (other than being a comparative genome analysis) and not in Figure 5.
>>>Response: Thank you for your comments, this section (C) of Figure 2 has been moved to the Additional file 5: Figure S3.
3. Lines 154-155: expression analyses are not included in the material and methods. This is very important because (1) the reader would need to understand how the expression data was generated: reads mapped to genome? To transcriptome? Using what program? (2) what program and type of normalization was done for the DE analyses? The way these two sentences are written and figure 3 legend, they imply that the authors did DE using P. tepidariorum legs as the control for the legs of O. sinensis. I have never seen a DE across species that way to be able to create a volcano plot as shown on figure 3B. How it was done needs to be included.
>>>Response: Thank you for your comments, we have added this section in the Methods (see lines 432–438, Methods: 5. RNA extraction, sequencing and expression analysis), the text is taken as follows:
“Clean reads were aligned to the genome using Hisat2 [60], followed by the quantification of all samples with HTSeq [61] to determine the count value. Subsequently, TPMs were derived through automated scripts.”
The “automation scripts” mentioned in the above modifications have been uploaded to the FTP server provided by GigaDB.
“In the differential expression analysis across two species, only Reciprocal Best Hits (RBH) genes were extracted for quantification. To facilitate comparison, ortholog (RBH) gene IDs of O. sinensis were replaced by P. tepidariorum gene IDs for comparison and figure illustration. Finally, differential expression analysis was conducted by R package limma [62].”
The new RNA-seq data of P. tepidariorum are deposited into ScienceDB (doi.org/10.57760/sciencedb.09166)
For the threshold for identifying differentially expressed genes, we have added relevant descriptions in the caption of Figure 3 (lines 176–177), as recorded below:
“(only Reciprocal Best Hits genes were considered, Fold-change > 1.5, $p < 0.05$)”
In addition, we also used stricter screening conditions (Fold-change > 4, $p < 0.05$) to search for differentially expressed genes, and we created Figure S2 using the relevant results, which is similar to Figure 3C.
4. One major issue I found was the conclusion. From lines 309 to 324 I feel the argument being made is not completely logical and I can't see how it contributes to the manuscript. The discussion should be about the results and conclusions generated in this study and not on whether venom evolved before or after prey-catching webs. Here the authors have a chance to talk about the metabolic related genes that are under positive selection and how it could relate to an increase endurance in O. sinensis. What are the potential functions of the expanded or new emergent gene families? How all of that relates to no having venom glands and using this extensive prey-wrapping technique. All of that is not included in the conclusion as it stands. This section needs to be rewritten.
>>>Response: Thanks for your comments. Based on comments, we have adjusted this section of Discussion and removed discussions about “whether venom evolved before or after prey-catching webs” (The second paragraph in the original Discussion)
In the revised Discussion, we discussed the genomic features related to excessive physical predation and venom glands loss in detail, and talk about how it could relate to an increase endurance in O. sinensis. Please refer to lines 324–355 of the revised manuscript for specific modifications.
5. Line 49: delete "changes"
>>>Response: Thank you for your suggestions, we have made the corresponding modifications (see lines 47–50).
6. Line 51: instead of "these spiders" use uloborids
>>>Response: Thank you for your reminder. We have made the corresponding modifications (see line 51).
“Due to the absence of venom glands, the predation methods of uloborids are also relatively specialized.”
7. Line 52: instead of "this group" use Uloboridae
>>>Response: Thank you for your suggestions, we have adjusted the language of this section to make it more relevant to the title of our article. This section comes from the discussion in the original version. (see lines 52–60).
“Generally speaking, using venom to paralyze prey is an effective chemical attack

strategy. However, many kinds of spiders integrate both chemical attack and physical attack strategies. Certain species within the Araneoidea family utilize entanglement initially to restrain larger prey before a venomous final strike [6]. Although this predatory tactic may alleviate the selective pressure associated with venom usage, entanglement appears rudimentary in comparison to the prey-wrapping behavior exhibited by uloborids. In Uloboridae, this physical attack as the sole means of attack can span from a few minutes to nearly an hour, with the spider silk utilized sometimes exceeding a hundred meters in length [7–9]. Therefore, considerable physical endurance is indispensable for the successful execution of this predatory tactic."

8. L55: add a reference to the first sentence

>>>Response: Thank you for your comments, we have made the corresponding modifications (see lines 60–62).

"Previous anatomical records indicate that spiders in the family Uloboridae have well-developed trachea [10], and many branches of the trachea extend into the prosoma and appendages [11]."

9. Line 75: delete "in its anatomy"

>>>Response: Thank you for your suggestions, we have made the corresponding modifications (see lines 80–81).

10. Line78: Suggest changing the title of the section to something like: Prey-wrapping behavior observations and fand morphology

>>>Response: Thank you for your suggestions, we have made the corresponding modifications (see line 84).

"1. Prey-wrapping behavior observations and fang morphology"

11. Line82: Araneoidea comes out of nowhere. Maybe say "time for other spider species (Arnaeoidea, 9.7+- …"

>>>Response: Thank you for your comments, we have made the corresponding modifications (see lines 88–89).

"This time is much higher than the previously recorded wrapping time of other spider species (Araneoidea, 9.7 ± 3.0 seconds for small prey, 26 ± 42 seconds for big prey) [6]."

12. Line 87: provides instead of providing. And "uloborids"

>>>Response: Thank you for your comments, we have made the corresponding modifications (see lines 92–93).

"which further provides evidence that uloborids are not equipped to deliver venom (Figure 1C, D)."

13. Line 90: add "s" to observation

>>>Response: Thank you for your comments. We have made the corresponding modifications (see line 96).

"Figure 1: Observations of Octonoba sinensis."

14. Line 91: delete channel (D)

>>>Response: Thank you for your suggestions, we have made the corresponding modifications (see line 97).

15. Line 92 indicates instead of indicating. Delete "channel"

>>>Response: Thank you for your reminder, we have made the corresponding modifications (see lines 97–98).

16. Line 99: spell out 9

>>>Response: Thank you for your reminder, we have made the corresponding modifications (see lines 104–105).

"A total of 20 scaffolds were obtained, of which more than 99.9% of the sequences were loaded onto nine scaffolds that reached the chromosome level (Figure 2A),"

17. L126: maybe add that the proportions circles are next to each species

>>>Response: Thank you for your reminder. We have reorganized the language of this entire piece of content, the text is taken as follows (lines 131–132):

"The collinearity between O. sinensis and other spider species is visualized through circle diagrams positioned adjacent to each respective species."

18. Line 156: add "with" after genes and delete "the" after expression

>>>Response: Thank you for your comments. Based on the feedback from you and another reviewer, this section has been rewritten (lines 160–162).

"Compared to the legs of P. tepidariorum, genes with higher expression in the legs of O. sinensis were predominantly enriched in the mitochondrial matrix, while significant enrichment was also observed in other GO terms pertaining to mitochondria (Figure 3C, Figure S2)."

19. Line 171 add "term" after GO

>>>Response: Thank you for your comments, we have made the corresponding

modifications (see line 179).
“(C) GO terms enrichment analysis of genes expressed at higher levels”
20. Line 172: delete (D-E).
>>>Response: Thank you for your reminder, we have made the corresponding modifications (see lines 180–181).
21. Section 5 should include how the authors identified the expanded and new emergent gene families. Just one or two sentences.
>>>Response: Thank you for your reminder. We have made the corresponding modifications (see lines 188–189).
“To compare the genomic differences between Uloboridae and other spiders, we used CAFE v4.2 [24] to analyze the gene family expansions and contractions.”
(lines 205–207)
“Based on the results of orthologous gene identification, we screened for gene families that are exclusively shared in Uloboridae and not found in any other spider species (species in Figure 2B), designating them as new emergent gene families.”
22.Section 5 Lines 181-182: could rephrase these sentences to be clearer. For example: In O. sinensis, we found four of these families to have a high number of annotated genes that corresponded to: …
>>>Response: Thank you for your reminder. We have made the corresponding modifications (see lines 190–191).
“In O. sinensis, we found four of these families to have a high number of annotated genes that corresponded to:”
23.Figure 4 shows with very low quality on my copy.
>>>Response: Thank you for your comments. We have thoroughly reexamined the image, confirming that the uploaded version possesses a size of 738 KB and a resolution of 2008 × 1279 pixels.
24.In general, when a number has more than 3 digits a comma should be added. For example, Line 243 - instead of 1088 write 1,088. There are too many instances to numbered them all. Please check the document.
>>>Response: Thank you for your comments. We have carefully reviewed the entire text and corrected all such issues.
25.Line 208: add Species before phylogeny
>>>Response: Thank you for your comments. We have made the corresponding modifications (see line 219).
“(A) Species phylogeny for calculating the expansion and contraction of gene families.”
26.Line234: spell out 5
>>>Response: Thank you for your comments. We have made the corresponding modifications (see line 245).
“we found that five genes belong to two toxin related gene families”
27. Line 245 delete "gene" after ")"
>>>Response: Thank you for your comments. We have made the corresponding modifications (see lines 255–256).
28. Figure 5 and 6. The expression plots do not indicate the type of values. Are this TPMs? RPKM? Raw reads?
>>>Response: Thank you for your comments. We have supplemented this information. The text is taken as follows:
(Figure 5 lines 262–266)
“The expression patterns of the genes specifically absent in Uloboridae were examined in the model species Parasteatoda tepidariorum. The heatmap is plotted based on the Z-score transformed from Transcripts Per Million (TPM) values. Different colored markers are used to distinguish between distinct gene types, while an asterisk identifies the gene that is notably absent from the venom gland-specific expression module of P. tepidariorum.”
(Figure 6 lines 308–309)
“The heatmap is plotted based on the Z-score transformed from Transcripts Per Million (TPM) values.”
29.Figure 5A legend. The GO enrichment is for which species? Ptep? O sin?
>>>Response: Thank you for your comments. O. sinensis. We have supplemented this information. The text is taken as follows:
(lines 260–261)
“(A) GO enrichment of genes under relaxed selection in Octonoba sinensis.”
30. Line 258 add "in O. sinensis, we .."
>>>Response: Thank you for your comments. We have made the corresponding modifications (see lines 269–271).

Powered by Editorial Manager® and ProduXion Manager® from Aries Systems Corporation

	“To search for toxin genes in O. sinensis, we integrated the results of previous studies and established a comprehensive toxin protein database (Additional file 6) and screened toxin gene homologs with the same threshold in different species.”<br>31.Line 260: delete have<br>>>>Response: Thank you for your comments. We have made the corresponding modifications (see lines 271–272).<br>32.Line 265: add " in the venom glands of P. tepidariorum"<br>>>>Response: Thank you for your comments. We have made the corresponding modifications (see lines 276–277).<br>“Nevertheless, through the characterization of expression patterns for these O. sinensis homologs in the venom glands of P. tepidariorum,”<br>33.Lines 267-270: these sentences are out of order and hard to follow. It is not clear why the authors are talking about latrotoxin or why an additional gene tree was generated for this venom component and not the other ones. I suggest the authors start with something along the lines of "Given that octonoba had four latrotoxin homologs out of 12 venom components (highest category, Table S14) and latrotoxins are not known outside of Theridida<br>>>>Response: Thank you for your comments. We have reorganized the language of this entire piece of content, the text is taken as follows:<br>(line 276–280)<br>“Nevertheless, through the characterization of expression patterns for these O. sinensis homologs in the venom glands of P. tepidariorum, we discovered that the toxin-related homologs of O. sinensis do not exhibit high expression levels in venom glands (Figure 6). Given that O. sinensis had three latrotoxin homologs out of 12 toxin homologs (highest category, Table S14) and latrotoxins are not known outside of Theridiidae, the phylogenetic analysis of this gene family has been deployed.”
**Additional Information:**	
**Question**	**Response**
Are you submitting this manuscript to a special series or article collection?	No
**Experimental design and statistics**<br><br>Full details of the experimental design and statistical methods used should be given in the Methods section, as detailed in our Minimum Standards Reporting Checklist. Information essential to interpreting the data presented should be made available in the figure legends.<br><br>Have you included all the information requested in your manuscript?	Yes
**Resources**<br><br>A description of all resources used, including antibodies, cell lines, animals and software tools, with enough information to allow them to be uniquely identified, should be included in the Methods section. Authors are strongly encouraged to cite Research Resource	Yes

Powered by Editorial Manager® and ProduXion Manager® from Aries Systems Corporation

Identifiers (RRIDs) for antibodies, model organisms and tools, where possible.<br><br>Have you included the information requested as detailed in our Minimum Standards Reporting Checklist?	
**Availability of data and materials**<br><br>All datasets and code on which the conclusions of the paper rely must be either included in your submission or deposited in publicly available repositories (where available and ethically appropriate), referencing such data using a unique identifier in the references and in the “Availability of Data and Materials” section of your manuscript.<br><br>Have you have met the above requirement as detailed in our Minimum Standards Reporting Checklist?	Yes

Manuscript Click here to access/download;Manuscript;giga-review.docx

A Trade-off in Evolution: The Adaptive Landscape of Spiders without Venom Glands

Yiming Zhang†[1,2,3], Yunxiao Shen†[1,3], Pengyu Jin[1], Bingyue Zhu[1,3], Yejie Lin[2], Tongyao Jiang[1,3], Xianting Huang[1,3], Yang Wang[1,3], Zhe Zhao[1] and Shuqiang Li[1]*

[1]Key Laboratory of Zoological Systematics and Evolution, Institute of Zoology, Chinese Academy of Sciences, Beijing 100101, China

[2]Hebei Key Laboratory of Animal Diversity, College of Life Sciences, Langfang Normal University, Langfang 065000, China

[3]University of Chinese Academy of Sciences, Beijing 101408, China

† Yiming Zhang and Yunxiao Shen contributed equally to this work.

*Corresponding author: E-mail: lisq@ioz.ac.cn

Abstract

Background: Venom glands play a key role in the predation and defense strategies of almost all spider groups. However, the spider family Uloboridae lacks venom glands and has evolved an adaptive strategy: They excessively wrap their prey directly with spider silk instead of paralyzing it first with toxins. This shift in survival strategy is very fascinating, but the genetic underpinnings behind it are poorly understood.

Results: Spanning multiple spider groups, we conducted multi-omics analyses on *Octonoba sinensis*, and described the adaptive evolution of the Uloboridae family at the genome level. We observed the coding genes of *myosin* and *twitchin* in muscles are under positive selection, energy metabolism functions are enhanced, and gene families related to tracheal development and tissue mechanical strength are expanded or emerged, all of which are related to the unique anatomical structure and predatory behavior of spiders in the family Uloboridae. In addition, we also scanned the elements that are absent or under relaxed purifying selection, as well as toxin gene homologs in the genomes of two species in this family. The results show that the absence of regions and regions under relaxed selection in these spiders' genomes are concentrated in areas related to development and neuro-system. The search

for toxin homologs also confirms that there are no toxin-coding genes available for hunting in the genome of this group.

Conclusions: This study demonstrates the trade-off between different predation strategies in spiders, either using chemical or physical strategy, and provides insights into the possible mechanism underlying this trade-off. Venomless spiders need to mobilize multiple developmental and metabolic pathways related to motor function and limb mechanical strength to cover the decline in adaptability caused by the absence of venom glands.

Keywords: Venom gland deficient, Adaptive evolution, Genome, *Octonoba sinensis*

Introduction

"Venomous" is a common way that people perceive spiders (Araneae). The toxic, painful, and even fatal bite is always frightening. Indeed, almost all spiders are venomous. In the earliest divergent suborder Mesothelae, they already had fangs that could inject venom [1], and some highly toxic species make spiders even more notorious. As an important means of hunting and defense, the toxin system gives spiders an outstanding advantage in environmental suitability and has allowed them to spread throughout the world. Of course, there are always exceptions, and some outliers are believed to be lacking venom glands. Currently, known spiders without venom grands include *Holarchaea* (2 species)[2–4] and the entire family Uloboridae, with the latter being the most prosperous group [5]. Compared to *Holarchaea*, which has a smaller body size, fewer species, and limited distribution, the family Uloboridae provides a satisfactory model for us to study the evolution of an important synapomorphy of spiders and the adaptation strategy brought about by the loss of important functional traits.

Due to the absence of venom glands, the predation methods of uloborids are also relatively specialized and excessive. Generally speaking, using venom to paralyze prey is an effective chemical attack strategy. However, many kinds of spiders integrate both chemical attack and physical attack strategies. Certain species within the Araneoidea family utilize entanglement initially to restrain larger prey before a

venomous final strike [6]. Although this predatory tactic may alleviate the selective pressure associated with venom usage, entanglement appears rudimentary in comparison to the prey-wrapping behavior exhibited by uloborids. In Uloboridae, this physical attack as the sole means of attack can span from a few minutes to nearly an hour, with the spider silk utilized sometimes exceeding a hundred meters in length [7–9]. Therefore, considerable physical endurance is indispensable for the successful execution of this predatory tactic. Previous anatomical records indicate uloborids have well-developed trachea [10], and many branches of the trachea extend into the prosoma and appendages [11]. however, there is no relevant research that can link these adaptive characteristics to the venom gland deficiency of this group.

The spider *Octonoba sinensis* belongs to the family Uloboridae. Their body size is relatively larger than other members of the family [12], and this species is widely distributed in East Asia, Southeast Asia and North America [2]. Its habitat is close to human buildings, and the populations are large, so they can be easily collected in cities (Figure 1A, B). These aforementioned characteristics make it a model species of the Uloboridae to study the biological characteristics of this family.

In this study, we generated a chromosome-level genome assembly of *O. sinensis*, and high-quality annotation was performed on the genomes of *Pardosa pseudoannulata* and *Dolomedes plantarius*, as well as optimization of annotation for the genomes of *Uloborus diversus* and *Latrodectus elegans*. Furthermore, we assembled a full-protein reference for *Deinopis* sp. using transcriptome data. By leveraging multi-omics datasets from various spider species, we explored the molecular basis of the unique adaptive strategies in the family Uloboridae. In the selection pressure analysis, we found that some important genes related to muscle movement have undergone significant positive selection. In gene family analysis, we observed that with the absence or relaxation of toxins and developmental related genes, gene families related to tracheal development, skeletal development, and tissue force-bearing structures have significantly expanded or emerged in the genomes of this group. Moreover, energy metabolism related genes exhibit high expression, and enzyme activity in related pathways is significantly increased. These all provide a plausible explanation for the improvement in respiratory efficiency and the well-developed tracheal system observed.

Results

1. Prey-wrapping behavior observations and fang morphology

Through our observations, like other species in the family Uloboridae, *O. sinensis* only arrest their prey through extensive silk wrapping. The time usually exceeds 3 minutes (sometimes even 8–9 minutes), during which there may be several brief breaks (Additional file 1–3). This time is much higher than the previously recorded wrapping time of other spider species (Araneoidea, 9.7 ± 3.0 seconds for small prey, 26 ± 42 seconds for big prey) [6].

In addition, for the first time, we examined the fangs of *O. sinensis* from multiple angles with a Scanning Electron Microscope (SEM). Generally speaking, if a species has venom glands, a channel opening for injecting venom should be found on the fangs [1, 13]. We did not observe this in *O. sinensis*, which further provides evidence that uloborids are not equipped to deliver venom (Figure 1C, D).

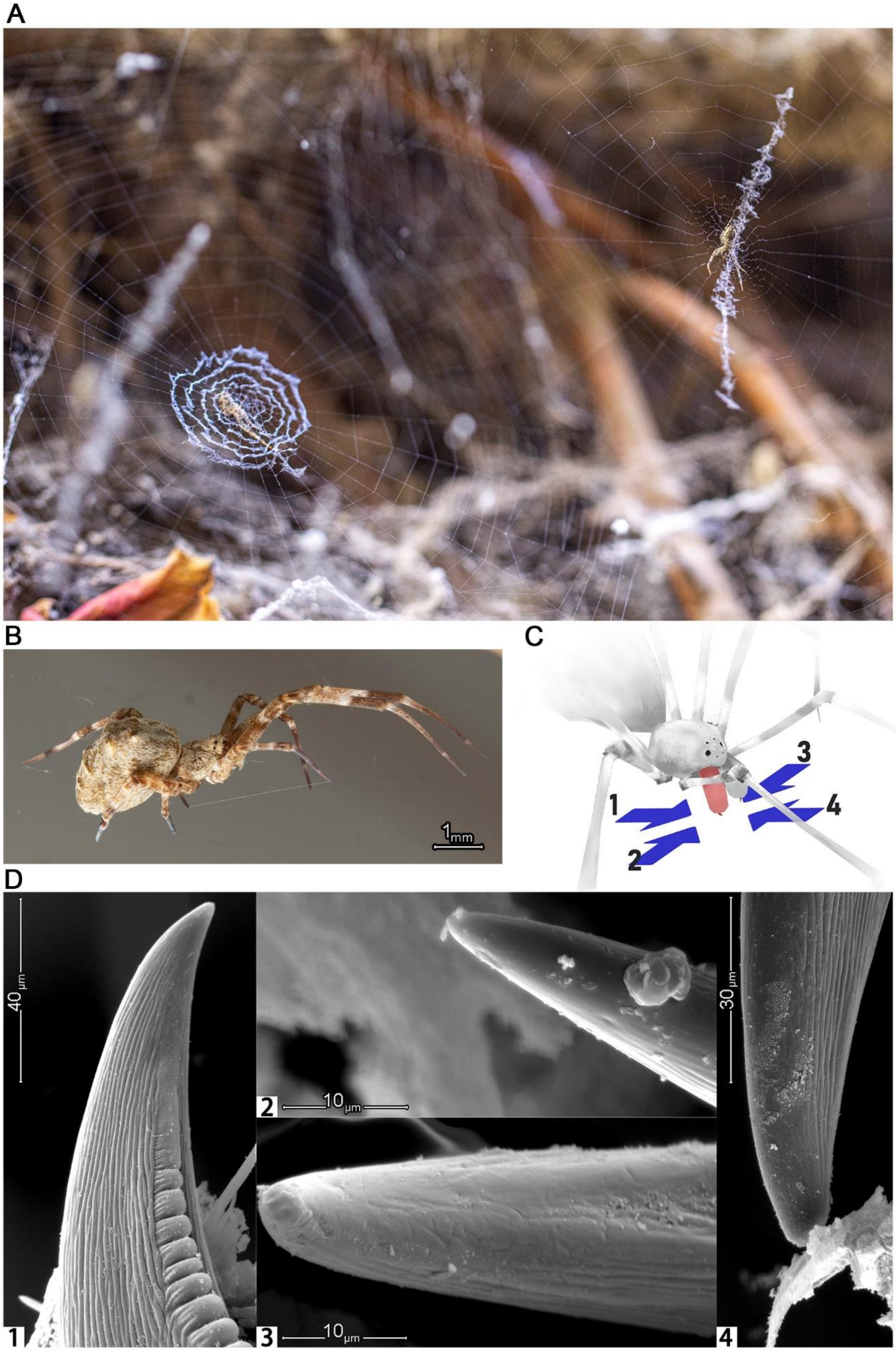
A
B
1mm
C
1
2
3
4
D
40 µm
10 µm
10 µm
30 µm
1
2
3
4

Figure 1: Observations of *Octonoba sinensis*. (A) Two specimens of *O. sinensis* on their orb webs in their natural environment. (B) Adult female of *O. sinensis*. (C) The perspective diagram in D, with arrows numbered 1–4 indicates the shooting angles of the four images in D. (D) Scanning electron microscope image of *O. sinensis* fangs.

2. Genome assembly and annotation

We assembled an *O. sinensis* genome of 1.34 Gb, which is slightly smaller than the prediction of 1.47 Gb based on Illumina data (Additional file 4: Table S1). The average GC content is 32.57%, N50 value is 139.92 Mb. A total of 20 scaffolds were obtained, of which more than 99.9% of the sequences were loaded onto nine scaffolds that reached the chromosome level (Figure 2A), which was consistent with the previous karyotype analysis of *O. sinensis* [14]. The Benchmarking Universal Single-Copy Orthologs (BUSCO) [15, 16] score is 95.3% in arachnida_odb10, (Additional file 4: Table S2).

In the annotation of repetitive sequences of the genome, we found that the proportion of repetitive regions was 55.08%, and the most recognizable element was DNA transposons, which accounted for 18.5% of the genome (Additional file 4: Table S3, Additional file 5: Figure S1). In other spider genomes, most species are dominated by DNA transposons [17–19]. In our assembly, 24,579 coding genes were annotated, and 24,563 genes have obtained effective functional annotation in at least one of the following databases: NCBI-Nr (http://www.ncbi.nlm.nih.gov), Swiss-Prot (http://www.uniprot.org/) or EggNOG v5.0 databases (http://eggnog5.embl.de/) [20]. The chromosome loading rate of coding genes is 99.09%. The BUSCO assessment of protein level is 94.8% (Additional file 4: Table S2).

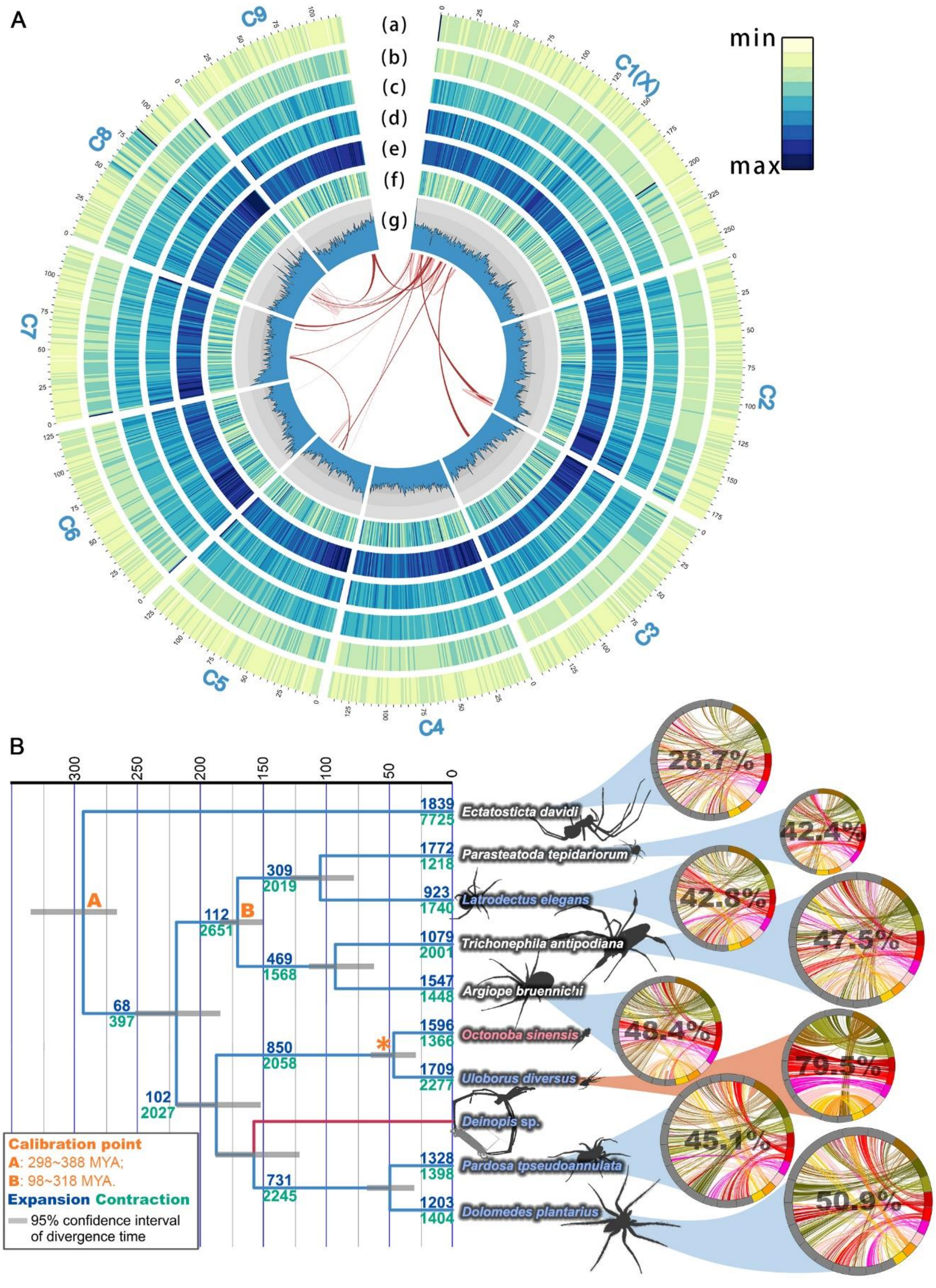

Figure 2: Genome and comparative genomic analysis results from *Octonoba sinensis*. (A) Assembly

and structural annotation of the *O. sinensis* genome. The distribution of different elements is marked with lowercase letters from the outside to the inside in the circle diagram, a: gene; b: SINE, c: LINE, d: LTR; e: DNA transposon; f: Highly-Conserved Elements (HCEs); g: GC content. The inner lines of the circle graph are collineation, dark red lines are collineation between chromosomes, pink lines represent collineation within the chromosome. (B) Phylogeny of multiple spider species. All nodes received 100% bootstrap support. We have provided new annotated genomes marked in light blue font, and new genome assemblies and annotations marked in light red font. The 95% confidence interval of the divergence time is represented by the gray strip on the phylogeny, and the number of expanded and contracted gene families at each node is represented by light blue and light green numbers respectively. The asterisk indicates the foreground branch in the selection pressure analysis. The branch with only transcriptome data cannot deploy CAFE analysis and is highlighted by a red branch. The collinearity between *O. sinensis* and other spider species is visualized through circle diagrams positioned adjacent to each respective species. The proportion of collinearity segments within the *O. sinensis* genome is emphasized in bold percentages.

3. Divergence time estimation and synteny analysis

Genomes of model species — the house spider (*Parasteatoda tepidariorum*), *O. sinensis*, *U. diversus* and seven other representative species (Additional file 4: Table S4) were selected for orthologous gene identification (see methods). A total of 1,560 single-copy gene families shared by all species were identified and used to construct the phylogenetic tree (Figure 2B). All nodes have 100% bootstrap support, and the topological structure and the divergence time of each node prediction are similar to those of previous studies [21–23].

Among the species involved in the above analysis, genomes with chromosome-level assemblies were selected for synteny analysis with *O. sinensis* (Additional file 4: Table S4). There is a trend that the closer the relationship, the stronger the collinearity (Figure 2B). Because *Deinopis* sp. lack comprehensive genomic data, this species was not included in the synteny analysis (red branch, Figure 2B).

4. Genes under positive selection and energy metabolism in muscle

In the selection pressure analysis, at the node of the Uloboridae (asterisk, Figure 2B), 401 genes were under positive selection (Additional file 4: Table S5). Although these genes did not achieve effective GO enrichment (p. adjust < 0.05), we found that there is tissue preference in the expression of some positive selection genes (PSGs), and, in *O. sinensis*, these genes have the highest enrichment in embryos and muscles (Figure 3A). It is worth noting that these PSGs in muscle tissue include *myosin* (gene ID: g27351, p-value = 2.59e-04), an important molecular motor [24, 25], and *twitchin* (gene ID: g8872, p-value = 7.55e-05), a key regulator of muscle movement [26].

To further explore the evolution of the motor function of Uloboridae, we compared the transcriptome data of the legs between *O. sinensis* and other species. We used the model species *P. tepidariorum*, which is also a web-building spider, as a control. Under consistent standardized conditions, the results showed that a large number of genes were differentially expressed (Figure 3B, Additional file 4: Table S6). Compared to the legs of *P. tepidariorum*, genes with higher expression in the legs of *O. sinensis* were most enriched in the mitochondrial matrix, meanwhile significant enrichment was also observed in other GO terms about mitochondria (Figure 3C, Figure S2). This result suggests that at least in the legs, *O. sinensis* requires greater energy consumption compared to typical web-building spiders. On this basis, we examined the activities of several key enzymes in the mitochondria and involved in energy metabolism, including hexokinase (HK), pyruvate dehydrogenase (PDH), α-ketoglutarate dehydrogenase (α-KGDHC), NADH dehydrogenase (ND), and ATP synthase (ATPase). Our results revealed that the activity levels of these five enzymes in the body of *O. sinensis* were higher compared to those in *P. tepidariorum* (Figure 3E). Furthermore, while there was no significant difference in CO_2 production rates between *O. sinensis* and *P. tepidariorum* in a resting state, *O. sinensis* exhibited significantly higher rates under fatigue treatment (Figure 3D). These findings suggest that the evolution related to energy metabolism in the motor organs may be a key factor in the sustained output power of species in the Uloboridae family.

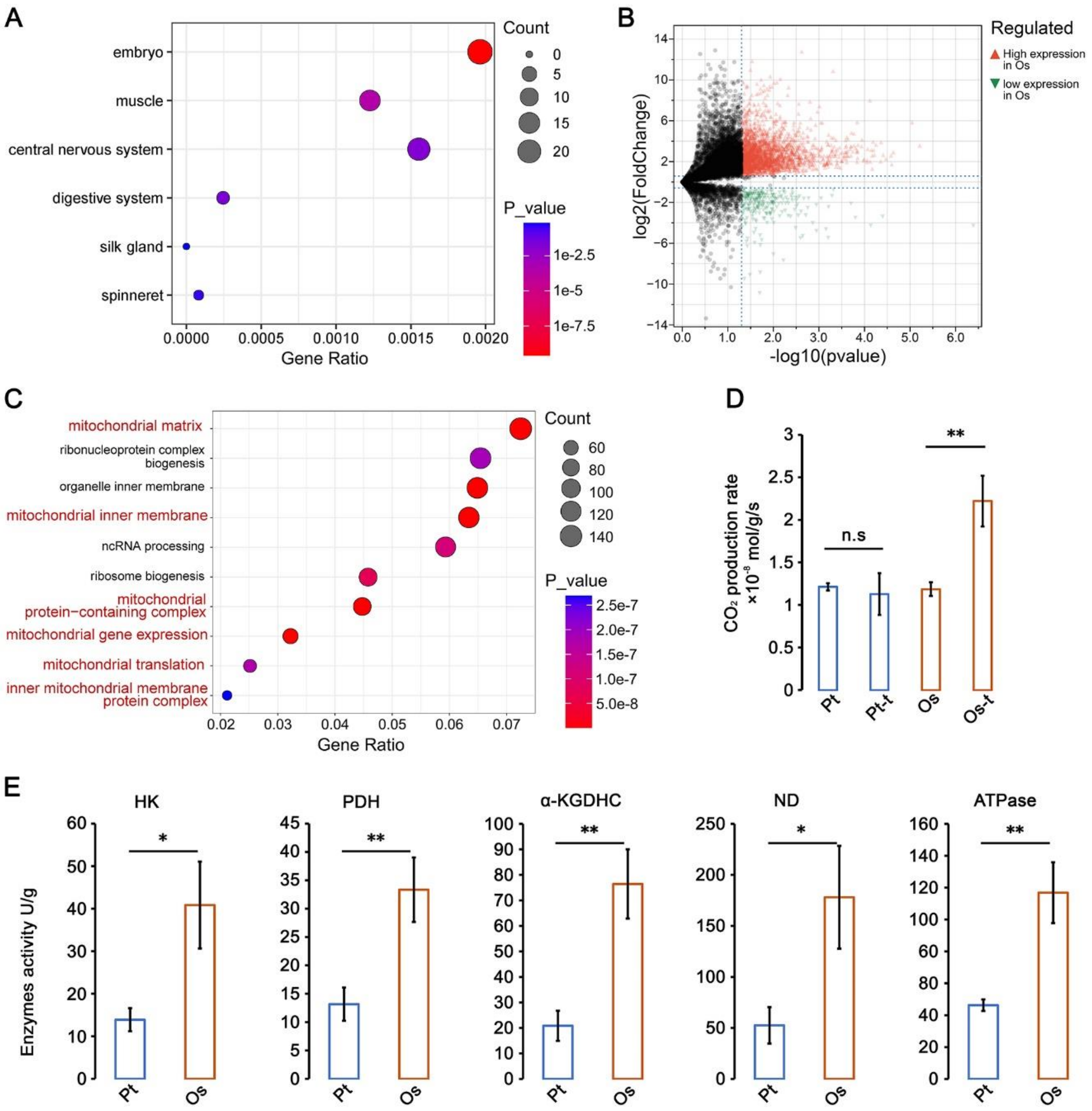

Figure 3: Positive selection and energy metabolism. (A) tissue enrichment of genes under positive selection. (B) Differential expression analysis of homologous genes in the legs of *Parasteatoda tepidariorum* and *Octonoba sinensis* (only Reciprocal Best Hits genes were considered, Fold-change > 1.5, $p < 0.05$). Genes exhibiting higher expression levels in *O. sinensis* legs are designated with red upper triangles, whereas those with higher expression in *P. tepidariorum* legs are designated with green lower triangles. (C) GO terms enrichment analysis. Compared to the legs of *P. tepidariorum*, genes with higher expression (Fold-change > 1.5, $p < 0.05$) in the legs of *O. sinensis* were analyzed. GO terms

related to the mitochondrion are highlighted in red. Metabolic rate measurement (D) and enzyme activity (E) of hexokinase, HK; pyruvate dehydrogenase, PDH; alpha-ketoglutarate dehydrogenase complex, α-KGDHC); NADH dehydrogenase, ND; and ATP synthase, ATPase. Pt: *P. tepidariorum*, Os: *O. sinensis*, -t: under fatigue treatment. Significant differences are denoted by *, $P < 0.05$; **, $P < 0.01$;; and n.s., not significant.

5. Expanded and new emergent gene families

To compare the genomic differences between Uloboridae and other spiders, we used CAFE v4.2 [27] to analyze the gene family expansions and contractions. Results indicate that 123 gene families have undergone significant expansion at the ancestral node of the Uloboridae (Figure 4A). In *O. sinensis*, we found four of these families to have a high number of annotated genes that corresponded to: FH2 domain containing 1 (FHDC1), FBN1, WD40 repeat proteins (WD40), and seven-(pass)-transmembrane domain receptors 1 (7tm_1) (Figure 4B, Additional file 4: Table S7). Interestingly, studies have shown that FHDC1 proteins not only play a crucial role in the development of the tracheal system in fruit flies [28] but also have significant implications in muscle movement [29, 30]. In addition, the preproprotein of FBN1 is proteolytically processed to generate two proteins including the extracellular matrix component fibrillin-1 and the protein hormone asprosin. Fibrillin-1 is an extracellular matrix glycoprotein that serves as a structural component of calcium-binding microfibrils. These microfibrils provide force-bearing structural support in elastic and nonelastic connective tissue throughout the body. Asprosin has been shown to regulate glucose homeostasis [31]. Apart from the four superfamilies mentioned above, we have deployed GO enrichment analysis of the other expanded gene families. REVIGO [32] results show that these annotated genes were mainly enriched in the transport of carbohydrates and organic acids, the immune system, and the functions related to transposable elements (Figure 4C).

Based on the results of orthologous gene identification, we screened for gene families that are exclusively shared in Uloboridae and not found in any other spider species (species in Figure 2B), designating them as new emergent gene families. 269 such gene families have been identified in both

O. sinensis and *U. diversus*, with a total of 658 members in the *O. sinensis* genome (Additional file 4: Table S8). GO enrichment results showed that these genes are more enriched in GO terms that are related to bone trabecular development (Figure 4D).

Generally speaking, spiders lack endurance, necessitating the prompt subduing of their prey within a brief timeframe during hunting [13]. However, uloborids can exercise intensely for nearly an hour [9]. Our results indicate that genes related to tracheal development, skeletal development, tissue force-bearing structures, and energy metabolism have significantly expanded or emerged in the genome of Uloboridae. We believe that the evolution of these aspects is highly likely to be related to their increased endurance.

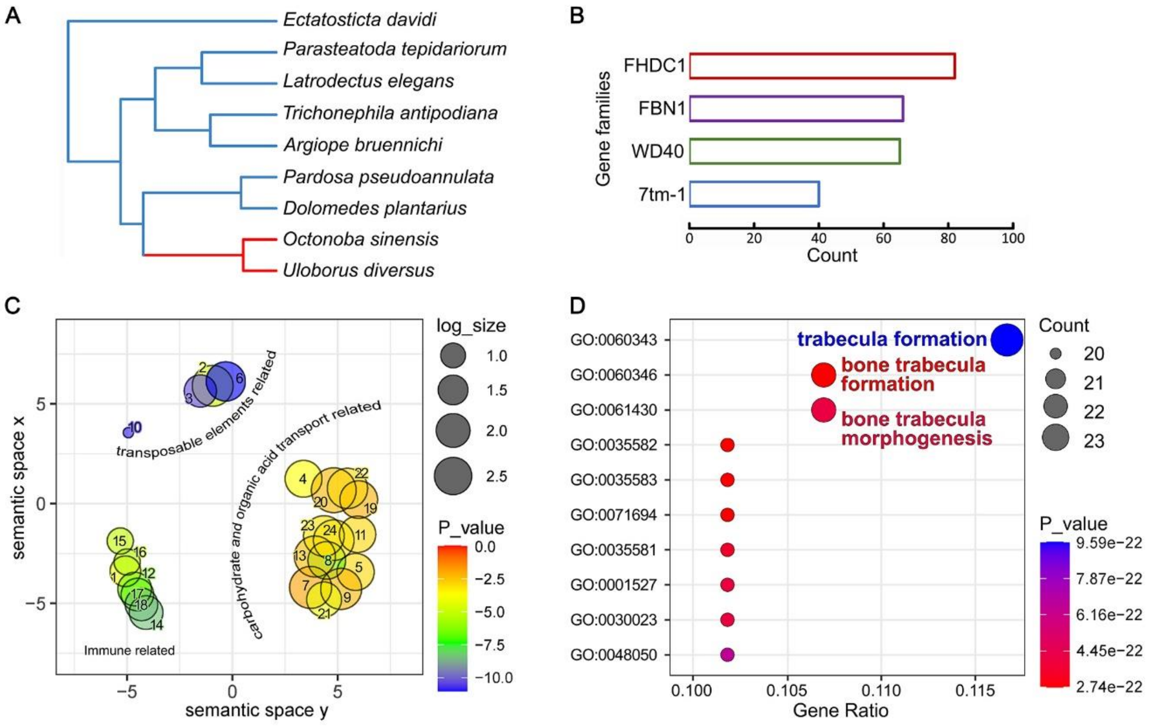

Figure 4. Expanded and new emergent gene families. (A) Species phylogeny for calculating the expansion and contraction of gene families. Uloboridae branches are in red. (B) The four superfamilies with the highest number of clearly annotated genes. (C) REVIGO plot of GO enrichment results for the other expanded gene families, excluding four superfamilies. The numbers in the figure refers to different GO terms: 1-response to molecules of bacterial origin, 2-DNA

recombination, 3-DNA transposition, 4-receptor-mediated endocytosis, 5-carbohydrate transport, 6-DNA integration, 7-organic anion transport, 8-sialic acid transport, 9-organic acid transport, 10-transposition, 11-carbohydrate transmembrane transport, 12-response to type II interferon, 13-carboxylic acid transport, 14-response to interleukin-4, 15-cellular response to biotic stimulus, 16-cellular response to molecule of bacterial origin, 17-cellular response to type II interferon, 18-cellular response to interleukin-4, 19-monoatomic anion transmembrane transport, 20-inorganic cation transmembrane transport, 21-carbohydrate derivative transport, 22-proton transmembrane transport, 23-organic acid transmembrane transport, 24-carboxylic acid transmembrane transport. (D) GO enrichment of new emergent gene families in Uloboridae.

6. Absent regions and genes under relaxed purifying selection

Due to the absence of venom glands, the genes or functional regions specifically involved in the venom gland system in Uloboridae may be subjected to relaxed purifying selection or gradually lost from the genome. To obtain this information, spanning nine spider genomes (Figure 4A), Highly-Conserved Elements (HCEs) were searched and the sites with Uloboridae-specific deletions among them were identified (Additional file 5: Figure S3, Additional file 4: Table S9 S10). In addition, absent genes and genes under selective relaxation in the *O. sinensis* and *U. diversus* genomes were analyzed against the background of species with venom glands (Figure 2B, Additional file 5: Figure S3, Additional file 4: Table S11 S12). We conducted functional enrichment on the above results and found that the biggest difference between Uloboridae and background species comes from the development, especially the neuro-development, related gene family (Figure 5A, B).

In the homologs missing from Uloboridae, we found that five genes belong to two toxin related gene families (LRR and CRISP), as well as three transcription factors. It is worth noting that the expression patterns of the homologs of two LRR genes (LOC122270931, LOC107442855) in *P. tepidariorum* indicate their highest expression in venom glands, although neither of them has been annotated as homologs to known toxin genes (Figure 5C). Three transcription factors belong to important components that activate transcription: “protein c-ets-2”; the “cooled coil and C2 domain containing

protein (CC2D)” that regulates neurotransmitter expression; and the component of the STAGA complex: “ataxin-7”.

In addition, we referred to the venom gland-specific expression module of *P. tepidariorum* obtained in a previous study (n = 1,088, Additional file 4: Table S13) [33] and found that only one gene (LOC107440400), which is specifically lost in Uloboridae, intersects with this module. This gene is a SOBP (Sine Oculis-Binding Protein Homolog), and the protein encoded by this gene is involved in the development of the cochlea, and genetic defects are also related to intellectual disability (Figure 5C).

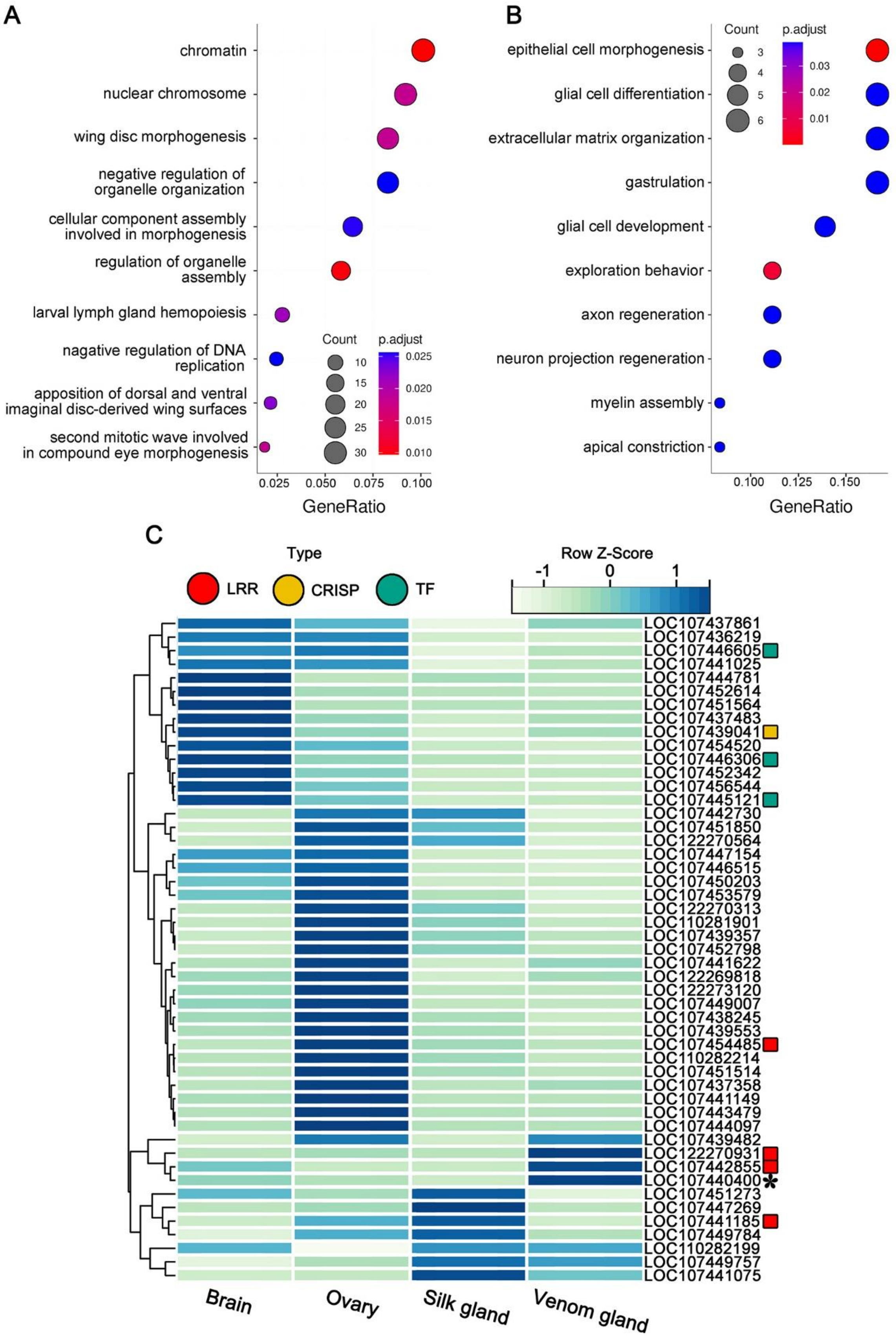

A
chromatin
nuclear chromosome
wing disc morphogenesis
negative regulation of organelle organization
cellular component assembly involved in morphogenesis
regulation of organelle assembly
larval lymph gland hemopoiesis
nagative regulation of DNA replication
apposition of dorsal and ventral imaginal disc-derived wing surfaces
second mitotic wave involved in compound eye morphogenesis
Count
10
15
20
25
30
p.adjust
0.025
0.020
0.015
0.010
0.025 0.050 0.075 0.100
GeneRatio
B
epithelial cell morphogenesis
glial cell differentiation
extracellular matrix organization
gastrulation
glial cell development
exploration behavior
axon regeneration
neuron projection regeneration
myelin assembly
apical constriction
Count
3
4
5
6
p.adjust
0.03
0.02
0.01
0.100 0.125 0.150
GeneRatio
C
Type
LRR
CRISP
TF
Row Z-Score
-1 0 1
LOC107437861
LOC107436219
LOC107446605
LOC107441025
LOC107444781
LOC107452614
LOC107451564
LOC107437483
LOC107439041
LOC107454520
LOC107446306
LOC107452342
LOC107456544
LOC107445121
LOC107442730
LOC107451850
LOC122270564
LOC107447154
LOC107446515
LOC107450203
LOC107453579
LOC122270313
LOC110281901
LOC107439357
LOC107452798
LOC107441622
LOC122269818
LOC122273120
LOC107449007
LOC107438245
LOC107439553
LOC107454485
LOC110282214
LOC107451514
LOC107437358
LOC107441149
LOC107443479
LOC107444097
LOC107439482
LOC122270931
LOC107442855
LOC107440400 *
LOC107451273
LOC107447269
LOC107441185
LOC107449784
LOC110282199
LOC107449757
LOC107441075
Brain
Ovary
Silk gland
Venom gland

Figure 5: Absent regions and regions under relaxed selection. (A) GO enrichment of genes under relaxed selection in *Octonoba sinensis*. (B) GO enrichment of genes related to missing Highly-Conserved Elements (HCEs) in *O. sinensis*. (C) The expression patterns of the genes specifically absent in Uloboridae were examined in the model species *Parasteatoda tepidariorum*. The heatmap is plotted based on the Z-score transformed from Transcripts Per Million (TPM) values. Different colored markers are used to distinguish between distinct gene types, while an asterisk identifies the gene that is notably absent from the venom gland-specific expression module of *P. tepidariorum*.

7. Deficiency of toxin genes in *O. sinensis*

To search for toxin genes in *O. sinensis*, we integrated the results of previous studies and established a comprehensive toxin protein database (Additional file 6) and screened toxin gene homologs with the same threshold in different species. In the *O. sinensis* and *U. diversus* genomes, we identified 12 and 11 homologs, respectively, that have similar structures to members of six major toxin or venom component gene families (Latrotoxin, Latrodectin, CRISP, ICK, TCTP, EF-hand and ctenitoxin). Our findings indicate that although the Uloboridae family has a relatively low number of toxin-related homologs, their count still exceeds that of some venomous spiders, such as *Ectatosticta davidi* [34–37] (Additional file 4: Table S14). Nevertheless, through the characterization of expression patterns for these *O. sinensis* homologs in the venom glands of *P. tepidariorum*, we discovered that the toxin-related homologs of *O. sinensis* do not exhibit high expression levels in venom glands (Figure 6). Given that *O. sinensis* had three latrotoxin homologs out of 12 toxin homologs (highest category, Table S14) and latrotoxins are not known outside of Theridiidae, a phylogenetic analysis of this gene family has been conducted. In this analysis, we found that the three homologs in *O. sinensis* are not clustered on the same branch as the reported Latrotoxin (Additional file 5: Figure S4) [38]. There is a hypothesis regarding the evolution of venom components that ancestors of toxin proteins were originally proteins with normal physiological functions that were recruited in venom glands to play the role of venom components [39], and our results also support this viewpoint.

To further explore the evolutionary processes of *O. sinensis* toxin genes in the absence of venom glands, we conducted a pseudogene search on non-coding regions of the *O. sinensis* genome, but traditional search methods did not identify pseudogenes (blastn, E-value 1e-5, matching length 50 bp) [40]. However, we did find more traces of toxin gene homologs in the blastx search through the protein sequence in the toxin genes database. These results include 48 different genomic regions, but only one of which had an effective hit with the relatively reliable toxin gene of *P. tepidariorum* (LOC107440051) (Additional file 4: Table S15, Figure 6).

By searching for toxin gene homologs in collinearity fragments of *O. sinensis* and *P. tepidariorum*, a particular class of genes was found in *O. sinensis*. These genes are located in the same place as the *P. tepidariorum* toxin gene homologs in the collinearity segment, but they can no longer be identified as toxin genes (below the minimum recognition threshold, see methods) (red ribbon, Figure 7B). This includes a pair of CRISP genes (Figure 7Bh), which is highly expressed in the venom glands of *P. tepidariorum* (LOC107437238, Figure 6). However, in *O. sinensis*, the gene (g31478) cannot be unambiguously classified as a homolog of toxin genes due to its possession of a significantly limited number of functional domains in comparison to LOC107437238 (Additional file 4: Table S16). Compared with other genes in which it is difficult to find pseudogenes, this gene has complete gene structures and CDS regions. Transcriptome data also show that it can be normally transcribed into mRNA (Additional file 4: Table S17). We speculate that this gene (g31478) may play a role as a non-toxic gene in *O. sinensis*.

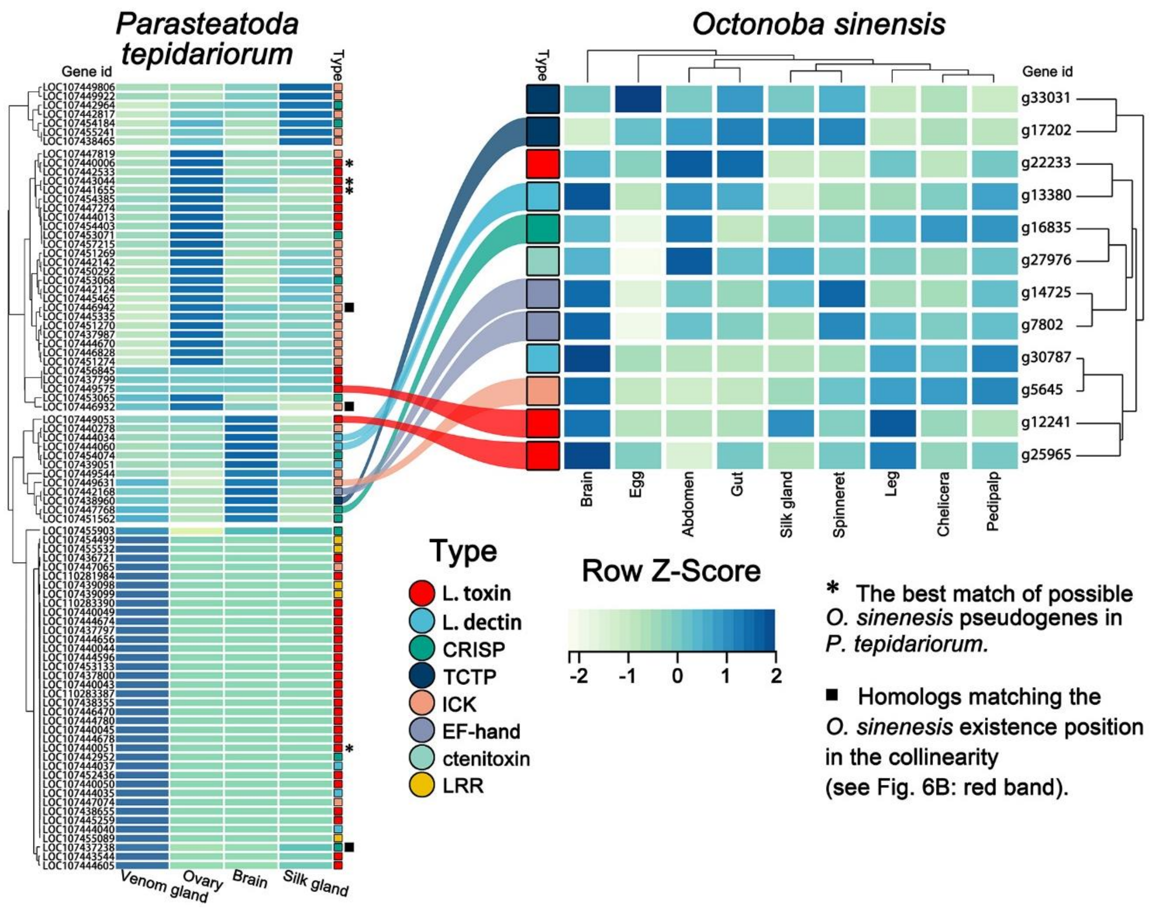

Figure 6. The expression patterns of toxin homolog coding genes in *Octonoba sinensis* and *Parasteatoda tepidariorum*. The heatmap is plotted based on the Z-score transformed from Transcripts Per Million (TPM) values. The ribbon connects the homologs genes. The black squares and asterisks indicate genes found in *P. tepidariorum* that are related to traces of the *O. sinensis* genome.

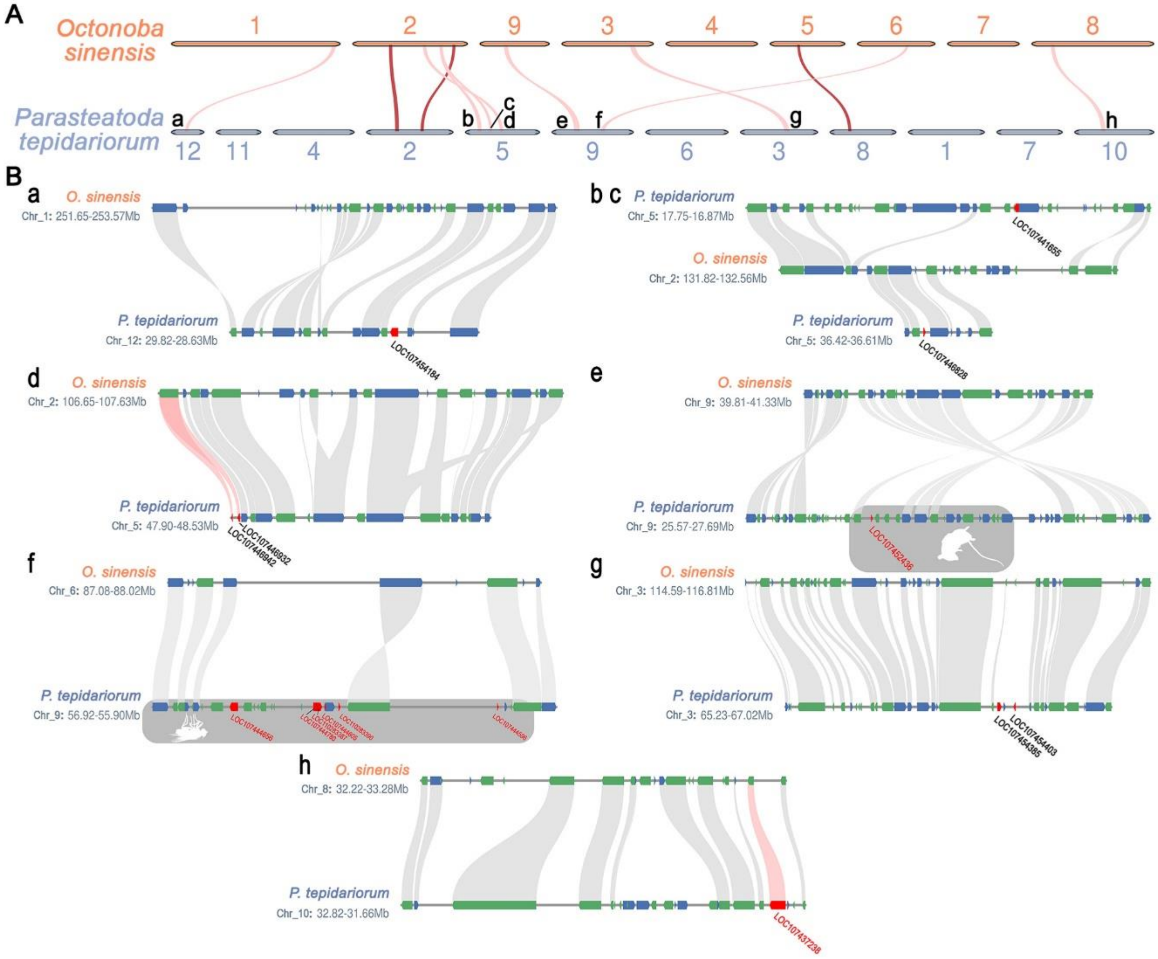

Figure 7. Collinearity containing toxin homologs between *Octonoba sinensis* and *Parasteatoda tepidariorum*. (A) Collinearity of all toxin homologs. If the toxin homologs were lost in *O. sinensis*, the collinearity fragments are represented by a pink ribbon. (B) Gene correspondence in each collinearity fragment where toxin gene deletion occurs in A. Each lowercase letter corresponds to the collinearity fragments represented by the same letter in A; the toxin gene names in *P. tepidariorum* are listed, and the red gene names highlight the toxin genes that are highly expressed in the venom glands. The toxin genes in the gray background belong to the Latrotoxin gene family, and different silhouettes indicate the type of toxin and the target group of toxicity. Mouse = vertebrates (alpha-latrotoxin), Fly = insects (delta-latrotoxin); the red ribbons indicate that the collinearity of the linked genes is extremely weak, below the threshold (see methods).

Discussion

During evolution, the loss of important organs is accompanied by a series of adaptive evolution. such as the enhancement of non-visual senses of some eyeless organisms [41–45]. In Uloboridae, due to the absence of venom glands, prey-wrapping as the sole attack strategy is excessive. Multi-omics analysis revealed the absence of toxin genes in *O. sinensis*. At the same time, compared to venomous spiders, a series of genomic changes related to muscle, aerobic respiration and metabolism of energy substances were observed in the genomes. These findings imply an evolutionary trade-off between the loss of venom glands and the enhancement of physical attack strategies.

Both gene family evolution and selection pressure shaped the genomic change related to the specialized physical attack of Uloboridae. For example, *myosin* and *twitchin* were under positive selection. Myosin, provides the driving force for muscle contraction as a molecular motor [24, 25], while twitchin, is located at the junction of muscle fibers and regulates the speed and force of muscle contraction [26]. The positive selection of these two genes suggests that they may play a role in the strengthening of muscle energy efficiency and contraction strength. Additionally, the gene family expansions of key proteins involved in muscle fiber connection, FHDC1 [29, 30], and the structural component of connective tissue, FBN1 [31], likely provided genetic resources for the optimization of muscle and connective tissue.

Aerobic respiration is the process that directly supplies energy to muscles. We found a higher expression of mitochondria-related genes in the legs of *O. sinensis* compared to *P. tepidariorum* (a venomous spider) (Figure 3C). In addition, physiological and biochemical measurements also indicate an advantage of uloborids in respiratory efficiency and related enzyme activity (Figure 3D, E). Moreover, the expansion of gene families involved in tracheal development [28], coupled with previous records that uloborids possess a more complex tracheal system compared to other web-building spiders [10, 11], all indicates that uloborids have the ability to provide sufficient oxygen supply for enhanced aerobic respiration.

The supply of energy substances is also crucial for sustained physical output. We found that the gene families involved in the transport of carbohydrates and organic acids have undergone expansion (Figure 4C), which may help uloborids enhanced utilization and metabolism of substrates and intermediate products in aerobic respiratory. Additionally, asprosin, encoded by an expanded gene family [31], acts

as a hormone regulating glucose homeostasis, potentially mobilizing energy reserves to further provide energy for long-term physical activity.

In conclusion, these adaptive evolutions, ranging from muscle to aerobic respiration and then to supply of energy substances, provide strong support for the exceptional physical endurance demands of Uloboridae.

Simultaneously, the absence of certain genes exclusively in uloborids raises concerns. These include protein c-ets-2, CC2D, and ataxin-7, which are transcription factors involved in various metabolic and developmental processes. Additionally, notable missing genes include the SOBP gene and two LRR genes. The former is involved in nervous and organ development, while the latter has a similar structural domain to LRR toxin genes, all of which are highly expressed in venom glands of model species. Although the association between these genes and the absence of spider venom glands remains unclear, they still hold potential for exploring the evolutionary mechanisms underlying this phenomenon.

For predators, there exists a pervasive evolutionary trade-off between chemical and physical attack strategies. Previous research has frequently favored the exploration of chemical strategies, particularly venom. However, against the backdrop of nearly all spiders being toxic predators, our study delves into the genetic basis underlying the alternative choice in this trade-off. Notably, in the *O. sinensis* genome, no reliable toxin gene was identified. Nevertheless, the adaptive evolution observed in Uloboridae, particularly pertaining to the respiratory system, motor system, and energy metabolism, may compensate for the reduced adaptability resulting from the absence of venom glands.

Methods

1. Sample collection and DNA extraction

Live *O. sinensis* were field-captured from Olympic Park, Chaoyang District, Beijing, China. To minimize the contamination of impurities in the digestive tract as much as possible, all samples were starvation reared for more than one week at room temperature. Genome DNA for both short and long read sequencing were isolated from the cephalothoraxes of adult female spiders using the Qiagen Blood & Cell Culture DNA Kit (QIAGEN, Hilden, Germany).

2. Observation of predation behavior and examination of fangs

We recorded a series of videos to observe the predation behavior. Wrapping duration was timed. If the spider stopped for more than 3 seconds, the timing also stopped but would continue if it began again. If the spider begins to soak (*O. sinensis* will emit liquid onto their prey before eating) or leave the prey, the video is terminated.

The dissected spider fangs were pasted onto a copper substrate at different angles, dried with a CO_2 critical point drying method, coated with gold, and then observed with the SEM (model: FEI Quanta 450).

3. Genome sequencing and genome size estimation

Short-insert libraries of *O. sinensis* were sequenced with the MGISEQ-2000 platform using paired-ends (PE) reads of 150 bp (BGI, Shenzhen, China). To remove low-quality reads and adapters, raw reads were trimmed by Trimmomatic v0.39 [46]. A total of 259.82 Gb of clean data were obtained for *O. sinensis* for survey analysis and assembly correction.

For long read sequencing, 'SMRTbell' (double-stranded DNA template capped by hairpin loops at both ends) libraries were constructed according to the standard protocol of PacBio using 15 kb of preparation solution (PacBio, California, USA). The high-fidelity (HiFi) libraries were sequenced on three SMRT cells on the PacBio Sequel II system in Circular Consensus Sequencing (CCS) mode at Novogene Technology Co. and generated 67.18 Gb HiFi data (3,967,026 reads) total [47].

To further improve the continuity of the assembled genomes, chromosome conformation capture (Hi-C) experiments were conducted [48]. Hi-C libraries were prepared following a published protocol with minor modifications [49]. For cross-linking, samples were fixed with 1% formaldehyde. The cross-linked DNA was digested with MboI restriction endonuclease and marked with biotin-14-dCTP to remove non-ligated DNA fragments. The ligated DNA was extracted with a QIAamp DNA Mini Kit (QIAGEN). The purified DNA was then sheared to ~350 bp fragments and followed by a standard Illumina library preparation protocol [50]. Hi-C sequencing of *O. sinensis* was conducted on the MGISEQ-2000 platform with PE 150 bp (BGI, Shenzhen, China). We then filtered the raw reads using Juicer v1.6.2 [51] to remove low-quality reads and adapters, yielding 201.99 Gb of clean data.

Before *de novo* assembly, we estimated the genome size of this species. Using the Illumina data, Jellyfish (v2.1.3) [52] was employed to calculate the frequency of each K-mer (k = 17–31). Then, the genome size of *O. sinensis* was estimated using a method based on K-mer distribution.

4. *De novo* Genome assembly and quality assessment

PacBio reads were first assembled using two *de novo* assemblers: hifiasm v0.15.2 [53] and wtdbg2 v2.5 [54]. The best assembly was selected according to the optimal continuity and completeness. The final version of contigs was polished with Racon v1.4.17 for three rounds (https://github.com/isovic/racon) based on long reads, and NextPolish v1.4.0 [55] using short reads. Contig level genome completeness assessment was performed using BUSCO v5.2.2 [15, 16]. Genome consistency assessment was evaluated by mapping the short reads to the genome with Minimap2 v2.24-r1122 [56] and Samtools v1.10 [57].

We used Hi-C-based proximity-guided assembly to generate chromosomal level genome assemblies for *O. sinensis*. Hi-C library sequencing data were mapped to the contig level genome using Juicer. The 3D-DNA v180922 [58] pipeline was executed to construct the chromosomes and correct the errors. We further performed correction with Juicebox Assembly Tools v1.11.08 [59]. The completeness of the chromosomal level assembly was assessed by BUSCO.

5. RNA extraction, sequencing and expression analysis

Different tissue samples (spinneret, leg, brain, gut, silk gland, pedipalp, chelicera and abdomen) from adult female *O. sinensis* and eggs (development stage undetermined) were dissected for total RNA extraction using an RNAsimple Total RNA kit (TIANGEN, Beijing, China). The RNA-seq libraries were constructed with insert sizes of ~150 bp and sequenced on the NovaSeq 6000 platform. We produced ~6 Gb of data per sample. Low-quality reads, reads with adapters, and unknown bases were filtered using Trimmomatic.

In addition, legs from adult female *P. tepidariorum* were subjected to transcriptome analysis according to the above process (see Availability of data and materials), and transcriptome data from four tissue transcriptomes (PRJNA934108, including brain, ovary, silk gland and venom gland) have been downloaded from the Sequence Read Archive (SRA) database.

Clean reads were aligned to the genome using Hisat2 [60], followed by the quantification of all samples with HTSeq [61] to determine the count value. Subsequently, TPMs were derived through automated scripts.

In the differential expression analysis across two species, only Reciprocal Best Hits (RBH) genes were extracted for quantification. To facilitate comparison, ortholog (RBH) gene IDs of *O. sinensis* were replaced by *P. tepidariorum* gene IDs for comparison and figure illustration. Finally, differential expression analysis was conducted by R package limma [62].

6. Genome annotation

The RepeatModeler v2.0.2 [63] and RepeatMasker v4.1.2-p1 [64] pipelines were used to annotate repetitive sequences in the genome.

Gene annotation was based on the braker v2.1.6 [65] pipeline, which combines the whole protein sequences of the 9 species in this study (Additional file 4: Table S4) and more than 320 Gb multi tissue transcriptome data for comprehensive annotation.

Gene function annotation is based on NCBI-Nr (http://www.ncbi.nlm.nih.gov), Swiss-Prot (http://www.uniprot.org/) and EggNOG v5.0 (http://eggnog5.embl.de/) databases. The tRNA was predicted using the program tRNAscan-SE v2.09 [66]. Other non-coding RNAs were annotated with the Rfam v14.8 [67] database through infernal v1.1.4 [68].

Among the other species involved in this study, *P. pseudoannulata* and *D. plantarius* only have assembled sequence data currently. The genome of *L. elegans* has high assembly quality, but the protein BUSCO score is only 63.7%; *U. diversus* only has 15,750 annotated protein coding genes, significantly less than other spider species. To obtain more reliable results for downstream analysis, we annotated the genomes of the aforementioned species based on transcriptome data. For *P. pseudoannulata*, *D. plantarius* and *L. elegans*, we used our annotations, and for *U. diversus*, we used our annotation to supplement the original one. All annotation strategies are based on transcriptome data from the SRA (Additional file 4: Table S18) database and carried out through the TransDecoder pipelines (v5.5.0, https://github.com/TransDecoder). We also added *Deinopis* sp. of Deinopidae and assembled all of its proteins sequence using transcriptome data (DRR297048), quality control was done using fastp version

0.21.0 [69]. De novo assemblies were done using Trinity-v2.11.0 [70] under default settings. ORF prediction was done using TransDecoder. Redundancy reduction was done with CD-HIT version 4.8.1 (-c 0.98 -n 10) [71]. All the newly provided annotations mentioned above have a protein BUSCO score of over 90% (Additional file 4: Table S2).

7. Orthologous gene identification, phylogenetic and synteny analysis

We used OrthoFinder v2.5.4 [72] to analyze the annotation information of species in this study (Additional file 4: Table S5). In the pipeline, Mafft v7.453 [73] was used to perform multiple sequence alignment, blastp v2.9.0+ [74] was used to perform sequence searches, and the phylogenetic tree was constructed using IQ-TREE v2.2.0 [75]. The calibration points are from fossil specimens [21]. The analysis was run twice, once to calculate the divergence time of different species, and the second to calculate the expansion and contraction of gene families. The latter did not include *Deinopis* sp., as the only transcriptome data cannot determine the number of gene copies. (Figure 4A).

Collinearity analysis was conducted between *O. sinensis* and other species with chromosome level genomes (*T. antipodiana*, *A. bruennichi*, *L. elegans*, *U. diversus, P. pseudoannulata*, *D. plantarius* and *E. davidi*.) using the MCscan pipeline (Python version) in the jcvi toolkit [76].

8. Test for selection pressure and gene family expansions/contractions

For all species (Figure 2B), we used the *O. sinensis* genome as a reference to extract the RBH based on blastp. Finally, 5,848 RBH clusters (Additional file 4: Table S19) were retained for analysis.

To scan for genes under positive selection in *O. sinensis*, RBH clusters were used for the branch site model analysis using CODEML in PAML v4.9j toolkit [77]. Each gene family sets *O. sinensis* and *U. diversus* as foreground branches (Figure 2B). “Model A” and “Model A-null” models were compared. “Model A” assumes that the selection pressure of foreground branches is greater than that of background branches, and “Model A-null” is an alternative hypothesis.

To statistically test which genes of Uloboridae are under relaxed purifying selection, we used RELAX in the HYPHY v2.5.2 [78] toolkit to infer the free relaxation parameter k at the node of *O. sinensis* and *U. diversus* branches for genes shared by all species in the Phylogeny of Figure 2B (Additional file 4: Table S4). The relaxation parameter k is an exponent for selection parameters between the foreground

and the background branches. A k > 1 suggests selection is more intensified in the foreground branch vs. the background branch and vice versa.

For gene family expansions/contractions, mcmctree in the PAML v4.9j toolkit was used to estimate the divergence time of each node in the phylogenetic tree of OrthoFinder pipeline results (without *Deinopis* sp.) (Figure 4A). Next, we used CAFE v4.2 [27] under the default parameters to analyze the gene family expansions and contractions of nine spider species.

9. Identification of HCEs

To identify the HCEs, we initially generated pairwise sequence alignments across all nine spider genomes (Figure 4A) with LASTZ v1.04.15 [79] and chainNet [80], using the *P. tepidariorum* genome as the reference. We then used MULTIZ v11.2 [81] to combine the pairwise alignments into multiple sequence alignments. Subsequently, we ran phyloFit in the PHAST package [82] with the topology from OrthoFinder to estimate the neutral ('nonconserved') model based on fourfold degenerate sites. With the nonconserved model as input, we ran phastCons [83] to estimate conserved models with its intrinsic function and predicted the HCEs.

The distribution of HCEs in exons, introns, 2,000 bp upstream and 2,000 bp downstream of genes and intergenic regions was summarized with Annovar (https://annovar.openbioinformatics.org/) [84, 85] based on the genome annotation information of *O. sinensis* or *P. tepidariorum*.

10. Homologs of toxin gene family identification and analysis

Previous datasets were used as references (Additional file 6) [36, 86–88], and we searched for target genes using blastp v2.9.0+ (E-value less than 1e-10, matching length greater than 70% of the reference sequence, and hit area mismatch less than 30%). We also established hidden Markov models (HMM) for different types of toxin proteins based on the database and further confirmed the results obtained from blastp using HMMER v 3.3 [89]. To search for hidden toxin-related pseudogenes in *O. sinensis*, we referred to the identification criteria of human genome pseudogenes [40], and used blastn v2.9.0+ and blastx v2.9.0+ to search for candidates.

11. Physiological index measurement

Assays were performed as described previously [90]. CO_2 production rate was used as a proxy for

metabolic rate (MR). The assays were conducted in a closed-circuit system with a volume of 73.3 ml at a temperature of 25 °C, a pressure of 100.6 kPa, and a flow rate of 110 ml/min. MR was calculated as the amount of CO_2 produced per gram of body mass per second, using the equation MR = MCO_2/T/body mass, where MCO_2 represents the amount of oxygen substance (in mol). To put spiders into a state of fatigue, we stimulated the spider's legs with a dissecting needle and kept it in a high-intensity state of exercise for ten minutes.

Enzyme activity was measured using the corresponding reagent kit (Wuhan Mosak Biotechnology Co., Ltd. KT50129, KT50577, KT42310, KT41589, KT87867). Female spider individuals in a resting state were fixed in liquid nitrogen after weighing and stored at -80 °C. Prior to testing, homogenize the samples were homogenized and diluted to 500 μl as the test solution following the supplier's protocol

Additional Files

Additional file 1–3: Hunting video.

Additional file 4: Table S1. Genome survey prediction of *Octonoba sinensis*. **Table S2.** Genome assembly. **Table S3.** Repeat sequences of *Octonoba sinensis* genome. **Table S4.** Transcriptome assembly and genomes used in this study. **Table S5.** Genes under positive selection pressure on the node of the family Uloboridae. **Table S6.** Transcriptome differential expression analysis of the legs of *Parasteatoda tepidariorum* and *Octonoba sinensis*. **Table S7.** Genes list of significantly expanded gene families in *Octonoba sinensis*. **Table S8.** New emergent gene family members in the *Octonoba sinensis* genome. **Table S9.** All HCEs, using the *Parasteatoda tepidariorum* genome as a reference. **Table S10.** Missing HCEs in *Octonoba sinensis* and *Uloborus diversus*. **Table S11.** Specific missing orthologous groups in *Octonoba sinensis* and *Uloborus diversus*. **Table S12.** Genes under relaxed selection pressure in Uloboridae. **Table S13.** *Parasteatoda tepidariorum* venom gland-specific expression module. **Table S14.** Quantitative distribution of different toxin homologs across species. **Table S15.** "Pseudogene" blastx. **Table S16.** Protein domain search. **Table S17.** Expression levels (TPM) in different organizations of the genes connected by the red band to the *Parasteatoda tepidariorum* toxin gene in Figure 7h. **Table S18.** The SRA data used for genome annotation. **Table S19.** Clusters of Reciprocal Best Hits (RBH)

and RBH between *Octonoba sinensis* and *Parasteatoda tepidariorum*.

Additional file 5: Figure S1. Recognizable elements of the *Octonoba sinensis* genome. **Figure S2.** GO enrichment analysis of genes expressed at higher levels (Fold-change > 4, $p < 0.05$) in *O. sinensis* legs relative to those in *P. tepidariorum* legs. **Figure S3.** Distribution of all Highly-Conserved Elements (HCEs) in *P. tepidariorum* and missing HCEs and genes in *O. sinensis*. **Figure S4.** Phylogenetic tree of homologs of neurotoxin genes (Latrotoxin).

Additional file 6: Dataset of toxin reference genes.

Acknowledgments

We are grateful to Prof. Zhonge Hou and Assis. Prof. Fengyuan Li for academic suggestions. We thank Wei Wang at Guangxi Normal University for her suggestions on anatomical techniques. Sincere thanks to Dr. Nadia Ayoub and Dr. Sandra Correa-Garhwal for their careful review and valuable suggestions on this article. Finally, Y.M.Z. wants to thank Lingling Liu, in particular, for the invaluable support over the years.

Authors' contributions

S.Q.L. and Y.M.Z. conceived and designed the project. Y.X.S. and Y.M.Z. finished the genome assembly and annotation. B.Y.Z., P.Y.J., Y.X.S., and Y.M.Z executed the comparison analysis. Y.J.L. and Z.Z. identified the species and provided the spider pictures used in the article. T.Y.J assembled and annotated transcriptome data of *Deinopis* sp. Y.W. recorded the original hunting videos and Y.M.Z. edited the videos. X.T.H. sent a new transcriptome for testing and analysis. All authors participated in the discussion and reviewed the final manuscript.

Funding

This study was supported by the Strategic Priority Research Program of the Chinese Academy of Sciences (XDB31000000), and the Program of National Natural Sciences Foundation of China (NSFC–32170447, NSFC–32370490).

Availability of data and materials

All data generated or analyzed during this study are included in this article, its supplementary

information files, and publicly available repositories. Original sequencing data has been uploaded to the NCBI database (PRJNA1019401) and ScienceDB (doi.org/10.57760/sciencedb.09166). New chromosome-level genome assemblies are deposited in the gigaDB Digital Repository ().

Declarations

Ethics approval and consent to participate

Not applicable.

Consent for publication

Not applicable.

Competing interests

The authors declare that they have no competing interests.

References

1. Foelix RF, Erb B. Mesothelae have venom glands. in: 2010.
2. NMBE - World Spider Catalog. https://wsc.nmbe.ch/. Accessed,2023.
3. Forster RR, Platnick NI. A review of the archaeid spiders and their relatives, with notes on the limits of the superfamily Palpimanoidea (Arachnida, Araneae). Bulletin of the AMNH; v. 178, article 1. in: 1984.
4. Rix MG. A Review of the Tasmanian Species of Pararchaeidae and Holarchaeidae (Arachnida, Araneae). The Journal of Arachnology 2005;**33**(1):135-152.
5. Opell BD. Revision of the genera and tropical American species of the spider family Uloboridae. Bulletin of the Museum of Comparative Zoology at Harvard College 1979;**148**:443-549.
6. Robinson MH, Olazarri J. Units of behavior and complex sequences in the predatory behavior of Argiope argentata (Fabricius): (Araneae: Araneidae). in: 1971.
7. Weng JL, Barrantes G, Eberhard WG. Feeding by Philoponella vicina (Araneae, Uloboridae) and how uloborid spiders lost their venom glands. Can J Zool 2006;**84**(12):1752-1762. doi:10.1139/z06-149.
8. Eberhard WG, Barrantes G, Weng JL. Tie them up tight: wrapping by Philoponella vicina spiders breaks, compresses and sometimes kills their prey. Sci Nat-Heidelberg 2006;**93**(5):251-4. doi:10.1007/s00114-006-0094-1.
9. Lubin YD. Web buiding and prey capture in the Uloboridae. Spiders:Webs, Behavior, and Evolution. 1986:132-171.
10. Opell BD. The relationship of book lung and tracheal systems in the spider family uloboridae. J Morphol 1990;**206**(2):211-216. doi:10.1002/jmor.1052060207.
11. Opell BD. The respiratory complementarity of spider book lung and tracheal systems. J Morphol

1998;**236**(1):57-64. doi:10.1002/(SICI)1097-4687(199804)236:1<57::AID-JMOR4>3.0.CO;2-L.

12. de Plancy VC. Arachnides recueillis aux environs de Pékin. In: Simon E, ed Annales de la Société Entomologique de France. Saint-Germain; 1880.
13. Rainer F. Biology of Spiders.: Oxford university press; 2011.
14. Wang X, Wang Y, Yang Z, et al. On the Karyotype of Octonnba sinensis. Journal of Hebei Normal University (Natural Science) 1997;(04):423-426.
15. Seppey M, Manni M, Zdobnov EM. BUSCO: Assessing Genome Assembly and Annotation Completeness. Methods Mol Biol 2019;**1962**:227-245. doi:10.1007/978-1-4939-9173-0_14.
16. Simao FA, Waterhouse RM, Ioannidis P, et al. BUSCO: assessing genome assembly and annotation completeness with single-copy orthologs. Bioinformatics 2015;**31**(19):3210-2. doi:10.1093/bioinformatics/btv351.
17. Hu W, Jia A, Ma S, et al. A molecular atlas reveals the tri-sectional spinning mechanism of spider dragline silk. Nat Commun 2023;**14**(1). doi:10.1038/s41467-023-36545-6.
18. Fan Z, Yuan T, Liu P, et al. A chromosome-level genome of the spiderTrichonephila antipodiana reveals the genetic basis of its polyphagy and evidence of an ancient whole-genome duplication event. Gigascience 2021;**10**(3). doi:10.1093/gigascience/giab016.
19. Liu S, Aagaard A, Bechsgaard J, et al. DNA Methylation Patterns in the Social Spider, Stegodyphus dumicola. Genes-Basel 2019;**10**(2):137. doi:10.3390/genes10020137.
20. Huerta-Cepas J, Szklarczyk D, Heller D, et al. eggNOG 5.0: a hierarchical, functionally and phylogenetically annotated orthology resource based on 5090 organisms and 2502 viruses. Nucleic Acids Res 2019;**47**(D1):D309-D314. doi:10.1093/nar/gky1085.
21. Magalhaes I, Azevedo G, Michalik P, et al. The fossil record of spiders revisited: implications for calibrating trees and evidence for a major faunal turnover since the Mesozoic. Biol Rev Camb Philos Soc 2019. doi:10.1111/brv.12559.
22. Shao L, Zhao Z, Li S. Is phenotypic evolution affected by spiders' construction behaviors? Syst Biol 2022. doi:10.1093/sysbio/syac063.
23. Wheeler WC, Coddington JA, Crowley LM, et al. The spider tree of life: phylogeny of Araneae based on target-gene analyses from an extensive taxon sampling. Cladistics 2017;**33**(6):574-616. doi:10.1111/cla.12182.
24. Foth BJ, Goedecke MC, Soldati D. New insights into myosin evolution and classification. Proc Natl Acad Sci U S A 2006;**103**(10):3681-6. doi:10.1073/pnas.0506307103.
25. Squire J. Special Issue: The Actin-Myosin Interaction in Muscle: Background and Overview. Int J Mol Sci 2019;**20**(22). doi:10.3390/ijms20225715.
26. Ayme-Southgate A, Vigoreaux J, Benian G, et al. Drosophila has a twitchin/titin-related gene that appears to encode projectin. Proc Natl Acad Sci U S A 1991;**88**(18):7973-7. doi:10.1073/pnas.88.18.7973.
27. De Bie T, Cristianini N, Demuth JP, et al. CAFE: a computational tool for the study of gene family evolution. Bioinformatics 2006;**22**(10):1269-71. doi:10.1093/bioinformatics/btl097.
28. Matusek T, Djiane A, Jankovics F, et al. The Drosophila formin DAAM regulates the tracheal cuticle pattern through organizing the actin cytoskeleton. Development 2006;**133**(5):957-66. doi:10.1242/dev.02266.
29. Iskratsch T, Ehler E. Formin-g muscle cytoarchitecture. Bioarchitecture 2011;**1**(2):66-68.

doi:10.4161/bioa.1.2.15467.
30. Valencia DA, Quinlan ME. Formins. Curr Biol 2021;**31**(10):R517-R522. doi:10.1016/j.cub.2021.02.047.
31. Summers KM, Bush SJ, Davis MR, et al. Fibrillin-1 and asprosin, novel players in metabolic syndrome. Mol Genet Metab 2023;**138**(1):106979. doi:10.1016/j.ymgme.2022.106979.
32. Supek F, Bosnjak M, Skunca N, et al. REVIGO summarizes and visualizes long lists of gene ontology terms. Plos One 2011;**6**(7):e21800. doi:10.1371/journal.pone.0021800.
33. Zhu B, Jin P, Zhang Y, et al. Genomic and transcriptomic analyses support a silk gland origin of spider venom glands. Bmc Biol 2023;**21**(1). doi:10.1186/s12915-023-01581-7.
34. Luo J, Ding Y, Peng Z, et al. Molecular diversity and evolutionary trends of cysteine-rich peptides from the venom glands of Chinese spider Heteropoda venatoria. Sci Rep 2021;**11**(1):3211. doi:10.1038/s41598-021-82668-5.
35. Wang Z, Zhu K, Li H, et al. Chromosome-level genome assembly of the black widow spider Latrodectus elegans illuminates composition and evolution of venom and silk proteins. Gigascience 2022;**11**. doi:10.1093/gigascience/giac049.
36. Zhu B, Jin P, Hou Z, et al. Chromosomal-level genome of a sheet-web spider provides insight into the composition and evolution of venom. Mol Ecol Resour 2022;**22**(6):2333-2348. doi:10.1111/1755-0998.13601.
37. Escuer P, Pisarenco VA, Fernandez-Ruiz AA, et al. The chromosome-scale assembly of the Canary Islands endemic spider Dysdera silvatica (Arachnida, Araneae) sheds light on the origin and genome structure of chemoreceptor gene families in chelicerates. Mol Ecol Resour 2022;**22**(1):375-390. doi:10.1111/1755-0998.13471.
38. Chen M, Blum D, Engelhard L, et al. Molecular architecture of black widow spider neurotoxins. Nat Commun 2021;**12**(1):6956. doi:10.1038/s41467-021-26562-8.
39. Luddecke T, Herzig V, von Reumont BM, et al. The biology and evolution of spider venoms. Biol Rev Camb Philos Soc 2022;**97**(1):163-178. doi:10.1111/brv.12793.
40. Zhang Z, Carriero N, Zheng D, et al. PseudoPipe: an automated pseudogene identification pipeline. Bioinformatics 2006;**22**(12):1437-9. doi:10.1093/bioinformatics/btl116.
41. Protas ME, Trontelj P, Patel NH. Genetic basis of eye and pigment loss in the cave crustacean, *Asellus aquaticus*. Proceedings of the National Academy of Sciences 2011;**108**(14):5702-5707. doi:doi:10.1073/pnas.1013850108.
42. Gore AV, Tomins KA, Iben J, et al. An epigenetic mechanism for cavefish eye degeneration. bioRxiv 2017:199018. doi:10.1101/199018.
43. Mojaddidi H, Fernandez FE, Erickson PA, et al. Embryonic origin and genetic basis of cave associated phenotypes in the isopod crustacean Asellus aquaticus. Sci Rep-Uk 2018;**8**(1):16589. doi:10.1038/s41598-018-34405-8.
44. Piatigorsky J. A Genetic Perspective on Eye Evolution: Gene Sharing, Convergence and Parallelism. Evolution: Education and Outreach 2008;**1**(4):403-414. doi:10.1007/s12052-008-0077-0.
45. Krishnan J, Rohner N. Cavefish and the basis for eye loss. Philos Trans R Soc Lond B Biol Sci 2017;**372**(1713). doi:10.1098/rstb.2015.0487.
46. Bolger AM, Lohse M, Usadel B. Trimmomatic: a flexible trimmer for Illumina sequence data. Bioinformatics 2014;**30**(15):2114-20. doi:10.1093/bioinformatics/btu170.

47. Wenger AM, Peluso P, Rowell WJ, et al. Accurate circular consensus long-read sequencing improves variant detection and assembly of a human genome. Nat Biotechnol 2019;**37**(10):1155-1162. doi:10.1038/s41587-019-0217-9.
48. Lu L, Liu X, Huang W, et al. Robust Hi-C Maps of Enhancer-Promoter Interactions Reveal the Function of Non-coding Genome in Neural Development and Diseases. Mol Cell 2020;**79**(3):521-534.e15. doi:10.1016/j.molcel.2020.06.007.
49. Rao SS, Huntley MH, Durand NC, et al. A 3D map of the human genome at kilobase resolution reveals principles of chromatin looping. Cell 2014;**159**(7):1665-80. doi:10.1016/j.cell.2014.11.021.
50. Meyer M, Kircher M. Illumina sequencing library preparation for highly multiplexed target capture and sequencing. Cold Spring Harb Protoc 2010;**2010**(6):pdb.prot5448. doi:10.1101/pdb.prot5448.
51. Durand NC, Shamim MS, Machol I, et al. Juicer Provides a One-Click System for Analyzing Loop-Resolution Hi-C Experiments. Cell Syst 2016;**3**(1):95-8. doi:10.1016/j.cels.2016.07.002.
52. Marcais G, Kingsford C. A fast, lock-free approach for efficient parallel counting of occurrences of k-mers. Bioinformatics 2011;**27**(6):764-70. doi:10.1093/bioinformatics/btr011.
53. Cheng H, Concepcion GT, Feng X, et al. Haplotype-resolved de novo assembly using phased assembly graphs with hifiasm. Nat Methods 2021;**18**(2):170-175. doi:10.1038/s41592-020-01056-5.
54. Ruan J, Li H. Fast and accurate long-read assembly with wtdbg2. Nat Methods 2020;**17**(2):155-158. doi:10.1038/s41592-019-0669-3.
55. Hu J, Fan J, Sun Z, et al. NextPolish: a fast and efficient genome polishing tool for long-read assembly. Bioinformatics 2020;**36**(7):2253-2255. doi:10.1093/bioinformatics/btz891.
56. Li H. Minimap2: pairwise alignment for nucleotide sequences. Bioinformatics 2018;**34**(18):3094-3100. doi:10.1093/bioinformatics/bty191.
57. Li H, Handsaker B, Wysoker A, et al. The Sequence Alignment/Map format and SAMtools. Bioinformatics 2009;**25**(16):2078-9. doi:10.1093/bioinformatics/btp352.
58. Dudchenko O, Batra SS, Omer AD, et al. De novo assembly of the Aedes aegypti genome using Hi-C yields chromosome-length scaffolds. Science 2017;**356**(6333):92-95. doi:10.1126/science.aal3327.
59. Dudchenko O, Shamim MS, Batra S, et al. The Juicebox Assembly Tools module facilitates de novo assembly of mammalian genomes with chromosome-length scaffolds for under $1000. Cold Spring Harbor: Cold Spring Harbor Laboratory Press; 2018.
60. Kim D, Paggi JM, Park C, et al. Graph-based genome alignment and genotyping with HISAT2 and HISAT-genotype. Nat Biotechnol 2019;**37**(8):907-915. doi:10.1038/s41587-019-0201-4.
61. Anders S, Pyl PT, Huber W. HTSeq--a Python framework to work with high-throughput sequencing data. Bioinformatics 2015;**31**(2):166-9. doi:10.1093/bioinformatics/btu638.
62. Ritchie ME, Phipson B, Wu D, et al. limma powers differential expression analyses for RNA-sequencing and microarray studies. Nucleic Acids Res 2015;**43**(7):e47. doi:10.1093/nar/gkv007.
63. Flynn JM, Hubley R, Goubert C, et al. RepeatModeler2 for automated genomic discovery of transposable element families. Proc Natl Acad Sci U S A 2020;**117**(17):9451-9457. doi:10.1073/pnas.1921046117.
64. Tarailo-Graovac M, Chen N. Using RepeatMasker to identify repetitive elements in genomic sequences. Curr Protoc Bioinformatics 2009;**Chapter 4**:4.10.1-4.10.14. doi:10.1002/0471250953.bi0410s25.
65. Bruna T, Hoff KJ, Lomsadze A, et al. BRAKER2: automatic eukaryotic genome annotation with GeneMark-EP+ and AUGUSTUS supported by a protein database. NAR Genom Bioinform

2021;**3**(1):lqaa108. doi:10.1093/nargab/lqaa108.

66. Chan PP, Lin BY, Mak AJ, et al. tRNAscan-SE 2.0: improved detection and functional classification of transfer RNA genes. Nucleic Acids Res 2021;**49**(16):9077-9096. doi:10.1093/nar/gkab688.
67. Kalvari I, Nawrocki EP, Ontiveros-Palacios N, et al. Rfam 14: expanded coverage of metagenomic, viral and microRNA families. Nucleic Acids Res 2021;**49**(D1):D192-D200. doi:10.1093/nar/gkaa1047.
68. Nawrocki EP, Eddy SR. Infernal 1.1: 100-fold faster RNA homology searches. Bioinformatics 2013;**29**(22):2933-5. doi:10.1093/bioinformatics/btt509.
69. Chen S, Zhou Y, Chen Y, et al. fastp: an ultra-fast all-in-one FASTQ preprocessor. Bioinformatics 2018;**34**(17):i884-i890. doi:10.1093/bioinformatics/bty560.
70. Grabherr MG, Haas BJ, Yassour M, et al. Full-length transcriptome assembly from RNA-Seq data without a reference genome. Nat Biotechnol 2011;**29**(7):644-52. doi:10.1038/nbt.1883.
71. Li W, Godzik A. Cd-hit: a fast program for clustering and comparing large sets of protein or nucleotide sequences. Bioinformatics 2006;**22**(13):1658-9. doi:10.1093/bioinformatics/btl158.
72. Emms DM, Kelly S. OrthoFinder: phylogenetic orthology inference for comparative genomics. Genome Biol 2019;**20**(1):238. doi:10.1186/s13059-019-1832-y.
73. Katoh K, Standley DM. MAFFT multiple sequence alignment software version 7: improvements in performance and usability. Mol Biol Evol 2013;**30**(4):772-80. doi:10.1093/molbev/mst010.
74. Camacho C, Coulouris G, Avagyan V, et al. BLAST+: architecture and applications. Bmc Bioinformatics 2009;**10**:421. doi:10.1186/1471-2105-10-421.
75. Minh BQ, Schmidt HA, Chernomor O, et al. IQ-TREE 2: New Models and Efficient Methods for Phylogenetic Inference in the Genomic Era. Mol Biol Evol 2020;**37**(5):1530-1534. doi:10.1093/molbev/msaa015.
76. Tang H, Bowers JE, Wang X, et al. Synteny and collinearity in plant genomes. Science 2008;**320**(5875):486-8. doi:10.1126/science.1153917.
77. Yang Z. PAML 4: phylogenetic analysis by maximum likelihood. Mol Biol Evol 2007;**24**(8):1586-91. doi:10.1093/molbev/msm088.
78. Kosakovsky PS, Poon A, Velazquez R, et al. HyPhy 2.5-A Customizable Platform for Evolutionary Hypothesis Testing Using Phylogenies. Mol Biol Evol 2020;**37**(1):295-299. doi:10.1093/molbev/msz197.
79. Harris RS. *IMPROVED PAIRWISE ALIGNMENT OF GENOMIC DNA*. Doctor of Philosophy, The Pennsylvania State University, 2007.
80. Kent WJ, Baertsch R, Hinrichs A, et al. Evolution's cauldron: duplication, deletion, and rearrangement in the mouse and human genomes. Proc Natl Acad Sci U S A 2003;**100**(20):11484-9. doi:10.1073/pnas.1932072100.
81. Blanchette M, Kent WJ, Riemer C, et al. Aligning multiple genomic sequences with the threaded blockset aligner. Genome Res 2004;**14**(4):708-15. doi:10.1101/gr.1933104.
82. Hubisz MJ, Pollard KS, Siepel A. PHAST and RPHAST: phylogenetic analysis with space/time models. Brief Bioinform 2011;**12**(1):41-51. doi:10.1093/bib/bbq072.
83. Siepel A, Bejerano G, Pedersen JS, et al. Evolutionarily conserved elements in vertebrate, insect, worm, and yeast genomes. Genome Res 2005;**15**(8):1034-50. doi:10.1101/gr.3715005.
84. Yang H, Wang K. Genomic variant annotation and prioritization with ANNOVAR and wANNOVAR. Nat Protoc 2015;**10**(10):1556-66. doi:10.1038/nprot.2015.105.

85. Wang K, Li M, Hakonarson H. ANNOVAR: functional annotation of genetic variants from high-throughput sequencing data. Nucleic Acids Res 2010;**38**(16):e164. doi:10.1093/nar/gkq603.
86. Haney RA, Matte T, Forsyth FS, et al. Alternative Transcription at Venom Genes and Its Role as a Complementary Mechanism for the Generation of Venom Complexity in the Common House Spider. Front Ecol Evol 2019;**7**. doi:10.3389/fevo.2019.00085.
87. Pineda SS, Chaumeil PA, Kunert A, et al. ArachnoServer 3.0: an online resource for automated discovery, analysis and annotation of spider toxins. Bioinformatics 2018;**34**(6):1074-1076. doi:10.1093/bioinformatics/btx661.
88. Wang Z, Zhu K, Li H, et al. Chromosome-level genome assembly of the black widow spider Latrodectus elegans illuminates composition and evolution of venom and silk proteins. Gigascience 2022;**11**. doi:10.1093/gigascience/giac049.
89. Mistry J, Finn RD, Eddy SR, et al. Challenges in homology search: HMMER3 and convergent evolution of coiled-coil regions. Nucleic Acids Res 2013;**41**(12):e121. doi:10.1093/nar/gkt263.
90. Roberts SP, Harrison JF, Dudley R. Allometry of kinematics and energetics in carpenter bees (Xylocopa varipuncta) hovering in variable-density gases. J Exp Biol 2004;**207**(Pt 6):993-1004. doi:10.1242/jeb.00850.

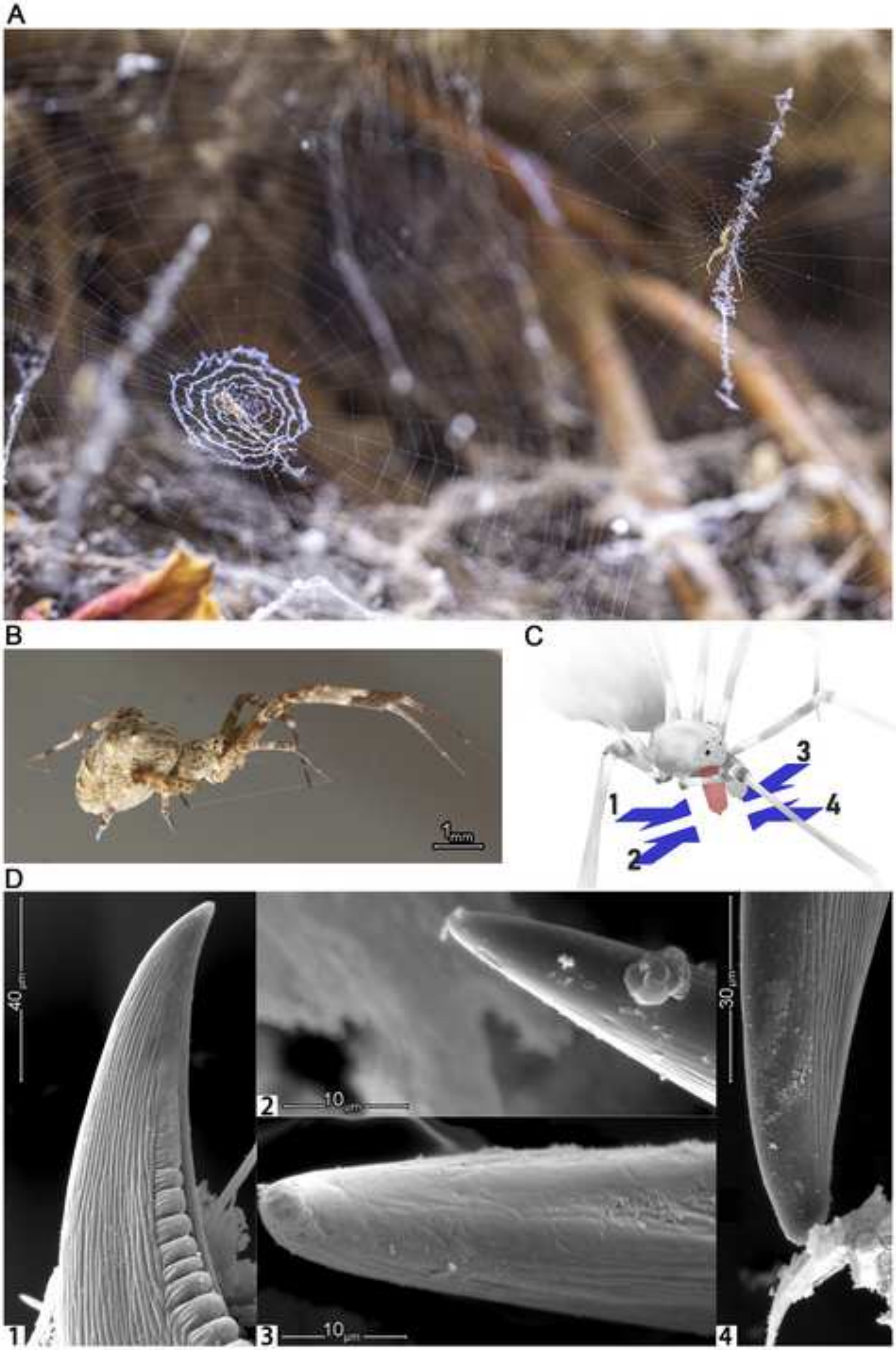
A
B
1mm
C
1
2
3
4
D
40 µm
2
10 µm
3
10 µm
30 µm
1
4

Figure 2

Click here to access/download;Figure;Fig.2-giga-re.jpg

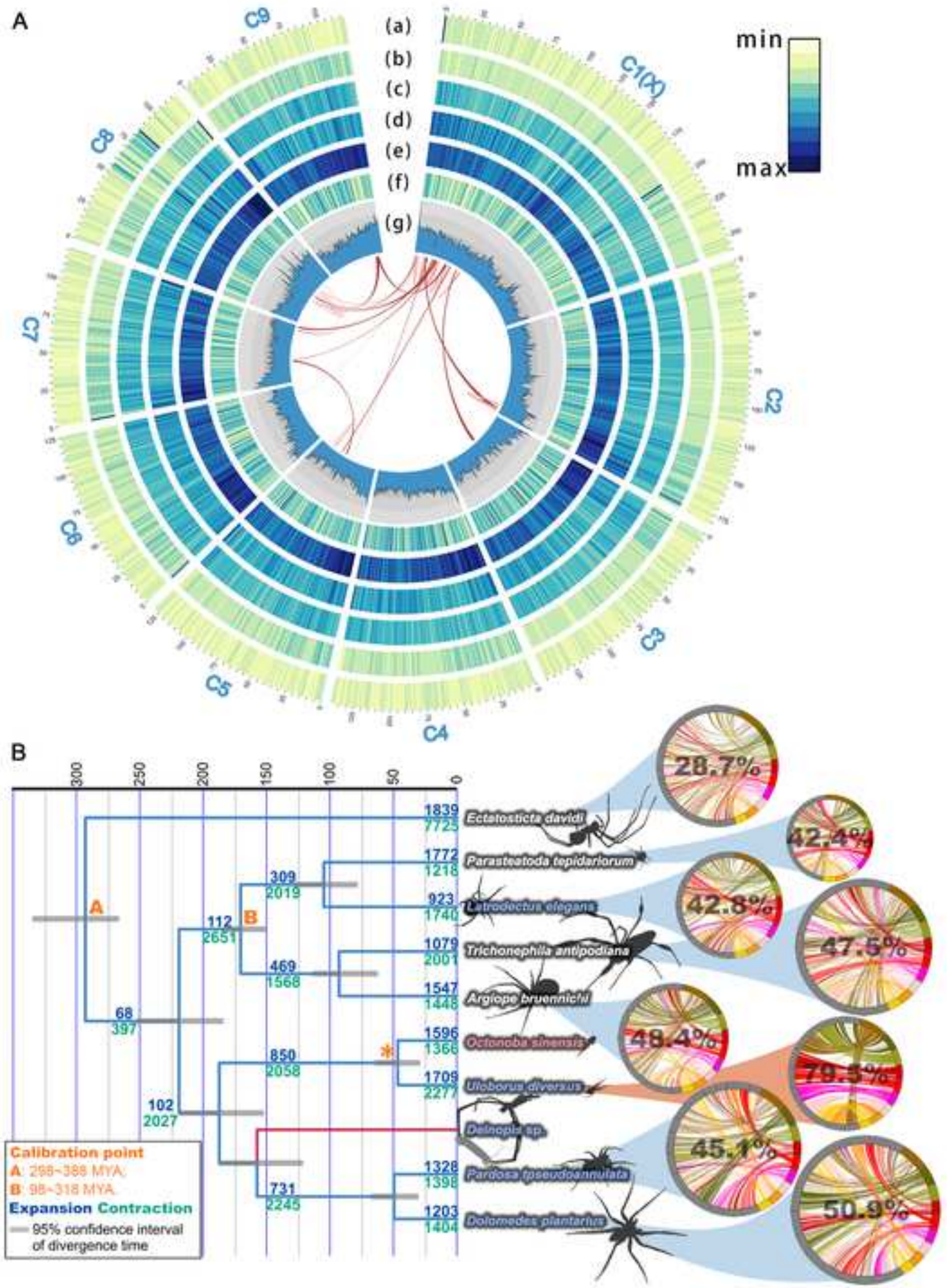

Figure 3

Click here to access/download;Figure;Fig.3-giga-re.jpg

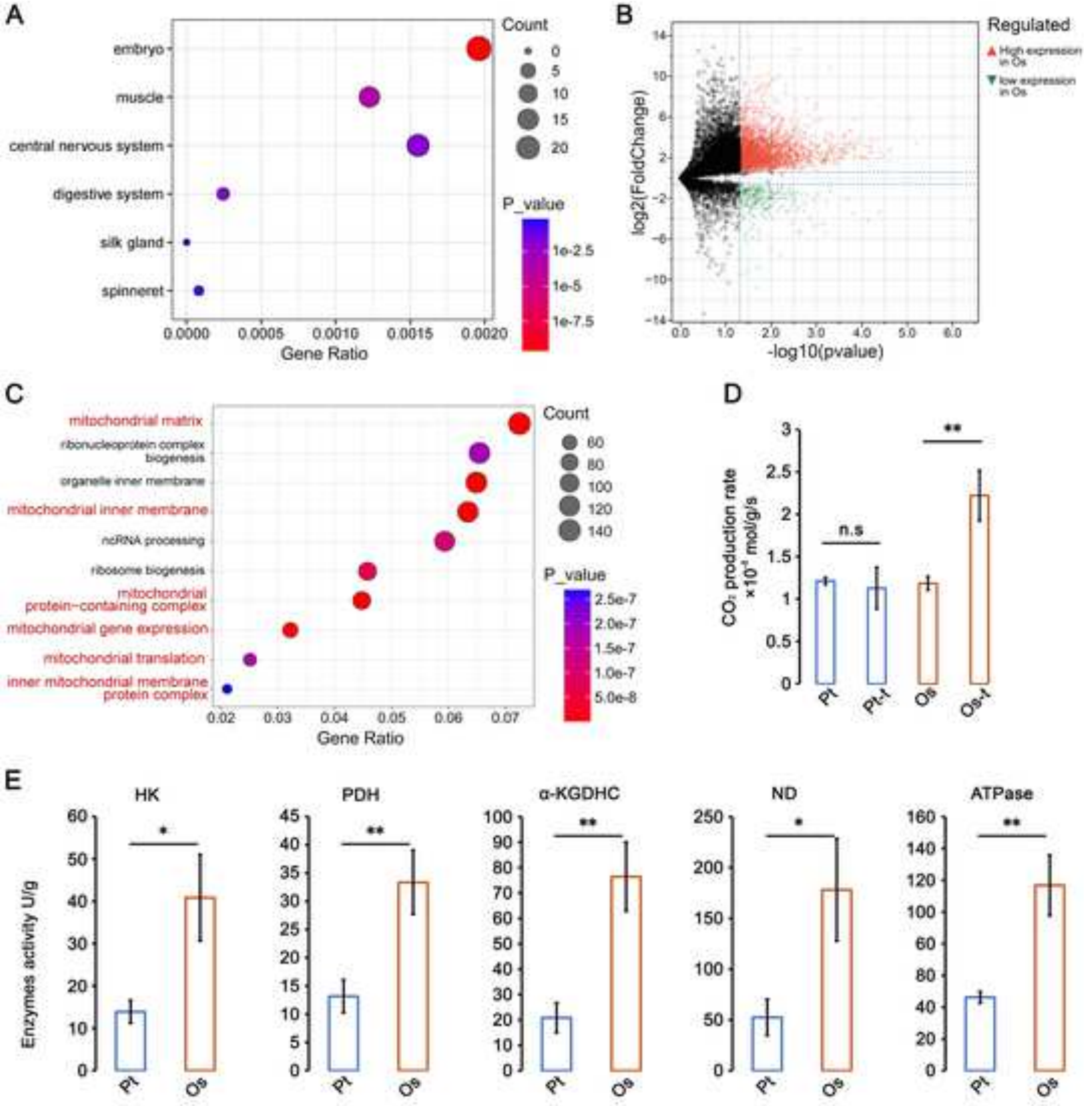

Figure 4

Click here to access/download;Figure;Fig.4-giga-re.jpg

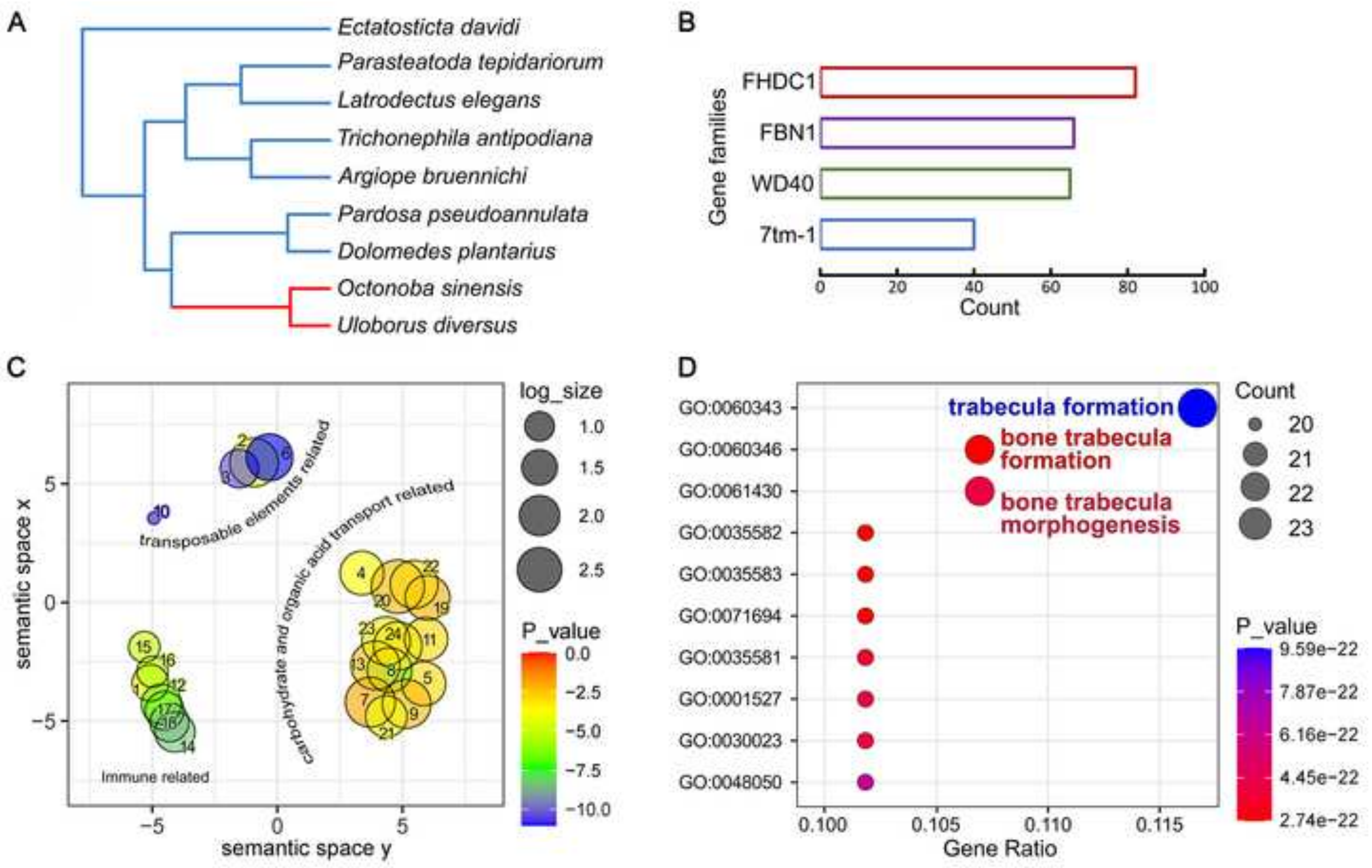

Figure 5

Click here to access/download;Figure;Fig.5-giga-re.jpg

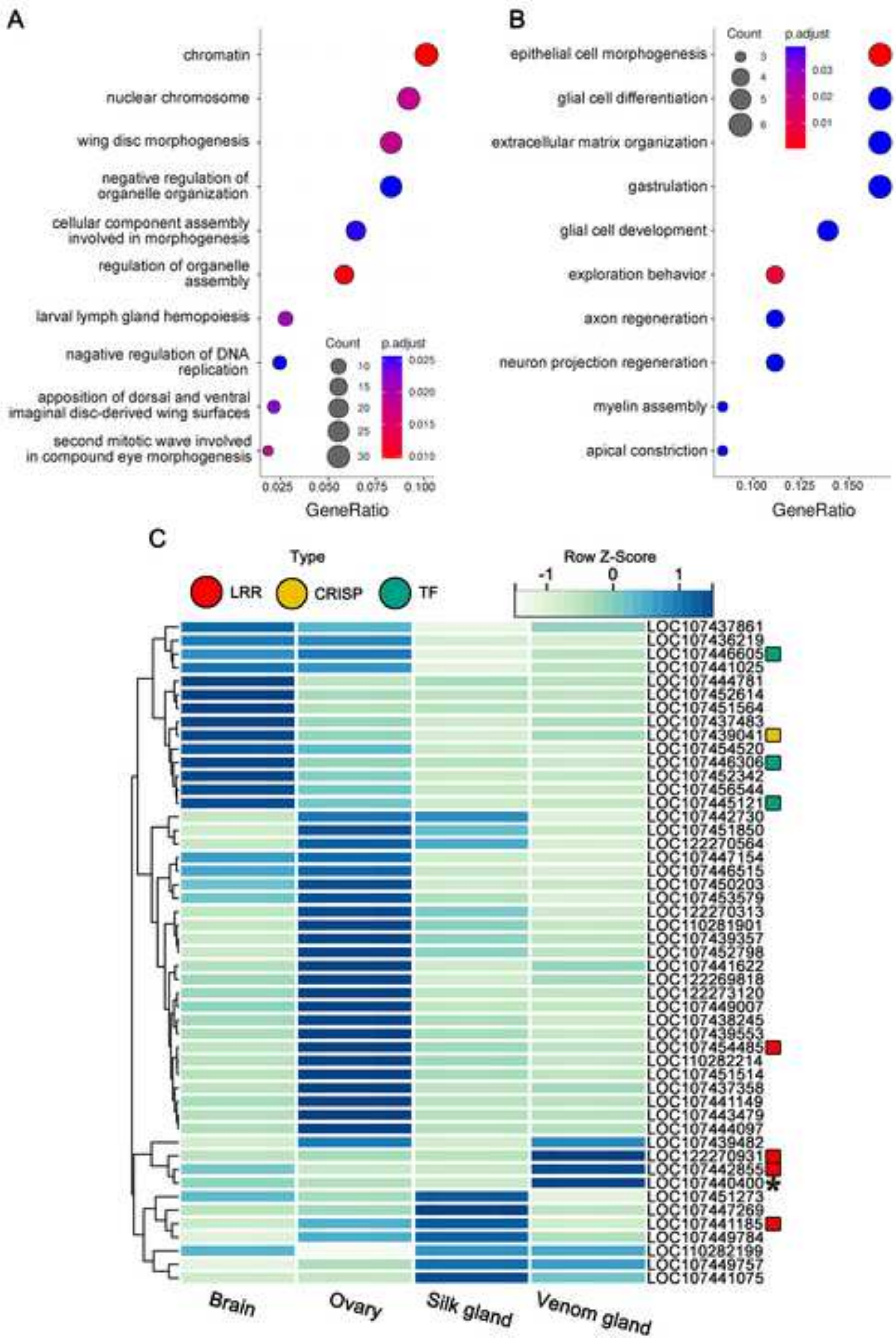

Figure 6

Click here to access/download;Figure;Fig.6-giga-re.jpg

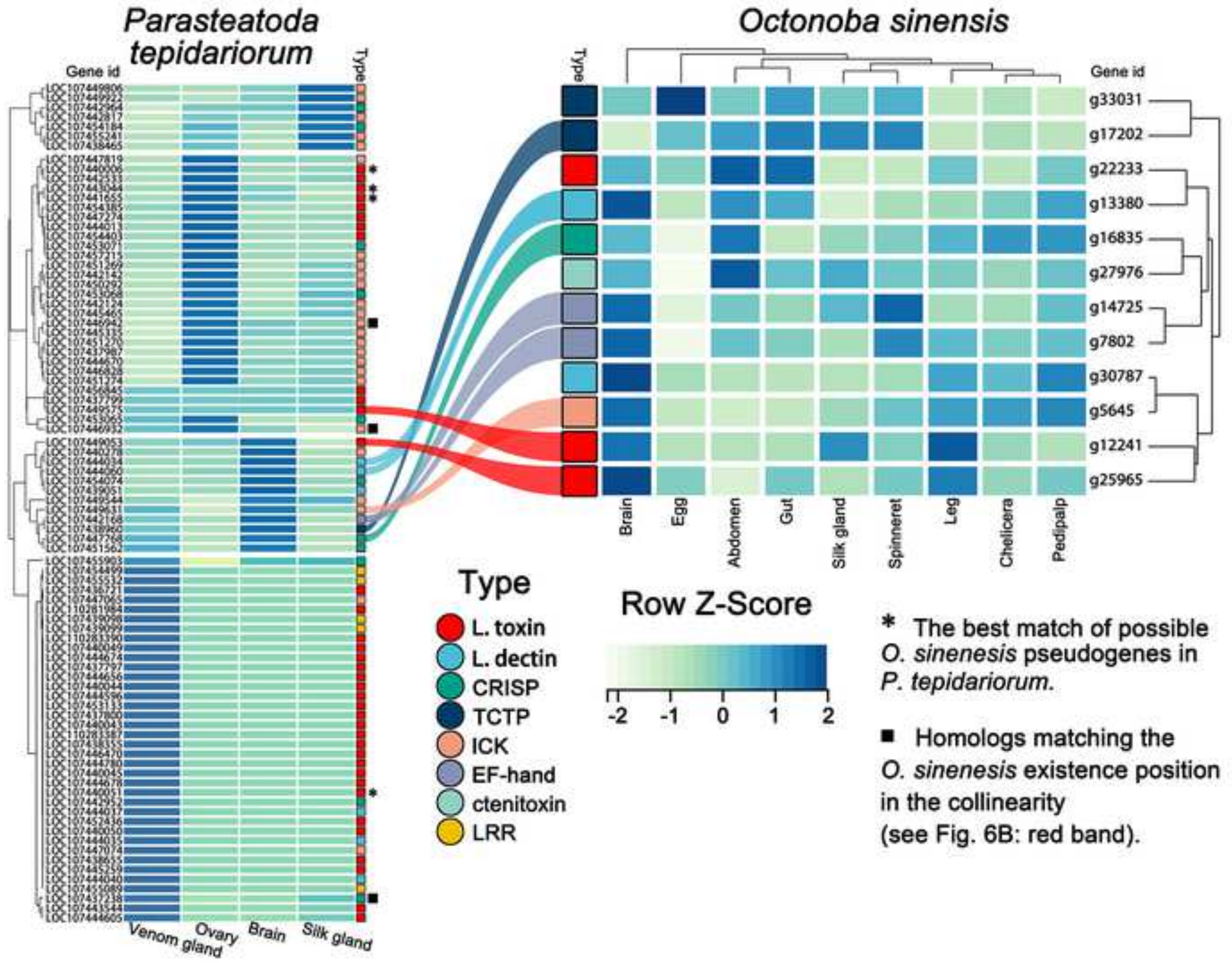

Figure 7

Click here to access/download;Figure;Fig.7-giga-re.jpg

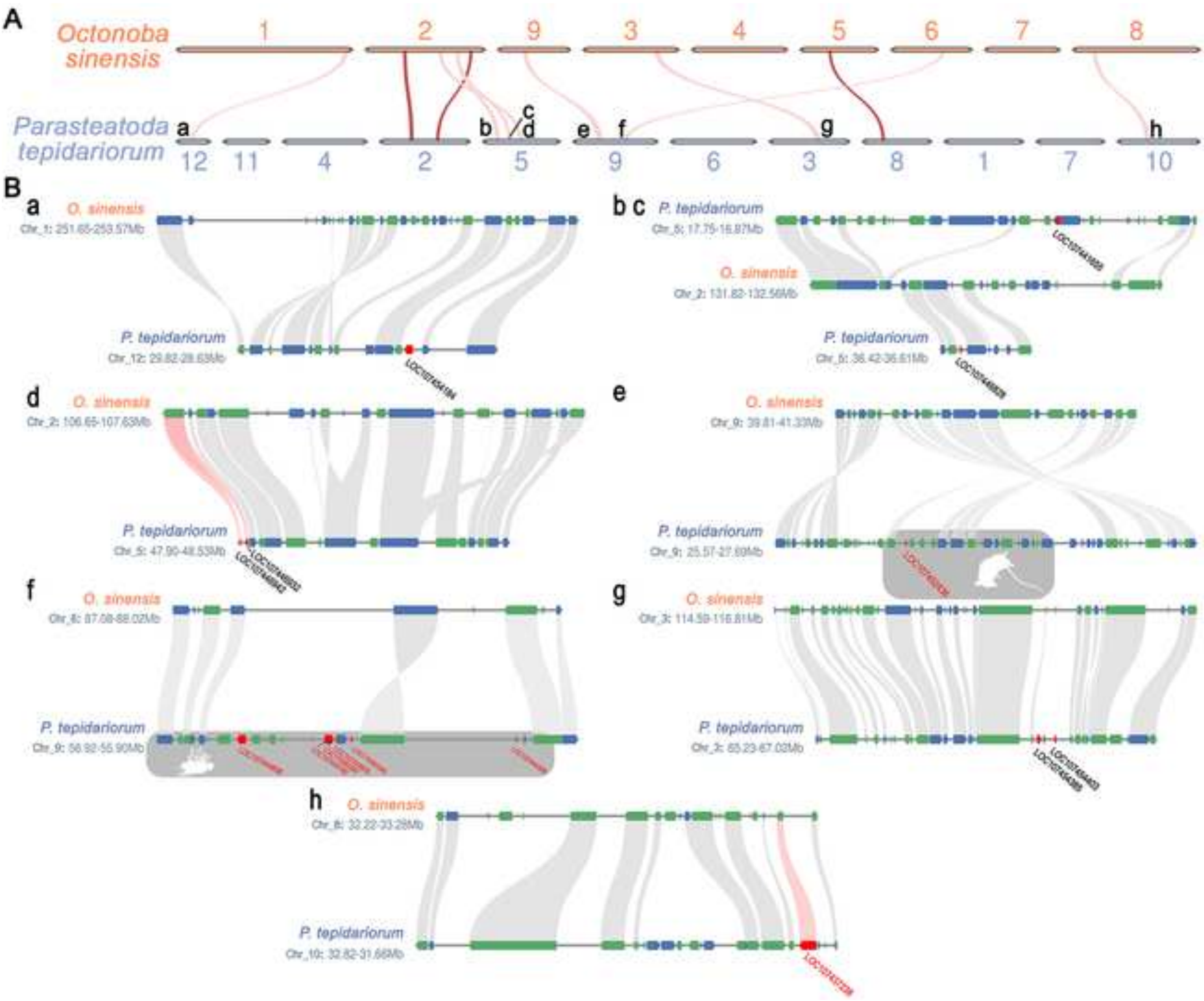

Additional file 1

Click here to access/download
Supplementary Material
Additional file 1.mp4

Additional file 2

Click here to access/download
Supplementary Material
Additional file 2.mp4

Additional file 3

Click here to access/download
Supplementary Material
Additional file 3.mp4

Additional file 4

Click here to access/download
Supplementary Material
Additional file 4-giga.xlsx

Additional file 5

Click here to access/download
Supplementary Material
Additional file 5-giga.docx

Additional file 6

Click here to access/download
Supplementary Material
Additional file 6.fa

Click here to access/download;Personal Cover;Response to reviewers.docx

Response to reviewers

Dear Zhang

Thanks again for the careful handling of our manuscript (GIGA-D-23-00275) submitted to GigaScience. Your profound and constructive feedback, along with the insightful comments from the Dr. Nadia Ayoub and Dr. Sandra Correa-Garhwal, has been invaluable in enhancing the quality of our work.

We have diligently reviewed the comments and suggestions made by the reviewers and have made every effort to incorporate the necessary changes. All modifications made to the manuscript are clearly highlighted, allowing for a comparison with the previous version. We believe that we have addressed all the concerns raised by the reviewers.

Enclosed is a point-by-point response to the reviewers' comments, detailing the changes made and explaining how each suggestion has been incorporated into the revised manuscript. We hope that this comprehensive response, coupled with the revised manuscript, will meet your expectations and further strengthen the scientific impact of our work.

Once again, we appreciate your dedication and commitment to ensuring the highest quality of publications in GigaScience. We look forward to your further guidance and feedback on our revised manuscript.

Thank you.

Best regards,

Yiming Zhang; Shuqiang Li

Institute of Zoology, Chinese Academy of Sciences

1. Beichen West Road, Chaoyang District

Beijing 100101, P. R. China

Tel: +86-13363636950

Fax: +86-10-64807216

Email: zhangyiming@ioz.ac.cn; lisq@ioz.ac.cn

Reviewer #1:

The revised manuscript addresses most of my initial concerns and is much better overall! I'm not totally convinced of a trade-off between venom and web production (or do y'all mean the tradeoff is with excessive physical predation???). Perhaps soften this language? Clarify in discussion. Some additional clarifications are needed prior to publication.

>>>**Response:** We are once again deeply grateful for your insightful comments and suggestions, which have been instrumental in further enhancing the quality of our manuscript.

The "evolutionary trade-off between the loss of venom glands and the enhancement of physical attack strategies" is exactly what we want to express. To avoid ambiguity in the expression, we have removed the third paragraph in the **Discussion**, which is "Although the web-building behavior…. this seems to be a trade-off".

In the revised **Introduction**, we presented the chemical attack properties of venom and emphasized the physical attack properties of "prey-wrapping". We also discussed the genomic changes related to muscle, aerobic respiration and metabolism of energy substances, in the **Discussion**. Please refer to **Introduction lines 52–60** and **Discussion lines 324–362** of the revised manuscript for specific modifications.

1. The gene expression analyses are never explained - how was expression level determined - e.g. read mapping using what? What program(s) with what parameters used to compare tissues within species? What constitutes a differentially expressed gene? What methods were used to compare expression patterns between species?

>>>**Response:** Thanks for your comments. We have added this section in the **Methods** (**see lines 432–439, Methods: 5. RNA extraction, sequencing and expression analysis**), the text is taken as follows:

"Clean reads were aligned to the genome using Hisat2 [60], followed by the quantification of all samples with HTSeq [61] to determine the count value. Subsequently, TPMs were derived through automated scripts."

The "**automation scripts**" mentioned in the above modifications have been uploaded to

the FTP server provided by GigaDB.

"In the differential expression analysis across two species, only Reciprocal Best Hits (RBH) genes were extracted for quantification. To facilitate comparison, ortholog (RBH) gene IDs of *O. sinensis* were replaced by *P. tepidariorum* gene IDs for comparison and figure illustration. Finally, differential expression analysis was conducted by R package limma [62]."

For the threshold for **identifying differentially expressed genes**, we have added relevant descriptions in the caption of **Figure 3 (lines 176–177)**, as recorded below:

"(only Reciprocal Best Hits genes were considered, Fold-change > 1.5, $p < 0.05$)"

In addition, we also used stricter screening conditions (Fold-change > 4, $p < 0.05$) to search for differentially expressed genes, and we created **Figure S2** in **Additional file 5** using the relevant results, which is similar to **Figure 3C**.

2. In Figure 3B, is expression analysis for both P. tepidariorum and O. sinensis? Is it comparison of the two? For Figure 3C, is the GO enrichment for genes significantly overexpressed in leg tissues of O. sinensis relative to some other O. sinensis tissue, or relative to P. tepidariorum legs?

>>>**Response:** Thanks for your comments. Yes, the differential expression analysis in **Figure 3B** is a comparison between the legs of two species. The revised description in the manuscript is copied as follows:

We have added relevant descriptions in the **Figure 3** legends **(lines 175–179**)

"(B) Differential expression analysis of homologous genes in the legs of *Parasteatoda tepidariorum* and *Octonoba sinensis* (only Reciprocal Best Hits genes were considered, Fold-change > 1.5, $p < 0.05$). Genes exhibiting higher expression levels in *O. sinensis* legs are designated with red upper triangles, whereas those with higher expression in *P. tepidariorum* legs are designated with green lower triangles."

For **Figure 3C**, relevant descriptions were added:

Results, 4. Genes under positive selection and energy metabolism in muscle (lines 160–

162)

“Compared to the legs of *P. tepidariorum*, genes with higher expression in the legs of *O. sinensis* were most enriched in the mitochondrial matrix, meanwhile significant enrichment was also observed in other GO terms pertaining to mitochondria (Figure 3C, Figure S2).”

we have also added relevant content to the caption, the text is taken as follows (**lines 179–181**):

“(C) GO terms enrichment analysis of genes expressed at higher levels (Fold-change > 1.5, $p < 0.05$) in *O. sinensis* legs relative to those in *P. tepidariorum* legs. GO terms related to the mitochondrion are highlighted in red.”

3. Is it possible to access all these new annotations? Would be great to point to their public location.

>>>**Response:** Thanks for your suggestion, the relevant data has been uploaded to the FTP server provided by GigaDB and will be made public after the article is published. I will further confirm with the administrator of the GigaDB that you can obtain this data at this stage of the process.

We have added a public location for sequencing data in SRR, which is ScienceDB (doi.org/10.57760/sciencedb.09166)

4. Why were reciprocal best blast groups used for scans of positive selection rather than the Orthofinder families?

>>>**Response:** Thanks for your comments. Both the RBH and Orthofinder were used to search for one-to-one orthologs across species, RBH method can obtain more orthologous genes (see Table S19). Therefore, we conducted a positive selection analysis using the reciprocal best blast groups.

5. Table S4 - what is the source of P.tepidariorum? D. spinosa?

>>>**Response:** Thanks for your comments. We have supplemented this information, as recorded below:

“

Species	Assembly version (source)	Source	Assembly level
Parasteatoda tepidariorum	10.11922/sciencedb.o00019.00014	Sciencedb	Chromosome
***Deinopis* sp.**		This study	Transcriptome assembly

”

6. Table S5 - there are no entries under O. sinensis. Should be, right?!

>>>**Response:** Thanks for your reminder. We have added this section.

7. Table S6 - is the lfc between P.tep and O.sin? How was this calculated? (see above). Is the p-value shown or the FDR? How were the numbers in Table S6 generated?

>>>**Response:** Thanks for your comments. Yes, this is a differential expression analysis between two species, where lfc represents log2 fold change, and the p-value was displayed in the table.

The calculation method was described in detail in the **Methods** section (**lines 432–439**). We used *P. tepidariorum* as a control, and homologous genes were also characterized by gene IDs in *P. tepidariorum*. A positive value of log2(Fold Change) indicates that the expression level of this gene in *O. sinensis* is higher than that in *P. tepidariorum*, while a negative value indicates that the expression level of this gene in *O. sinensis* is lower than that in *P. tepidariorum*.

8. Table S7 - gene families or genes?

>>>**Response:** Thank you for your reminder. We have reorganized the table according to the attribution of gene families.

9. Table S8 - expanded gene families?

>>>**Response:** Thank you, we have reorganized the table according to the attribution of gene families.

10. S16 - I don't know what this table provides.

>>>**Response:** Thanks for your comments. This table presents the alterations in the

quantity of structural domains observed in a pair of homologous genes, as detailed in the manuscript (**lines 298–300**). To enhance clarity and facilitate comprehension, we have included a descriptive header for each column.

11. Table S8 - Asianopis shows up but was never mentioned in text.

>>>**Response:** Thank you for your reminder. This is an oversight, we found it in Table 18, it should be *Deinopis* sp., we have corrected it.

12. Additional file 6 - Expression matrix - are these TPM values or something else? Is the * in this table the same as the Toxin gene expression matrix?

>>>**Response:** Thanks for your comments. The previous table was a TPM value expression matrix of genes in different *O. sinensis* tissues, and there was an error in the title, which should be "**Figure 7**". Its function is only to demonstrate that two genes in the *O. sinensis* in Figure 7B(h, linked with red ribbon) do indeed have expression levels. We have removed other genes in the new modification, leaving only the two required genes. We have renamed the table as **Table S17** and placed it in **Additional file 4**.

13. Some typos:

>>>**Response:** Thanks for your comments. We have made modifications according to the comments.

a. Line 42: delete However,

>>> **Response:** See **line 42.** We have delete "However"

b. Line 59: sinensis needs an s on the end

>>> **Response:** See **line 64.** "The spider *Octonoba sinensis* belongs to the family Uloboridae."

c. Line 87: change providing to provides

>>> **Response:** See **line 93.** "which further provides evidence that uloborids are not equipped to deliver venom (Figure 1C, D)."

d. Line 242: lower case we

>>>**Response:** See **line 253.** “In addition, we referred to the venom gland specific expression module”

e. Line 267-268: incomplete sentence

>>>**Response:** Thanks for your comments. We have reorganized the language of this entire piece of content, with the following excerpts (**lines 278–280**):

“Given that *O. sinensis* had three latrotoxin homologs out of 12 venom components (highest category, Table S14) and latrotoxins are not known outside of Theridiidae, a phylogenetic analysis of this gene family has been conducted.”

f. Line 316: Should there be a "lost" between "some of these" and "genes may be"?

>>>**Response:** Thanks for your comments (f–g). Based on the feedback of you and another reviewer, this section has been removed. Please refer to the first reply provided to you for details (**Discussion lines 324–357**).

g. Line 320: are the RTA clade really the youngest group of spiders?

>>>**Response:** Thanks for your comments (f–g). Based on the feedback of you and another reviewer, this section has been removed. Please refer to the first reply provided to you for details (**Discussion lines 324–357**).

Reviewer #2:

Zhang et al provide a multi-omics analyses on Octonoba sinensis (Uloboridae) and describe the genetics that could explain an adaptive response to the lack of venom in the group. The revised version of the manuscript is much improved with new data and new conclusions. However, I still have some concerns that need to be addressed before its publication.

>>>**Response:** We are once again deeply grateful for your insightful comments and suggestions, which have been instrumental in further enhancing the quality of our manuscript. Below, you will find our comprehensive response to each of your comments, addressing them point by point.

1. While the addition of Deinopis transcriptome to help with the gene family analyses is fine, this information needs to be included. For example, there is no mention in Figure 1 what the red dotted line means that leads to Deinopis and why it does not have a circle diagram. Table S4 does not have the NCBI info and Table S18 is labeled as Asianopis and not Deinopis. Given that the authors have added other genomes from RTA spiders I am not sure how much this transcriptome is added since it was not included in other analyses in the study. At this point, if it is not adding relevant information to the story, I would recommend removal. It almost looks like a last minute add on.

>>>**Response:** Thank you for your comments. Only one transcriptome is added. The transcriptome assembly of *Deinopis* sp. has served as an important resource of the manuscript, including the establishment of phylogenetic tree in Figure 2B, the formation of reciprocal best hit (RBH) gene sets, the analysis of selection pressure, and the quest for newly emerging as well as missing genes within the Uloboridae family. However, due to limitations in determining the copy number of homologous genes in the transcriptome assembly and the absence of contiguous genomic fragments, the transcriptome assembly of this spider was excluded from the investigation of gene family expansion and contraction, as well as the analysis of genome Highly-Conserved Elements (HCEs).

The RNA-seq data for transcriptome assembly is described in the **Methods: 6. Genome annotation (lines 457–461)**

To enhance clarity and readability, I have made several adjustments in the revised

manuscript.

Lines 144–146: (The “red dashed line” in the revised manuscript has been changed to “red branch”)

“Because *Deinopis* sp. lacks comprehensive genomic data and is limited to transcriptome information, this species was not included in the synteny analysis (red branch, Figure 2B).”

Lines 469–470:

“The latter did not include *Deinopis* sp., as the only transcriptome data cannot determine the number of gene copies.”

Furthermore, unless explicitly stated that *Deinopis* sp. data was not utilized, we have incorporated the transcriptome assembly results of *Deinopis* sp. into various other analytical frameworks.

Regarding the entry pertaining to the **Asianopis** in **Table S18 (Table S19 in the Revised version)**, it is indeed an error. The intended Asianopis should be ***Deinopis* sp.**, and we have rectified this mistake in the revised version of the manuscript.

We have supplemented this information (**Additional file 4: Table S4**). The text is taken as follows:

“

Species	Assembly version (source)	Source	Assembly level
**…**			
Parasteatoda tepidariorum	10.11922/sciencedb.o00019.00014	Sciencedb	Chromosome
**…**			
***Deinopis* sp.**		This study	Transcriptome assembly

”

2. Figure 2C seems to be out of order to me. While reading it was hard to understand why it was there (other than being a comparative genome analysis) and not in Figure 5.

>>>**Response:** Thank you for your comments, this section (C) of **Figure 2** has been moved to the **Additional file 5: Figure S3**.

3. Lines 154-155: expression analyses are not included in the material and methods. This is very important because (1) the reader would need to understand how the expression data was generated: reads mapped to genome? To transcriptome? Using what program? (2) what program and type of normalization was done for the DE analyses? The way these two sentences are written and figure 3 legend, they imply that the authors did DE using P. tepidariorum legs as the control for the legs of O. sinensis. I have never seen a DE across species that way to be able to create a volcano plot as shown on figure 3B. How it was done needs to be included.

>>>**Response:** Thank you for your comments, we have added this section in the **Methods** (**see lines 432–438, Methods: 5. RNA extraction, sequencing and expression analysis**), the text is taken as follows:

"Clean reads were aligned to the genome using Hisat2 [60], followed by the quantification of all samples with HTSeq [61] to determine the count value. Subsequently, TPMs were derived through automated scripts."

The "automation scripts" mentioned in the above modifications have been uploaded to the FTP server provided by GigaDB.

"In the differential expression analysis across two species, only Reciprocal Best Hits (RBH) genes were extracted for quantification. To facilitate comparison, ortholog (RBH) gene IDs of O. sinensis were replaced by P. tepidariorum gene IDs for comparison and figure illustration. Finally, differential expression analysis was conducted by R package limma [62]."

The new RNA-seq data of *P. tepidariorum* are deposited into ScienceDB (doi.org/10.57760/sciencedb.09166)

For the threshold for **identifying differentially expressed genes**, we have added relevant descriptions in the caption of **Figure 3 (lines 176–177)**, as recorded below:

"(only Reciprocal Best Hits genes were considered, Fold-change > 1.5, $p < 0.05$)"

In addition, we also used stricter screening conditions (Fold-change > 4, $p < 0.05$) to search for differentially expressed genes, and we created **Figure S2** using the relevant results, which is similar to **Figure 3C**.

4. One major issue I found was the conclusion. From lines 309 to 324 I feel the argument being made is not completely logical and I can't see how it contributes to the manuscript. The discussion should be about the results and conclusions generated in this study and not on whether venom evolved before or after prey-catching webs. Here the authors have a chance to talk about the metabolic related genes that are under positive selection and how it could relate to an increase endurance in O. sinensis. What are the potential functions of the expanded or new emergent gene families? How all of that relates to no having venom glands and using this extensive prey-wrapping technique. All of that is not included in the conclusion as it stands. This section needs to be rewritten.

>>>**Response:** Thanks for your comments. Based on comments, we have adjusted this section of **Discussion** and removed discussions about "whether venom evolved before or after prey-catching webs" **(The second paragraph in the original Discussion)**

In the revised **Discussion**, we discussed the genomic features related to excessive physical predation and venom glands loss in detail, and talk about how it could relate to an increase endurance in *O. sinensis*. Please refer to **lines 324–355** of the revised manuscript for specific modifications.

5. Line 49: delete "changes"

>>>**Response:** Thank you for your suggestions, we have made the corresponding modifications (**see lines 47–50**).

6. Line 51: instead of "these spiders" use uloborids

>>>**Response:** Thank you for your reminder. We have made the corresponding modifications (**see line 51**).

"Due to the absence of venom glands, the predation methods of uloborids are also relatively specialized."

7. Line 52: instead of "this group" use Uloboridae

>>>**Response:** Thank you for your suggestions, we have adjusted the language of this section to make it more relevant to the title of our article. This section comes from the discussion in the original version. (**see lines 52–60**).

“Generally speaking, using venom to paralyze prey is an effective chemical attack strategy. However, many kinds of spiders integrate both chemical attack and physical attack strategies. Certain species within the Araneoidea family utilize entanglement initially to restrain larger prey before a venomous final strike [6]. Although this predatory tactic may alleviate the selective pressure associated with venom usage, entanglement appears rudimentary in comparison to the prey-wrapping behavior exhibited by uloborids. In Uloboridae, this physical attack as the sole means of attack can span from a few minutes to nearly an hour, with the spider silk utilized sometimes exceeding a hundred meters in length [7–9]. Therefore, considerable physical endurance is indispensable for the successful execution of this predatory tactic.”

8. L55: add a reference to the first sentence

>>>**Response:** Thank you for your comments, we have made the corresponding modifications (**see lines 60–62**).

“Previous anatomical records indicate that spiders in the family Uloboridae have well-developed trachea [10], and many branches of the trachea extend into the prosoma and appendages [11].”

9. Line 75: delete "in its anatomy"

>>>**Response:** Thank you for your suggestions, we have made the corresponding modifications (**see lines 80–81**).

10. Line78: Suggest changing the title of the section to something like: Prey-wrapping behavior observations and fand morphology

>>>**Response:** Thank you for your suggestions, we have made the corresponding modifications (**see line 84**).

“**1. Prey-wrapping behavior observations and fang morphology**”

11. Line82: Araneoidea comes out of nowhere. Maybe say "time for other spider species (Arnaeoidea, 9.7+- …"

>>>**Response:** Thank you for your comments, we have made the corresponding modifications (**see lines 88–89**).

"This time is much higher than the previously recorded wrapping time of other spider species (Araneoidea, 9.7 ± 3.0 seconds for small prey, 26 ± 42 seconds for big prey) [6]."

12. Line 87: provides instead of providing. And "uloborids"

>>>**Response:** Thank you for your comments, we have made the corresponding modifications (**see lines 92–93**).

"which further provides evidence that uloborids are not equipped to deliver venom (Figure 1C, D)."

13. Line 90: add "s" to observation

>>>**Response:** Thank you for your comments. We have made the corresponding modifications (**see line 96**).

"Figure 1: Observations of *Octonoba sinensis*."

14. Line 91: delete channel (D)

>>>**Response:** Thank you for your suggestions, we have made the corresponding modifications (**see line 97**).

15. Line 92 indicates instead of indicating. Delete "channel"

>>>**Response:** Thank you for your reminder, we have made the corresponding modifications (**see lines 97–98**).

16. Line 99: spell out 9

>>>**Response:** Thank you for your reminder, we have made the corresponding modifications (**see lines 104–105**).

"A total of 20 scaffolds were obtained, of which more than 99.9% of the sequences were

loaded onto nine scaffolds that reached the chromosome level (Figure 2A),"

17. L126: maybe add that the proportions circles are next to each species

>>>**Response:** Thank you for your reminder. We have reorganized the language of this entire piece of content, the text is taken as follows (**lines 131–132**):

"The collinearity between *O. sinensis* and other spider species is visualized through circle diagrams positioned adjacent to each respective species."

18. Line 156: add "with" after genes and delete "the" after expression

>>>**Response:** Thank you for your comments. Based on the feedback from you and another reviewer, this section has been rewritten (**lines 160–162**).

"Compared to the legs of *P. tepidariorum*, genes with higher expression in the legs of *O. sinensis* were predominantly enriched in the mitochondrial matrix, while significant enrichment was also observed in other GO terms pertaining to mitochondria (Figure 3C, Figure S2)."

19. Line 171 add "term" after GO

>>>**Response:** Thank you for your comments, we have made the corresponding modifications (**see line 179**).

"(C) GO terms enrichment analysis of genes expressed at higher levels"

20. Line 172: delete (D-E).

>>>**Response:** Thank you for your reminder, we have made the corresponding modifications (**see lines 180–181**).

21. Section 5 should include how the authors identified the expanded and new emergent gene families. Just one or two sentences.

>>>**Response:** Thank you for your reminder. We have made the corresponding modifications (**see lines 188–189)**.

"To compare the genomic differences between Uloboridae and other spiders, we used CAFE v4.2 [24] to analyze the gene family expansions and contractions."

(lines 205–207)

“Based on the results of orthologous gene identification, we screened for gene families that are exclusively shared in Uloboridae and not found in any other spider species (species in Figure 2B), designating them as new emergent gene families.”

22. Section 5 Lines 181-182: could rephrase these sentences to be clearer. For example: In O. sinensis, we found four of these families to have a high number of annotated genes that corresponded to: …

>>>**Response:** Thank you for your reminder. We have made the corresponding modifications (**see lines 190–191)**.

“In *O. sinensis*, we found four of these families to have a high number of annotated genes that corresponded to:”

23. Figure 4 shows with very low quality on my copy.

>>>**Response:** Thank you for your comments. We have thoroughly reexamined the image, confirming that the uploaded version possesses a size of 738 KB and a resolution of 2008 × 1279 pixels.

24. In general, when a number has more than 3 digits a comma should be added. For example, Line 243 - instead of 1088 write 1,088. There are too many instances to numbered them all. Please check the document.

>>>**Response:** Thank you for your comments. We have carefully reviewed the entire text and corrected all such issues.

25. Line 208: add Species before phylogeny

>>>**Response:** Thank you for your comments. We have made the corresponding modifications (**see line 219)**.

“(A) Species phylogeny for calculating the expansion and contraction of gene families.”

26. Line234: spell out 5

>>>**Response:** Thank you for your comments. We have made the corresponding

modifications (**see line 245)**.

"we found that five genes belong to two toxin related gene families"

27. Line 245 delete "gene" after ")"

>>>**Response:** Thank you for your comments. We have made the corresponding modifications (**see lines 255–256)**.

28. Figure 5 and 6. The expression plots do not indicate the type of values. Are this TPMs? RPKM? Raw reads?

>>>**Response:** Thank you for your comments. We have supplemented this information. The text is taken as follows:

(Figure 5 lines 262–266)

"The expression patterns of the genes specifically absent in Uloboridae were examined in the model species *Parasteatoda tepidariorum*. The heatmap is plotted based on the Z-score transformed from Transcripts Per Million (TPM) values. Different colored markers are used to distinguish between distinct gene types, while an asterisk identifies the gene that is notably absent from the venom gland-specific expression module of *P. tepidariorum*."

(Figure 6 lines 308–309)

"The heatmap is plotted based on the Z-score transformed from Transcripts Per Million (TPM) values."

29. Figure 5A legend. The GO enrichment is for which species? Ptep? O sin?

>>>**Response:** Thank you for your comments. *O. sinensis*. We have supplemented this information. The text is taken as follows:

(lines 260–261)

"(A) GO enrichment of genes under relaxed selection in *Octonoba sinensis.*"

30. Line 258 add "in O. sinensis, we .."

>>>**Response:** Thank you for your comments. We have made the corresponding

modifications (**see lines 269–271)**.

"To search for toxin genes in *O. sinensis*, we integrated the results of previous studies and established a comprehensive toxin protein database (Additional file 6) and screened toxin gene homologs with the same threshold in different species."

31. Line 260: delete have

>>>**Response:** Thank you for your comments. We have made the corresponding modifications (**see lines 271–272)**.

32. Line 265: add " in the venom glands of P. tepidariorum"

>>>**Response:** Thank you for your comments. We have made the corresponding modifications **(see lines 276–277)**.

"Nevertheless, through the characterization of expression patterns for these *O. sinensis* homologs in the venom glands of *P. tepidariorum*,"

33. Lines 267-270: these sentences are out of order and hard to follow. It is not clear why the authors are talking about latrotoxin or why an additional gene tree was generated for this venom component and not the other ones. I suggest the authors start with something along the lines of "Given that octonoba had four latrotoxin homologs out of 12 venom components (highest category, Table S14) and latrotoxins are not known outside of Theridida

>>>**Response:** Thank you for your comments. We have reorganized the language of this entire piece of content, the text is taken as follows:

(line 276–280)

"Nevertheless, through the characterization of expression patterns for these *O. sinensis* homologs in the venom glands of *P. tepidariorum*, we discovered that the toxin-related homologs of *O. sinensis* do not exhibit high expression levels in venom glands (Figure 6). Given that *O. sinensis* had three latrotoxin homologs out of 12 toxin homologs (highest category, Table S14) and latrotoxins are not known outside of Theridiidae, the phylogenetic analysis of this gene family has been deployed."
