# Supplementary material for: A trade-off in evolution: the adaptive landscape of spiders without venom glands
Source: Gigascience. 2024 Aug 5;13:giae048. doi: 10.1093/gigascience/giae048 (PMC11299198; doi:10.1093/gigascience/giae048)
Supplement: giae048_Supplemental_Files [file giae048_supplemental_files.zip › Additional file 5-giga.docx]

**
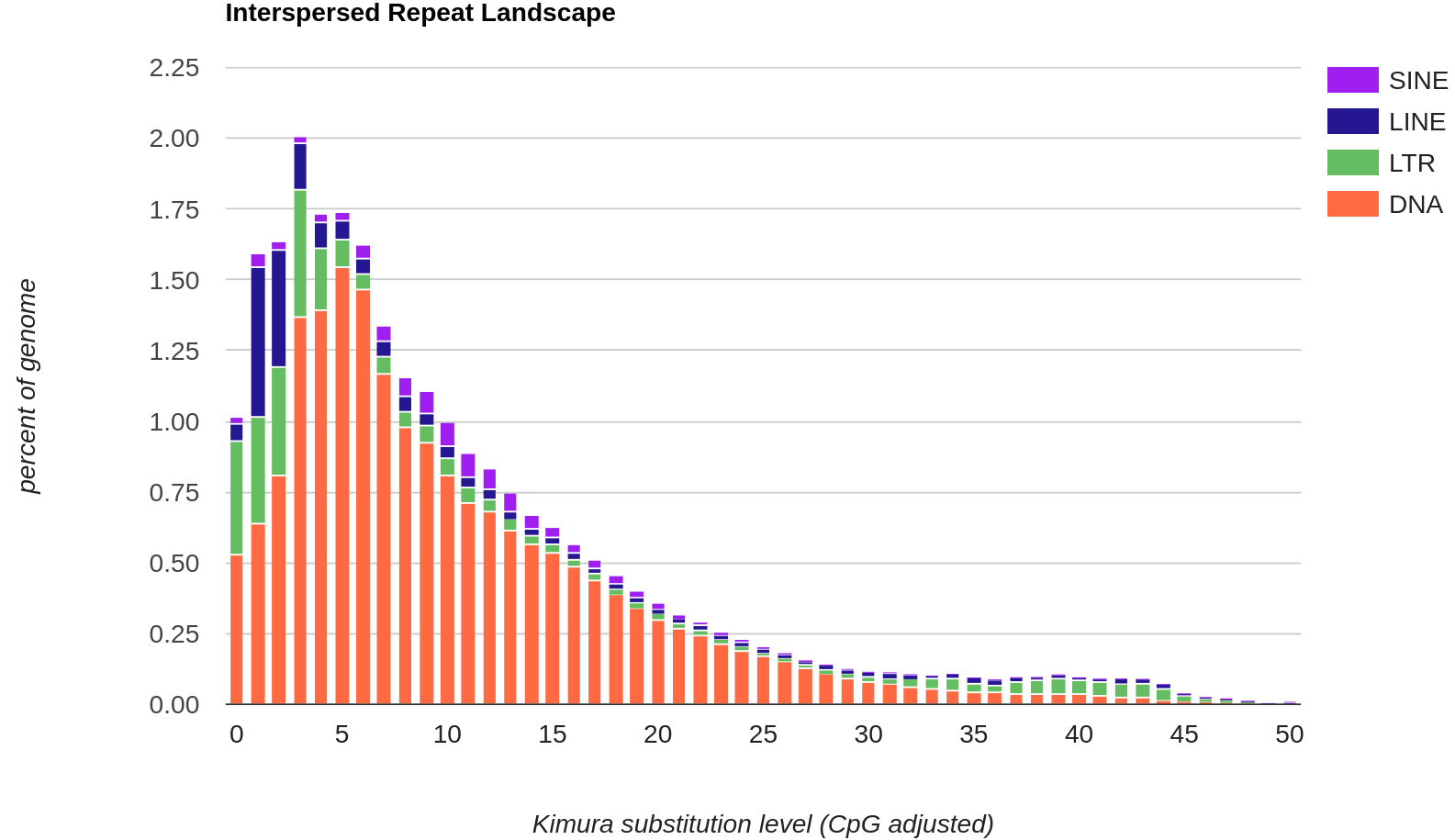
**

**Figure S1:** Recognizable elements of the *Octonoba sinensis* genome.


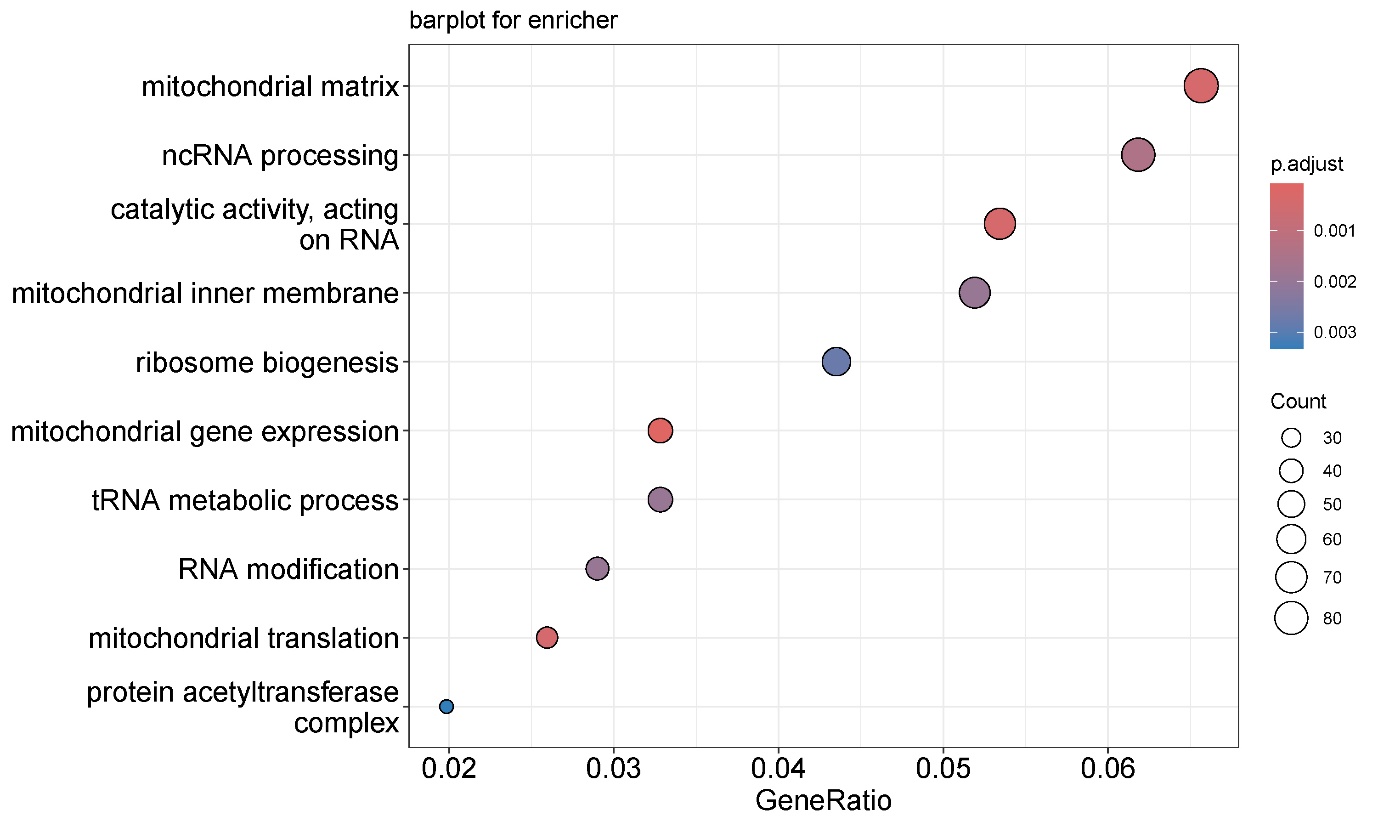


**Figure S2:** GO enrichment analysis of genes expressed at higher levels (Fold-change > 4, p < 0.05) in *O. sinensis* legs relative to those in *P. tepidariorum* legs.

**
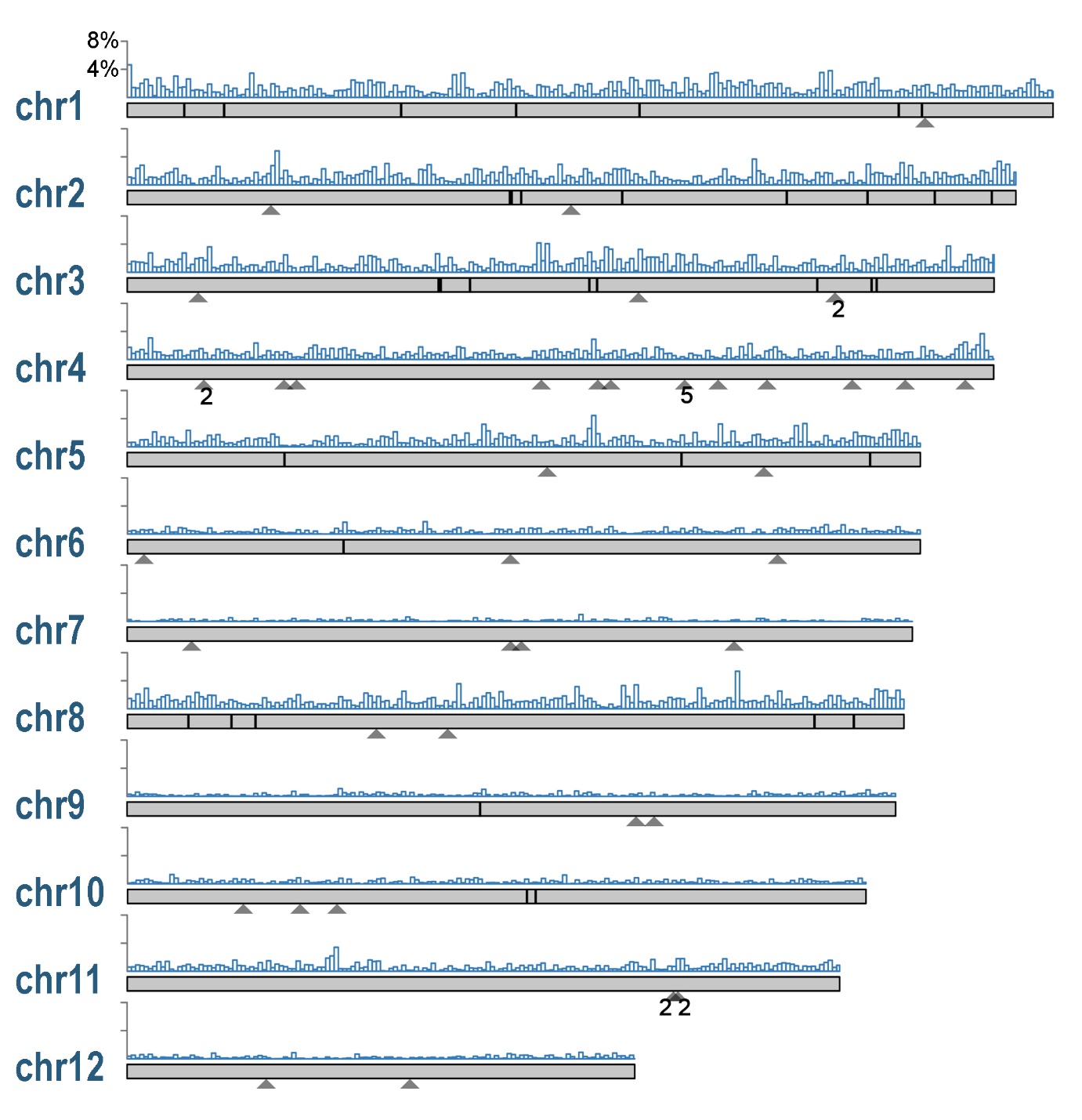
**

**Figure S3:** Distribution of all Highly-Conserved Elements (HCEs) in *P. tepidariorum* and missing HCEs and genes in *O. sinensis*. The blue bar chart represents the distribution of HCEs, decimals on the ruler represent the proportion of HCEs; the black band represents HCEs with specific deletions in *O. sinensis*; the gray arrow indicates the location of genes with specific deletions in *O. sinensis*. If the number of missing genes at the location is greater than 1, it is indicated with a number.


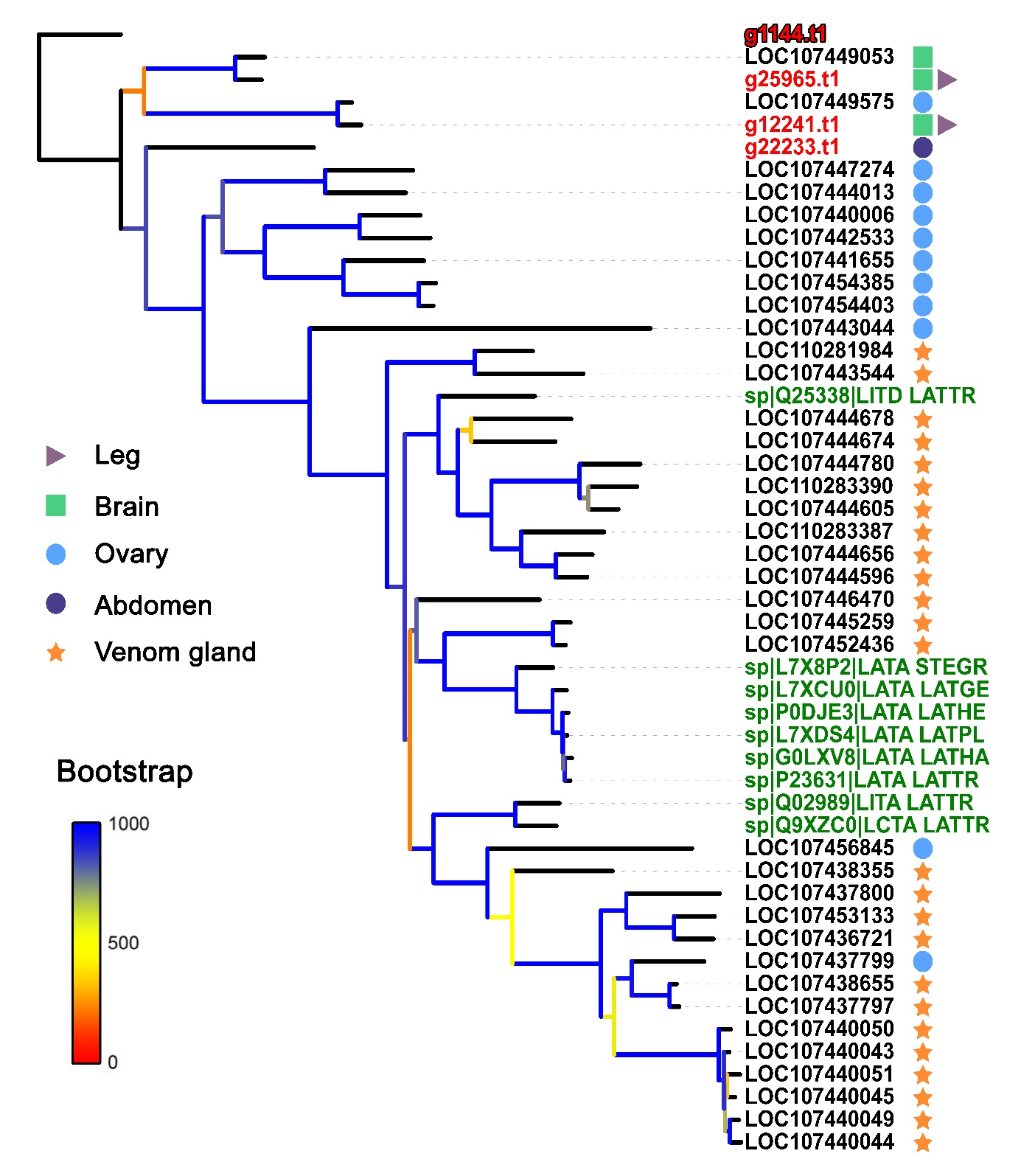


**Figure S4:** Phylogenetic tree of neurotoxin genes (Latrotoxin). Tips labelled in red font represent *Octonoba sinensis* genes, those labelled in green font represent the reference sequences of different Latrotoxins genes previously studied, others represent *Parasteatoda tepidariorum* genes. Tips labelled in green font come from different species, *Latrodectus tredecimguttatus*: sp|Q25338|LITD_LATTR, sp|P23631|LATA_LATTR, sp|Q02989|LITA_LATTR, sp|Q9XZC0|LCTA_LATTR; *Steatoda grossa*: sp|L7X8P2|LATA_STEGR; *Latrodectus geometricus*: sp|L7XCU0|LATA_LATGE; *Latrodectus hesperus*: sp|P0DJE3|LATA_LATHE; *Latrodectus pallidus*: sp|L7XDS4|LATA_LATPL; *Latrodectus hasselti*: sp|G0LXV8|LATA_LATHA. The red font with black stroke is a similar object below the recognition threshold as an outlier.
