# Supplementary material for: A trade-off in evolution: the adaptive landscape of spiders without venom glands
Source: Gigascience. 2024 Aug 5;13:giae048. doi: 10.1093/gigascience/giae048 (PMC11299198; doi:10.1093/gigascience/giae048)
Supplement: giae048_Response_to_Reviewer_Comments_Original_Submission [file giae048_response_to_reviewer_comments_original_submission.pdf]

Author's Response To Reviewer Comments

Response to reviewers
Dear Zhang
Thanks very much for handling our manuscript (GIGA-D-23-00275) submitted to GigaScience. We greatly appreciate the profound and constructive comments from you and the two reviewers, which are very helpful to improve our manuscript. We have been able to incorporate changes to reflect the suggestions provided by the reviewers. all he changes are highlighted within the manuscript. We believed that we have addressed all the concerns from the reviewers. A point-by-point response to reviewers' comments is enclosed.

Sincerely,
Yiming Zhang; Shuqiang Li
Institute of Zoology, Chinese Academy of Sciences
1. Beichen West Road, Chaoyang District
Beijing 100101, P. R. China
Tel: +86-10-64807216
Fax: +86-10-64807216
Email: zhangyiming@ioz.ac.cn; lisq@ioz.ac.cn

Editor comments
As the reviewer mentioned that the genome of Uloborus diversus should be included in the manuscript.
>>>Response: Thanks for your suggestions. We added this genome to the new analysis and supplemented the annotation, increasing the number of coding genes with transcriptome support from 15750 to 19036. Subsequently, we also added some genomes proposed by reviewers to the new analysis and conducted all downstream analyses based on these data.

Reviewer #1:
Zhang et al. provide a chromosome level assembly of Octonoba sinensis, a member of the one and only family of spiders that does not possess venom glands. The sequence and annotation is highly valuable for arachnologists and arthropod biologists more generally. The manuscript also provides some interesting analyses, focusing on potential gene loss associated with loss of venom glands. However, I have concerns that need to be addressed prior to publication.
>>>Response: Thank you for your positive assessment of our work. In this revision, we have strengthened the exploration of the trade-offs between motor function and the absence of venom glands. In addition to some highlights in the original manuscript, new discoveries have also emerged under a new comparative system. These results provide a more comprehensive characterization of the adaptive evolution of this group under the absence of venom glands.
1. First, the closest relative included in the gene family analyses, Stegodyphus dumicola (Eresidae), is quite distantly related from Uloboridae. Thus, inferences about gene loss or positive selection associated with the loss of venom glands are quite difficult to make with confidence. At a minimum, the authors need to clarify the lack of inclusion of any other members of the UDOH grade. As written (lines 180-183), the authors insinuate that these close relatives are included in the analysis of gene loss and positive/relaxed selection. This is not true. Although, there are no genomes currently available for members of Deinopidae, Oecobiidae, Hersiliidae, there are transcriptomes. The authors could consider an additional analysis using these transcriptomes to track gene loss.
>>>Response: Thanks for your suggestions. Based on your suggestion, we assembled the transcriptome of an Ogre-faced spider (Deinopis sp.), and obtained a total protein Busco score of 93.1% (see Fig. 2B, 3A and Methods: 6. Genome annotation). We used this portion of data to enrich selection pressure analysis and assist in searching for specific gene deletions and emergence.
2. Related to the above comment, a high quality chromosome-level assembly is available for Uloborus diversus. I strongly recommend including Uloborus in synteny and gene family analyses. As another

uloborid species, its inclusion would bolster inferences about gene loss in Uloboridae or positive selection/relaxed selection in Uloboridae, and not just in Octonoba.
>>>Response: Thanks for your comments. We added this genome to the new analysis (see Fig. 2B, 3A) and supplemented the annotation, increasing the number of coding genes with transcriptome support from 15750 to 19036. Subsequently, we also added some genomes proposed by reviewers to the new analysis and conducted all downstream analyses based on these data.
3. Related to above, there are three genomes available for RTA clade species in NCBI. It doesn't look like there are associated publications yet, but the authors might consider inclusion to break up the evolutionary distance between Uloboridae and other species currently in the analysis; if the authors are set on only using species with whole genomes.
>>>Response: Based on your suggestion, we have annotated the Pardosa pseudoannulata and Dolomedes plantarius genomes, and the total protein Busco scores reached 93.6% and 95.0%, respectively. In addition, we replaced the Dysdera silvatica genome with the newly published Ectatosticta davidi genome as the basal group, and re-annotated the Latrodectus elegans genome, resulting in an increase in its protein Busco score from 63.7% to 96.5%. In the end, all of these genomes have a protein Busco score of over 90% (Additional file 4: Table S2), and a more suitable evolutionary tree was obtained (see Fig. 2B, 3A).
4. For positive selection analyses, is finding metabolic genes unusual? E.g. if the same analysis was done moving a species with venom glands to the target would metabolic genes still arise? Perhaps a different set of genes, but could be something more general to arachnids/arthropods?
>>>Response: Based on your suggestion, using the new dataset mentioned above, we conducted a new positive selection analysis using the nodes of the Uloboridae family, where the O. sinensis and U. diversus genomes are located, as the foreground branch. Unfortunately, the new significant positive selection gene set does not support enrichment in energy metabolism pathways (see Results: 4. Genes under positive selection and energy metabolism in muscle). However, in comparative transcriptome analysis of legs of different species, genes highly expressed in the O. sinensis can be significantly enriched in mitochondrial related GO entries (Fig. 3C).
5. The relevance of the "absent" regions and regions under relaxed selection wasn't clear. Was there a specific hypothesis being tested? E.g. that toxin genes are more likely to be lost or under relaxed selection? Clarify.
>>>Response: Thanks for your comments. We speculate that due to the absence of venom glands, the genes or functional regions specifically involved in the venom gland system in the Uloboridae family may be subjected to relaxed purifying selection or gradually lost from genome. In order to obtain this information, Highly-Conserved Elements (HCEs), absent genes and genes under selective relaxation in the O. sinensis and U. diversus genome were searched and identified. We have added the relevant description to the "Results: 6. Absent regions and genes under relaxed purifying selection" to make it clearer.
6. Similarly, the point of the SNP analysis wasn't clear. Was there a driving hypothesis? What is learned from the analysis?
>>>Response: Thanks for your comments. The distribution of SNPs may reflect information from recent evolutionary processes or divergence characteristics between closely related lineage. It may not properly reflect the evolution information on a larger time scale (such as in this study) (doi: 10.1093/gbe/evz128). Therefore, we have deleted this section in the revised manuscript.
7. How were venom specific modules identified in Parasteatoda? If the authors generated their own RNA seq for Parasteatoda, please include accessions.
>>>Response: Thank you for pointing this out. This “venom specific modules” was the result of our previous work (doi: 10.1186/s12915-023-01581-7). It was obtained through weighted correlation network analysis using transcriptome data from different tissues, and the relevant results have been published. Here, we directly cite this result (see reference 29).
In the revised manuscript, we have generated transcriptome data for legs from Parasteatoda, while remaining transcriptome data of the Parasteatoda are sourced from the aforementioned references. We have provided clarification in the revised manuscript (see Methods: 5. RNA extraction and sequencing).
8. I'm not convinced latrotoxins exist outside of Theridiidae. Is it possible the significant BLAST alignments are based on ankyrin repeats found in latrotoxins and other types of genes? Please provide more evidence in support of latrotoxins in distant relatives of Latrodectus.
>>>Response: Thank you for your reminder. You have raised an important point here. The toxin gene homologs we obtained through our search were all based on the results of blast analysis using the spider toxin database annotated by previous researchers.
In the revised manuscript, we also established the hidden Markov models (HMM) for different types of toxin

proteins based on the database, and further confirmed the results obtained from blastp using HMMER v 3.3. The confirmation of any toxin protein requires strict toxicology or virulence testing. In the revised manuscript, we refer to the target gene obtained through our search as a toxin gene homolog. Based on this, we have also revised some of the less rigorous viewpoints in the discussion. Thank you again for your prompt.
9. Language and readability were mostly fine, but the manuscript could use editing in many places-too many for me to enumerate. One item that jumped out was the use of "losed" for "lost" in some figures. Please correct and have someone read the whole manuscript carefully.
>>>Response: Thank you, we will do our best to review and revise the manuscript.

Reviewer #2:
The manuscript titled "A Trade-off in Evolution: The Adaptive Landscape of Spiders without Venom Glands" described the a comprehensive study of the spider Octonoba sinensis to identify pathways related to metabolism and trachea development that could help explain energy requirements by the spider's extensive wrapping behavior. The manuscript also explores genetic footprint of venom components still present in the venom less spider. The manuscript has a lot of exciting new data, I find the metabolic and trachea genetics very interesting to specialist as well as the general public. I do however have some issues with the venom sections that in my view need to be addressed.
>>>Response: We sincerely appreciate all your valuable comments and suggestions, which helped us in improving the quality of the manuscript. Below is our point-by-point response to each comment.
1. It is worth noting that Uloboridae is not the only spider family to not have venom glands. Holarchaea is another family that has also been described as not having venom glands. This should be mentioned/addressed.
>>>Response: Thank you for your reminder. We have added relevant descriptions in the introduction section (see line 40–44). The text is taken as follows:
“However, as an important means of hunting and defense, the toxin system gives spiders an outstanding advantage in environmental suitability and has allowed them to spread throughout the world. Of course, there are always exceptions, some outliers are believed to be lacking venom glands. Currently, known spiders without venom grands include the Holarchaea genus (2 species)[2–4] and the entire family Uloboridae, with the latter being the most prosperous group of them [5].”
2. I have some issues with Section 7 of the manuscript: "Differential retention of major toxin genes in O. sinensis". Knowing that the family does not have venom glands and has not many millions of years it is not acceptable for the authors to refer to the genes they find in O. sinensis with homology to known venom components as toxin genes. This will lead to many misinterpretations although admittedly it sounds a lot catchier. Moreover, the families that were identified while all are known to be venom components, not all are toxins. For example, the Translationally Controlled Tumor Protein (TCTP) a histamine-releasing factor acts together with Together the cysteine-rich secretory protein (CRISP) family, contributing to inflammatory responses (Sade et al., 2012; Boia-ferreira et al., 2019; Justa et al., 2020). Given this, the authors will need to tone down the claim of having found toxin in a spider that does not have venom glands. This is not surprising assuming a secondarily loss in the family (the most parsimonious interpretation). I believe this also leads to some misinterpretations of the results; it is my understanding that most venom components are hypothesized to have evolved from ancestor proteins that had normal physiological functions that were then recruited. This information is not concluded from the current manuscript, there is a large body of evidence that supports the evolution of venom proteins via processes such as gene duplication followed by subfunctionalization and/or neofunctionalization, horizontal gene transfer, single gene co-option, etc. It follows that expression of venom homologs in the brain region should not come as a surprise since a lot of what are refer to as toxins are thought to be recruited from the neuropeptides, molecules such as latrodectins (McCowan & Garb 2014). I suggest a major modification of the results/discussion section having more emphasis on the metabolic data and how this can potentially be a trade-off because as written, the manuscript does not describes a trade-off between wrapping and lack of venom.
>>>Response: Thank you for your comments, and we fully accept your suggestion. The toxin gene homologs we obtained through our search were all based on the results of blast analysis using the spider toxin database annotated by previous researchers.
In the revised manuscript, we also established the hidden Markov models (HMM) for different types of toxin proteins based on the database, and further confirmed the results obtained from blastp using HMMER v 3.3. The confirmation of any toxin protein requires strict toxicology or virulence testing. In the revised

manuscript, we refer to the target gene obtained through our search as “toxin gene homolog”. Based on our analysis results, we ultimately believe that the toxin genes used for hunting in Uloboridae have been lost, and changed the Result section 7 to “Deficiency of toxin genes in O. sinensis”. We have also deleted some of the less rigorous viewpoints in the Discussion. Thank you again for your prompt.

3. The reason why P. tepidarioriom was used as a reference comparison needs to be stated somewhere. I think it is somewhat arbitrary and I personally would like to hear the authors' reasons. Also why U. diversus, another Uloboridae was not included in the study is beyond me. This species also has a chromosome level genome and could add to the story.
>>>Response: P. tepidarioriom is a globally distributed species and also a type species of spider (10.1186/s12915-017-0399-x; 10.1186/s13227-015-0011-9; 10.1093/molbev/msad239; 10.1016/j.dib.2018.05.106). It has the best protein annotation currently available in spiders and a good genome assembly at the chromosome level. In addition, it has rich online data and annotation resources in major databases such as KEGG and GProfiler. Therefore, using it as a control species can obtain a more comprehensive evolutionary landscape. Moreover, P. tepidarioriom have relatively small differences in body size, ecological niches, and hunting targets compared to O. sinensis. Therefore, considering these aspects, the two species also have comparative value. We have added relevant descriptions in the revision, and the screenshot is as follows:
Indeed, U. diversus is a very important species for the scientific issues involved in this study. In this revision, we have incorporated this important genome into our analysis system (see Fig. 2B, 3A) and supplemented the annotation, increasing the number of coding genes with transcriptome support from 15750 to 19036. Subsequently, we also added some genomes proposed by reviewers to the new analysis and conducted all downstream analyses based on these data.
4. L45-47: I am not sure I understand the meaning of these sentences and I suggest their removal.
>>>Response: We have deleted these sentences according your suggestion.
5. L51-54: There are many spider families that have well-developed trachea with branches extended into their prosoma, such as salticidae and Cybaeidae. To make the case more compelling for why it is particularly important for Uloboridae to have this adaptation I suggest the authors include more detrain about the greater need for energy in the limbs or greater metabolic input and thus there could be a greater presence of mitochondria in the legs or upregulation of genes related to energy metabolism. If this is not expanded, it is unclear why O. sinensis is indeed a good model for this study.
>>>Response: Thank you for your suggestions. Other groups may also require leg strength, but many have abandoned webs in predatory activities. Uloboridae is all web-building spiders, so we pay more attention to the compensation for the loss of venom glands in web-building spiders, and did not pay special attention to other hunting type groups. Comparative transcriptome analysis of motor organs is indeed crucial, and we have included this work in our revised manuscript. Excerpt as follows:
“In order to further explore the evolution in motor function of Uloboridae family, we compared the transcriptome data of the legs between O. sinensis and other species. We used the model species: P. tepidariorum, which is also a web-building spider, as a control. Under consistent standardized conditions, the results showed that a large number of genes were differentially expressed (Figure 3B). Compared to P. tepidariorum, genes higher expression in the O. sinensis legs were most enriched in the mitochondrial matrix, and, other GO terms related to mitochondria also detected high enrichment (Figure 4C). This result suggests that at least in the legs, O. sinensis require greater energy consumption compared to typical web-building spiders.”
6. L55: Do not start a sentence with a species name.
>>>Response: Thank you for your reminder. We have made the corresponding modifications (see line 58).
7. L76-80: Uloborid spiders have been described as lacking both venom glands and openings on the fangs for the delivery of venom (Opell 1979; Weng et al. 2006). It is not surprise that you did not observe fang openings in O. sinensis fangs. Therefore, I suggest the authors change the wording from 'prove' to further providing evidence that uloborids are not equipped to deliver venom.
>>>Response: Thank you for your suggestions, we have made the corresponding modifications (see line 83–86).
8. L 94: Not sure "as a percentage of … 98.11%" is needed.
>>>Response: Thank you for your comments, we had anticipated that someone would pay attention to this type of information, now we have moved it to the Additional file (see Results: 2. Observation of behavior and fangs).
9. L100: change to 24,579 and 24,563

>>>Response: Thank you for your suggestions, we have made the corresponding modifications (see line 104–105).
10. L101: "in at least one the following databases: "
>>>Response: Thank you for your suggestions, we have made the corresponding modifications (see line 105).
11. L250-253: I agree that it could be possible that these genes were removed from the genome but it is hard to tell from the current data if this was quick or not after selective pressures changed.
>>>Response: Thank you for your reminder. We have reorganized this section (Section 7) based on the new analysis results. The expression you mentioned here has been rewritten and taken as follows:
"Compared with other genes which it is difficult to find pseudogenes, this gene has complete gene structures and CDS regions, transcriptome data also show that it can be normally transcribed into mRNA (Additional file 6). We speculate that this gene (g31478) may plays a role as a non-toxic gene in O. sinensis."
12. L297-298: While I do not agree with the statement, at the very least the authors need to provide a lot more information to back up this claim.
>>>Response: Thank you for your reminder. This section of the discussion is indeed too radical and lacks research support. We have reorganized the language and removed the expressions about "preadaptation" and "extraction".
13. L139-140: Sentence is confusing as written and should be rewritten or deleted.
>>>Response: Thank you for your comments. In the revision, the analysis results under the new background differ from the original manuscript, and this section has been rewritten. We deliberately avoided such issues during the revision process.
14. L324: Where the samples use males or females? Mature or immature? This will be important for expression levels. Why were the spiders starved for a week?
>>>Response: Thank you for your reminder. We have added detailed descriptions of this section in the revision. Excerpt as follows:
"To minimize contamination of intestinal DNA as much as possible, all samples were starvation reared for more than one week at room temperature. Genome DNA for both short and long read sequencing were isolated from the cephalothoraxes of adult female spiders using the Qiagen Blood & Cell Culture DNA Kit (QIAGEN, Hilden, Germany)."
15. L338: what type of tissue was used to generate the genome? Whole spiders?
>>>Response: Thank you for your reminder. We have added detailed descriptions of this section in the revision (see Methods: 1. Sample collection and DNA extraction).
16. L349: experiments "were" conducted.
>>>Response: Thank you for your reminder. We checked for similar errors to the best of our ability.
17. L372: BUSCO should be spelled out, explained and cited the first time is used.
>>>Response: Thank you for your reminder. The Benchmarking Universal Single-Copy Orthologs (BUSCO) method first appeared in the Results: 2. Genome assembly and annotation, and in the revision, we spelled out its full name and cited it (see line 95, 96).

18. L375: what do you mead by spinneret? If you dissected the silk glands out (all the silk glands out) then you not have any tissue other than connective and/or fat attached the spinneret. This should be clarified. If the authors took out the larger glands and left the smaller ones behind then the names should reflect that. Are there any images of the tissues used? I will be curious to see what part of the brain and the gut was used for example. Whether the spiders where male or female, at what stage and how many where used should be included for both species.
>>>Response: Thank you for your comments. Our approach to dissecting spider spinnerets is similar to that of other appendages. It is to use tweezers to separate it from the body and remove visible internal organs as much as possible. However, the spinner was too small to be completely cleaned and may have a small portion of the silk gland.
We have not saved official images. We shoot a few videos and photos to demonstrate the anatomical process, mainly highlighting the anatomy of the intestine and brain. This information will be uploaded to the gigaDB.
19. L396: Why was the Uloboridae Uloborus diversus not included in this study? To my knowledge it is also a chromosome level assembly and would have made a stranger case for the conclusions.
>>>Response: Thank you for your comments. We have addressed this section in our response to your previous comments. Here we would like to point out that in addition to the Uloborus diversus, we also

added two species from RTA clade (Pardosa pseudoannulata and Dolomedes plantarius), replaced the Dysdera silvatica with a better quality genome of Ectatosticta davidi, and removed the less important Hylyphantes graminicola and Stegodyphus mimosarum. In the end, all of these genomes have a protein Busco score of over 90%, and a more suitable evolutionary tree was obtained (see Fig. 2B).

20. The manuscript contains both Fig and Figure. For consistency only use one.

>>>Response: Thank you for your reminder. We checked for similar errors to the best of our ability.

21. Figure 1 suggestions: B- Adult male or female? Move the scale bar to bottom right. C- I find this section to be very confusing and should perhaps be its own figure. The numbering inside the circles make it hard to understand to me. I suggest a different organization for this figure.

>>>Response: Thank you for your reminder. We reorganized the Figure 1 and placed the scale bar in channel B in the bottom right corner of that channel; We have added a separate channel. Used to describe the shooting angle in SEM photographs (see Fig. 1).
