# Supplementary material for: A trade-off in evolution: the adaptive landscape of spiders without venom glands
Source: Gigascience. 2024 Aug 5;13:giae048. doi: 10.1093/gigascience/giae048 (PMC11299198; doi:10.1093/gigascience/giae048)
Supplement: giae048_Response_to_Reviewer_Comments_Revision_1 [file giae048_response_to_reviewer_comments_revision_1.pdf]

Author's Response To Reviewer Comments

Response to reviewers
Dear Zhang

Thanks again for the careful handling of our manuscript (GIGA-D-23-00275) submitted to GigaScience. Your profound and constructive feedback, along with the insightful comments from the Dr. Nadia Ayoub and Dr. Sandra Correa-Garhwal, has been invaluable in enhancing the quality of our work.
We have diligently reviewed the comments and suggestions made by the reviewers and have made every effort to incorporate the necessary changes. All modifications made to the manuscript are clearly highlighted, allowing for a comparison with the previous version. We believe that we have addressed all the concerns raised by the reviewers.
Enclosed is a point-by-point response to the reviewers' comments, detailing the changes made and explaining how each suggestion has been incorporated into the revised manuscript. We hope that this comprehensive response, coupled with the revised manuscript, will meet your expectations and further strengthen the scientific impact of our work.
Once again, we appreciate your dedication and commitment to ensuring the highest quality of publications in GigaScience. We look forward to your further guidance and feedback on our revised manuscript.

Thank you.
Best regards,

Yiming Zhang; Shuqiang Li
Institute of Zoology, Chinese Academy of Sciences
1. Beichen West Road, Chaoyang District
Beijing 100101, P. R. China
Tel: +86-13363636950
Fax: +86-10-64807216
Email: zhangyiming@ioz.ac.cn; lisq@ioz.ac.cn

Reviewer #1:
The revised manuscript addresses most of my initial concerns and is much better overall! I'm not totally convinced of a trade-off between venom and web production (or do y'all mean the tradeoff is with excessive physical predation???). Perhaps soften this language? Clarify in discussion. Some additional clarifications are needed prior to publication.
>>>Response: We are once again deeply grateful for your insightful comments and suggestions, which have been instrumental in further enhancing the quality of our manuscript.
The "evolutionary trade-off between the loss of venom glands and the enhancement of physical attack strategies" is exactly what we want to express. To avoid ambiguity in the expression, we have removed the third paragraph in the Discussion, which is "Although the web-building behavior…. this seems to be a trade-off".
In the revised Introduction, we presented the chemical attack properties of venom and emphasized the physical attack properties of "prey-wrapping". We also discussed the genomic changes related to muscle, aerobic respiration and metabolism of energy substances, in the Discussion. Please refer to Introduction lines 52–60 and Discussion lines 324–362 of the revised manuscript for specific modifications.
1. The gene expression analyses are never explained - how was expression level determined - e.g. read mapping using what? What program(s) with what parameters used to compare tissues within species? What constitutes a differentially expressed gene? What methods were used to compare expression patterns between species?
>>>Response: Thanks for your comments. We have added this section in the Methods (see lines 432–439, Methods: 5. RNA extraction, sequencing and expression analysis), the text is taken as follows:
"Clean reads were aligned to the genome using Hisat2 [60], followed by the quantification of all samples with HTSeq [61] to determine the count value. Subsequently, TPMs were derived through automated scripts."

The “automation scripts” mentioned in the above modifications have been uploaded to the FTP server provided by GigaDB.
“In the differential expression analysis across two species, only Reciprocal Best Hits (RBH) genes were extracted for quantification. To facilitate comparison, ortholog (RBH) gene IDs of O. sinensis were replaced by P. tepidariorum gene IDs for comparison and figure illustration. Finally, differential expression analysis was conducted by R package limma [62].”
For the threshold for identifying differentially expressed genes, we have added relevant descriptions in the caption of Figure 3 (lines 176–177), as recorded below:
“(only Reciprocal Best Hits genes were considered, Fold-change > 1.5, $p < 0.05$)”
In addition, we also used stricter screening conditions (Fold-change > 4, $p < 0.05$) to search for differentially expressed genes, and we created Figure S2 in Additional file 5 using the relevant results, which is similar to Figure 3C.
2. In Figure 3B, is expression analysis for both P. tepidariorum and O. sinensis? Is it comparison of the two? For Figure 3C, is the GO enrichment for genes significantly overexpressed in leg tissues of O. sinensis relative to some other O. sinensis tissue, or relative to P. tepidariorum legs?
>>>Response: Thanks for your comments. Yes, the differential expression analysis in Figure 3B is a comparison between the legs of two species. The revised description in the manuscript is copied as follows:
We have added relevant descriptions in the Figure 3 legends (lines 175–179)
“(B) Differential expression analysis of homologous genes in the legs of Parasteatoda tepidariorum and Octonoba sinensis (only Reciprocal Best Hits genes were considered, Fold-change > 1.5, $p < 0.05$). Genes exhibiting higher expression levels in O. sinensis legs are designated with red upper triangles, whereas those with higher expression in P. tepidariorum legs are designated with green lower triangles.”
For Figure 3C, relevant descriptions were added:
Results, 4. Genes under positive selection and energy metabolism in muscle (lines 160–162)
“Compared to the legs of P. tepidariorum, genes with higher expression in the legs of O. sinensis were most enriched in the mitochondrial matrix, meanwhile significant enrichment was also observed in other GO terms pertaining to mitochondria (Figure 3C, Figure S2).”
we have also added relevant content to the caption, the text is taken as follows (lines 179–181):
“(C) GO terms enrichment analysis of genes expressed at higher levels (Fold-change > 1.5, $p < 0.05$) in O. sinensis legs relative to those in P. tepidariorum legs. GO terms related to the mitochondrion are highlighted in red.”
3. Is it possible to access all these new annotations? Would be great to point to their public location.
>>>Response: Thanks for your suggestion, the relevant data has been uploaded to the FTP server provided by GigaDB and will be made public after the article is published. I will further confirm with the administrator of the GigaDB that you can obtain this data at this stage of the process.
We have added a public location for sequencing data in SRR, which is ScienceDB (doi.org/10.57760/sciencedb.09166)
4. Why were reciprocal best blast groups used for scans of positive selection rather than the Orthofinder families?
>>>Response: Thanks for your comments. Both the RBH and Orthofinder were used to search for one-to-one orthologs across species, RBH method can obtain more orthologous genes (see Table S19). Therefore, we conducted a positive selection analysis using the reciprocal best blast groups.
5. Table S4 - what is the source of P.tepidariorum? D. spinosa?
>>>Response: Thanks for your comments. We have supplemented this information, as recorded below:
“
Species Assembly version (source) Source Assembly level
Parasteatoda
tepidariorum 10.11922/sciencedb.o00019.00014 Sciencedb Chromosome
Deinopis sp. This study Transcriptome assembly
”
6. Table S5 - there are no entries under O. sinensis. Should be, right?!
>>>Response: Thanks for your reminder. We have added this section.
7. Table S6 - is the lfc between P.tep and O.sin? How was this calculated? (see above). Is the p-value shown or the FDR? How were the numbers in Table S6 generated?
>>>Response: Thanks for your comments. Yes, this is a differential expression analysis between two species, where lfc represents log2 fold change, and the p-value was displayed in the table.
The calculation method was described in detail in the Methods section (lines 432–439). We used P.

tepidariorum as a control, and homologous genes were also characterized by gene IDs in P. tepidariorum. A positive value of log2(Fold Change) indicates that the expression level of this gene in O. sinensis is higher than that in P. tepidariorum, while a negative value indicates that the expression level of this gene in O. sinensis is lower than that in P. tepidariorum.
8. Table S7 - gene families or genes?
>>>Response: Thank you for your reminder. We have reorganized the table according to the attribution of gene families.
9. Table S8 - expanded gene families?
>>>Response: Thank you, we have reorganized the table according to the attribution of gene families.
10. S16 - I don't know what this table provides.
>>>Response: Thanks for your comments. This table presents the alterations in the quantity of structural domains observed in a pair of homologous genes, as detailed in the manuscript (lines 298–300). To enhance clarity and facilitate comprehension, we have included a descriptive header for each column.
11. Table S8 - Asianopis shows up but was never mentioned in text.
>>>Response: Thank you for your reminder. This is an oversight, we found it in Table 18, it should be Deinopis sp., we have corrected it.
12. Additional file 6 - Expression matrix - are these TPM values or something else? Is the * in this table the same as the Toxin gene expression matrix?
>>>Response: Thanks for your comments. The previous table was a TPM value expression matrix of genes in different O. sinensis tissues, and there was an error in the title, which should be "Figure 7". Its function is only to demonstrate that two genes in the O. sinensis in Figure 7B(h, linked with red ribbon) do indeed have expression levels. We have removed other genes in the new modification, leaving only the two required genes. We have renamed the table as Table S17 and placed it in Additional file 4.
13. Some typos:
>>>Response: Thanks for your comments. We have made modifications according to the comments.
a. Line 42: delete However,
>>> Response: See line 42. We have delete "However"
b. Line 59: sinensis needs an s on the end
>>> Response: See line 64. "The spider Octonoba sinensis belongs to the family Uloboridae."
c. Line 87: change providing to provides
>>> Response: See line 93. "which further provides evidence that uloborids are not equipped to deliver venom (Figure 1C, D)."
d. Line 242: lower case we
>>>Response: See line 253. "In addition, we referred to the venom gland specific expression module"
e. Line 267-268: incomplete sentence
>>>Response: Thanks for your comments. We have reorganized the language of this entire piece of content, with the following excerpts (lines 278–280):
"Given that O. sinensis had three latrotoxin homologs out of 12 venom components (highest category, Table S14) and latrotoxins are not known outside of Theridiidae, a phylogenetic analysis of this gene family has been conducted."
f. Line 316: Should there be a "lost" between "some of these" and "genes may be"?
>>>Response: Thanks for your comments (f–g). Based on the feedback of you and another reviewer, this section has been removed. Please refer to the first reply provided to you for details (Discussion lines 324–357).
g. Line 320: are the RTA clade really the youngest group of spiders?
>>>Response: Thanks for your comments (f–g). Based on the feedback of you and another reviewer, this section has been removed. Please refer to the first reply provided to you for details (Discussion lines 324–357).

Reviewer #2:
Zhang et al provide a multi-omics analyses on Octonoba sinensis (Uloboridae) and describe the genetics that could explain an adaptive response to the lack of venom in the group. The revised version of the manuscript is much improved with new data and new conclusions. However, I still have some concerns that need to be addressed before its publication.
>>>Response: We are once again deeply grateful for your insightful comments and suggestions, which have been instrumental in further enhancing the quality of our manuscript. Below, you will find our comprehensive response to each of your comments, addressing them point by point.

1. While the addition of Deinopis transcriptome to help with the gene family analyses is fine, this information needs to be included. For example, there is no mention in Figure 1 what the red dotted line means that leads to Deinopis and why it does not have a circle diagram. Table S4 does not have the NCBI info and Table S18 is labeled as Asianopis and not Deinopis. Given that the authors have added other genomes from RTA spiders I am not sure how much this transcriptome is added since it was not included in other analyses in the study. At this point, if it is not adding relevant information to the story, I would recommend removal. It almost looks like a last minute add on.
>>>Response: Thank you for your comments. Only one transcriptome is added. The transcriptome assembly of Deinopis sp. has served as an important resource of the manuscript, including the establishment of phylogenetic tree in Figure 2B, the formation of reciprocal best hit (RBH) gene sets, the analysis of selection pressure, and the quest for newly emerging as well as missing genes within the Uloboridae family. However, due to limitations in determining the copy number of homologous genes in the transcriptome assembly and the absence of contiguous genomic fragments, the transcriptome assembly of this spider was excluded from the investigation of gene family expansion and contraction, as well as the analysis of genome Highly-Conserved Elements (HCEs).
The RNA-seq data for transcriptome assembly is described in the Methods: 6. Genome annotation (lines 457–461)
To enhance clarity and readability, I have made several adjustments in the revised manuscript.
Lines 144–146: (The "red dashed line" in the revised manuscript has been changed to "red branch")
"Because Deinopis sp. lacks comprehensive genomic data and is limited to transcriptome information, this species was not included in the synteny analysis (red branch, Figure 2B)."
Lines 469–470:
"The latter did not include Deinopis sp., as the only transcriptome data cannot determine the number of gene copies."
Furthermore, unless explicitly stated that Deinopis sp. data was not utilized, we have incorporated the transcriptome assembly results of Deinopis sp. into various other analytical frameworks.
Regarding the entry pertaining to the Asianopis in Table S18 (Table S19 in the Revised version), it is indeed an error. The intended Asianopis should be Deinopis sp., and we have rectified this mistake in the revised version of the manuscript.
We have supplemented this information (Additional file 4: Table S4). The text is taken as follows:
"
Species Assembly version (source) Source Assembly level
…
Parasteatoda
tepidariorum 10.11922/sciencedb.o00019.00014 Sciencedb Chromosome
…
Deinopis sp. This study Transcriptome assembly
"
2. Figure 2C seems to be out of order to me. While reading it was hard to understand why it was there (other than being a comparative genome analysis) and not in Figure 5.
>>>Response: Thank you for your comments, this section (C) of Figure 2 has been moved to the Additional file 5: Figure S3.
3. Lines 154-155: expression analyses are not included in the material and methods. This is very important because (1) the reader would need to understand how the expression data was generated: reads mapped to genome? To transcriptome? Using what program? (2) what program and type of normalization was done for the DE analyses? The way these two sentences are written and figure 3 legend, they imply that the authors did DE using P. tepidariorum legs as the control for the legs of O. sinensis. I have never seen a DE across species that way to be able to create a volcano plot as shown on figure 3B. How it was done needs to be included.
>>>Response: Thank you for your comments, we have added this section in the Methods (see lines 432–438, Methods: 5. RNA extraction, sequencing and expression analysis), the text is taken as follows:
"Clean reads were aligned to the genome using Hisat2 [60], followed by the quantification of all samples with HTSeq [61] to determine the count value. Subsequently, TPMs were derived through automated scripts."
The "automation scripts" mentioned in the above modifications have been uploaded to the FTP server provided by GigaDB.
"In the differential expression analysis across two species, only Reciprocal Best Hits (RBH) genes were

extracted for quantification. To facilitate comparison, ortholog (RBH) gene IDs of O. sinensis were replaced by P. tepidariorum gene IDs for comparison and figure illustration. Finally, differential expression analysis was conducted by R package limma [62].”
The new RNA-seq data of P. tepidariorum are deposited into ScienceDB (doi.org/10.57760/sciencedb.09166)
For the threshold for identifying differentially expressed genes, we have added relevant descriptions in the caption of Figure 3 (lines 176–177), as recorded below:
“(only Reciprocal Best Hits genes were considered, Fold-change > 1.5, $p < 0.05$)”
In addition, we also used stricter screening conditions (Fold-change > 4, $p < 0.05$) to search for differentially expressed genes, and we created Figure S2 using the relevant results, which is similar to Figure 3C.
4. One major issue I found was the conclusion. From lines 309 to 324 I feel the argument being made is not completely logical and I can't see how it contributes to the manuscript. The discussion should be about the results and conclusions generated in this study and not on whether venom evolved before or after prey-catching webs. Here the authors have a chance to talk about the metabolic related genes that are under positive selection and how it could relate to an increase endurance in O. sinensis. What are the potential functions of the expanded or new emergent gene families? How all of that relates to no having venom glands and using this extensive prey-wrapping technique. All of that is not included in the conclusion as it stands. This section needs to be rewritten.
>>>Response: Thanks for your comments. Based on comments, we have adjusted this section of Discussion and removed discussions about “whether venom evolved before or after prey-catching webs” (The second paragraph in the original Discussion)
In the revised Discussion, we discussed the genomic features related to excessive physical predation and venom glands loss in detail, and talk about how it could relate to an increase endurance in O. sinensis. Please refer to lines 324–355 of the revised manuscript for specific modifications.
5. Line 49: delete "changes"
>>>Response: Thank you for your suggestions, we have made the corresponding modifications (see lines 47–50).
6. Line 51: instead of "these spiders" use uloborids
>>>Response: Thank you for your reminder. We have made the corresponding modifications (see line 51).
“Due to the absence of venom glands, the predation methods of uloborids are also relatively specialized.”
7. Line 52: instead of "this group" use Uloboridae
>>>Response: Thank you for your suggestions, we have adjusted the language of this section to make it more relevant to the title of our article. This section comes from the discussion in the original version. (see lines 52–60).
“Generally speaking, using venom to paralyze prey is an effective chemical attack strategy. However, many kinds of spiders integrate both chemical attack and physical attack strategies. Certain species within the Araneoidea family utilize entanglement initially to restrain larger prey before a venomous final strike [6]. Although this predatory tactic may alleviate the selective pressure associated with venom usage, entanglement appears rudimentary in comparison to the prey-wrapping behavior exhibited by uloborids. In Uloboridae, this physical attack as the sole means of attack can span from a few minutes to nearly an hour, with the spider silk utilized sometimes exceeding a hundred meters in length [7–9]. Therefore, considerable physical endurance is indispensable for the successful execution of this predatory tactic.”
8. L55: add a reference to the first sentence
>>>Response: Thank you for your comments, we have made the corresponding modifications (see lines 60–62).
“Previous anatomical records indicate that spiders in the family Uloboridae have well-developed trachea [10], and many branches of the trachea extend into the prosoma and appendages [11].”
9. Line 75: delete "in its anatomy"
>>>Response: Thank you for your suggestions, we have made the corresponding modifications (see lines 80–81).
10. Line78: Suggest changing the title of the section to something like: Prey-wrapping behavior observations and fand morphology
>>>Response: Thank you for your suggestions, we have made the corresponding modifications (see line 84).
“1. Prey-wrapping behavior observations and fang morphology”
11. Line82: Araneoidea comes out of nowhere. Maybe say "time for other spider species (Arnaeoidea,

9.7+- …"
>>>Response: Thank you for your comments, we have made the corresponding modifications (see lines 88–89).
“This time is much higher than the previously recorded wrapping time of other spider species (Araneoidea, 9.7 ± 3.0 seconds for small prey, 26 ± 42 seconds for big prey) [6].”
12. Line 87: provides instead of providing. And "uloborids"
>>>Response: Thank you for your comments, we have made the corresponding modifications (see lines 92–93).
“which further provides evidence that uloborids are not equipped to deliver venom (Figure 1C, D).”
13. Line 90: add "s" to observation
>>>Response: Thank you for your comments. We have made the corresponding modifications (see line 96).
“Figure 1: Observations of Octonoba sinensis.”
14. Line 91: delete channel (D)
>>>Response: Thank you for your suggestions, we have made the corresponding modifications (see line 97).
15. Line 92 indicates instead of indicating. Delete "channel"
>>>Response: Thank you for your reminder, we have made the corresponding modifications (see lines 97–98).
16. Line 99: spell out 9
>>>Response: Thank you for your reminder, we have made the corresponding modifications (see lines 104–105).
“A total of 20 scaffolds were obtained, of which more than 99.9% of the sequences were loaded onto nine scaffolds that reached the chromosome level (Figure 2A),”
17. L126: maybe add that the proportions circles are next to each species
>>>Response: Thank you for your reminder. We have reorganized the language of this entire piece of content, the text is taken as follows (lines 131–132):
“The collinearity between O. sinensis and other spider species is visualized through circle diagrams positioned adjacent to each respective species.”
18. Line 156: add "with" after genes and delete "the" after expression
>>>Response: Thank you for your comments. Based on the feedback from you and another reviewer, this section has been rewritten (lines 160–162).
“Compared to the legs of P. tepidariorum, genes with higher expression in the legs of O. sinensis were predominantly enriched in the mitochondrial matrix, while significant enrichment was also observed in other GO terms pertaining to mitochondria (Figure 3C, Figure S2).”
19. Line 171 add "term" after GO
>>>Response: Thank you for your comments, we have made the corresponding modifications (see line 179).
“(C) GO terms enrichment analysis of genes expressed at higher levels”
20. Line 172: delete (D-E).
>>>Response: Thank you for your reminder, we have made the corresponding modifications (see lines 180–181).
21. Section 5 should include how the authors identified the expanded and new emergent gene families. Just one or two sentences.
>>>Response: Thank you for your reminder. We have made the corresponding modifications (see lines 188–189).
“To compare the genomic differences between Uloboridae and other spiders, we used CAFE v4.2 [24] to analyze the gene family expansions and contractions.”
(lines 205–207)
“Based on the results of orthologous gene identification, we screened for gene families that are exclusively shared in Uloboridae and not found in any other spider species (species in Figure 2B), designating them as new emergent gene families.”
22. Section 5 Lines 181-182: could rephrase these sentences to be clearer. For example: In O. sinensis, we found four of these families to have a high number of annotated genes that corresponded to: …
>>>Response: Thank you for your reminder. We have made the corresponding modifications (see lines 190–191).
“In O. sinensis, we found four of these families to have a high number of annotated genes that

corresponded to:”
23. Figure 4 shows with very low quality on my copy.
>>>Response: Thank you for your comments. We have thoroughly reexamined the image, confirming that the uploaded version possesses a size of 738 KB and a resolution of 2008 × 1279 pixels.
24. In general, when a number has more than 3 digits a comma should be added. For example, Line 243 - instead of 1088 write 1,088. There are too many instances to numbered them all. Please check the document.
>>>Response: Thank you for your comments. We have carefully reviewed the entire text and corrected all such issues.
25. Line 208: add Species before phylogeny
>>>Response: Thank you for your comments. We have made the corresponding modifications (see line 219).
“(A) Species phylogeny for calculating the expansion and contraction of gene families.”
26. Line234: spell out 5
>>>Response: Thank you for your comments. We have made the corresponding modifications (see line 245).
“we found that five genes belong to two toxin related gene families”
27. Line 245 delete "gene" after ")"
>>>Response: Thank you for your comments. We have made the corresponding modifications (see lines 255–256).
28. Figure 5 and 6. The expression plots do not indicate the type of values. Are this TPMs? RPKM? Raw reads?
>>>Response: Thank you for your comments. We have supplemented this information. The text is taken as follows:
(Figure 5 lines 262–266)
“The expression patterns of the genes specifically absent in Uloboridae were examined in the model species Parasteatoda tepidariorum. The heatmap is plotted based on the Z-score transformed from Transcripts Per Million (TPM) values. Different colored markers are used to distinguish between distinct gene types, while an asterisk identifies the gene that is notably absent from the venom gland-specific expression module of P. tepidariorum.”
(Figure 6 lines 308–309)
“The heatmap is plotted based on the Z-score transformed from Transcripts Per Million (TPM) values.”
29. Figure 5A legend. The GO enrichment is for which species? Ptep? O sin?
>>>Response: Thank you for your comments. O. sinensis. We have supplemented this information. The text is taken as follows:
(lines 260–261)
“(A) GO enrichment of genes under relaxed selection in Octonoba sinensis.”
30. Line 258 add "in O. sinensis, we .."
>>>Response: Thank you for your comments. We have made the corresponding modifications (see lines 269–271).
“To search for toxin genes in O. sinensis, we integrated the results of previous studies and established a comprehensive toxin protein database (Additional file 6) and screened toxin gene homologs with the same threshold in different species.”
31. Line 260: delete have
>>>Response: Thank you for your comments. We have made the corresponding modifications (see lines 271–272).
32. Line 265: add " in the venom glands of P. tepidariorum"
>>>Response: Thank you for your comments. We have made the corresponding modifications (see lines 276–277).
“Nevertheless, through the characterization of expression patterns for these O. sinensis homologs in the venom glands of P. tepidariorum,”
33. Lines 267-270: these sentences are out of order and hard to follow. It is not clear why the authors are talking about latrotoxin or why an additional gene tree was generated for this venom component and not the other ones. I suggest the authors start with something along the lines of "Given that octonoba had four latrotoxin homologs out of 12 venom components (highest category, Table S14) and latrotoxins are not known outside of Theridida
>>>Response: Thank you for your comments. We have reorganized the language of this entire piece of

content, the text is taken as follows:
(line 276–280)
“Nevertheless, through the characterization of expression patterns for these O. sinensis homologs in the venom glands of P. tepidariorum, we discovered that the toxin-related homologs of O. sinensis do not exhibit high expression levels in venom glands (Figure 6). Given that O. sinensis had three latrotoxin homologs out of 12 toxin homologs (highest category, Table S14) and latrotoxins are not known outside of Theridiidae, the phylogenetic analysis of this gene family has been deployed.”
