# Supplementary material for: A trade-off in evolution: the adaptive landscape of spiders without venom glands
Source: Gigascience. 2024 Aug 5;13:giae048. doi: 10.1093/gigascience/giae048 (PMC11299198; doi:10.1093/gigascience/giae048)
Supplement: giae048_Response_to_Reviewer_Comments_Revision_2 [file giae048_response_to_reviewer_comments_revision_2.pdf]

# Author's Response To Reviewer Comments

Response to reviewers
Dear Editor Zhang

I hope this email finds you well. We have carefully considered all the feedback and have made the necessary revisions to our paper.
We would like to emphasize that the entire review process has been an incredibly enriching experience for us. The insights and suggestions from the reviewers and editor have not only strengthened our research but have also broadened our understanding of the field. We are grateful for the time and effort the reviewers have invested in providing us with such valuable feedback.
Thank you.
Best regards,

Yiming Zhang; Shuqiang Li
Institute of Zoology, Chinese Academy of Sciences
1. Beichen West Road, Chaoyang District
Beijing 100101, P. R. China
Tel: +86-13363636950
Fax: +86-10-64807216
Email: zhangyiming@ioz.ac.cn; lisq@ioz.ac.cn

Editor comments
Please include a point-by-point within the 'Response to Reviewers' box in the submission system. Please ensure you describe additional experiments that were carried out and include a detailed rebuttal of any criticisms or requested revisions that you disagreed with. Please remember to make revisions per Dr. Sandra Correa-Garhwal's comments 34-48 in the second round of review.
>>>Response: Thank you for giving us the opportunity to supplement these responses. We have responded to each point individually.
34. Figure S4: The black text in the figure should be added the figure legend. For the green genes, add the species they come from.
>>>Response: Thank you for your comments. We have moved the black text from the figure to the figure legend and listed the species sources of the reference toxin genes (marked with green color).
“Figure S4: Phylogenetic tree of neurotoxin genes (Latrotoxin). Tips labelled in red font represent Octonoba sinensis genes, those labelled in green font represent the reference sequences of different Latrotoxins genes previously studied, others represent Parasteatoda tepidariorum genes. Tips labelled in green font come from different species, Latrodectus tredecimguttatus: sp|Q25338|LITD_LATTR, sp|P23631|LATA_LATTR, sp|Q02989|LITA_LATTR, sp|Q9XZC0|LCTA_LATTR; Steatoda grossa: sp|L7X8P2|LATA_STEGR; Latrodectus geometricus: sp|L7XCU0|LATA_LATGE; Latrodectus h esperus: sp|P0DJE3|LATA_LATHE; Latrodectus pallidus: sp|L7XDS4|LATA_LATPL; Latrodectus hasselti: sp|G0LXV8|LATA_LATHA. The red font with black stroke is a similar object below the recognition threshold as an outlier.”
35. Lines 281-290: I think is very interesting that there is conservation in the where these genes are in both species regardless of function. The paragraph will benefit from editing because the results are not coming thru. I also suggest that the authors describe all genes that fall under this category, I think that there are only three by looking at Figure 6. Given the small number of instances, it seems odd to only describe one of them. These results should also be added to the discussion: we found these genes, they are expressed (or not) in XX tissue, and we think the function in octonoba is XX.
>>>Response: It is true that there are three pairs of toxin homologs preserved in the syntenic region, but only one of them was highly expressed in the venom glands of P. tepidariorum, which we consider a relatively reliable toxin gene in P. tepidariorum. The others showed their highest expression in other tissues of P. tepidariorum (Figure 6) and may be performing non-toxic functions, so we didn't have a detailed discussion on the two genes mentioned later.
In the revised manuscript, we mentioned the above content and added some discussion at this paragraph.

“By searching for toxin gene homologs in collinearity fragments of O. sinensis and P. tepidariorum, a particular class of genes was found in O. sinensis. These genes are located in the same place as the P. tepidariorum toxin gene homologs in the collinearity segment, but they can no longer be identified as toxin genes (below the minimum recognition threshold, see methods) (Figure 7B, red ribbon). There are three pairs of such genes, includes one pair of CRISP genes (g31478~LOC107437238) and two pairs of ICK genes (g6736~LOC107446942 and g6736~LOC107446932). Their expression patterns in P. tepidariorum indicate that only the CRISP gene is a reliable toxin gene (Figure 6, black squares). In O. sinensis, this CRISP gene (g31478) cannot be unambiguously classified as a homolog of toxin genes due to changes in protein structure (Additional file 4: Table S16). However, compared with other toxin genes which it is difficult to find pseudogenes, the ortholog of this CRISP gene in O. sinensis have complete gene structures, and can be expressed in multiple tissues of a venomless spider (Additional file 4: Table S17). These all indicate that this CRISP gene (g31478) must play the role of a non-toxic gene. We believe that this observation suggests a potential functional shift between toxic and non-toxic genes in spiders.” (see lines 292–305)
We also mentioned relevant content in the Discussion section of the article.
“Multi-omics analysis revealed the absence or possible functional shift of toxin genes in O. sinensis.” (see lines 330–331)
“Simultaneously, through analyzing expression patterns and synteny relationships, it was discovered that toxin genes in venomous spiders are expressed in other tissues of uloborids and may perform non-toxic functions. This observation suggests a potential functional shift between toxic and non-toxic genes in spiders. In addition to toxin genes, the absence of certain genes in uloborids raises concerns. These include protein c-ets-2, CC2D, and …” (see lines 357–361)
36. Figure 6 The legend that is inside the figure, starting with the asterisk should be included in the legend and maybe not in the figure. It also refers to itself which is odd)
>>>Response: Thank you for your comments. We have moved the corresponding information from the figure to the figure legend.
37. Figure 7. What the red line in A means needs to be included in the legend for panel A. Also, include that the different colors (green vs. blue) in the genes for panel B indicate.
>>>Response: Thank you for your comments. The “red line” indicates that the original toxin homolog has been retained in the collinearity. The genes with different colors in the Figure 7B represent different transcription directions. We have added relevant information in the figure legend.
38. Line 300: add () to A
>>>Response: Thank you, we have made the correction.
39. Line 301: add () to A
>>>Response: Thank you, we have made the correction.
40. For the methods, include the type of camera that was used to film the pre-wrapping behavior.
>>>Response: Thank you for your comments. We have supplemented this information. The text is taken as follows:
“We recorded a series of videos to observe the predation behavior, using Logitech StreamCam 960-001282.” (lines 386–387)
41. Line 393 Additional file 6 has no information related to tissue samples
>>>Response: Thank you for your comments. The tissue sample information involved in Additional file 6 is in the Section 5 of Methods (see lines 428–431). The purpose of Additional file 6 is to demonstrate two genes (Figure 7B, genes linked with red ribbon) in the O. sinensis have indeed been expressed. We have removed other genes in the new modification, leaving only the two required genes. We have renamed the table as Table S17 and placed it in Additional file 4.
42. Line 400: replace "have been" with were
>>>Response: Thank you, we have made the correction.
43. Line 406 spell out 9. Add "of" after Gb
>>>Response: Thank you, we have made the correction.
44. Section 7 - was this methodology used for figure s4? How was that tree generated?
>>>Response: Thank you, the phylogenetic tree of latrotoxin homologs (Figure S4) was reconstructed using the neighbor-joining method, following alignment of the full-length protein sequences via Mafft. We have supplemented this information in this Section.
45. How were the cluster analyses done?
>>>Response: Thank you for your comments. One-to-one ortholog identification among ten species (Figure 2B) was performed using the RBH method by blastp. O. sinensis was used as a reference species.

Finally, 5,848 RBH clusters (Additional file 4: Table S19) were retained for analysis. We have supplemented this information. (lines 484–486)
46. Line 456 spell out HCEs
>>>Response: Thank you, we have made the correction. (see line 502)
47. Line 468: instead of "previous databases" explain what those are. Downloaded sequences from multiple sources (NCBI/Arachnoserver?) that includes multiple species? Or includes venom components from all spiders and other animals?
>>>Response: Thank you, we have supplemented this information. This information comes from previous studies, and we have listed the articles published in these studies as references and added a brief description in the manuscript.
“Based on ArachnoServer 3.0 [89], a specialized spider venom database, and integrating toxin protein sequences obtained from other toxin research of spiders [37, 38, 90], we have compiled a new reference dataset.” (see lines 514–516)
48. Availability of data: the NCBI code given is not functional and there is an empty parenthesis for the gigaDB Digital Repository.
>>>Response: Thanks for your suggestion, the relevant data has been uploaded to the FTP server provided by GigaDB and will be made public after the article is published. At that time, there will be a valid link filled in the current parentheses. At this stage, you can obtain the corresponding information through the FTP link.
We have provided a new available BioProject Accession number (PRJNA1018860)
In addition, we have added a public location for sequencing data in SRR, which is ScienceDB (doi.org/10.57760/sciencedb.09166).

Reviewer #2:
I believe the manuscript has shown improvement since the last revision and has effectively addressed most of my concerns and comments. However, I think the discussion section still requires further refinement to convey the main message clearly. For instance, consolidating the concluding remarks into the final paragraph and keeping all conclusions within that paragraph would enhance coherence.
>>>Response: I would like to express my sincere gratitude for your assistance and support throughout the entire process. We have learned a great deal from your feedback and suggestions. Thank you for your valuable contributions.
We have consolidated all concluding remarks into the last paragraph of the discussion.
“For predators, there exists a pervasive evolutionary trade-off between chemical and physical attack strategies. Previous research has frequently favored the exploration of chemical strategies, particularly venom. However, against the backdrop of nearly all spiders being toxic predators, our study delves into the genetic basis underlying the alternative choice in this trade-off. Unsurprisingly, reliable toxin gene was not identified in Uloboridae, but these adaptive evolutions ranging from muscle to aerobic respiration and then to supply of energy substances, provide strong support for the exceptional physical endurance demands of this group, and compensate for their decreased adaptability due to the absence of venom glands. Furthermore, some development-related gene and element deletions were observed in uloborids. Although the association between these deletions and the absence of spider venom glands remains unclear, they still hold potential for exploring the evolutionary mechanisms underlying this phenomenon.”
(see lines 366–376)
Please ensure that numbers under ten are spelled out. Additionally, after "protocol" in line 520, add a period. Include the number for the GigaDB Digital Repository. Furthermore, review the references for format consistency. For example, Reference 50 has the year in bold instead of the issue, and Reference 79 is written in all caps.
>>>Response: Thank you for your comments. We searched the entire text and spelled out the numbers under ten in the manuscript. Except for some special positions, such as such as “k > 1” in line 496
There is a period missing at the end of the main text (after "protocol"). Thank you for your reminder. We have made the necessary changes here.
For the access address of GigaDB, currently there is only an FTP server connection method available for reviewers to access. I have communicated with the GigaDB office and they said that the corresponding link will be provided when the article is published.
We have thoroughly organized the references. The reference 50 you mentioned has the same volume

number as the publication year, so the reference management APP automatically generate it in bold (see reference 51). Reference 79, we have made the correction. (see reference 82).
