# Supplementary material for: A trade-off in evolution: the adaptive landscape of spiders without venom glands
Source: Gigascience. 2024 Aug 5;13:giae048. doi: 10.1093/gigascience/giae048 (PMC11299198; doi:10.1093/gigascience/giae048)
Supplement: giae048_Reviewer_1_Report_Original_Submission — Nadia Ayoub -- 11/10/2023 Reviewed [file giae048_reviewer_1_report_original_submission.pdf]

Reviewer Report

Title: A Trade-off in Evolution: The Adaptive Landscape of Spiders without Venom Glands

Version: Original Submission **Date:** 11/10/2023

Reviewer name: Nadia Ayoub

Reviewer Comments to Author:

Zhang et al. provide a chromosome level assembly of Octonoba sinensis, a member of the one and only family of spiders that does not possess venom glands. The sequence and annotation is highly valuable for arachnologists and arthropod biologists more generally. The manuscript also provides some interesting analyses, focusing on potential gene loss associated with loss of venom glands. However, I have concerns that need to be addressed prior to publication.
First, the closest relative included in the gene family analyses, Stegodyphus dumicola (Eresidae), is quite distantly related from Uloboridae. Thus, inferences about gene loss or positive selection associated with the loss of venom glands are quite difficult to make with confidence. At a minimum, the authors need to clarify the lack of inclusion of any other members of the UDOH grade. As written (lines 180-183), the authors insinuate that these close relatives are included in the analysis of gene loss and positive/relaxed selection. This is not true. Although, there are no genomes currently available for members of Deinopidae, Oecobiidae, Hersiliidae, there are transcriptomes. The authors could consider an additional analysis using these transcriptomes to track gene loss.
Related to the above comment, a high quality chromosome-level assembly is available for Uloborus diversus. I strongly recommend including Uloborus in synteny and gene family analyses. As another uloborid species, its inclusion would bolster inferences about gene loss in Uloboridae or positive selection/relaxed selection in Uloboridae, and not just in Octonoba.
Related to above, there are three genomes available for RTA clade species in NCBI. It doesn't look like there are associated publications yet, but the authors might consider inclusion to break up the evolutionary distance between Uloboridae and other species currently in the analysis; if the authors are set on only using species with whole genomes.
For positive selection analyses, is finding metabolic genes unusual? E.g. if the same analysis was done moving a species with venom glands to the target would metabolic genes still arise? Perhaps a different set of genes, but could be something more general to arachnids/arthropods?
The relevance of the "absent" regions and regions under relaxed selection wasn't clear. Was there a specific hypothesis being tested? E.g. that toxin genes are more likely to be lost or under relaxed selection? Clarify.
Similarly, the point of the SNP analysis wasn't clear. Was there a driving hypothesis? What is learned from the analysis?
How were venom specific modules identified in Parasteatoda? If the authors generated their own RNA seq for Parasteatoda, please include accessions.
I'm not convinced latrotoxins exist outside of Theridiidae. Is it possible the significant BLAST alignments are based on ankyrin repeats found in latrotoxins and other types of genes? Please provide more

evidence in support of latrotoxins in distant relatives of Latrodectus.
Language and readability were mostly fine, but the manuscript could use editing in many places - too many for me to enumerate. One item that jumped out was the use of "losed" for "lost" in some figures. Please correct and have someone read the whole manuscript carefully.

Methods

Are the methods appropriate to the aims of the study, are they well described, and are necessary controls included? Choose an item.

Conclusions

Are the conclusions adequately supported by the data shown? Choose an item.

Reporting Standards

Does the manuscript adhere to the journal's guidelines on minimum standards of reporting? Choose an item.

Choose an item.

Statistics

Are you able to assess all statistics in the manuscript, including the appropriateness of statistical tests used? Choose an item.

Quality of Written English

Please indicate the quality of language in the manuscript: Choose an item.

Declaration of Competing Interests

Please complete a declaration of competing interests, considering the following questions:

- Have you in the past five years received reimbursements, fees, funding, or salary from an organisation that may in any way gain or lose financially from the publication of this manuscript, either now or in the future?
- Do you hold any stocks or shares in an organisation that may in any way gain or lose financially from the publication of this manuscript, either now or in the future?
- Do you hold or are you currently applying for any patents relating to the content of the manuscript?
- Have you received reimbursements, fees, funding, or salary from an organization that holds or has applied for patents relating to the content of the manuscript?
- Do you have any other financial competing interests?

- Do you have any non-financial competing interests in relation to this paper?

If you can answer no to all of the above, write 'I declare that I have no competing interests' below. If your reply is yes to any, please give details below.

I declare that I have no competing interests.

I agree to the open peer review policy of the journal. I understand that my name will be included on my report to the authors and, if the manuscript is accepted for publication, my named report including any attachments I upload will be posted on the website along with the authors' responses. I agree for my report to be made available under an Open Access Creative Commons CC-BY license (http://creativecommons.org/licenses/by/4.0/). I understand that any comments which I do not wish to be included in my named report can be included as confidential comments to the editors, which will not be published.

Choose an item.

To further support our reviewers, we have joined with Publons, where you can gain additional credit to further highlight your hard work (see: https://publons.com/journal/530/gigascience). On publication of this paper, your review will be automatically added to Publons, you can then choose whether or not to claim your Publons credit. I understand this statement.

Yes Choose an item.
