# Supplementary material for: A trade-off in evolution: the adaptive landscape of spiders without venom glands
Source: Gigascience. 2024 Aug 5;13:giae048. doi: 10.1093/gigascience/giae048 (PMC11299198; doi:10.1093/gigascience/giae048)
Supplement: giae048_Reviewer_1_Report_Revision_1 — Nadia Ayoub -- 2/18/2024 Reviewed [file giae048_reviewer_1_report_revision_1.pdf]

Reviewer Report

Title: A Trade-off in Evolution: The Adaptive Landscape of Spiders without Venom Glands

Version: Revision 1 **Date:** 2/18/2024

Reviewer name: Nadia Ayoub

Reviewer Comments to Author:

The revised manuscript addresses most of my initial concerns and is much better overall! I'm not totally convinced of a trade-off between venom and web production (or do y'all mean the tradeoff is with excessive physical predation???). Perhaps soften this language? Clarify in discussion. Some additional clarifications are needed prior to publication.

1. The gene expression analyses are never explained - how was expression level determined - e.g. read mapping using what? What program(s) with what parameters used to compare tissues within species? What constitutes a differentially expressed gene? What methods were used to compare expression patterns between species?
2. In Figure 3B, is expression analysis for both P. tepidariorum and O. sinensis? Is it comparison of the two? For Figure 3C, is the GO enrichment for genes significantly overexpressed in leg tissues of O. sinensis relative to some other O. sinensis tissue, or relative to P. tepidariorum legs?
3. Is it possible to access all these new annotations? Would be great to point to their public location.
4. Why were reciprocal best blast groups used for scans of positive selection rather than the Orthofinder families?
5. Table S4 - what is the source of P.tepidariorum? D. spinosa?
6. Table S5 - there are no entries under O. sinensis. Should be, right?!
7. Table S6 - is the lfc between P.tep and O.sin? How was this calculated? (see above). Is the p-value shown or the FDR? How were the numbers in Table S6 generated?
8. Table S7 - gene families or genes?
9. Table S8 - expanded gene families?
10. S16 - I don't know what this table provides.
11. Table S8 - Asianopis shows up but was never mentioned in text.
12. Additional file 6 - Expression matrix - are these TPM values or something else? Is the * in this table the same as the Toxin gene expression matrix?
13. Some typos:

a. Line 42: delete However,
b. Line 59: sinensis needs an s on the end
c. Line 87: change providing to provides
d. Line 242: lower case we
e. Line 267-268: incomplete sentence
f. Line 316: Should there be a "lost" between "some of these" and "genes may be"?
g. Line 320: are the RTA clade really the youngest group of spiders?

Methods

Are the methods appropriate to the aims of the study, are they well described, and are necessary controls included? Choose an item.

Conclusions

Are the conclusions adequately supported by the data shown? Choose an item.

Reporting Standards

Does the manuscript adhere to the journal's guidelines on minimum standards of reporting? Choose an item.

Choose an item.

Statistics

Are you able to assess all statistics in the manuscript, including the appropriateness of statistical tests used? Choose an item.

Quality of Written English

Please indicate the quality of language in the manuscript: Choose an item.

Declaration of Competing Interests

Please complete a declaration of competing interests, considering the following questions:

- Have you in the past five years received reimbursements, fees, funding, or salary from an organisation that may in any way gain or lose financially from the publication of this manuscript, either now or in the future?
- Do you hold any stocks or shares in an organisation that may in any way gain or lose financially from the publication of this manuscript, either now or in the future?
- Do you hold or are you currently applying for any patents relating to the content of the manuscript?
- Have you received reimbursements, fees, funding, or salary from an organization that holds or has applied for patents relating to the content of the manuscript?
- Do you have any other financial competing interests?
- Do you have any non-financial competing interests in relation to this paper?

If you can answer no to all of the above, write 'I declare that I have no competing interests' below. If your reply is yes to any, please give details below.

I declare that I have no competing interests.

I agree to the open peer review policy of the journal. I understand that my name will be included on my report to the authors and, if the manuscript is accepted for publication, my named report including any attachments I upload will be posted on the website along with the authors' responses. I agree for my report to be made available under an Open Access Creative Commons CC-BY license (http://creativecommons.org/licenses/by/4.0/). I understand that any comments which I do not wish to be included in my named report can be included as confidential comments to the editors, which will not be published.

Choose an item.

To further support our reviewers, we have joined with Publons, where you can gain additional credit to further highlight your hard work (see: https://publons.com/journal/530/gigascience). On publication of this paper, your review will be automatically added to Publons, you can then choose whether or not to claim your Publons credit. I understand this statement.

Yes Choose an item.
