# Supplementary material for: A trade-off in evolution: the adaptive landscape of spiders without venom glands
Source: Gigascience. 2024 Aug 5;13:giae048. doi: 10.1093/gigascience/giae048 (PMC11299198; doi:10.1093/gigascience/giae048)
Supplement: giae048_Reviewer_2_Report_Original_Submission — Sandra Correa-Garhwal -- 11/13/2023 Reviewed [file giae048_reviewer_2_report_original_submission.pdf]

Reviewer Report

Title: A Trade-off in Evolution: The Adaptive Landscape of Spiders without Venom Glands

Version: Original Submission **Date:** 11/13/2023

Reviewer name: Sandra Correa-Garhwal

Reviewer Comments to Author:

The manuscript titled "A Trade-off in Evolution: The Adaptive Landscape of Spiders without Venom Glands" described the a comprehensive study of the spider Octonoba sinensis to identify pathways related to metabolism and trachea development that could help explain energy requirements by the spider's extensive wrapping behavior. The manuscript also explores genetic footprint of venom components still present in the venom less spider. The manuscript has a lot of exciting new data, I find the metabolic and trachea genetics very interesting to specialist as well as the general public. I do however have some issues with the venom sections that in my view need to be addressed.

It is worth noting that Uloboridae is not the only spider family to not have venom glands. Holarchaea is another family that has also been described as not having venom glands. This should be mentioned/addressed.

I have some issues with Section 7 of the manuscript: "Differential retention of major toxin genes in O. sinensis". Knowing that the family does not have venom glands and has not many millions of years it is not acceptable for the authors to refer to the genes they find in O. sinensis with homology to known venom components as toxin genes. This will lead to many misinterpretations although admittedly it sounds a lot catchier. Moreover, the families that were identified while all are known to be venom components, not all are toxins. For example, the Translationally Controlled Tumor Protein (TCTP) a histamine-releasing factor acts together with Together the cysteine-rich secretory protein (CRISP) family, contributing to inflammatory responses (Sade et al., 2012; Boia-ferreira et al., 2019; Justa et al., 2020). Given this, the authors will need to tone down the claim of having found toxin in a spider that does not have venom glands. This is not surprising assuming a secondarily loss in the family (the most parsimonious interpretation). I believe this also leads to some misinterpretations of the results; it is my understanding that most venom components are hypothesized to have evolved from ancestor proteins that had normal physiological functions that were then recruited. This information is not concluded from the current manuscript, there is a large body of evidence that supports the evolution of venom proteins via processes such as gene duplication followed by subfunctionalization and/or neofunctionalization, horizontal gene transfer, single gene co-option, etc. It follows that expression of venom homologs in the brain region should not come as a surprise since a lot of what are refer to as toxins are thought to be recruited from the neuropeptides, molecules such as latrodectins (McCowan & Garb 2014). I suggest a major modification of the results/discussion section having more emphasis on the metabolic data and how this can potentially be a trade-off because as written, the manuscript does not describes a trade-off between wrapping and lack of venom.

The reason why P. tepidarioriom was used as a reference comparison needs to be stated somewhere. I think it is somewhat arbitrary and I personally would like to hear the authors' reasons. Also why U.

diversus, another Uloboridae was not included in the study is beyond me. This species also has a chromosome level genome and could add to the story.
L45-47: I am not sure I understand the meaning of these sentences and I suggest their removal.
L51-54: There are many spider families that have well-developed trachea with branches extended into their prosoma, such as salticidae and Cybaeidae. To make the case more compelling for why it is particularly important for Uloboridae to have this adaptation I suggest the authors include more detrain about the greater need for energy in the limbs or greater metabolic input and thus there could be a greater presence of mitochondria in the legs or upregulation of genes related to energy metabolism. If this is not expanded, it is unclear why O. sinensis is indeed a good model for this study.
L55: Do not start a sentence with a species name.
L76-80: Uloborid spiders have been described as lacking both venom glands and openings on the fangs for the delivery of venom (Opell 1979; Weng et al. 2006). It is not surprise that you did not observe fang openings in O. sinensis fangs. Therefore, I suggest the authors change the wording from 'prove' to further providing evidence that uloborids are not equipped to deliver venom.
L 94: Not sure "as a percentage of … 98.11%" is needed.
L100: change to 24,579 and 24,563
L101: "in at least one the following databases: "
L250-253: I agree that it could be possible that these genes were removed from the genome but it is hard to tell from the current data if this was quick or not after selective pressures changed.
L297-298: While I do not agree with the statement, at the very least the authors need to provide a lot more information to back up this claim.
L139-140: Sentence is confusing as written and should be rewritten or deleted.
L324: Where the samples use males or females? Mature or immature? This will be important for expression levels. Why were the spiders starved for a week?
L338: what type of tissue was used to generate the genome? Whole spiders?
L349: experiments "were" conducted.
L372: BUSCO should be spelled out, explained and cited the first time is used.
L375: what do you mead by spinneret? If you dissected the silk glands out (all the silk glands out) then you not have any tissue other than connective and/or fat attached the spinneret. This should be clarified. If the authors took out the larger glands and left the smaller ones behind then the names should reflect that. Are there any images of the tissues used? I will be curious to see what part of the brain and the gut was used for example. Whether the spiders where male or female, at what stage and how many where used should be included for both species.
L396: Why was the Uloboridae Uloborus diversus not included in this study? To my knowledge it is also a chromosome level assembly and would have made a stranger case for the conclusions.
The manuscript contains both Fig and Figure. For consistency only use one.
Figure 1 suggestions:
B- Adult male or female? Move the scale bar to bottom right.
C- I find this section to be very confusing and should perhaps be its own figure. The numbering inside the circles make it hard to understand to me. I suggest a different organization for this figure.

Methods

Are the methods appropriate to the aims of the study, are they well described, and are necessary controls included? Choose an item.

Conclusions

Are the conclusions adequately supported by the data shown? Choose an item.

Reporting Standards

Does the manuscript adhere to the journal's guidelines on minimum standards of reporting? Choose an item.

Choose an item.

Statistics

Are you able to assess all statistics in the manuscript, including the appropriateness of statistical tests used? Choose an item.

Quality of Written English

Please indicate the quality of language in the manuscript: Choose an item.

Declaration of Competing Interests

Please complete a declaration of competing interests, considering the following questions:

- Have you in the past five years received reimbursements, fees, funding, or salary from an organisation that may in any way gain or lose financially from the publication of this manuscript, either now or in the future?
- Do you hold any stocks or shares in an organisation that may in any way gain or lose financially from the publication of this manuscript, either now or in the future?
- Do you hold or are you currently applying for any patents relating to the content of the manuscript?
- Have you received reimbursements, fees, funding, or salary from an organization that holds or has applied for patents relating to the content of the manuscript?
- Do you have any other financial competing interests?
- Do you have any non-financial competing interests in relation to this paper?

If you can answer no to all of the above, write 'I declare that I have no competing interests' below. If your reply is yes to any, please give details below.

I declare that I have no competing interests

I agree to the open peer review policy of the journal. I understand that my name will be included on my report to the authors and, if the manuscript is accepted for publication, my named report including any attachments I upload will be posted on the website along with the authors' responses. I agree for my report to be made available under an Open Access Creative Commons CC-BY license (http://creativecommons.org/licenses/by/4.0/). I understand that any comments which I do not wish to be included in my named report can be included as confidential comments to the editors, which will not be published.

Choose an item.

To further support our reviewers, we have joined with Publons, where you can gain additional credit to further highlight your hard work (see: https://publons.com/journal/530/gigascience). On publication of this paper, your review will be automatically added to Publons, you can then choose whether or not to claim your Publons credit. I understand this statement.

Yes Choose an item.
