# Supplementary material for: A trade-off in evolution: the adaptive landscape of spiders without venom glands
Source: Gigascience. 2024 Aug 5;13:giae048. doi: 10.1093/gigascience/giae048 (PMC11299198; doi:10.1093/gigascience/giae048)
Supplement: giae048_Reviewer_2_Report_Revision_1 — Sandra Correa-Garhwal -- 3/7/2024 Reviewed [file giae048_reviewer_2_report_revision_1.pdf]

Reviewer Report

Title: A Trade-off in Evolution: The Adaptive Landscape of Spiders without Venom Glands

Version: Revision 1 **Date:** 3/7/2024

Reviewer name: Sandra Correa-Garhwal

Reviewer Comments to Author:

Zhang et al provide a multi-omics analyses on Octonoba sinensis (Uloboridae) and describe the genetics that could explain an adaptive response to the lack of venom in the group. The revised version of the manuscript is much improved with new data and new conclusions. However, I still have some concerns that need to be addressed before its publication.

1. While the addition of Deinopis transcriptome to help with the gene family analyses is fine, this information needs to be included. For example, there is no mention in Figure 1 what the red dotted line means that leads to Deinopis and why it does not have a circle diagram. Table S4 does not have the NCBI info and Table S18 is labeled as Asianopis and not Deinopis. Given that the authors have added other genomes from RTA spiders I am not sure how much this transcriptome is added since it was not included in other analyses in the study. At this point, if it is not adding relevant information to the story, I would recommend removal. It almost looks like a last minute add on.
2. Figure 2C seems to be out of order to me. While reading it was hard to understand why it was there (other than being a comparative genome analysis) and not in Figure 5.
3. Lines 154-155: expression analyses are not included in the material and methods. This is very important because (1) the reader would need to understand how the expression data was generated: reads mapped to genome? To transcriptome? Using what program? (2) what program and type of normalization was done for the DE analyses? The way these two sentences are written and figure 3 legend, they imply that the authors did DE using P. tepidariorum legs as the control for the legs of O. sinensis. I have never seen a DE across species that way to be able to create a volcano plot as shown on figure 3B. How it was done needs to be included.
4. One major issue I found was the conclusion. From lines 309 to 324 I feel the argument being made is not completely logical and I can't see how it contributes to the manuscript. The discussion should be about the results and conclusions generated in this study and not on whether venom evolved before or after prey-catching webs. Here the authors have a chance to talk about the metabolic related genes that are under positive selection and how it could relate to an increase endurance in O. sinensis. What are the potential functions of the expanded or new emergent gene families. How all of that relates to no having venom glands and using this extensive prey-wrapping technique. All of that is not included in the conclusion as it stands. This section needs to be rewritten.

Here are some other small comments:

5. Line 49: delete "changes"
6. Line 51: instead of "these spiders" use uloborids
7. Line 52: instead of "this group" use Uloboridae
8. L55: add a reference to the first sentence

9. Line 75: delete "in its anatomy"
10. Line78: Suggest changing the title of the section to something like: Prey-wrapping behavior observations and fand morphology
11. Line82: Araneoidea comes out of nowhere. Maybe say "time for other spider species (Arnaeoidea, 9.7+- …"
12. Line 87: provides instead of providing. And "uloborids"
13. Line 90: add "s" to observation
14. Line 91: delete channel (D)
15. Line 92 indicates instead of indicating. Delete "channel"
16. Line 99: spell out 9
17. L126: maybe add that the proportions circles are next to each species
18. Line 156: add "with" after genes and delete "the" after expression
19. Line 171 add "term" after GO
20. Line 172: delete (D-E).
21. Section 5 should include how the authors identified the expanded and new emergent gene families. Just one or two sentences.
22. Section 5 Lines 181-182: could rephrase these sentences to be clearer. For example: In O. sinensis, we found four of these families to have a high number of annotated genes that corresponded to: …
23. Figure 4 shows with very low quality on my copy.
24. In general, when a number has more than 3 digits a comma should be added. For example, Line 243 - instead of 1088 write 1,088. There are too many instances to numbered them all. Please check the document.
25. Line 208: add Species before phylogeny
26. Line234: spell out 5
27. Line 245 delete "gene" after ")"
28. Figure 5 and 6. The expression plots do not indicate the type of values. Are this TPMs? RPKM? Raw reads?
29. Figure 5A legend. The GO enrichment is for which species? Ptep? O sin?
30. Line 258 add "in O. sinensis, we .."
31. Line 260: delete have
32. Line 265: add " in the venom glands of P. tepidariorum"
33. Lines 267-270: these sentences are out of order and hard to follow. It is not clear why the authors are talking about latrotoxin or why an additional gene tree was generated for this venom component and not the other ones. I suggest the authors start with something along the lines of "Given that octonoba had four latrotoxin homologs out of 12 venom components (highest category, Table S14) and latrotoxins are not known outside of Therididae (cob-web weavers) we investigated further." At least something like that would set the reader up for what is coming. Then the results from the gene tree analysis can be explained. Make sure to be clear on the message. As I said the text is confusing.
34. Figure S4: The black text in the figure should be added the figure legend. For the green genes, add the species they come from.
35. Lines 281-290: I think is very interesting that there is conservation in the where these genes are in both species regardless of function. The paragraph will benefit from editing because the results are not

coming thru. I also suggest that the authors describe all genes that fall under this category, I think that there are only three by looking at Figure 6. Given the small number of instances, it seems odd to only describe one of them. These results should also be added to the discussion: we found these genes, they are expressed (or not) in XX tissue, and we think the function in octonoba is XX.
36. Figure 6 The legend that is inside the figure, starting with the asterisk should be included in the legend and maybe not in the figure. It also refers to itself which is odd)
37. Figure 7. What the red line in A means needs to be included in the legend for panel A. Also, include that the different colors (green vs. blue) in the genes for panel B indicate.
38. Line 300: add () to A
39. Line 301: add () to A
40. For the methods, include the type of camera that was used to film the pre-wrapping behavior.
41. Line 393 Additional file 6 has no information related to tissue samples
42. Line 400: replace "have been" with were
43. Line 406 spell out 9. Add "of" after Gb
44. Section 7 - was this methodology used for figure s4? How was that tree generated?
45. How were the cluster analyses done?
46. Line 456 spell out HCEs
47. Line 468: instead of "previous databases" explain what those are. Downloaded sequences from multiple sources (NCBI/Arachnoserver?) that includes multiple species? Or includes venom components from all spiders and other animals?
48. Availability of data: the NCBI code given is not functional and there is an empty parenthesis for the gigaDB Digital Repository.

Methods

Are the methods appropriate to the aims of the study, are they well described, and are necessary controls included? Choose an item.

Conclusions

Are the conclusions adequately supported by the data shown? Choose an item.

Reporting Standards

Does the manuscript adhere to the journal's guidelines on [minimum standards of reporting?] Choose an item.

Choose an item.

Statistics

Are you able to assess all statistics in the manuscript, including the appropriateness of statistical tests used? Choose an item.

Quality of Written English

Please indicate the quality of language in the manuscript: Choose an item.

Declaration of Competing Interests

Please complete a declaration of competing interests, considering the following questions:

- Have you in the past five years received reimbursements, fees, funding, or salary from an organisation that may in any way gain or lose financially from the publication of this manuscript, either now or in the future?
- Do you hold any stocks or shares in an organisation that may in any way gain or lose financially from the publication of this manuscript, either now or in the future?
- Do you hold or are you currently applying for any patents relating to the content of the manuscript?
- Have you received reimbursements, fees, funding, or salary from an organization that holds or has applied for patents relating to the content of the manuscript?
- Do you have any other financial competing interests?
- Do you have any non-financial competing interests in relation to this paper?

If you can answer no to all of the above, write 'I declare that I have no competing interests' below. If your reply is yes to any, please give details below.

I declare that I have no competing interests

I agree to the open peer review policy of the journal. I understand that my name will be included on my report to the authors and, if the manuscript is accepted for publication, my named report including any attachments I upload will be posted on the website along with the authors' responses. I agree for my report to be made available under an Open Access Creative Commons CC-BY license (http://creativecommons.org/licenses/by/4.0/). I understand that any comments which I do not wish to be included in my named report can be included as confidential comments to the editors, which will not be published.

Choose an item.

To further support our reviewers, we have joined with Publons, where you can gain additional credit to further highlight your hard work (see: https://publons.com/journal/530/gigascience). On publication of this paper, your review will be automatically added to Publons, you can then choose whether or not to claim your Publons credit. I understand this statement.

Yes Choose an item.
